# *γ*-Selective C(sp^3^)–H amination via controlled migratory hydroamination

**DOI:** 10.1038/s41467-021-25696-z

**Published:** 2021-09-27

**Authors:** Changseok Lee, Huiyeong Seo, Jinwon Jeon, Sungwoo Hong

**Affiliations:** 1 Department of Chemistry, Korea Advanced Institute of Science and Technology (KAIST), Daejeon, 34141 Korea; 2 Center for Catalytic Hydrocarbon Functionalizations, Institute for Basic Science (IBS), Daejeon, 34141 Korea

**Keywords:** Homogeneous catalysis, Synthetic chemistry methodology

## Abstract

Remote functionalization of alkenes via chain walking has generally been limited to C(sp^3^)–H bonds *α* and *β* to polar-functional units, while *γ*-C(sp^3^)–H functionalization through controlled alkene transposition is a longstanding challenge. Herein, we describe NiH-catalyzed migratory formal hydroamination of alkenyl amides achieved via chelation-assisted control, whereby various amino groups are installed at the *γ*-position of aliphatic chains. By tuning olefin isomerization and migratory hydroamination through ligand and directing group optimization, *γ*-selective amination can be achieved via stabilization of a 6-membered nickellacycle by an 8-aminoquinoline directing group and subsequent interception by an aminating reagent. A range of amines can be installed at the *γ*-C(sp^3^)–H bond of unactivated alkenes with varying alkyl chain lengths, enabling late-stage access to value-added *γ*-aminated products. Moreover, by employing picolinamide-coupled alkene substrates, this approach is further extended to *δ*-selective amination. The chain-walking mechanism and pathway selectivity are investigated by experimental and computational methods.

## Abstract (web summary)

Sequential alkene isomerization and cross-coupling enables remote functionalization, but coupling has been limited to positions at a carbon chain terminus or proximal to a functional group. Here the authors show a method to interrupt isomerization with a nickellacycle, which enables coupling at an atypical, unreactive position.

## Introduction

**Fig. 1:**
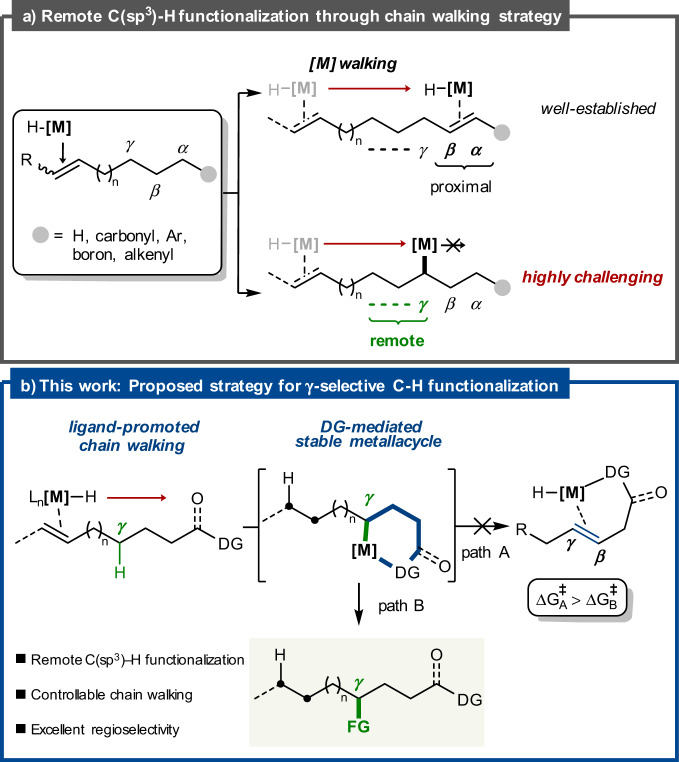
Regioselective migratory hydrofunctionalization of unactivated alkenes. **a** Remote C(*sp*^3^)–H functionalization through chain walking strategy. **b** Proposed strategy for *γ*-selective C(*sp*^3^)–H functionalization.

The site-selective C–H functionalization of electronically and sterically similar aliphatic C–H bonds, ubiquitous in organic molecules, represents a powerful disconnection in chemical synthesis^1,2^. These methods typically require the presence of directing groups in close proximity to the reaction site to govern site selectivity. Recently, migratory cross-coupling reactions for remote C–H functionalization have attracted considerable attention as a powerful alternative strategy that does not require a local directing group^3–8^. For example, remote olefin functionalization via a combination of transition-metal-catalyzed alkene isomerization and cross-coupling chemistry allows for the direct installation of functionalities into inert *sp*^3^ C–H sites that are distant from the alkene initiation point, which would otherwise be difficult to achieve using conventional approaches. Because the driving force of alkene isomerization is the formation of the most thermodynamically favorable alkyl-metal intermediate generated upon chain walking, functionalization with coupling reagents generally occurs at terminal^9–20^ or the C(*sp*^3^)–H bonds *α* to functional groups such as aryl^21–35^, vinyl^36^, oxygen^37–39^, nitrogen^40,41^, boryl^42,43^, and nitrile motifs^44^. Recently, *β*-C(*sp*^3^)–H functionalization has been achieved by leveraging the haptotropic stability^25,45^ and electrophilic character of the *β*-carbon^46^ in *α*,*β*-unsaturated systems or using chelation-assisted strategies^47^. Despite significant advances in the field of alkene isomerization/functionalization, remote functionalization involving chain walking processes has been restricted to the installation of functional groups at C–H bonds *α* or *β* to polar-functional units in the substrate, as chain walking proceeds rapidly to form the most favorable *α-* or *β*-alkyl-metal intermediate prior to the cross-coupling process for product formation (Fig. 1a). In this regard, selective functionalization of remote *γ*-C(*sp*^3^)–H bonds through controllable alkene transposition remains a significant unexplored challenge.

With our objective being the efficient construction of a valuable class of *γ*-aminated building blocks^48^, we were intrigued by the possibility of achieving remote *γ*-selective C(*sp*^3^)–H functionalization of unactivated alkenes. The primary challenge in such an approach is the rapid and reversible chain walking process occurring along the alkyl chain to form the most stable conjugated system in such reactions. We reasoned that an alkyl-metal intermediate at the *γ*-methylene position generated in situ as a result of chain walking must be sufficiently stable to terminate further chain walking^49^. If successful, the versatility of this strategy would provide a synthetically flexible tool for the direct transformation of unactivated C(*sp*^3^)–H bonds at the *γ-*position to carbon-heteroatom bonds.

Recently, alkene functionalization reactions of alkenyl amides bearing the 8-aminoquinoline (AQ) directing group have been developed, in which stable AQ-chelated metallacycles were found to suppress competing *β*-H elimination^48,50–58^. Based on the features of the AQ directing group, we speculated that chain walking along the hydrocarbon chain could be controlled by a chelation-assisted strategy^59,60^ to dictate the regioselectivity of the olefin isomerization and functionalization site. Specifically, we considered whether NiH-catalyzed alkene isomerization^61^ induced by an appropriate external ligand could be interrupted by the thermodynamic stability of an AQ-chelated six-membered metallacycle in preference to *α*,*β*-unsaturated carbonyl intermediates. The resultant six-membered metallacycle could be subsequently intercepted by coupling partners to realize selective *γ*-C(*sp*^3^)–H functionalization (Fig. 1b).

Herein, we present an *γ*-C(*sp*^3^)–H amination strategy achieved via controlled migratory hydroamination of various *δ*,*ε*-, *ε*,*ζ*-, and *ζ*,*η*-alkene substrates under mild conditions, whereby a range of amino groups are installed at the *γ*-position with excellent levels of site selectivity. Moreover, unusual *δ*-selective amination is achieved by employing picolinamide (PA)-coupled alkene substrates to afford synthetically challenging and important chemical building blocks.

## Results

### Reaction discovery and optimization

**Fig. 2:**
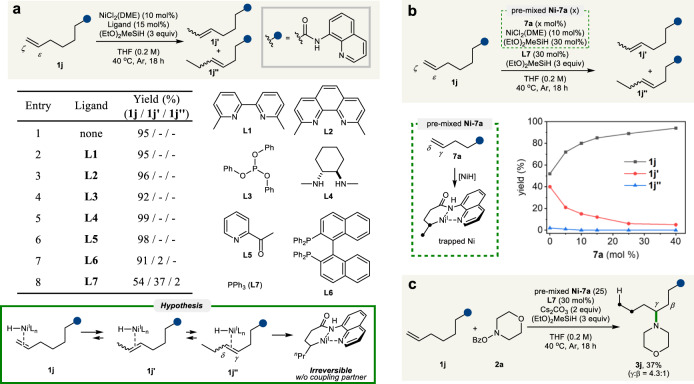
Preliminary results. **a** Ligand screening for alkene isomerization with **1j**; All reactions were performed on a 0.1 mmol scale; Yields were determined by ^1^H NMR spectroscopy; 30 mol% of **L3**, **L5**, **L7** were used; THF: tetrahydrofuran. **b** Sequestration of a nickel catalyst. **c** Migratory hydroamination with a sequestered nickel catalyst.

At the outset, we anticipated challenges with the proposed approach because the initial formation of the Ni-bound bidentate directing complex could retard the kinetics of the chain walking process. Therefore, we first explored the feasibility of alkene isomerization using eight-AQ-coupled alkenamide **1j** by investigating possible ligand candidates (Fig. 2a). Substituted 2,2′-bipyridine (**L1**)^21–24^, 1,10-phenanthroline (**L2**)^34^, triphenylphosphite (**L3**)^59^, and cyclohexanediamine (**L4**)^43^, all of which have previously been employed in nickel-catalyzed migratory functionalization, failed to promote alkene isomerization in the current system. 2-Acetylpyridine (**L5**)^26^, used previously in palladium-catalyzed 1,*n*-arylamination was also tested, but proved to be ineffective. When bisphosphine ligand **L6** was used, only a trace amount of the isomerization product was detected. The Zhu group reported that remote C–H amination can proceed at either terminal or benzylic site with excellent levels of regioselectivity based on ligand control, where the use of monophosphine ligand enabled benzylic-selective C–H amination^24^. Interestingly, the use of triphenylphosphine (**L7**) led to an improved yield of olefin isomer mixtures arising from olefin isomerization, and **1j**′ was obtained in 37% yield, accompanied by the formation of **1j**″ in only 2% of yield (Fig. 2a, entry 8).

Despite extensive optimization using **L7**, however, our attempts to fully isomerize **1j** to the *γ*,*δ*-alkene **1j**″ were unsuccessful, and only partial conversion was observed at a certain point. Considering this unfavorable outcome, we assumed that the thermodynamically stable Ni-bound six-membered complex formed from in situ generated **1j**″ possibly led to the sequestration of the nickel catalyst, thus resulting in cessation of the isomerization process. This consideration prompted us to examine whether the isomerization process could be influenced by the addition of *γ*,*δ*-alkenyl amide **7a**, which readily forms a chelated six-membered metallacycle (Fig. 2b). Upon testing the hypothesis, we found that the addition of only 5% of **7a** significantly inhibited alkene isomerization, and the isomerization process deteriorated in accordance to increasing amounts of **7a**. Considering this process, we speculated that the catalytic cycle could be restored by the participation of the coupling partners to deliver the *γ*-functionalized product and release the free Ni catalyst. Indeed, when *O*-benzoylhydroxylamine **2a** was present as an aminating reagent^62–65^, we were pleased to find that *γ*-aminated product **3j** was readily obtained as a major product along with about 60% of aminated product formed from **7a** in the presence of Ni premixed with 25 mol% of **7a**, thus confirming the validity of our conceptual design (Fig. 2c).

Based on these encouraging preliminary results, we next set out to optimize the migratory hydroamination using *N*-(quinolin-8-yl)-hex-5-enamide (**1a**), and morpholino benzoate (**2a**) as the electrophile in the presence of (EtO)_2_MeSiH as the hydride source (Table 1). After careful evaluation of the reaction parameters, we observed that amination occurred predominantly at the *γ-*position to deliver the desired *γ*-aminated product **3a** with minimal formation of the *β*-aminated product (78%, >20:1; entry 1) by employing NiCl_2_(DME), tris(4-methoxyphenyl)phosphine (**L8**), and Cs_2_CO_3_ (see Supplementary Table 1). Among the solvents screened, the THF/DMA cosolvent system was found to be optimal, with inferior results being obtained with other tested solvents or with the use of only THF or DMA (entries 9 and 10). Of the various nickel precatalysts tested, NiCl_2_(DME) gave rise to optimal results in terms of both reactivity and selectivity (entry 2). As expected, the presence of the ligand influenced the reaction outcome substantially, and a significantly lower yield was obtained in the absence thereof (entry 3). Our evaluation of the electronic effects of substituted phosphine ligands revealed that electron-poor phosphine ligands, such as tris(4-trifluoromethyl)phosphine (**L9**), were less effective in this system, resulting in a decrease in reactivity (entries 4 and 5). The use of bisphosphine ligand **L6** led to a significantly diminished yield of **3a** (entry 6). NiCl_2_(PPh_3_)_2_ was also applicable, being second in order of efficacy (entry 7). Replacement of (EtO)_2_MeSiH with Ph_2_SiH_2_ resulted in a diminished yield (entry 8). Notably, we found that the transformation gave the best results in the presence of Cs_2_CO_3_, with other carbonate counter-cations resulting in decreased reactivity and selectivity (entries 11 and 12). Control experiments revealed that the reaction did not proceed in the absence of either catalyst or silane (entry 13).

**Table 1:** Optimization of the reaction conditions.


Entry	Deviation from standard conditions	Yield (%)^a^	*γ*:*β*
1	None	78 (74)^b^	>20:1
2	NiBr_2_(DME), Ni(acac)_2_, Ni(COD)_2_	67–77	9:1–20:1
3	w/o ligand	24	>20:1
4	**L7**, instead of **L8**	66	10:1
5	**L9**, instead of **L8**	49	>20:1
6^c^	**L6**, instead of **L8**	34	>20:1
7	NiCl_2_(PPh_3_)_2_, instead of NiCl_2_(DME), **L8**	66	10:1
8	Ph_2_SiH_2_, instead of (EtO)_2_MeSiH	43	>20:1
9	THF only	72	12:1
10	DMA only	59	5.7:1
11	K_2_CO_3_, instead of Cs_2_CO_3_	22	1.8:1
12	Na_2_CO_3_, Li_2_CO_3_, no base	trace	-
13	w/o catalyst or silane	0	-

Reaction conditions: **1a** (0.1 mmol), **2a** (0.2 mmol), NiCl_2_(DME) (10 mol%), **L8** (30 mol%), base (0.2 mmol), hydride source (0.3 mmol), and solvent (0.5 mL) at 40 °C under Ar for 18 h.

*DMA:N*,*N*-dimethylacetamide.

^a^Yields and product isomer ratios (γ:β) were determined by ^1^H NMR spectroscopy.

^b^1 mmol scale.

^c^Ligand (15 mol%) was used.

### Substrate scope studies

**Fig. 3:**
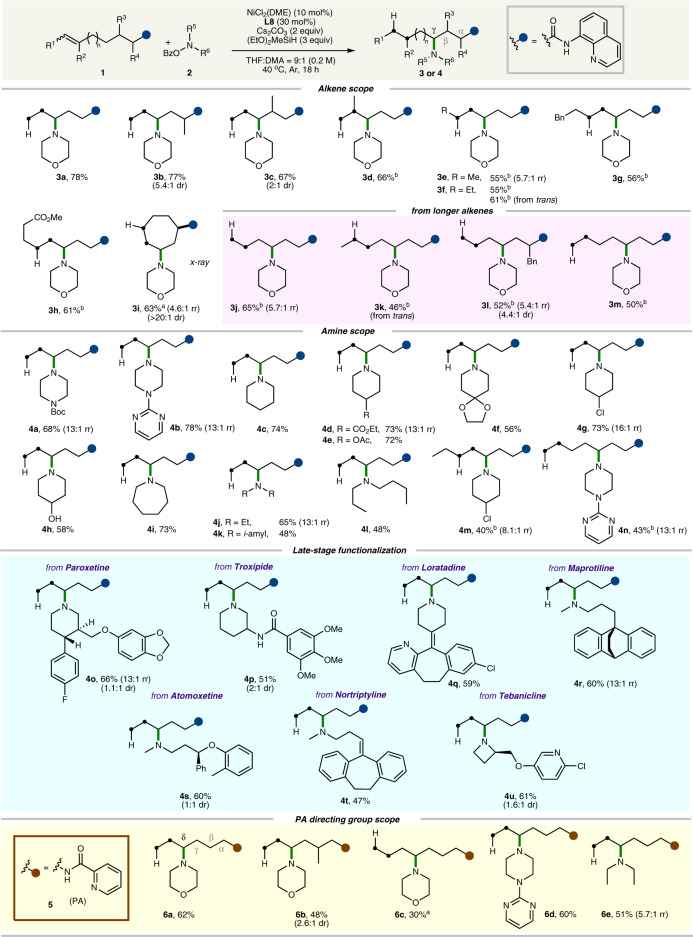
Substrate scope. Reaction conditions: **1** or **5** (0.1 mmol), **2** (0.2 mmol), NiCl_2_(DME) (10 mol%), **L8** (30 mol%), Cs_2_CO_3_ (0.2 mmol) and (EtO)_2_MeSiH (0.3 mmol) in THF/DMA = 9:1 (0.5 mL) at 40 °C under Ar for 18 h. *cis*-Alkenes were used if not denoted otherwise. Yields of isolated *γ*-product. Isomeric ratios (*γ*:*β*) were determined by ^1^H NMR analysis of the crude mixture (>20:1 rr if not denoted otherwise). ^a^**2** (2.5 equiv) was used at 60 °C. ^b^NiCl_2_(DME), **2** and (EtO)_2_MeSiH were added at 60 °C in two batches of 5 mol%, 1.25 equiv and 1.5 equiv, respectively.

With the optimized conditions in hand, we investigated the generality of the Ni-catalyzed migratory hydroamination. Gratifyingly, a diverse range of alkene structures were found to be compatible with this protocol, providing excellent site selectivity (>20:1 rr if not denoted otherwise) and reactivity, as summarized in Fig. 3. First, *δ*,*ε*-unsaturated alkene substrates with diverse methyl substitution patterns afforded the desired *γ*-selective products with excellent regioselectivity (**3b**–**3e**). Importantly, exploration of the alkene scope revealed that not only terminal olefins but also internal olefins engaged in the reaction successfully to afford corresponding products **3e** and **3f**. In addition, various functional groups on the internal alkene, such as benzyl and ester groups, were well tolerated under the optimized conditions (**3g** and **3h**). The intriguing synthetic value of this protocol was further demonstrated by the distinct preference for the *γ*-position over the benzylic position in the alkyl chain (**3g**) without the occurrence of olefin migration into the benzylic position, as previously reported^21–35^. Employing a *δ*,*ε*-cyclic alkene with a cycloheptene motif resulted in the formation of a synthetically useful building block (**3i**) (confirmed by X-ray crystallographic analysis, see the Supplementary information, Appendix II). The preparation of cyclohept-4-enecarboxylic acid is more efficient than the corresponding *γ*,*δ*-unsaturated alkene substrate^66^. Therefore, the synthetic utility of this approach is highlighted to construct target molecule **3i** by employing cyclohept-4-enecarboxylic acid as a substrate. Moreover, expanding the scope from *δ*,*ε*-alkenes to a variety of alkenes with an increasingly distal amide functionality broadened the synthetic utility of the current method. For example, the reaction with *ε*,*ζ*-alkenes furnished the desired *γ*-selective products (**3j**) with excellent regiocontrol. Similarly, *ε*,*ζ*-alkenes bearing methyl (**3k**) and benzyl (**3l**) groups in the alkane chain also readily participated in this reaction. Importantly, beyond the *ε*,*ζ*-alkenes, the current strategy could be extended to *ζ*,*η*-alkene substrates with an excellent degree of selectivity for the *γ* position (**3m**). We next explored the scope of amine electrophiles and established that the protocol could be successfully applied to various amine sources. Piperazine derivatives bearing a Boc (**4a**) or pyrimidine (**4b**) group, often found in biologically active compounds, could be well accommodated in this reaction. Amine sources bearing an ester (**4d** and **4e**) or acetal (**4f**) group were successfully employed to furnish the desired products. Notably, we observed that amine sources containing a chloride (**4g**) and hydroxyl (**4h**) group were well tolerated, providing a handle for further synthetic transformations. In addition, our method could be expanded to substrates containing seven-membered cyclic amine (**4i**) and acyclic amines (**4j–4l**) moieties. When primary amine electrophile such as *O*-benzoyl-*N*-(*tert*-butyl)hydroxylamine was used, less than 20% of the desired products were obtained. To further extend the versatility of our reaction, we evaluated the reactivity of an internal *δ*,*ε*-alkene, which enabled the incorporation of 4-chloropiperidine (**4m**) at the *γ*-position. Furthermore, pyrimidine-bearing piperazine could also be incorporated into the *γ*-position when an *ζ*,*η*-alkene was employed to furnish the corresponding desired product **4n**. To further highlight the utility of the present method, we investigated the late-stage modification of complex and biologically active compounds, as outlined in Fig. 3. The amine moiety present in several complex substances could be successfully coupled with *δ*,*ε*-alkenyl amides to afford the desired *γ*-selective products with excellent site selectivity. As this method enables rapid access to various amine-substituted *γ*-acid derivatives, it provides ready access to medicinally relevant complex *γ*-amino acid derivatives. Specifically, a series of amine-based pharmaceuticals, including paroxetine (**4o**), troxipide (**4p**), loratadine (**4q**), maprotiline (**4r**), atomoxetine (**4s**), and nortriptyline (**4t**), were efficiently employed to afford the corresponding products with excellent regiocontrol and functional group tolerance. Notably, both aryl fluoride and chloride were found to be compatible during the reactions. Furthermore, the structurally strained azetidine in the analgesic tebanicline could be effectively installed at the *γ*-position to afford the desired product **4u**.

As amines are ubiquitous in organic molecules, we next sought to extend the scope with respect to amine-derived PA alkene substrates to broaden the utility of the unified protocol. We observed that the current strategy could be successfully applied to *ε*,*ζ*-unsaturated PA alkenes to access *δ*-amination products (**6a** and **6b**) with excellent site selectivity (>20:1) under the standard conditions. Furthermore, a longer *ζ*,*η*-alkene substrate bearing a PA group proved suitable, enabling *δ*-selective amination (**6c**). In addition, the use of various amine electrophiles, such as pyrimidine-bearing piperazine (**6d**) and acyclic amine (**6e**) afforded the corresponding products.

### Control experiments and mechanistic studies

**Fig. 4:**
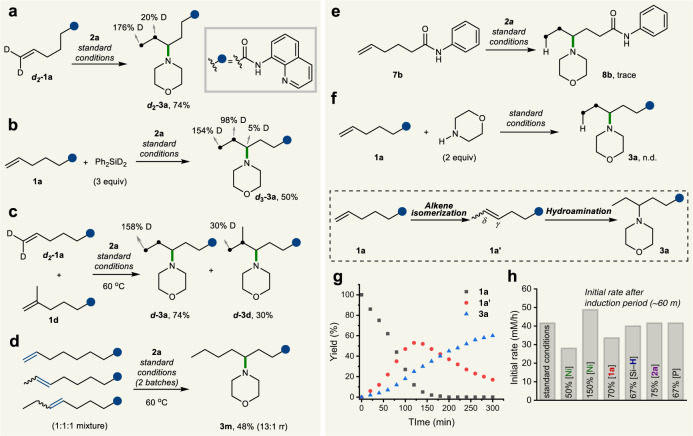
Deuterium labeling studies and control experiments. **a** Deuterium migration experiment. **b** Deuteration with Ph_2_SiD_2_. **c** Crossover experiment. **d** Regioconvergent experiment. **e** Monodentate directing group. **f** Free amine as an coupling partner. **g** Time profiling experiment. **h** Summary of different-excess experiment.

To acquire further insights into the migratory process, a series of deuterium labeling experiments were performed, as shown in Fig. 4a–c. First, when geminally dideuterated alkene ***d***_***2***_**-1a** was subjected to the standard conditions, the incorporation of deuterium at the *δ* (0.20D) and *ε* (1.76D) positions was observed (Fig. 4a). Moreover, when an isotope labeling experiment was performed with Ph_2_SiD_2_ instead of (EtO)_2_MeSiH, the corresponding product ***d***_***3***_**-3a** was obtained in 50% yield with deuterium incorporation at the *γ* (0.05D), *δ* (0.98D), and *ε* (1.54D) positions (Fig. 4b). These results collectively support that the NiH catalyst migrates along the alkyl chain via iterative *β*-H elimination and reinsertion processes. In particular, the sum of deuterium incorporation in ***d***_***3***_**-3a** was above 1.0, giving a strong indication of rapid dissociation and reassociation processes of free NiH/NiD. Subsequently, we conducted a crossover experiment with ***d***_***2***_**-1a** and **1d** as alkene substrates to afford deuterium-incorporated products ***d*-3a** and ***d*-3d**, respectively (Fig. 4c), further supporting the dissociation of NiH/NiD species from the NiH/NiD-alkene complex throughout the relay process in this transformation. The method is highly regioconvergent, and the *γ*- aminated product **3m** (*γ:β* = 13:1) was obtained from isomeric mixtures of three olefins, as shown in Fig. 4d. When the reaction was performed with a substrate bearing an aniline-derived directing group (**7b**), only trace amounts of the product could be detected, demonstrating the importance of the bidentate directing group (Fig. 4e). We next confirmed that the free amine species generated via direct reduction under metal hydride conditions^67^ did not serve as the amine source (Fig. 4f). We next performed kinetic analysis to gain further information about the reaction mechanism (Fig. 4g, h). The time course of the reaction showed alkene isomerization to proceed at a faster rate than the hydroamination process, suggesting that the hydroamination step contributes significantly to the overall reaction rate. Next, we measured the fractional order of NiCl_2_(DME) and **1a** by conducting different-excess experiments^68^, thus suggesting the off-cycle formation of NiH dimers^48^ as the catalyst resting state (Supplementary Fig. 11).

**Fig. 5:**
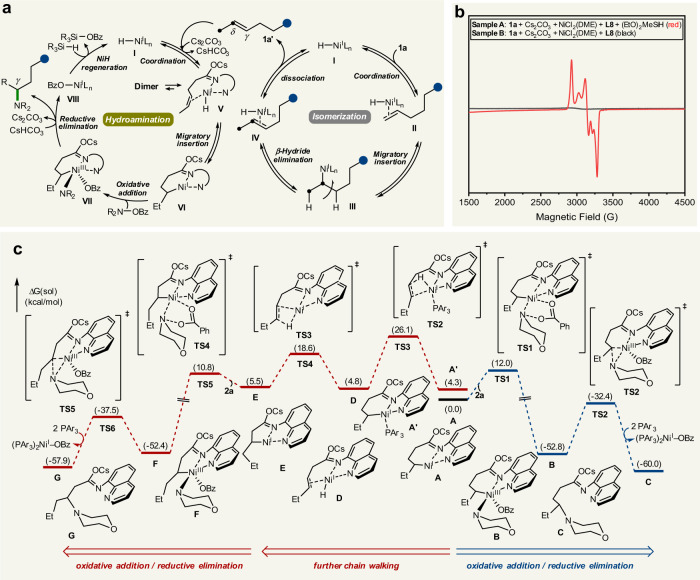
Proposed reaction mechanism and DFT calculation result. **a** Proposed reaction mechanism. **b** EPR spectra of paramagnetic Ni(I) complex. **c** Free energy profile from nickellacyle. Geometry, vibrational frequency, and solvation energy were calculated using the B3LYP-D3 and LACVP**, and B3LYP-D3 and cc-pVTZ(-f)/LACV3P** were employed to determine single-point energy.

Based on the aforementioned mechanistic studies, a plausible pathway for this remote C–H amination is illustrated in Fig. 5a. Active nickel(I) hydride species **I**, formed from the combination between the nickel precatalyst and hydrosilane^69,70^, inserts into the olefin to generate alkyl-nickel intermediate **III**, which undergoes reversible iterative chain walking along the alkyl chain to eventually form isomeric *γ*,*δ*-alkene intermediate **IV**. At this stage, ligand exchange from the phosphine ligand to the AQ directing group, followed by a migratory insertion, leads to nickellacycle **V** in preference to other alkyl-nickel species, providing a driving force for chain walking. Controlled by the stability of the AQ-chelated six-membered complex, selective cross-coupling with an aminating reagent readily proceeds through oxidative addition and reductive elimination processes to afford the desired *γ*-aminated product along with L_n_Ni(I)–OBz (**VIII**). Active catalyst **I** is then regenerated by hydrosilane to complete the catalytic cycle for the migratory hydroamination reaction. Our DFT calculations revealed that the oxidative addition of complex **A** with **2a** is a reasonable reaction pathway with a moderate barrier of 12.0 kcal/mol and is exergonic by −52.8 kcal/mol to give rise to thermodynamically stable intermediate **B** (Fig. 5c). On the other hand, *β*-hydride elimination of **A** leading to intermediate **D** occurs with a higher energy barrier of 26.1 kcal/mol. These results were consistent with the experimentally observed site selectivity.

In addition to our DFT studies, we conducted EPR experiments to support that Ni(I) species participate in this catalytic system. First, the reaction mixtures in the absence of an electrophile (sample **A**) resulted in a new EPR signal while this signal disappeared when hydrosilane was not added (sample **B**), as shown in Fig. 5b. To observe the ligand environment of the Ni(I) species, we further conducted EPR measurements with control sample **C** (**1a'**, base, NiCl_2_(DME), and hydrosilane) and sample **D** (**L8**, NiCl_2_, and hydrosilane) (Supplementary Figs. 7 and 8). As expected, the ligand employed influenced the outcome of the EPR spectra. The EPR spectrum of sample **C** (Supplementary Fig. 8) exhibited a similar signal to that of sample **A**, suggesting the intermediacy of the Ni(I) complex with 8-AQ under the standard reaction conditions.

## Discussion

In summary, we have developed a NiH-catalyzed remote *γ*-C(*sp*^3^)–H amination protocol that proceeds via a controlled migratory hydroamination relay process. A wide range of amines can be selectively installed at the *γ*-position of various aliphatic chains of alkenes under mild reaction conditions with good functional group tolerance, providing efficient access to a series of value-added *γ*-aminated products in a late-stage fashion. Chain walking occurs to translocate the alkyl-nickel complex to the *γ*-position, enabled by AQ directing group stabilization of the six-membered nickellacycle, subsequently intercepted by an aminating reagent. The current method constitutes a unique approach for achieving various *γ*-C(*sp*^3^)–H amination disconnections in organic synthesis. We believe that this study has potential application in the further development of site-selective remote C–H functionalization of significant synthetic utility.

## Methods

### Representative procedure for the migratory hydroamination of unactivated alkenes

To a flame-dried 12 mL test tube equipped with a Teflon-coated magnetic bar were added *N*-(quinolin-8-yl)hex-5-enamide (**1a**) (24.0 mg, 0.10 mmol), morpholino benzoate (**2a**) (41.4 mg, 0.20 mmol), and tris(4-methoxyphenyl)phosphine (10.6 mg, 0.030 mmol). The test tube was sealed with a PTFE/silicone septa cap, which was pierced by a 22-gauge needle. The sealed test tube was placed into an argon-filled glovebox. In glovebox, Cs_2_CO_3_ (65.2 mg, 0.20 mmol) and NiCl_2_(DME) (2.2 mg, 0.010 mmol) were added to the test tube. The reaction mixture was diluted with THF:DMA = 9:1 (0.5 mL, 0.2 M) and stirred at room temperature for 5 min. After the addition of (EtO)_2_MeSiH (48.1 μL, 0.30 mmol), the reaction test tube was sealed with a septa cap, and removed from the glovebox. The reaction mixture was stirred at 40 °C for 18 h. The reaction mixture was monitored by TLC using EA:Hx = 1:1 (Rf = 0.3) as the mobile phase. After the disappearance of starting material, the reaction mixture was diluted with 50 mL of ethyl acetate and washed with aqueous NaHCO_3_ (2 × 25 mL) and brine (25 mL). The combined organic layer was dried over Na_2_SO_4._ After removal of solvent, the residue was purified by flash column chromatography on silica gel (EA:Hx = 1:1 to MeOH:EA:Hx = 1:9:10) to give a desired product compound **3a** (25.6 mg, 78%) as yellowish oil.

## Supplementary information

Supplementary Information

Description of Additional Supplementary Files

Supplementary Data 1

Supplementary Data 2

Supplementary Data 3

Supplementary Data 4

## Data Availability

Experimental procedure and characterization data of new compounds are available within Supplementary Information. Computational details, optimized Cartesian coordinates of all structures, vibrational frequencies, and energy components. This material is available free of charge via the Internet. The X-ray crystallographic coordinate for structure **3i** has been deposited at the Cambridge Crystallographic Data Centre (CCDC) under the deposition number CCDC 2071903, and can be obtained free of charge from The Cambridge Crystallographic Data Centre via www.ccdc.cam.ac.uk/data_request/cif. All other requests for materials and information are available from the corresponding author upon reasonable request.
