# Supplementary material for: γ-Selective C(sp3)–H amination via controlled migratory hydroamination
Source: Nat Commun. 2021 Sep 27;12:5657. doi: 10.1038/s41467-021-25696-z (PMC8476554; doi:10.1038/s41467-021-25696-z)
Supplement: Supplementary file 1 — Supplementary Information [file 41467_2021_25696_MOESM1_ESM.pdf]

Supplementary Information

γ-Selective C(sp^3)–H Amination via Controlled Migratory Hydroamination

Lee et al.

Supplementary Methods

General Information.

Unless stated otherwise, reactions were performed in flame-dried glassware. Analytical thin layer chromatography (TLC) was performed on precoated silica gel 60 F^{254} plates and visualization on TLC was achieved by UV light (254 and 365 nm). Flash column chromatography was performed on silica gel (400-630 mesh) or a Combi*Flash*® R_f^+ system with Redi*Sep*® R_f silica columns (230-400 mesh) using a proper eluent. ^{1}H NMR was recorded on Bruker Avance 400 MHz or Agilent Technologies DD2 600 MHz and chemical shifts were quoted in parts per million (ppm) referenced to the appropriate solvent peak (7.26 ppm for $CDCl_3$ or 5.32 ppm for CD_2Cl_2). The following abbreviations were used to describe peak splitting patterns when appropriate: br = broad, s = singlet, d = doublet, t = triplet, q = quartet, m = multiplet, dd = doublet of doublet, td = triplet of doublet, ddd = doublet of doublet of doublet. Coupling constants, *J*, were reported in hertz unit (Hz). ^{13}C NMR was recorded on Bruker Avance 100 MHz or Agilent Technologies DD2 150 MHz and was fully decoupled by broad band proton decoupling. Chemical shifts were reported in ppm referenced to the center line of appropriate solvent peak (triplet at 77.16 ppm of $CDCl_3$ at pentet at 54.0 ppm of CD_2Cl_2). ^{19}F NMR was recorded on Bruker Avance 376 MHz and was fully decoupled by broad band proton decoupling. High-resolution mass spectras were obtained by using EI or FAB method from Korea Basic Science Institute (Daegu) or ESI method from KAIST Research Analysis Center (Daejeon). Data collections of parabar-oil-coated single crystal (**3i**) were carried out on a Bruker D8 Quest diffractometer equipped with a monochromator in the Mo Kα radiation and PHOTON 2 area detector. The diffraction data was integrated, scaled, and reduced by using the Bruker APEX3 software. The structure was solved and refined using SHELX programs. X-band CW electron paramagnetic resonance (EPR) spectroscopy was performed using Bruker EMXplus spectrometer equipped with standard resonator. Commercial grade reagents and solvents were used without further purification except as indicated below. Cesium carbonate was dried (120 °C) for 18 hrs under vacuum and stored in glove box.

Procedures for the Substrate Synthesis.

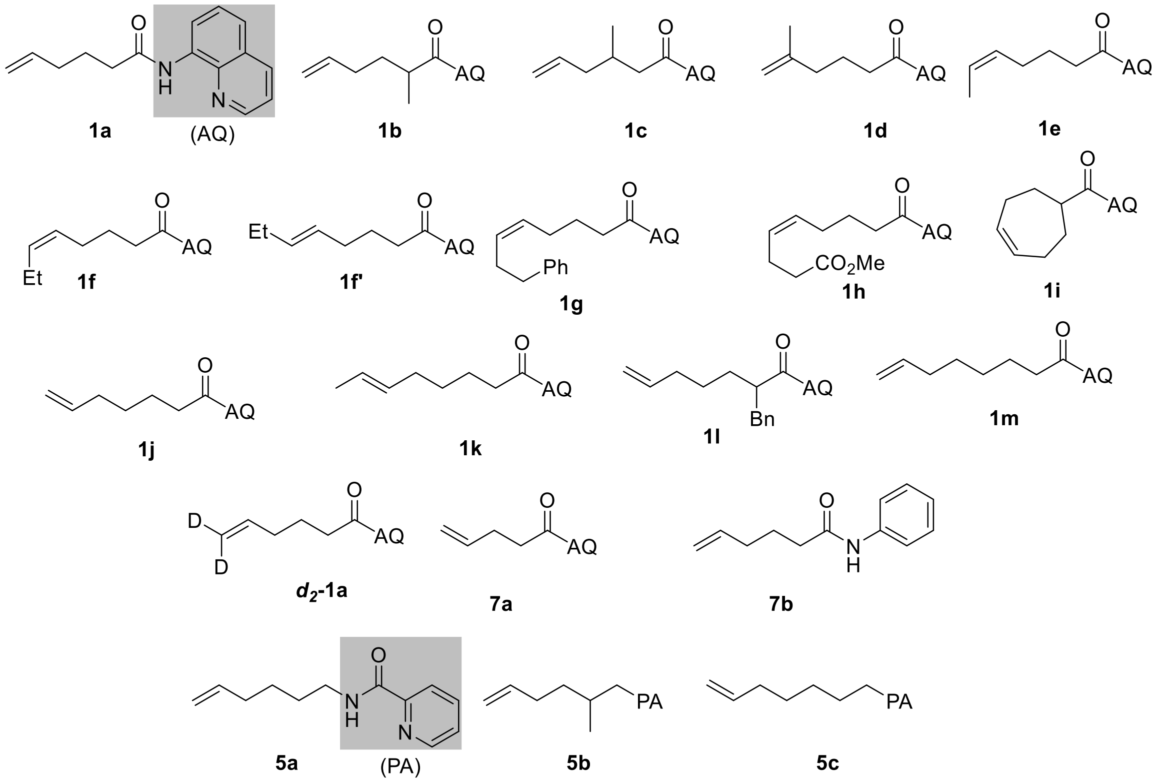

Preparation of alkene substrates except **1b**, **1c**, **1d**, **1e**, **1f**, **1f'**, **1g, 1h, 1i, 1k, 1l, 1m,** ***d*$_2$-1a, 5b** and **5c** was based on literature methods.[1–4]

The procedure to synthesize of **1b**, **1c**, **1d**, **1e**, **1f**, **1f'**, **1g, 1h, 1i, 1k, 1l, 1m,** ***d*$_2$-1a, 5b** and **5c** is shown below based on the modified literature methods.[5-16]

Preparation of substituted *δ,ε*-alkene substrate (1b)

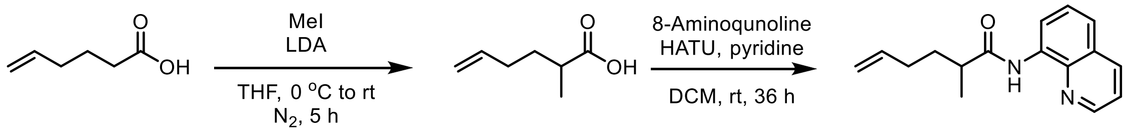

α-Methylation of acid[5]

To a solution of hex-5-enoic acid (0.60 mL, 5.0 mmol) in dry THF (10.0 mL, 0.5M) was added 1.0 M LDA solution in THF (11.0 mL, 11.0 mmol) dropwisely at -78 °C under N_2 atmosphere. The reaction mixture was slowly warmed to 0 °C and stirred for 40 min. To a reaction mixture was added iodomethane (0.31 mL, 5.0 mmol) at 0 °C and stirred for 30 min. Then, the reaction mixture was stirred at room temperature for 3h. The reaction mixture was quenched with water (20 mL) dropwisely. After the removal of organic layer, the pH value of the aqueous layer was adjusted to 2.0 with HCl (1 N) and the aqueous layer was extracted with ethyl acetate (2 × 30 mL). The combined organic layer was washed with water (30 mL) and brine (30 mL) and dried over Na_2SO_4. After the removal of the solvent, the crude mixture was used in the next step without further purification.

Amide bond formation[6]

To a solution of 2-methylhex-5-enoic acid (769 mg, 5.0 mmol) in DCM (15.0 mL, 0.33 M) were added 8-aminoquinoline (571 mg, 5.0 mmol), pyridine (0.49 mL, 6.0 mmol) and HATU (2.28 g, 6.0 mmol). The reaction mixture was stirred at room temperature for 36 h. The reaction mixture was monitored by TLC using EA:Hx = 1:3 as the mobile phase. After disappearance of starting material, the reaction mixture was concentrated under reduced pressure. The residue was diluted with ethyl acetate (30 mL) and washed with aqueous $NaHCO_3$ (3 × 50 mL) and brine (50 mL). The combined organic layer was dried over Na_2SO_4. After the removal of solvent, the residue was purified by flash chromatography on silica gel (ethyl acetate:hexane = 1:20) to give 2-methyl-*N*-(quinolin-8-yl)hex-5-enamide as colorless oil (619 mg, 48% for 2 steps).

^{1}H NMR (600 MHz, $CDCl_3$) δ 9.85 (s, 1H), 8.91 – 8.65 (m, 2H), 8.15 (dd, *J* = 8.2, 1.7 Hz, 1H), 7.53 (t, *J* = 7.9 Hz, 1H), 7.49 (dd, *J* = 8.3, 1.5 Hz, 1H), 7.45 (dd, *J* = 8.3, 4.2 Hz, 1H), 5.86 – 5.73 (m, 1H), 5.05 (dq, *J* = 17.1, 1.7 Hz, 1H), 4.98 (dq, *J* = 10.1, 1.4 Hz, 1H), 2.63 (dt, *J* = 8.1, 6.5 Hz, 1H), 2.21 – 2.12 (m, 2H), 2.00 – 1.90 (m, 1H), 1.63 (ddt, *J* = 13.6, 8.6, 6.9 Hz, 1H), 1.32 (d, *J* = 6.9 Hz, 3H). ^{13}C NMR (100 MHz, $CDCl_3$) δ 175.3, 148.3, 138.6, 138.2, 136.5, 134.7, 128.1, 127.6, 121.7, 121.5, 116.6, 115.4, 77.5, 77.2, 76.8, 42.3, 33.6, 31.7, 18.1. HRMS (EI$^+$) m/z calcd. For $[C_{16}H_{18}N_2O]^+$: 254.1419, found : 254.1417.

Preparation of substituted *δ,ε*-alkene substrate (1c)

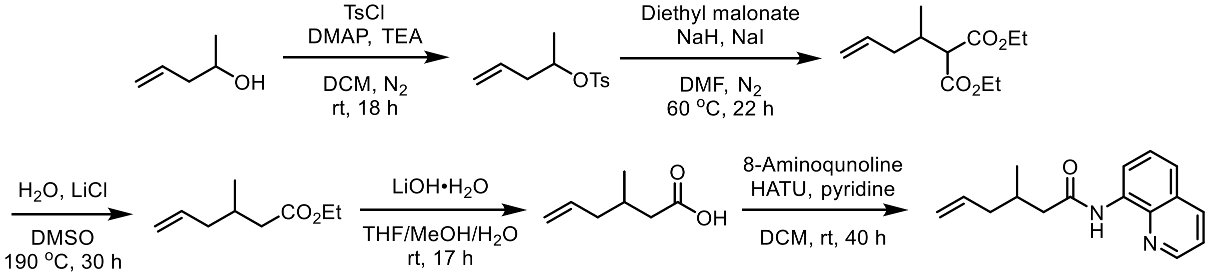

Tosylation

To tosyl chloride (4.20 g, 22.0 mmol) and DMAP (0.61 g, 5.0 mmol) in dry DCM (50.0 mL) was added triethylamine (5.58 mL, 40.0 mmol) under N_2 atmosphere and the reaction mixture was cooled to 0 °C. To the reaction mixture was added pent-4-en-2-ol (2.06 mL, 20.0 mmol), which is diluted with dry DCM (50.0 mL), dropwisely and the mixture was stirred at room temperature overnight. The reaction mixture was monitored by TLC using EA:Hx = 1:2 as the mobile phase. After disappearance of starting material, the reaction mixture was washed with aqueous $NaHCO_3$ (1 × 100 mL) and water (100 mL). The combined aqueous layer was extracted with CH_2Cl_2 (3 × 200 mL). The combined organic layer was dried over Na_2SO_4. After the removal of solvent, the residue was purified by flash chromatography on silica gel (ethyl acetate:hexane = 1:20) to give pent-4-en-2-yl 4-methylbenzenesulfonate as colorless

oil (4.20 g, 88%)

Alkylation of malonic ester[7]

To diethyl malonate (2.92 mL, 19.2 mmol) in dry DMF (70.0 mL, 0.25 M) was added NaH (60% in mineral oil, 0.770 g, 19.2 mmol) at 0 °C under N_2 atmosphere. The mixture was allowed to warm to room temperature and stirred for 30 min. The mixture was cooled to 0 °C and to the reaction mixture were added alkyl tosylate (4.2 g, 17.5 mmol) dropwisely and NaI (2.6 g, 17.5 mmol). The reaction mixture was allowed to warm to room temperature and stirred for 22 h. The reaction mixture was monitored by TLC using EA:Hx = 1:20 as the mobile phase. After disappearance of starting material, The reaction mixture was quenched with water (80 mL) at 0 °C and extracted with diethyl ether (3 × 100 mL). The combined organic layer was washed with saturated NH_4Cl solution (2 × 300 mL) and brine (300 mL), dried over Na_2SO_4. After the removal of solvent, the residue was purified by flash chromatography on silica gel (ethyl acetate:hexane = 1:20) to give diethyl 2-(pent-4-en-2-yl)malonate as colorless oil (3.99 g, quant.).

Krapcho decarboxylation[8]

To diethyl 2-(pent-4-en-2-yl)malonate (3.99 g, 17.5 mmol) in DMSO (70.0 mL, 0.25 M) was added LiCl (1.48 g, 35.0 mmol) and H_2O (0.63 mL, 35.0 mmol). The mixture was heated to 190 °C for 30 h. The reaction mixture was monitored by TLC using EA:Hx = 1:9 as the mobile phase. After disappearance of starting material, the reaction mixture was quenched with saturated NH_4Cl solution (100 mL) at room termperature and extracted with diethyl ether (3 × 100 mL). The combined organic layer was washed with water (2 × 100 mL) and brine (100 mL), dried over Na_2SO_4. After the removal of solvent, the crude mixture was used in the next step without further purification.

Ester hydrolysis[9]

To a solution of ethyl 3-methylhex-5-enoate (1.56 g, 10.0 mmol) in THF:H_2O:MeOH = 1:1:1 (40.1 mL, 0.25 M) was added lithium hydroxide monohydrate (840 mg, 20.0 mmol). The reaction mixture was stirred at room temperature for 17 h. The reaction mixture was concentrated under reduced pressure and diluted with distilled water (50 mL). The aqueous layer was acidified with 1 N HCl at 0 °C and extracted with DCM (3 × 50 mL). The combined organic layer was dried over Na_2SO_4 and concentrated under reduced pressure. After the removal of solvent, the crude mixture was used in the next step without further purification.

Amide bond formation[6]

To a solution of 3-methylhex-5-enoic acid (920 mg, 7.18 mmol) in DCM (18.1 mL, 0.33 M) were added 8-aminoquinoline (863 mg, 5.98 mmol), pyridine (968 μL, 12.0 mmol) and HATU (2.73 g, 7.18 mmol).

The reaction mixture was stirred at room temperature for 40 h. The reaction mixture was monitored by TLC using EA:Hx = 1:9 as the mobile phase. After disappearance of starting material, the reaction mixture was concentrated under reduced pressure. The residue was diluted with ethyl acetate (30 mL) and washed with aqueous $NaHCO_3$ (3 × 30 mL) and brine (30 mL). The combined organic layer was dried over Na_2SO_4. After the removal of solvent, the residue was purified by flash chromatography on silica gel (ethyl acetate:hexane = 1:9) to give 3-methyl-*N*-(quinolin-8-yl)hex-5-enamide as yellowish oil (666 mg, 15% for 3 steps).

^1H NMR (600 MHz, $CDCl_3$) δ 9.79 (s, 1H), 8.87 – 8.70 (m, 2H), 8.16 (dd, *J* = 8.2, 1.7 Hz, 1H), 7.56 – 7.48 (m, 2H), 7.46 (dd, *J* = 8.3, 4.2 Hz, 1H), 5.85 (ddt, *J* = 17.2, 10.1, 7.1 Hz, 1H), 5.12 – 5.00 (m, 2H), 2.60 (dd, *J* = 14.0, 5.8 Hz, 1H), 2.35 (dd, *J* = 14.1, 8.1 Hz, 1H), 2.32 – 2.26 (m, 1H), 2.25 – 2.17 (m, 1H), 2.14 – 2.07 (m, 1H), 1.07 (d, *J* = 6.5 Hz, 3H). ^{13}C NMR (100 MHz, $CDCl_3$) δ 171.3, 148.3, 138.5, 136.7, 136.5, 134.7, 128.1, 127.6, 121.7, 121.5, 116.8, 116.6, 77.5, 77.2, 76.8, 45.2, 41.2, 30.8, 19.8. HRMS (EI^+) m/z calcd. For $[C_{16}H_{18}N_2O]^+$: 254.1419, found : 254.1418.

Preparation of substituted *δ,ε*-alkene substrate (1d)

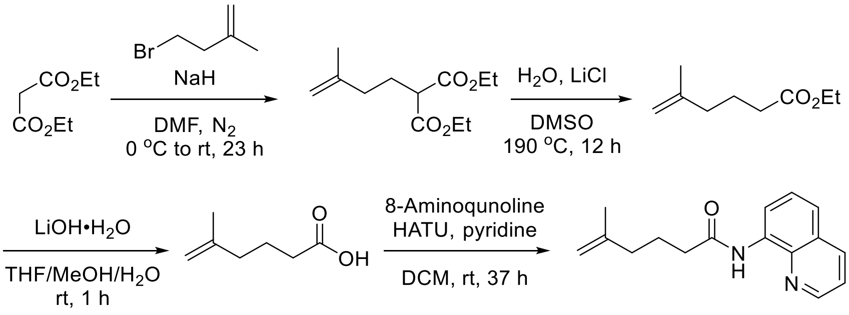

Alkylation of malonic ester[7]

To diethyl malonate (1.34 mL, 8.8 mmol) in dry DMF (32.0 mL, 0.25 M) was added NaH (60% in mineral oil, 0.352 g, 8.8 mmol) at 0 °C under N_2 atmosphere. The mixture was allowed to warm to room temperature and stirred for 30 min. The mixture was cooled to 0 °C and alkyl bromide (1.00 mL, 8.0 mmol) was added dropwise. The reaction mixture was allowed to warm to room temperature and stirred for 23 h. The reaction mixture was quenched with saturated NH_4Cl solution (60 mL) at 0 °C and extracted with diethyl ether (3 × 50 mL). The combined organic layer was washed with saturated NH_4Cl solution (2 × 50 mL) and brine (50 mL), dried over $MgSO_4$. After the removal of solvent, the residue was purified by flash chromatography on silica gel (Ethyl acetate:hexane = 1:14) to give diethyl 2-(3-methylbut-3-en-1-yl)malonate as colorless oil (1.06 g, 58%).

Krapcho decarboxylation[8]

To diethyl 2-(3-methylbut-3-en-1-yl)malonate (1.06 g, 4.7 mmol) in DMSO (18.0 mL, 0.25 M) was added LiCl (0.394 g, 9.3 mmol) and H_2O (0.168 mL, 9.3 mmol). The mixture was heated to 190 °C for

12 h. After the reaction mixture was quenched with saturated NH_4Cl solution (50 mL) at room termperature and extracted with diethyl ether (3 × 50 mL). The combined organic layer was washed with water (2 × 50 mL) and brine (50 mL), dried over $MgSO_4$. After the removal of solvent, the residue was purified by flash chromatography on silica gel (ethyl acetate:hexane = 1:9) to give ethyl 5-methylhex-5-enoate as colorless oil (135 mg, 19%).

Ester hydrolysis[9]

To a solution of ethyl 5-methylhex-5-enoate (135 mg, 0.86 mmol) in THF:H_2O:MeOH = 1:1:1 (3.6 mL, 0.25 M) was added lithium hydroxide monohydrate (180 mg, 4.3 mmol). The reaction mixture was stirred at room temperature for 1 h. The reaction mixture was concentrated under reduced pressure and diluted with distilled water (25 mL). The aqueous layer was acidified with 1 N HCl at 0 °C and extracted with DCM (3 × 25 mL). The combined organic layer was dried over Na_2SO_4 and concentrated under reduced pressure to give 5-methylhex-5-enoic acid as colorless oil (108 mg, 98%) . The residue was used in the next step without further purification.

Amide bond formation[6]

To a solution of 5-methylhex-5-enoic acid (108 mg, 0.84 mmol) in DCM (4 mL, 0.2 M) were added 8-aminoquinoline (122 mg, 0.84 mmol) pyridine (140 μL, 1.7 mmol) and HATU (479 mg, 1.3 mmol). The reaction mixture was stirred at room temperature for 37 h. The reaction mixture was monitored by TLC using EA:Hx = 1:9 as the mobile phase. After disappearance of starting material, the reaction mixture was concentrated under reduced pressure. The residue was diluted with ethyl acetate (30 mL) and washed with aqueous $NaHCO_3$ (3 × 30 mL) and brine (30 mL). The combined organic layer was dried over Na_2SO_4. After the removal of solvent, the residue was purified by flash chromatography on silica gel (ethyl acetate:hexane = 1:9) to give 5-methyl-*N*-(quinolin-8-yl)hex-5-enamide as colorless oil (137 mg, 64%).

^{1}H NMR (400 MHz, $CDCl_3$) δ 9.80 (s, 1H), 9.20 – 8.73 (m, 2H), 8.15 (dd, *J* = 8.3, 1.7 Hz, 1H), 7.58 – 7.51 (m, 1H), 7.49 (dd, *J* = 8.2, 1.7 Hz, 1H), 7.45 (dd, *J* = 8.3, 4.2 Hz, 1H), 4.80 – 4.61 (m, 2H), 2.68 – 2.47 (m, 2H), 2.17 (t, *J* = 7.5 Hz, 2H), 2.04 – 1.83 (m, 2H), 1.76 (t, *J* = 1.1 Hz, 3H). ^{13}C NMR (100 MHz, $CDCl_3$) δ 171.8, 148.2, 145.1, 138.5, 136.5, 134.7, 128.1, 127.6, 121.7, 121.5, 116.5, 110.9, 37.6, 37.3, 23.5, 22.4. HRMS (EI$^+$) m/z calcd. For $[C_{16}H_{18}N_2O]^+$: 254.1419, found : 254.1417.

Preparation of internal *δ,ε*-alkene substrate (1e)

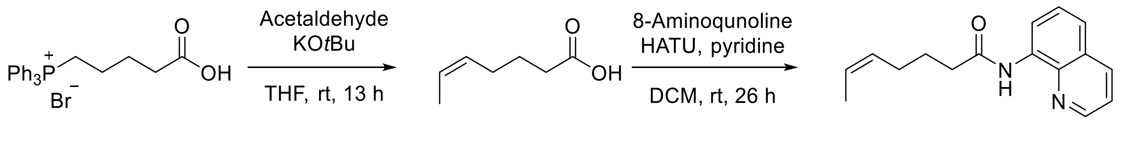

Wittig reaction[10]

To a suspension of (4-carboxybutyl)triphenylphosphonium bromide (4.65 g, 10.5 mmol) in dry THF (22 mL) was added KO*t*Bu (2.36 g, 21.0 mmol) portionwise at 0 °C under N_2 atmosphere and the mixture is stirred for 30 min at room temperature. A solution of the acetaldehyde (0.36 mL, 7.0 mmol) in dry THF (3.5 mL, 2 M) was added dropwise at 0 ºC. The reaction mixutre was allowed to warm to room temperature and stirred for 13 h. After the reaction mixture was acidified with 1 N HCl solution at 0 ºC and extracted with diethyl ether (3 × 60 mL). The combined organic layer was washed with water (2 × 60 mL) and brine (60 mL). The combined organic layer was dried over Na_2SO_4 and concentrated under reduced pressure to give hept-5-enoic acid. The crude mixture was used in the next step without further purification.

Amide bond formation[6]

To a solution of hept-5-enoic acid (7.0 mmol) in DCM (28 mL, 0.25 M) were added 8-aminoquinoline (1.01 g, 7.0 mmol), pyridine (1.13 mL, 14.0 mmol) and HATU (3.99 g, 10.5 mmol). The reaction mixture was stirred at room temperature for 26 h. The reaction mixture was monitored by TLC using ethyl acetate:hexane = 1:9 as the mobile phase. After disappearance of starting material, the reaction mixture was concentrated under reduced pressure. The residue was diluted with ethyl acetate (60 mL) and washed with aqueous $NaHCO_3$ (3 × 100 mL) and brine (100 mL). The combined organic layer was dried over Na_2SO_4. After the removal of solvent, the residue was purified by flash chromatography on silica gel (ethyl acetate:hexane = 1:9) to give N-(quinolin-8-yl)oct-5-enamide as colorless oil (E:Z ~ 1:10, 805 mg, 47% for 2 steps).

^{1}H NMR (400 MHz, $CDCl_3$) δ 9.80 (s, 1H), 9.37 – 8.54 (m, 2H), 8.15 (dd, *J* = 8.2, 1.7 Hz, 1H), 7.53 (t, *J* = 7.8 Hz, 1H), 7.49 (dd, *J* = 8.3, 1.6 Hz, 1H), 7.44 (dd, *J* = 8.3, 4.2 Hz, 1H), 5.60 – 5.39 (m, 2H), 2.65 – 2.46 (m, 2H), 2.20 (q, *J* = 7.2 Hz, 2H), 1.90 (p, *J* = 7.4 Hz, 2H), 1.66 – 1.50 (m, 3H). ^{13}C NMR (100 MHz, $CDCl_3$) δ 171.8, 148.2, 138.5, 136.5, 134.7, 129.6, 128.1, 127.6, 125.1, 121.7, 121.5, 116.5, 37.7, 26.4, 25.5, 13.0. HRMS (EI$^+$) m/z calcd. For $[C_{16}H_{18}N_2O]^+$: 254.1419, found : 254.1419.

Preparation of internal *δ,ε*-alkene substrate (1f)

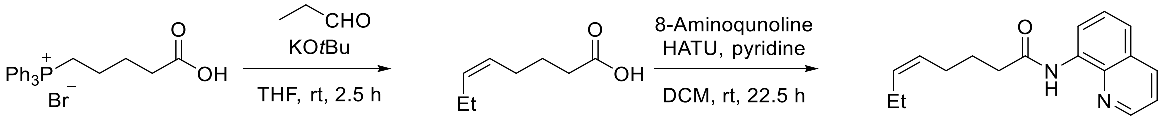

Wittig reaction[10]

To a suspension of (4-carboxybutyl)triphenylphosphonium bromide (3.32 g, 7.5 mmol) in dry THF (13.5 mL) was added KO*t*Bu (1.68 g, 15.0 mmol) portionwise at 0 °C under N_2 atmosphere and the mixture is stirred for 30 min at room temperature. A solution of the propionaldehyde (0.36 mL, 5.0 mmol) in dry THF (2.5 mL, 2 M) was added dropwise at 0 ºC. The reaction mixutre was allowed to warm to room temperature and stirred for 2.5 h. After the reaction mixture was acidified with 1 N HCl

solution at 0 °C and extracted with diethyl ether (3 × 60 mL). The combined organic layer was washed with water (2 × 60 mL) and brine (60 mL). The combined organic layer was dried over Na_2SO_4 and concentrated under reduced pressure to give oct-5-enoic acid. The crude mixture was used in the next step without further purification.

Amide bond formation[6]

To a solution of oct-5-enoic acid (5.0 mmol) in DCM (20 mL, 0.25 M) were added 8-aminoquinoline (721 mg, 5.0 mmol), pyridine (0.83 mL, 10.0 mmol) and HATU (2.85 g, 7.5 mmol). The reaction mixture was stirred at room temperature for 34 h. The reaction mixture was monitored by TLC using ethyl acetate:hexane = 1:9 as the mobile phase. After disappearance of starting material, the reaction mixture was concentrated under reduced pressure. The residue was diluted with ethyl acetate (60 mL) and washed with aqueous $NaHCO_3$ (3 × 100 mL) and brine (100 mL). The combined organic layer was dried over Na_2SO_4. After the removal of solvent, the residue was purified by flash chromatography on silica gel (ethyl acetate:hexane = 1:9) to give N-(quinolin-8-yl)hept-5-enamide as colorless oil (E:Z ~ 1:7, 805 mg, 36% for 2 steps).

^{1}H NMR (400 MHz, $CDCl_3$) δ 9.80 (s, 1H), 8.95 – 8.52 (m, 2H), 8.15 (dd, *J* = 8.2, 1.7 Hz, 1H), 7.53 (t, *J* = 7.8 Hz, 1H), 7.49 (dd, *J* = 8.3, 1.6 Hz, 1H), 7.44 (dd, *J* = 8.3, 4.2 Hz, 1H), 5.56 – 5.14 (m, 2H), 2.57 (t, *J* = 7.6 Hz, 2H), 2.19 (q, *J* = 7.2 Hz, 2H), 2.05 (p, *J* = 7.1 Hz, 2H), 1.89 (p, *J* = 7.4 Hz, 2H), 0.95 (t, *J* = 7.6 Hz, 3H). ^{13}C NMR (100 MHz, $CDCl_3$) δ 171.8, 148.2, 138.5, 136.5, 134.7, 132.9, 128.1, 128.1, 127.6, 121.7, 121.5, 116.5, 37.7, 26.7, 25.7, 20.7, 14.5. HRMS (EI^+) m/z calcd. For $[C_{17}H_{20}N_2O]^+$: 268.1576, found : 268.1573.

Preparation of internal *δ,ε*-alkene substrate (1f')

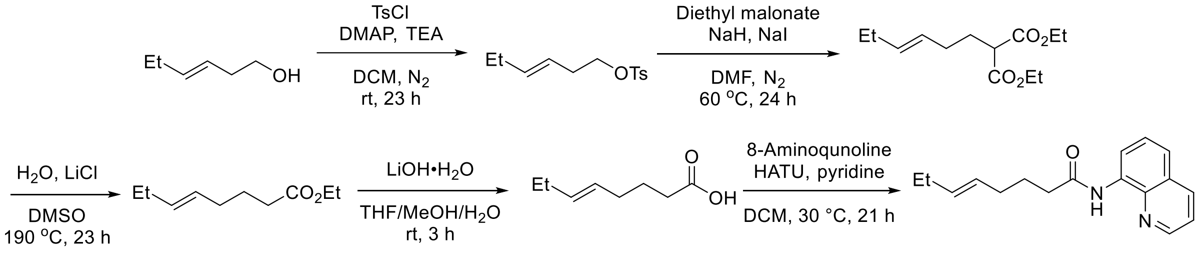

Tosylation

To tosyl chloride (2.1 g, 11.0 mmol) and DMAP (0.31 g, 2.5 mmol) in dry DCM (25.0 mL) was added triethylamine (2.8 mL, 20.0 mmol) under N_2 atmosphere and the reaction mixture was cooled to 0 °C. To the reaction mixture was added trans-3-hexanol (1.2 mL, 10.0 mmol) dropwisely and the mixture was stirred at room temperature overnight. The reaction mixture was monitored by TLC using EA:Hx = 1:3 as the mobile phase. After disappearance of starting material, the reaction mixture was washed with aqueous $NaHCO_3$ (1 × 30 mL) and water (30 mL). The combined aqueous layer was extracted with CH_2Cl_2 (3 × 50 mL). The combined organic layer was dried over Na_2SO_4. After the removal of

solvent, the residue was purified by flash chromatography on silica gel (ethyl acetate:hexane = 1:9) to give (E)-hex-3-en-1-yl 4-methylbenzenesulfonate as colorless oil (2.00 g, 78%)

Alkylation of malonic ester[7]

To diethyl malonate (1.3 mL, 8.6 mmol) in dry DMF (39.1 mL, 0.2 M) was added NaH (60% in mineral oil, 344 mg, 8.6 mmol) at 0 °C under N_2 atmosphere. The mixture was allowed to warm to room temperature and stirred for 30 min. The mixture was cooled to 0 °C and to the reaction mixture were added alkyl tosylate (2.0 g, 7.8 mmol) dropwisely and NaI (1.2 g, 7.8 mmol). The reaction mixture was allowed to warm to room temperature and stirred for 24 h. The reaction mixture was monitored by TLC using EA:Hx = 1:3 as the mobile phase. After disappearance of starting material, The reaction mixture was quenched with water (50 mL) at 0 °C and extracted with diethyl ether (3 × 50 mL). The combined organic layer was washed with saturated NH_4Cl solution (2 × 150 mL) and brine (150 mL), dried over Na_2SO_4. After the removal of solvent, the residue was purified by flash chromatography on silica gel (ethyl acetate:hexane = 1:20) to give diethyl (E)-2-(hex-3-en-1-yl)malonate as colorless oil (724 mg, 38%).

Krapcho decarboxylation[8]

To diethyl (E)-2-(hex-3-en-1-yl)malonate (724 mg, 3.0 mmol) in DMSO (12.0 mL, 0.25 M) was added LiCl (253 mg, 6.0 mmol) and H_2O (0.11 mL, 6.0 mmol). The mixture was heated to 190 °C for 23 h. The reaction mixture was monitored by TLC using EA:Hx = 1:3 as the mobile phase. After disappearance of starting material, the reaction mixture was quenched with saturated NH_4Cl solution (50 mL) at room termperature and extracted with diethyl ether (3 × 50 mL). The combined organic layer was washed with water (2 × 150 mL) and brine (150 mL), dried over Na_2SO_4. After the removal of solvent, the crude mixture was used in the next step without further purification.

Ester hydrolysis[9]

To a solution of ethyl (E)-oct-5-enoate (509 mg, 3.0 mmol) in THF:H_2O:MeOH = 1:1:1 (12.0 mL, 0.25 M) was added lithium hydroxide monohydrate (627 mg, 15.0 mmol). The reaction mixture was stirred at room temperature for 3 h. The reaction mixture was concentrated under reduced pressure and diluted with distilled water (20 mL). The aqueous layer was acidified with 1 N HCl at 0 °C and extracted with DCM (3 × 50 mL). The combined organic layer was dried over Na_2SO_4 and concentrated under reduced pressure. After the removal of solvent, the crude mixture was used in the next step without further purification.

Amide bond formation[3]

To a solution of (E)-oct-5-enoic acid (270 mg, 1.9 mmol) in DCM (4.8 mL, 0.33 M) were added 8-

aminoquinoline (228 mg, 1.6 mmol), pyridine (256 μL, 3.2 mmol) and HATU (722 mg, 1.9 mmol). The reaction mixture was stirred at 30 °C for 40 h. The reaction mixture was monitored by TLC using EA:Hx = 1:9 as the mobile phase. After disappearance of starting material, the reaction mixture was concentrated under reduced pressure. The residue was diluted with ethyl acetate (30 mL) and washed with aqueous $NaHCO_3$ (3 × 30 mL) and brine (30 mL). The combined organic layer was dried over Na_2SO_4. After the removal of solvent, the residue was purified by flash chromatography on silica gel (ethyl acetate:hexane = 1:7) to give (E)-*N*-(quinolin-8-yl)oct-5-enamide as colorless oil (327 mg, 41% for 3 steps).

^1H NMR (600 MHz, $CDCl_3$) δ 9.80 (s, 1H), 8.82 – 8.76 (m, 2H), 8.16 (dd, *J* = 8.2, 1.7 Hz, 1H), 7.54 (t, *J* = 7.9 Hz, 1H), 7.50 (dd, *J* = 8.2, 1.5 Hz, 1H), 7.46 (dd, *J* = 8.2, 4.2 Hz, 1H), 5.53 (dt, *J* = 15.3, 6.3 Hz, 1H), 5.43 (dt, *J* = 15.5, 6.7 Hz, 1H), 2.56 (t, *J* = 7.6 Hz, 2H), 2.14 (q, *J* = 7.1 Hz, 2H), 2.02 (p, *J* = 7.4 Hz, 2H), 1.90 (p, *J* = 7.4 Hz, 2H), 0.97 (t, *J* = 7.4 Hz, 3H). ^{13}C NMR (100 MHz, $CDCl_3$) δ 172.0, 148.2, 138.5, 136.5, 134.7, 133.5, 128.2, 128.1, 127.6, 121.7, 121.5, 116.5, 77.5, 77.2, 76.8, 37.6, 32.1, 25.7, 25.6, 14.0. HRMS (EI^+) m/z calcd. For $[C_{17}H20N_2O]^+$: 268.1576, found : 268.1577.

Preparation of internal *δ,ε*-alkene substrate (1g)

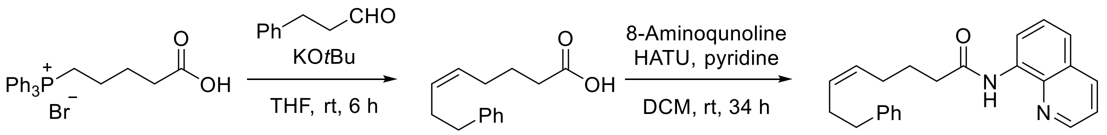

Wittig reaction[10]

To a suspension of (4-carboxybutyl)triphenylphosphonium bromide (3.32 g, 7.5 mmol) in dry THF (13.5 mL) was added KO*t*Bu (1.68 g, 15.0 mmol) portionwise at 0 °C under N_2 atmosphere and the mixture is stirred for 30 min at room temperature. A solution of the 3-phenylpropanal (0.66 mL, 5.0 mmol) in dry THF (2.5 mL, 2 M) was added dropwise at 0 °C. The reaction mixutre was allowed to warm to room temperature and stirred for 6h. After the reaction mixture was acidified with 1 N HCl solution at 0 °C and extracted with diethyl ether (3 × 60 mL). The combined organic layer was washed with water (2 × 60 mL) and brine (60 mL). The combined organic layer was dried over Na_2SO_4 and concentrated under reduced pressure to give 8-phenyloct-5-enoic acid. The crude mixture was used in the next step without further purification.

Amide bond formation[6]

To a solution of 8-phenyloct-5-enoic acid (5.0 mmol) in DCM (20 mL, 0.25 M) were added 8-aminoquinoline (721 mg, 5.0 mmol), pyridine (0.83 mL, 10.0 mmol) and HATU (2.85 g, 7.5 mmol). The reaction mixture was stirred at room temperature for 34 h. The reaction mixture was monitored by TLC using ethyl acetate:hexane = 1:9 as the mobile phase. After disappearance of starting material, the reaction mixture was concentrated under reduced pressure. The residue was diluted with ethyl acetate

(60 mL) and washed with aqueous $NaHCO_3$ (3 × 100 mL) and brine (100 mL). The combined organic layer was dried over Na_2SO_4. After the removal of solvent, the residue was purified by flash chromatography on silica gel (ethyl acetate:hexane = 1:9) to give 8-phenyl-*N*-(quinolin-8-yl)oct-5-enamide as a white solid (E:Z ~ 1:7, 805 mg, 47% for 2 steps).

^{1}H NMR (400 MHz, $CDCl_3$) δ 9.77 (s, 1H), 8.87 – 8.64 (m, 2H), 8.16 (dd, *J* = 8.3, 1.8 Hz, 1H), 7.57 – 7.52 (m, 1H), 7.50 (dd, *J* = 8.3, 1.6 Hz, 1H), 7.45 (dd, *J* = 8.2, 4.2 Hz, 1H), 5.55 – 5.37 (m, 2H), 2.66 (t, *J* = 7.6 Hz, 2H), 2.54 – 2.44 (m, 2H), 2.42 – 2.29 (m, 2H), 2.13 (q, *J* = 7.1 Hz, 2H), 1.82 (p, *J* = 7.4 Hz, 2H). ^{13}C NMR (100 MHz, $CDCl_3$) δ 171.8, 148.2 142.1, 138.5, 136.5, 134.7, 130.0, 129.6, 128.6, 128.4, 128.1, 127.6, 125.9, 121.7, 121.5, 116.6, 37.5, 36.0, 29.4, 26.8, 25.5. HRMS (EI$^+$) m/z calcd. For $[C_{23}H_{24}N_2O]^+$: 344.1889, found : 344.1887.

Preaparation of internal *δ,ε*-alkene substrate (1h)

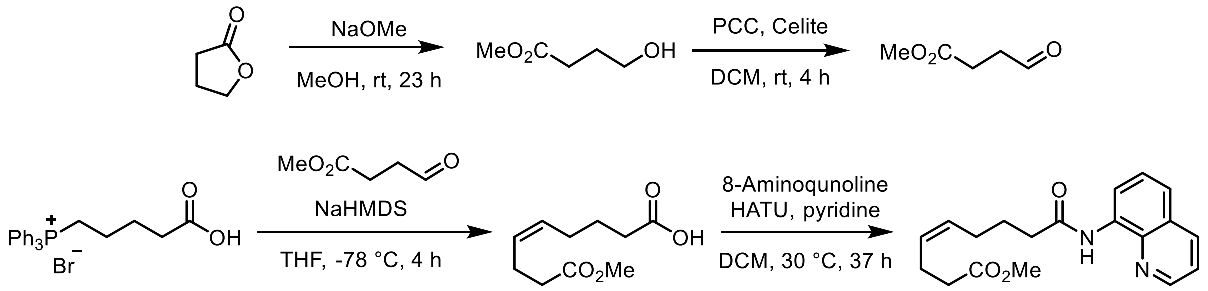

Lactone cleavage [11]

To the 0.5 M sodium methoxide solution in MeOH (2.0 ml, 1.0 mmol) diluted with MeOH (8.0 mL, 1.0 M) was added γ-Butyrolactone (0.77 mL, 10.0 mmol) at room temperature under argon atmosphere. The mixture was refluxed for 23 h. The reaction mixture was monitored by TLC using DCM/Et_2O/Hx = 1:1:2 as mobile phase. After disappearance of starting material, the reaction mixture was diluted with diethyl ether (30 mL), filtered off through a pad of celite and silica. After washing with diethyl ether, the filtrate was concentrated under reduced pressure. The residue was purified by flash chromatography on silica gel (DCM/Et_2O/Hx = 1:1:2 to 3:3:4) to give 4-hydroxybutanoate as pale yellowish oil (606 mg, 51%).

Alcohol oxidation[12]

To a solution of 4-hydroxybutanoate (606 mg, 5.13 mmol) in dry DCM (8.6 mL, 0.6 M) were added Pyridinium chlorochromate (1.33 g, 6.16 mmol) and celite (0.31 g, 5.13 mmol) under argon atmosphere. The reaction mixture was stirred at room temperature for 4 h. The reaction mixture was monitored by TLC using EA/Hx = 1:3 as the mobile phase. After disappearance of starting material, the reaction mixture was diluted with ether (50 mL) and filtered through silica gel and celite with ether (50 mL). The reaction mixture was concentrated under reduced pressure to give methyl 4-oxobutanoate (500 mg, 84%). The residue was used in the next step without further purification.

Wittig reaction[10]

To a suspension of (4-carboxybutyl)triphenylphosphonium bromide (665 g, 1.5 mmol) in dry THF (2.3 mL) was added 1.0 M NaHMDS solution in THF (2.5 mL, 2.5 mmol) portionwise at -78 °C under argon atmosphere and the mixture is stirred for 30 min at room temperature. A solution of the methyl 4-oxobutanoate (0.12 mg, 1.0 mmol) in dry THF (1.5 mL, 0.16 M) was added dropwise at -78 °C. Then, the reaction mixutre was stirred at -78 °C for 4 h. The reaction mixture was monitored by TLC using MeOH:DCM = 1:40 as the mobile phase. After disappearance of starting material, the reaction mixture was allowed to warm to room temperature gradyally and quenched by 1 N HCl solution at 0 °C and extracted with diethyl ether (3 × 20 mL). The combined organic layer was washed with water (2 × 20 mL) and brine (20 mL). The combined organic layer was dried over Na_2SO_4 and concentrated under reduced pressure to give 9-methoxy-9-oxonon-5-enoic acid. The crude mixture was used in the next step without further purification.

Amide bond formation[6]

To a solution of 9-methoxy-9-oxonon-5-enoic acid (356 mg, 1.0 mmol) in DCM (2.5 mL, 0.33 M) were added 8-aminoquinoline (120 mg, 0.83 mmol) pyridine (135 µL, 1.67 mmol) and HATU (380 mg, 1.0 mmol). The reaction mixture was stirred at 30 °C for 37 h. The reaction mixture was monitored by TLC using EA:Hx = 1:5 as the mobile phase. After disappearance of starting material, the reaction mixture was concentrated under reduced pressure. The residue was diluted with ethyl acetate (30 mL) and washed with aqueous $NaHCO_3$ (3 × 30 mL) and brine (30 mL). The combined organic layer was dried over Na_2SO_4. After the removal of solvent, the residue was purified by flash chromatography on silica gel (ethyl acetate:hexane = 1:5) to give 5-methyl-*N*-(quinolin-8-yl)hex-5-enamide as colorless oil (E:Z ~ 1:10, 84.8 mg, 31% for 2 steps).

^{1}H NMR (600 MHz, $CDCl_3$) δ 9.80 (s, 1H), 8.85 – 8.73 (m, 2H), 8.16 (dd, *J* = 8.2, 1.7 Hz, 1H), 7.56 – 7.48 (m, 2H), 7.46 (dd, *J* = 8.3, 4.2 Hz, 1H), 5.51 – 5.44 (m, 1H), 5.41 (dt, *J* = 11.3, 6.5 Hz, 1H), 3.64 (s, 3H), 2.57 (t, *J* = 7.6 Hz, 2H), 2.39 – 2.32 (m, 4H), 2.22 (q, *J* = 7.3 Hz, 2H), 1.94 – 1.84 (m, 2H). ^{13}C NMR (100 MHz, $CDCl_3$) δ 171.3, 148.1, 138.4, 136.4, 134.6, 128.5, 128.0, 127.5, 125.7, 121.6, 121.4, 116.5, 38.0, 23.1, 12.9. HRMS (EI$^+$) m/z calcd. For $[C_{19}H_{22}N_2O_3]^+$: 326.1630, found : 326.1632.

Preaparation of cylic *δ,ε*-alkene substrate (1i)

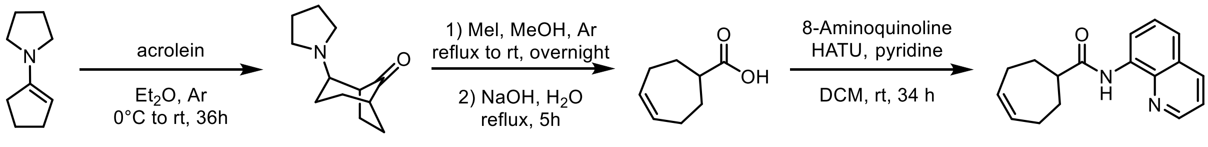

Michael addition and intramolecular Mannich reaction[13]

To a solution of 1-pyrrolidino-1-cyclopentene (5.8 mL, 40.0 mmol) in dry diethyl ether (13 mL) was

added acrolein (2.9 mL, 44.0 mmol) dropwisely for 6 min at 0 ºC under Ar and stirred until the reaction mixture was changed into colorless solution. Then, it was stirred at room temperature for 36h. The reaction mixture was monitored by TLC using EA:Hx = 1:3 (1% TEA) as the mobile phase. After disappearance of starting material, the pH value of the reaction mixture was adjusted to 4.0 with HCl (6 N) at 0 ºC and washed with ethyl acetate (3 × 50 mL). Then the pH value of the aqueous layer was adjusted to 10.0 with KOH (1 N) and extracted with ethyl acetate (3 × 50 mL). The combined organic layer was dried over Na_2SO_4 and concentrated under reduced pressure to give 2-(pyrrolidin-1-yl)bicyclo[3.2.1]octan-8-one. The crude mixture was used in the next step without further purification.

Nucleophillic addition and Grob fragmentation[13]

To a solution of 2-(pyrrolidin-1-yl)bicyclo[3.2.1]octan-8-one (2.59 g, 13.4 mmol) in dry MeOH (13 mL) was added iodomethane (1.00 mL, 16.1 mmol) dropwisely under Ar. Then the reaction mixture was refluxed at 80 ºC for 45 min and stirred at room temperature overnight. The reaction mixture was monitored by TLC using DCM:MeOH = 20:1 as the mobile phase. After disappearance of starting material, the rection mixture was concentrated under reduced pressure and was added 20% NaOH solution (8.05 mL, 40.2 mmol). The reaction mixture was heated at reflux for 5 h and cooled to room temperature. The reaction mixture was diluted with water (20 mL) and washed with diethyl ether (2 × 30 mL). The combined aqueous layer was cooled to 0 ºC. Then the pH value of the aqueous layer was adjusted to 1.0 with HCl (6 N) and the aqueous layer was extracted with diethyl ether (2 × 30 mL). The combined organic layer was dried over Na_2SO_4 and concentrated under reduced pressure to give cyclohept-4-ene-1-carboxylic acid. The crude mixture was used in the next step without further purification.

Amide bond formation[6]

To a solution of cyclohept-4-ene-1-carboxylic acid (891 mg, 6.36 mmol) in DCM (15.9 mL, 0.33 M) were added 8-aminoquinoline (764 mg, 5.30 mmol), pyridine (0.51 mL, 6.36 mmol) and HATU (2.42 g, 6.36 mmol). The reaction mixture was stirred at room temperature for 34 h. The reaction mixture was monitored by TLC using EA:Hx = 1:6 as the mobile phase. After disappearance of starting material, the reaction mixture was concentrated under reduced pressure. The residue was diluted with ethyl acetate (20 mL) and washed with aqueous $NaHCO_3$ (3 × 50 mL) and brine (50 mL). The combined organic layer was dried over Na_2SO_4. After the removal of solvent, the residue was purified by flash chromatography on silica gel (EA:Hx = 1:6) to give N-(quinolin-8-yl)cyclohept-4-ene-1-carboxamide as white solid (853 mg, 7% for 3 steps).

^{1}H NMR (400 MHz, $CDCl_3$) δ 10.02 – 9.46 (m, 1H), 8.96 – 8.69 (m, 2H), 8.19 (dd, *J* = 8.3, 1.7 Hz, 1H), 7.59 – 7.50 (m, 2H), 7.48 (dd, *J* = 8.3, 4.2 Hz, 1H), 2.75 (tt, *J* = 10.3, 3.7 Hz, 1H), 2.49 – 2.36 (m, 2H), 2.30 – 2.19 (m, 2H), 2.20 – 2.10 (m, 2H), 1.91 – 1.77 (m, 2H). ^{13}C NMR (100 MHz, $CDCl_3$) δ 175.1,

148.1, 138.5, 136.4, 134.7, 131.9, 128.0, 127.5, 121.6, 121.3, 116.4, 51.4, 30.2, 27.1. HRMS (EI^+) m/z calcd. For $[C_{17}H_{18}N_2O]^+$: 266.1419, found : 266.1422.

Preparation of internal *ε,ζ*-alkene substrate (1k)

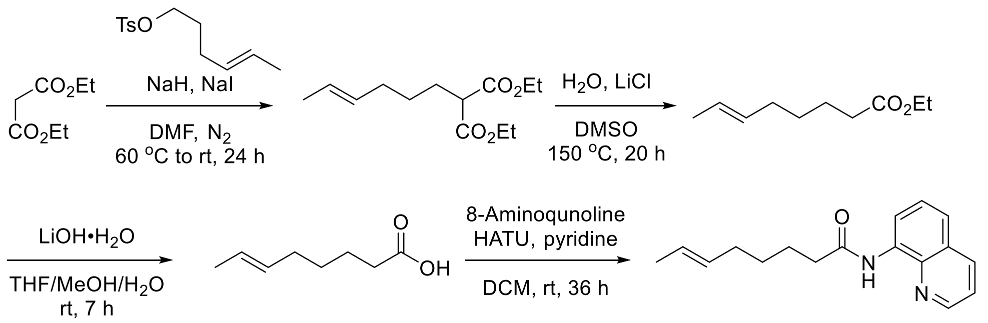

Alkylation of malonic ester[7]

To diethyl malonate (1.36 mL, 9.0 mmol) in dry DMF (32.0 mL, 0.25 M) was added NaH (60% in mineral oil, 0.359 g, 8.98 mmol) at 0 °C under N_2 atmosphere. The mixture was allowed to warm to room temperature and stirred for 30 min. The mixture was cooled to 0 °C and alkyl tosylate (2.08 g, 8.2 mmol) was added dropwise. The reaction mixture was allowed to warm to room temperature and stirred for 23 h. The reaction mixture was quenched with saturated NH_4Cl solution (60 mL) at 0 °C and extracted with diethyl ether (3 × 50 mL). The combined organic layer was washed with saturated NH_4Cl solution (2 × 50 mL) and brine (50 mL), dried over $MgSO_4$. After the removal of solvent to give diethyl (E)-2-(hex-4-en-1-yl)malonate as colorless oil (2.14 g, quant). The residue was used in the next step without further purification.

Krapcho decarboxylation[8]

To diethyl (E)-2-(hex-4-en-1-yl)malonate (2.14 g, 8.8 mmol) in DMSO (35.0 mL, 0.25 M) was added LiCl (0.746 g, 17.6 mmol) and H_2O (0.317 mL, 17.6 mmol). The mixture was heated to 150 °C for 20 h. After the reaction mixture was quenched with saturated NH_4Cl solution (50 mL) at room termperature and extracted with diethyl ether (3 × 50 mL). The combined organic layer was washed with water (2 × 50 mL) and brine (50 mL), dried over $MgSO_4$. After the removal of solvent to give ethyl (E)-oct-6-enoate as colorless oil (1.19 g, 79%). The residue was used in the next step without further purification.

Ester hydrolysis[9]

To a solution of ethyl (E)-oct-6-enoate (1.19 g, 7.0 mmol) in THF:H_2O:MeOH = 1:1:1 (28.0 mL, 0.25 M) was added lithium hydroxide monohydrate (1.47 g, 35.0 mmol). The reaction mixture was stirred at room temperature for 7 h. The reaction mixture was concentrated under reduced pressure and diluted with distilled water (50 mL). The aqueous layer was acidified with 1 N HCl at 0 °C and extracted with DCM (3 × 50 mL). The combined organic layer was dried over Na_2SO_4 and concentrated under

reduced pressure to give (E)-oct-6-enoic acid as colorless oil (468 mg, 47%) . The residue was used in the next step without further purification.

Amide bond formation[6]

To a solution of (E)-oct-6-enoic acid (468 mg, 3.3 mmol) in DCM (13 mL, 0.2 M) were added 8-aminoquinoline (476 mg, 3.3 mmol) pyridine (546 µL, 6.6 mmol) and HATU (1.88 g, 5.0 mmol). The reaction mixture was stirred at room temperature for 36 h. The reaction mixture was monitored by TLC using EA:Hx = 1:9 as the mobile phase. After disappearance of starting material, the reaction mixture was concentrated under reduced pressure. The residue was diluted with ethyl acetate (50 mL) and washed with aqueous $NaHCO_3$ (3 × 50 mL) and brine (50 mL). The combined organic layer was dried over Na_2SO_4. After the removal of solvent, the residue was purified by flash chromatography on silica gel (ethyl acetate:hexane = 1:9) to give (E)-*N*-(quinolin-8-yl)oct-6-enamide as colorless oil (564 mg, 64%).

^{1}H NMR (400 MHz, $CDCl_3$) δ 9.80 (s, 1H), 8.92 – 8.64 (m, 2H), 8.15 (dd, *J* = 8.3, 1.7 Hz, 1H), 7.56 – 7.50 (m, 1H), 7.48 (dd, *J* = 8.3, 1.6 Hz, 1H), 7.44 (dd, *J* = 8.3, 4.2 Hz, 1H), 5.61 – 5.26 (m, 2H), 2.62 – 2.48 (m, 2H), 2.10 – 2.01 (m, 2H), 1.88 – 1.76 (m, 2H), 1.67 – 1.60 (m, 3H), 1.56 – 1.40 (m, 2H). ^{13}C NMR (100 MHz, $CDCl_3$) δ 171.9, 148.2, 138.5, 136.5, 134.7, 131.1, 128.1, 127.6, 125.3, 121.7, 121.4, 116.5, 38.3, 32.4, 29.3, 25.3, 18.0. HRMS (EI$^+$) m/z calcd. For $[C_{17}H_{20}N_2O]^+$: 268.1576, found : 268.1573.

Preparation of substituted *ε,ζ*-alkene substrate (1l)

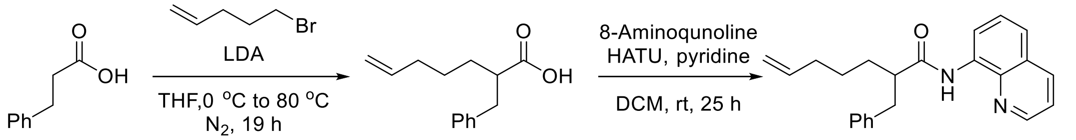

α-Benzylation of acid[14]

To a solution LDA (12 mL, 12.0 mmol, 1 M in THF) in THF (18 mL) were added 3-phenylpropanoic acid at 0 °C under N_2. The reaction mixture was slowly wamed to 80 °C and stirred for 2 h. 5-bromopent-1-ene (711 µL, 6.0 mmol)was then added dropwise at 0 °C. The reaction mixture was wamed to 80 °C again and stirred for 17 h. Then the reaction mixture was quenched with water (30 mL) and ethyl acetate (30 mL), and washed with water (2 × 30 mL). Then the pH value of the combined water layer was adjusted to 4.0 with HCl (1 M) and extracted with ethyl acetate (3 × 50 mL). The combined organic layer was dried over $MgSO_4$ and concentrated under reduced pressure to give 2-benzylhept-6-enoic acid. The crude mixture was used in the next step without further purification.

Amide bond formation[6]

To a solution of 2-benzylhept-6-enoic acid (6.0 mmol) in DCM (20 mL, 0.25 M) were added 8-

aminoquinoline (865 mg, 6.0 mmol), pyridine (0.97 mL, 12.0 mmol) and HATU (3.42 g, 9.0 mmol). The reaction mixture was stirred at room temperature for 25 h. The reaction mixture was monitored by TLC using ethyl acetate:hexane = 1:9 as the mobile phase. After disappearance of starting material, the reaction mixture was concentrated under reduced pressure. The residue was diluted with ethyl acetate (60 mL) and washed with aqueous $NaHCO_3$ (3 × 100 mL) and brine (100 mL). The combined organic layer was dried over Na_2SO_4. After the removal of solvent, the residue was purified by flash chromatography on silica gel (ethyl acetate:hexane = 1:9) to give 2-benzyl-*N*-(quinolin-8-yl)hept-6-enamide as colorless oil (1.12 g, 54% for 2 steps).

^{1}H NMR (400 MHz, $CDCl_3$) δ 9.69 (s, 1H), 8.79 (dd, *J* = 7.4, 1.6 Hz, 1H), 8.75 (dd, *J* = 4.2, 1.7 Hz, 1H), 8.13 (dd, *J* = 8.3, 1.7 Hz, 1H), 7.52 (dd, *J* = 8.3, 7.4 Hz, 1H), 7.48 (dd, *J* = 8.3, 1.6 Hz, 1H), 7.42 (dd, *J* = 8.3, 4.2 Hz, 1H), 7.29 – 7.23 (m, 3H), 7.23 – 7.19 (m, 1H), 7.15 – 7.10 (m, 1H), 5.76 (ddt, *J* = 16.9, 10.2, 6.7 Hz, 1H), 5.03 – 4.94 (m, 1H), 4.94 – 4.85 (m, 1H), 3.16 (dd, *J* = 13.6, 8.2 Hz, 1H), 2.87 (dd, *J* = 13.6, 6.5 Hz, 1H), 2.76 (dddd, *J* = 9.3, 8.2, 6.4, 4.6 Hz, 1H), 2.14 – 2.05 (m, 2H), 1.94 – 1.80 (m, 1H), 1.71 – 1.42 (m, 3H). ^{13}C NMR (100 MHz, $CDCl_3$) δ 173.9, 148.2, 139.8, 138.5, 138.5, 136.4, 134.5, 129.1, 128.5, 128.0, 127.5, 126.4, 121.6, 121.5, 116.6, 114.9, 51.3, 39.4, 33.9, 32.4, 26.9. HRMS (EI$^+$) m/z calcd. For $[C_{23}H_{24}N_2O]^+$: 344.1889, found : 344.1890.

Preaparation of substituted *ζ,η*-alkene substrate (1m)[6]

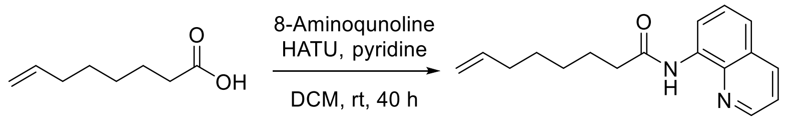

To a solution of oct-7-enoic acid (5.0 mmol) in DCM (20 mL, 0.25 M) were added 8-aminoquinoline (721 mg, 5.0 mmol), pyridine (0.83 mL, 10.0 mmol) and HATU (2.85 g, 7.5 mmol). The reaction mixture was stirred at room temperature for 40 h. The reaction mixture was monitored by TLC using ethyl acetate:hexane = 1:9 as the mobile phase. After disappearance of starting material, the reaction mixture was concentrated under reduced pressure. The residue was diluted with ethyl acetate (60 mL) and washed with aqueous $NaHCO_3$ (3 × 100 mL) and brine (100 mL). The combined organic layer was dried over Na_2SO_4. After the removal of solvent, the residue was purified by flash chromatography on silica gel (ethyl acetate:hexane = 1:9) to give N-(quinolin-8-yl)oct-7-enamide (1.20 g, 90%).

^{1}H NMR (400 MHz, $CDCl_3$) δ 9.80 (s, 1H), 8.95 – 8.49 (m, 2H), 8.15 (dd, *J* = 8.3, 1.7 Hz, 1H), 7.53 (t, *J* = 7.8 Hz, 1H), 7.48 (dd, *J* = 8.3, 1.6 Hz, 1H), 7.44 (dd, *J* = 8.2, 4.2 Hz, 1H), 5.81 (ddt, *J* = 16.9, 10.2, 6.6 Hz, 1H), 5.08 – 4.97 (m, 1H), 4.97 – 4.87 (m, 1H), 2.68 – 2.13 (m, 2H), 2.30 – 1.99 (m, 2H), 1.92 – 1.76 (m, 2H), 1.58 – 1.31 (m, 4H). ^{13}C NMR (100 MHz, $CDCl_3$) δ 171.9, 148.2, 139.0, 138.5, 136.5, 134.7, 128.1, 127.6, 121.7, 121.4, 116.5, 114.5, 38.3, 33.7, 28.9, 28.8, 25.6. HRMS (EI$^+$) m/z calcd. For $[C_{17}H_{20}N_2O]^+$: 268.1576, found : 268.1579.

Preparation of substituted PA *ε,ζ*-alkene substrate (5b)

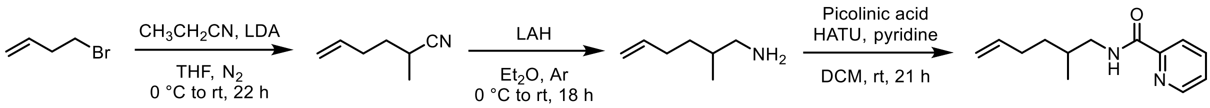

Alkylation of nitrile[15]

To a solution of 1.0 M Lithium diisopropylamide in THF (6.0 mL, 6.0 mmol) diluted with dry THF (4.0 mL, 0.5 M) was added propiononitrile (0.36 mL, 5.0 mmol) dropwisely at 0 °C under N_2 atmosphere. Then, the reaction mixutre was stirred at 0 °C for 30 min. Then, 4-bromobut-1-ene (0.51 mL, 5.0 mmol) was added and the reaction mixture was stirred at 0 °C for 15 min. Then the reaction mixture was allowed to warm to room temperature and stirred overnight. The reaction mixture was monitored by TLC using EA:Hx = 1:3 as the mobile phase. After disappearance of starting material, The reaction mixture was quenched with water (30 mL). Then, the reaction mixture was extracted with diethyl ether (3 × 30 mL) and the combined organic layer was dried over Na_2SO_4. After the removal of solvent, the crude mixture was used in the next step without further purification.

Nitrile Reduction[16]

To a solution of Lithium aluminum hydride (285 mg, 7.5 mmol) in dry diethyl ether (10.0 mL) was added 2-methylhex-5-enenitrile (546 mg, 5.0 mmol), which is diluted with dry diethyl ether (5.0 mL), dropwisely at 0 °C. The reaction mixture was stirred at 0 °C for 15 min, slowly warmed to room temperature and stirred overnight. The reaction mixture was monitored by TLC using EA:Hx = 1:3 as the mobile phase. After disappearance of starting material, the reaction mixture was cooled down to 0 °C, quenched with saturated Rochelle salt solution (20 mL) dropwisely and stirred at room temperature until the color of the mixture changed into white color. Then, the mixture was dried over Na_2SO_4 and filtered with diethyl ether (50 mL). After the removal of solvent, the crude mixture was used in the next step without further purification.

Amide bond formation[6]

To a solution of 2-methylhex-5-en-1-amine (566 mg, 5.0 mmol) in DCM (15.0 mL, 0.33 M) were added picolinic acid (739 mg, 6.0 mmol), pyridine (809 μL, 10.0 mmol) and HATU (2.28 g, 6.0 mmol). The reaction mixture was stirred at room temperature for 21 h. The reaction mixture was monitored by TLC using EA:Hx = 1:4 as the mobile phase. After disappearance of starting material, the reaction mixture was concentrated under reduced pressure. The residue was diluted with ethyl acetate (30 mL) and washed with aqueous $NaHCO_3$ (3 × 30 mL) and brine (30 mL). The combined organic layer was dried over Na_2SO_4. After the removal of solvent, the residue was purified by flash chromatography on silica gel (ethyl acetate:hexane = 1:4) to give N-(2-methylhex-5-en-1-yl)picolinamide as yellowish oil (131 mg, 12% for 3 steps).

^{1}H NMR (400 MHz, $CDCl_3$) δ 8.55 (ddd, J = 4.8, 1.8, 0.9 Hz, 1H), 8.21 (dt, J = 7.8, 1.1 Hz, 1H), 8.13

(s, 1H), 7.85 (td, *J* = 7.7, 1.7 Hz, 1H), 7.42 (ddd, *J* = 7.6, 4.8, 1.3 Hz, 1H), 5.81 (ddt, *J* = 16.9, 10.2, 6.6 Hz, 1H), 5.03 (dq, *J* = 17.1, 1.7 Hz, 1H), 4.95 (ddt, *J* = 10.2, 2.3, 1.3 Hz, 1H), 3.43 (dt, *J* = 13.3, 6.1 Hz, 1H), 3.31 (ddd, *J* = 13.4, 7.2, 6.4 Hz, 1H), 2.24 – 2.01 (m, 2H), 1.81 (dddd, *J* = 12.6, 8.3, 7.0, 5.7 Hz, 1H), 1.55 (dddd, *J* = 13.4, 9.5, 6.4, 5.3 Hz, 1H), 1.30 (dddd, *J* = 13.8, 9.3, 8.3, 5.8 Hz, 1H), 0.99 (d, *J* = 6.7 Hz, 3H). ^{13}C NMR (100 MHz, $CDCl_3$) δ 164.4, 150.2, 148.1, 138.8, 137.5, 126.2, 122.4, 114.7, 45.4, 33.7, 33.2, 31.3, 17.7. HRMS (EI^+) m/z calcd. For $[C_{13}H_{18}N_2O]^+$: 218.1419, found : 218.1417.

Preparation of PA *ζ,η*-alkene substrate (5c)

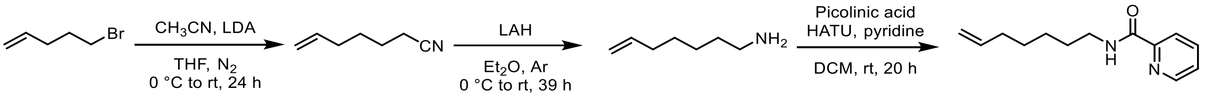

Alkylation of nitrile[15]

To a solution of 1.0 M Lithium diisopropylamide in THF (7.2 mL, 7.2 mmol) diluted with dry THF (4.8 mL, 0.5 M) was added acetonitrile (0.31 mL, 6.0 mmol) dropwisely at 0 ºC under N_2 atmosphere. Then, the reaction mixutre was stirred at 0 ºC for 30 min. Then, 5-bromopent-1-ene (0.71 mL, 6.0 mmol) was added and the reaction mixture was stirred at 0 ºC for 15 min. Then the reaction mixture was allowed to warm to room temperature and stirred overnight. The reaction mixture was monitored by TLC using EA:Hx = 1:3 as the mobile phase. After disappearance of starting material, The reaction mixture was quenched with water (30 mL). Then, the reaction mixture was extracted with diethyl ether (3 × 30 mL) and the combined organic layer was dried over Na_2SO_4. After the removal of solvent, the crude mixture was used in the next step without further purification.

Nitrile Reduction[16]

To a solution of Lithium aluminum hydride (324 mg, 9.0 mmol) in dry diethyl ether (15.0 mL) was added hept-6-enenitrile (655 mg, 6.0 mmol), which is diluted with dry diethyl ether (3.0 mL), dropwisely at 0 ºC. The reaction mixture was stirred at 0 ºC for 15 min, slowly warmed to room temperature and stirred overnight. The reaction mixture was monitored by TLC using EA:Hx = 1:3 as the mobile phase. After disappearance of starting material, the reaction mixture was cooled down to 0 ºC, quenched with saturated Rochelle salt solution (20 mL) dropwisely and stirred at room temperature until the color of the mixture changed into white color. Then, the mixture was dried over Na_2SO_4 and filtered with diethyl ether (50 mL). After the removal of solvent, the crude mixture was used in the next step without further purification.

Amide bond formation[6]

To a solution of hept-6-en-1-amine (679 mg, 6.0 mmol) in DCM (18.0 mL, 0.33 M) were added picolinic acid (886 mg, 7.2 mmol), pyridine (971 μL, 12.0 mmol) and HATU (2.7 g, 7.2 mmol). The reaction

mixture was stirred at room temperature for 20 h. The reaction mixture was monitored by TLC using EA:Hx = 1:4 as the mobile phase. After disappearance of starting material, the reaction mixture was concentrated under reduced pressure. The residue was diluted with ethyl acetate (30 mL) and washed with aqueous $NaHCO_3$ (3 × 30 mL) and brine (30 mL). The combined organic layer was dried over Na_2SO_4. After the removal of solvent, the residue was purified by flash chromatography on silica gel (ethyl acetate:hexane = 1:4) to give N-(hept-6-en-1-yl)picolinamide as yellowish oil (210 mg, 16% for 3 steps).

^{1}H NMR (600 MHz, $CDCl_3$) δ 8.54 (d, *J* = 4.8 Hz, 1H), 8.20 (d, *J* = 7.8 Hz, 1H), 8.05 (s, 1H), 7.84 (td, *J* = 7.7, 1.7 Hz, 1H), 7.42 (dd, *J* = 7.6, 4.8 Hz, 1H), 5.80 (ddt, *J* = 16.9, 10.1, 6.7 Hz, 1H), 5.00 (dd, *J* = 17.1, 2.1 Hz, 1H), 4.94 (d, *J* = 10.2 Hz, 1H), 3.47 (q, *J* = 6.8 Hz, 2H), 2.07 (q, *J* = 6.9 Hz, 2H), 1.69 – 1.60 (m, 2H), 1.44 (tt, *J* = 10.4, 4.7 Hz, 4H). ^{13}C NMR (100 MHz, $CDCl_3$) δ 164.3, 150.2, 148.1, 139.0, 137.6, 126.2, 122.4, 114.6, 39.6, 33.8, 29.7, 28.7, 26.6. HRMS (EI^+) m/z calcd. For $[C_{13}H_{18}N_2O]^+$: 218.1419, found : 218.1420.

Preparation of Amine benzoate substrates

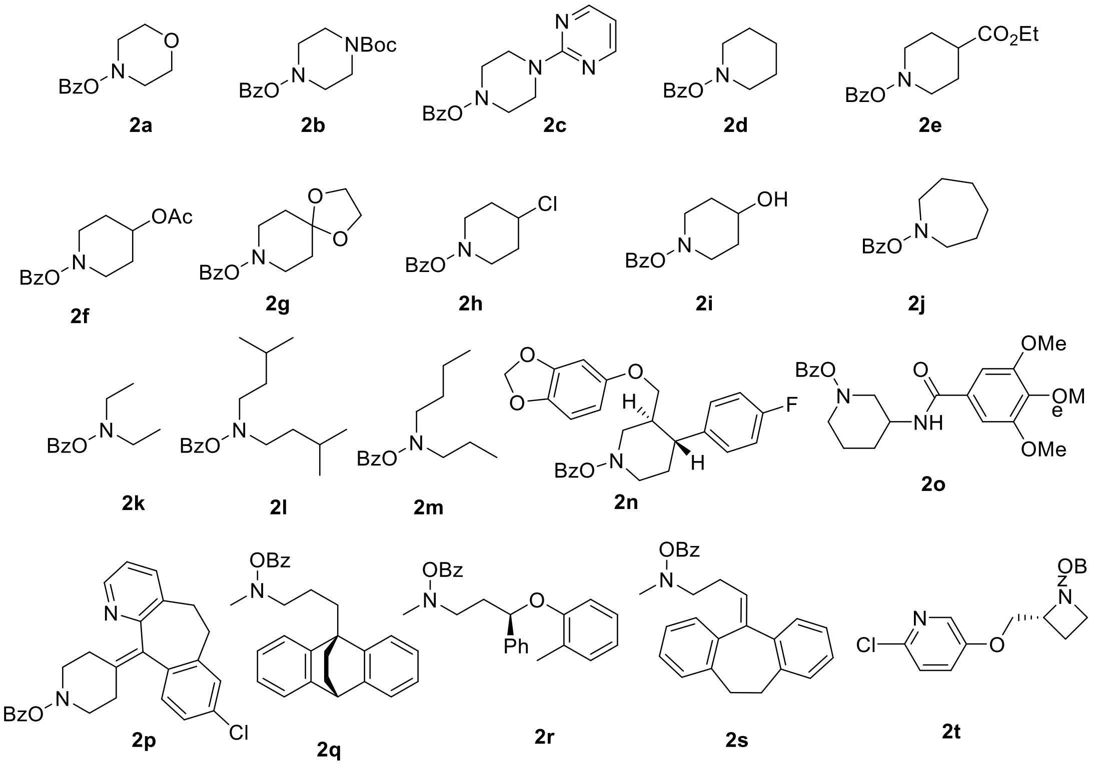

Preparation of amine benzoate substrates except **2o**, **2q**, **2r** and **2t** was based on literature methods.[17–27] The procedure to synthesize of **2o**, **2q**, **2r** and **2t** is shown below based on the modified literature methods.[28,29]

General procedure for the preparation of amine electrophiles (GP1)[28]

$$H{-}NR_2 \xrightarrow[\text{DMF, rt, 12 h}]{(PhCO_2)_2,\ K_2HPO_4} BzO{-}NR_2$$

To a solution of benzoyl peroxide (1.0 equiv) in DMF (0.4 M) were added potassium phosphate dibasic (1.5 equiv or 3.0 equiv if amine hydrochloride (or TFA) salt was used) and amine (or amine hydrochloride salt) (1.5 equiv) at 0 °C. After 30 min, the mixture was allowed to warm to room temperature and stirred for 12 h. The reaction mixture was monitored by TLC. After disappearance of starting material, the reaction mixture was quenched with distilled water and extracted with EA (2 times). The combined organic layer was washed with aqueous $NaHCO_3$ (2 times) and brine (1 time), dried over Na_2SO_4. After removal of solvent, the residue was purified by flash chromatography on silica gel to give a corresponding desired product compound.

General procedure for the preparation of amine electrophiles (GP2)[29]

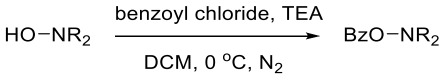

To a solution of hydroxyl amine (1.0 equiv) in CH_2Cl_2 (0.5 M) was added TEA (1.2 equiv) at room temperature under argon atmosphere. After cooled to 0 °C, benzoyl chloride (1.0 equiv) was added to reaction mixture and reaction mixture was stirred at 0 °C for 1 h. The reaction mixture was monitored by TLC. After disappearance of starting material, the reaction mixture was quenched with aqueous $NaHCO_3$ and extracted with CH_2Cl_2 (3 times). The combined organic layer was dried over Na_2SO_4. After removal of solvent, the residue was purified by flash chromatography on silica gel to give a corresponding desired product compound.

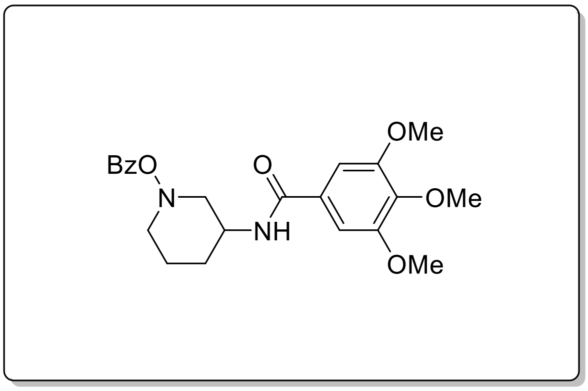

3-(3,4,5-Trimethoxybenzamido)piperidin-1-yl benzoate (2o).

Prepared according to GP2. Hydroxylamine was prepared according to the reference.[21] Purified with flash column chromatography (MeOH:CH_2Cl_2 = 1:49 to MeOH:CH_2Cl_2 = 1:29). From hydroxylamine (476 g, 1.54 mmol), compound **2o** (606 mg, 95%) was obtained. White solid.

^{1}H NMR (400 MHz, $CDCl_3$) δ 8.00 – 7.92 (m, 2H), 7.76 – 7.48 (m, 2H), 7.43 (t, *J* = 7.7 Hz, 2H), 7.16 (s, 2H), 4.55 (s, 1H), 3.92 (s, 6H), 3.87 (s, 3H), 3.46 (s, 2H), 3.22 – 2.69 (m, 2H), 2.11 – 1.88 (m, 2H), 1.87 – 1.75 (m, 1H), 1.58 (s, 1H). ^{13}C NMR (100 MHz, $CDCl_3$) δ 166.5, 165.0, 153.2, 140.8, 133.4, 130.0, 129.4, 129.1, 128.6, 104.6, 61.0, 61.0, 56.6, 56.4, 47.0, 27.8, 21.4. HRMS (FAB$^+$) m/z calcd. For $[C_{22}H_{27}N_2O_6]^+$: 415.1869, found : 415.1871.

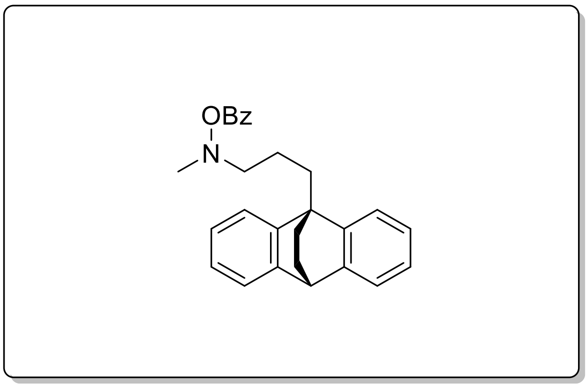

***N*-(3-((9*R*,10*R*)-9,10-Ethanoanthracen-9(10*H*)-yl)propyl)-*O*-benzoyl-*N*-methylhydroxylamine (2q).**

Prepared according to GP2. Hydroxylamine was prepared according to the reference.[21] Purified with flash column chromatography (CH_2Cl_2:Hx = 3:2 to CH_2Cl_2 (100%)). From hydroxylamine (487 g, 1.66 mmol), compound **2q** (540 mg, 82%) was obtained. White solid.

^{1}H NMR (400 MHz, $CDCl_3$) δ 8.25 – 8.01 (m, 2H), 7.60 (tt, *J* = 6.9, 1.2 Hz, 1H), 7.49 (t, *J* = 7.7 Hz, 2H), 7.28 (dd, *J* = 7.1, 1.5 Hz, 4H), 7.15 – 7.01 (m, 4H), 4.29 (t, *J* = 2.6 Hz, 1H), 3.34 (t, *J* = 6.5 Hz, 2H), 3.07 (s, 3H), 2.73 – 2.47 (m, 2H), 2.19 (dq, *J* = 14.5, 7.1 Hz, 2H), 1.95 – 1.76 (m, 2H), 1.68 – 1.51 (m, 2H). ^{13}C NMR (100 MHz, $CDCl_3$) δ 165.2, 145.2, 144.9, 133.1, 129.5, 129.3, 128.5, 125.3, 125.2, 123.3, 121.3, 62.1, 47.4, 44.7, 44.5, 29.7, 28.7, 27.6, 22.6. HRMS (EI$^+$) m/z calcd. For $[C_{27}H_{27}NO_2]^+$: 397.2042, found : 397.2043.

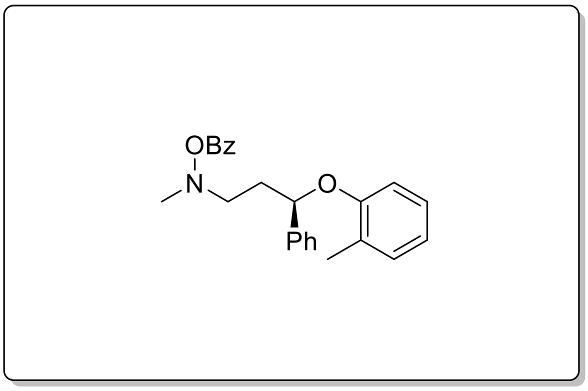

(*R*)-*O*-Benzoyl-*N*-methyl-*N*-(3-phenyl-3-(*o*-tolyloxy)propyl)hydroxylamine (2r).

Prepared according to GP1. Purified with flash column chromatography (CH_2Cl_2:Hx = 1:2 to CH_2Cl_2 (100%)). From amine HCl salt source (876 mg g, 3.0 mmol), compound **2r** (555 mg, 74%) was obtained. Yellow oil.

^{1}H NMR (400 MHz, $CDCl_3$) δ 7.99 (dd, *J* = 8.3, 1.3 Hz, 2H), 7.56 (tt, *J* = 7.0, 1.3 Hz, 1H), 7.47 – 7.39 (m, 2H), 7.36 – 7.27 (m, 4H), 7.25 – 7.18 (m, 1H), 7.16 – 7.05 (m, 1H), 6.98 – 6.89 (m, 1H), 6.76 (td, *J* = 7.4, 0.8 Hz, 1H), 6.63 (d, *J* = 8.1 Hz, 1H), 5.38 (dd, *J* = 8.2, 4.0 Hz, 1H), 3.27 – 3.08 (m, 2H), 2.90

(s, 3H), 2.37 – 2.25 (m, 4H), 2.14 (dtd, *J* = 14.1, 7.3, 4.3 Hz, 1H). ^{13}C NMR (100 MHz, $CDCl_3$) δ 165.4, 156.0, 141.8, 133.2, 130.7, 129.6, 129.3, 128.7, 128.6, 127.7, 127.1, 126.8, 125.9, 120.4, 113.0, 77.4, 57.8, 47.5, 36.5, 16.7. HRMS (EI$^+$) m/z calcd. For $[C_{24}H_{25}NO_3]^+$: 375.1834, found : 375.1832.

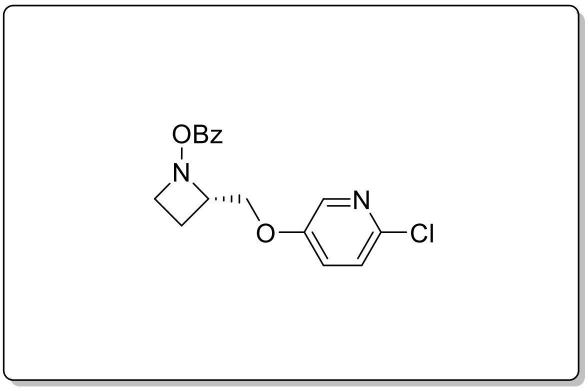

(*S*)-2-(((6-Chloropyridin-3-yl)oxy)methyl)azetidin-1-yl benzoate (2t).

Prepared according to GP1. Purified with flash column chromatography (EA:Hx = 1:3). From amine source (512 g, 2.6 mmol), compound **2t** (124 mg, 18%) was obtained. Colorless oil.

^{1}H NMR (400 MHz, $CDCl_3$) δ 8.05 (dd, *J* = 2.9, 0.7 Hz, 1H), 7.96 (d, *J* = 7.2 Hz, 2H), 7.61 – 7.52 (m, 1H), 7.43 (t, *J* = 7.7 Hz, 2H), 7.26 – 7.16 (m, 2H), 4.28 – 4.21 (m, 2H), 4.00 (t, *J* = 6.6 Hz, 1H), 3.71 (q, *J* = 9.5 Hz, 1H), 2.41 – 2.27 (m, 1H), 2.19 – 2.06 (m, 1H), 1.25 – 1.16 (m, 1H). ^{13}C NMR (100 MHz, $CDCl_3$) δ 165.1, 154.3, 143.0, 137.1, 133.4, 129.5, 128.8, 128.6, 125.2, 124.5, 69.9, 69.2, 56.6, 17.4. HRMS (EI$^+$) m/z calcd. For $[C_{16}H_{15}ClN_2O_3]^+$: 318.0771, found : 318.0774.

III. General Procedures for Migratory Hydroamination of Unactivated Alkenes.

General procedure for migratory hydroamination (GP3)

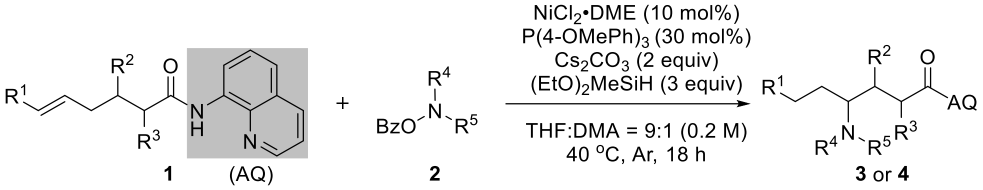

To a flame-dried 12 mL test tube equipped with a Teflon-coated magnetic bar were added alkene substrate (0.10 mmol), amine-*O*-benzoate (0.20 mmol), tris(4-methoxyphenyl)phosphine (10.6 mg, 0.030 mmol). The test tube was sealed with a PTFE/silicon septa cap, which was pierced by a 22-gauge needle. The sealed test tube was placed into an argon-filled glovebox. In glovebox, Cs_2CO_3 (65.2 mg, 0.20 mmol) and $NiCl_2$(DME) (2.2 mg, 0.010 mmol) and were added to the test tube. The reaction mixture was diluted with THF:DMA = 9:1 (0.5 mL, 0.2 M) and stirred at room temperature for 5 min. After addition of $(EtO)_2$MeSiH (48.1 μL, 0.30 mmol), the reaction test tube was sealed with septa cap, and removed from the glovebox. The reaction mixture was stirred at 40 °C for 18 h. The reaction mixture was monitored by TLC. After disappearance of starting material, the reaction mixture was diluted with 50 mL of ethyl acetate and washed with aqueous $NaHCO_3$ (2 × 25 mL) and brine (25 mL). The combined organic layer was dried over Na_2SO_4. After removal of solvent, the residue was purified by flash chromatography on silica gel to give a corresponding desired product compound.

General procedure for migratory hydroamination (GP4)

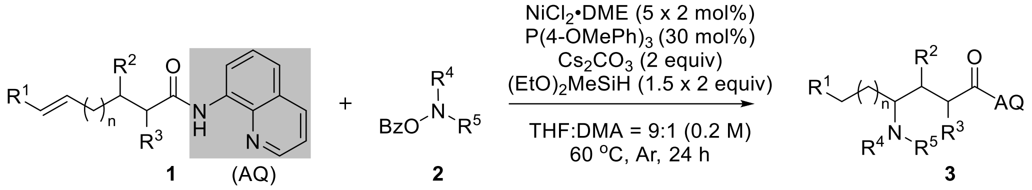

To a flame-dried 12 mL test tube equipped with a Teflon-coated magnetic bar were added alkene substrate (0.10 mmol), amine-*O*-benzoate (0.125 mmol), tris(4-methoxyphenyl)phosphine (10.6 mg, 0.030 mmol). The test tube was sealed with a PTFE/silicon septa cap, which was pierced by a 22-gauge needle. The sealed test tube was placed into an argon-filled glovebox. In glovebox, Cs_2CO_3 (65.2 mg, 0.20 mmol) and $NiCl_2$(DME) (1.1 mg, 0.005 mmol) and were added to the test tube. The reaction mixture was diluted with THF:DMA = 9:1 (0.5 mL, 0.2 M) and stirred at room temperature for 5 min. After addition of $(EtO)_2$MeSiH (24.0 μL, 0.15 mmol), the reaction test tube was sealed with septa cap. The reaction mixture was stirred at 60 °C for 18 h. Amine-*O*-benzoate (0.125 mmol), $NiCl_2$(DME) (1.1 mg, 0.005 mmol) and $(EtO)_2$MeSiH (24.0 μL, 0.15 mmol) were added more to the test tube, and removed from the glovebox. The reaction mixture was stirred at 60 °C for 6 h. The reaction mixture was monitored by TLC. After disappearance of starting material, the reaction mixture was diluted with

50 mL of ethyl acetate and washed with aqueous $NaHCO_3$ (2 × 25 mL) and brine (25 mL). The combined organic layer was dried over Na_2SO_4. After removal of solvent, the residue was purified by flash chromatography on silica gel to give a corresponding desired product compound.

General procedure for migratory hydroamination (GP5)

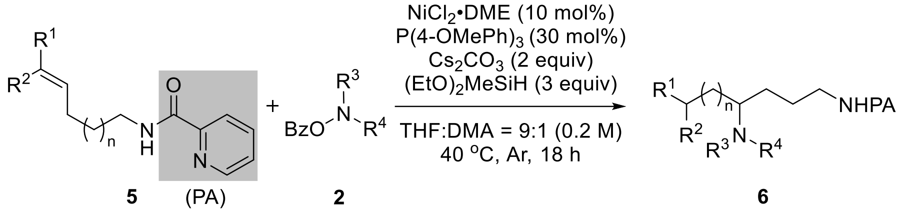

To a flame-dried 12 mL test tube equipped with a Teflon-coated magnetic bar were added alkene substrate (0.10 mmol), amine-*O*-benzoate (0.20 mmol), tris(4-methoxyphenyl)phosphine (10.6 mg, 0.030 mmol). The test tube was sealed with a PTFE/silicon septa cap, which was pierced by a 22-gauge needle. The sealed test tube was placed into an argon-filled glovebox. In glovebox, Cs_2CO_3 (65.2 mg, 0.20 mmol) and $NiCl_2$(DME) (2.2 mg, 0.010 mmol) and were added to the test tube. The reaction mixture was diluted with THF:DMA = 9:1 (0.5 mL, 0.2 M) and stirred at room temperature for 5 min. After addition of $(EtO)_2MeSiH$ (48.1 μL, 0.30 mmol), the reaction test tube was sealed with septa cap, and removed from the glovebox. The reaction mixture was stirred at 40 °C for 18 h. The reaction mixture was monitored by TLC. After disappearance of starting material, the reaction mixture was diluted with 50 mL of ethyl acetate and washed with aqueous $NaHCO_3$ (2 × 25 mL) and brine (25 mL). The combined organic layer was dried over Na_2SO_4. After removal of solvent, the residue was purified by flash chromatography on silica gel to give a corresponding desired product compound.

1 mmol scale-up procedure for migratory hydroamination (3a)

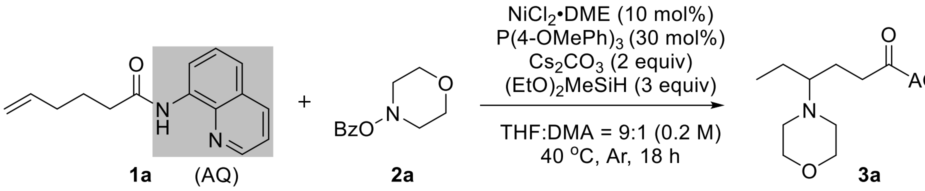

To a flame-dried 50 mL round bottom flask equipped with a Teflon-coated magnetic bar were added *N*-(quinolin-8-yl)hex-5-enamide (240 mg, 1.0 mmol), morpholino benzoate (414 mg, 2.0 mmol) and tris(4-methoxyphenyl)phosphine (106 mg, 0.3 mmol). The round bottom flask was sealed with a septum, which was pierced by a 22-gauge needle. In glovebox, Cs_2CO_3 (652 mg, 2.0 mmol) and $NiCl_2$(DME) (22.0 mg, 0.10 mmol) and were added. The reaction mixture was diluted with THF:DMA = 9:1 (5.0 mL, 0.2 M) and stirred at room temperature for 5 min. After addition of $(EtO)_2MeSiH$ (4.81 mL, 3.0 mmol), the round bottom flask was sealed with septum, and removed from the glovebox. The reaction mixture was stirred at 40 °C for 18 h. The reaction mixture was monitored by TLC using EA:Hx = 1:1 as the

mobile phase. After disappearance of starting material, the reaction mixture was diluted with 100 mL of ethyl acetate and washed with aqueous $NaHCO_3$ (2 × 25 mL) and brine (25 mL). The combined organic layer was dried over Na_2SO_4. After removal of solvent, the residue was purified by flash chromatography on silica gel (EA:Hx = 1:1 to MeOH:EA:Hx = 1:9:10) to give a corresponding desired product compound **3a** as yellowish oil (245 mg, 74%).

Ligand screening for alkene isomerization

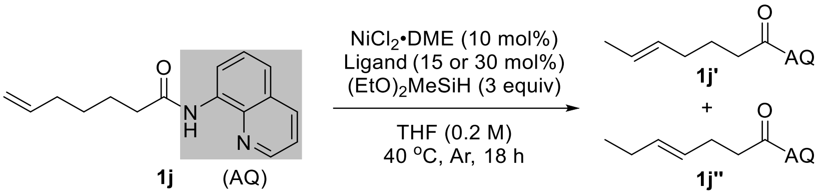

To a flame-dried 12 mL test tube equipped with a Teflon-coated magnetic bar were added N-(quinolin-8-yl)hept-6-enamide (**1j**) (25.4 mg, 0.10 mmol), ligand (0.015 or 0.030 mmol). The test tube was sealed with a PTFE/silicon septa cap, which was pierced by a 22-gauge needle. The sealed test tube was placed into an argon-filled glovebox. In glovebox, $NiCl_2$(DME) (2.2 mg, 0.010 mmol) and were added to the test tube. The reaction mixture was diluted with tetrahydrofuran (0.5 mL, 0.2 M) and stirred at room temperature for 5 min. After addition of $(EtO)_2$MeSiH (48.1 µL, 0.30 mmol), the reaction test tube was sealed with septa cap, and removed from the glovebox. The reaction mixture was stirred at 40 °C for 18 h. The reaction mixture was diluted with 50 mL of ethyl acetate and washed with aqueous $NaHCO_3$ (2 × 25 mL) and brine (25 mL). The combined organic layer was dried over Na_2SO_4. After removal of solvent, the crude mixture was analyzed by ^{1}H NMR.

Supplementary Table 1. Optimization of reaction conditions for migratory hydroamination[a,b]

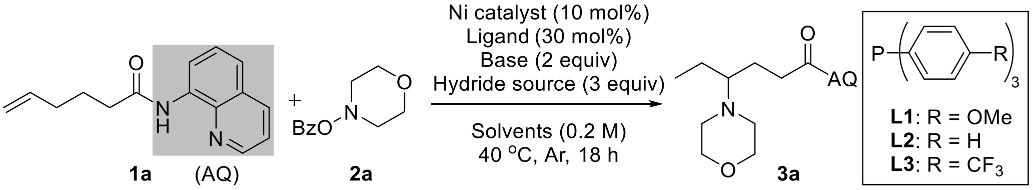

Entry	Ni catalyst	Ligand	Hydride source (3 equiv)	Base (2 equiv)	note	Solvent (0.2 M)	Yield (%)[b]
1[c]	$NiCl_2$(DME)	**L1**	$(EtO)_2$MeSiH	Cs_2CO_3	H_2O (50 mol%)	THF	61(97:3)
2[c]	$NiCl_2$(DME)	**L1**	$(EtO)_2$MeSiH	Cs_2CO_3	H_2O (100 mol%)	THF	55(98:2)
3[c]	$NiCl_2$(DME)	**L1**	$(EtO)_2$MeSiH	Cs_2CO_3	H_2O (150 mol%)	THF	45(98:2)
4	$NiCl_2$(DME)	**L1**	$(EtO)_2$MeSiH	Cs_2CO_3	H_2O (50 mol%)	THF	75(93:7)
**5[d]**	$NiCl_2$(DME)	**L1**	$(EtO)_2$MeSiH	Cs_2CO_3	H_2O (50 mol%)	THF	73(93:7)

6	$NiCl_2$(DME)	**L1**	$(EtO)_2MeSiH$	Cs_2CO_3	H_2O (50 mol%) 50 °C	THF	74(92:8)
**7**	**$NiCl_2$(DME)**	**L1**	**$(EtO)_2MeSiH$**	**Cs_2CO_3**	**-**	**THF:DMA (9:1)**	**78(95:5)**
8	$NiCl_2$(DME)	**L1**	$(EtO)_2MeSiH$	Cs_2CO_3	-	THF	72(92:8)
9	$NiCl_2$(DME)	**L1**	$(EtO)_2MeSiH$	Cs_2CO_3	-	THF:DMA (19:1)	74(93:7)
10	$NiCl_2$(DME)	**L1**	$(EtO)_2MeSiH$	Cs_2CO_3	-	THF:DMA (4:1)	73(96:4)
11	$NiCl_2$(DME)	**L1**	$(EtO)_2MeSiH$	Cs_2CO_3	-	DMA	59(85:15)
12	$Ni(COD)_2$	**L1**	$(EtO)_2MeSiH$	Cs_2CO_3	-	THF:DMA (9:1)	70(97:3)
13	$Ni(acac)_2$	**L1**	$(EtO)_2MeSiH$	Cs_2CO_3	-	THF:DMA (9:1)	67(90:10)
14	$Ni(PPh)_2Cl_2$	-	$(EtO)_2MeSiH$	Cs_2CO_3	-	THF:DMA (9:1)	66(91:9)
15	$NiBr_2$(DME)	**L1**	$(EtO)_2MeSiH$	Cs_2CO_3	-	THF:DMA (9:1)	77(94:6)
16	$NiCl_2$(DME)	**-**	$(EtO)_2MeSiH$	Cs_2CO_3	-	THF:DMA (9:1)	24(96:4)
17	$NiCl_2$(DME)	**L2**	$(EtO)_2MeSiH$	Cs_2CO_3	-	THF:DMA (9:1)	66(91:9)
18	$NiCl_2$(DME)	**L3**	$(EtO)_2MeSiH$	Cs_2CO_3	-	THF:DMA (9:1)	49(94:6)
19	$NiCl_2$(DME)	**L1**	$(EtO)_2MeSiH$	Rb_2CO_3	-	THF:DMA (9:1)	72(85:15)
20	$NiCl_2$(DME)	**L1**	$(EtO)_2MeSiH$	K_2CO_3	-	THF:DMA (9:1)	22(64:36)
21	$NiCl_2$(DME)	**L1**	$(EtO)_2MeSiH$	Na_2CO_3	-	THF:DMA (9:1)	trace
22	$NiCl_2$(DME)	**L1**	$(EtO)_2MeSiH$	Li_2CO_3	-	THF:DMA (9:1)	trace
23	$NiCl_2$(DME)	**L1**	$(EtO)_2MeSiH$	-	-	THF:DMA (9:1)	trace
24	$NiCl_2$(DME)	**L1**	$(MeO)_2MeSiH$	Cs_2CO_3	-	THF:DMA (9:1)	74(95:5)
25	$NiCl_2$(DME)	**L1**	Ph_2SiH_2	Cs_2CO_3	-	THF:DMA (9:1)	43(98:2)
26	$NiCl_2$(DME)	**L1**	-	Cs_2CO_3	-	THF:DMA (9:1)	0
27	-	**L1**	$(EtO)_2MeSiH$	Cs_2CO_3	-	THF:DMA (9:1)	0
28	$NiCl_2$(DME)	**L1**	$(EtO)_2MeSiH$	Cs_2CO_3	Air	THF:DMA (9:1)	27(81:19)
29	$NiCl_2$(DME)	**L1**	$(EtO)_2MeSiH$	Cs_2CO_3	O_2	THF:DMA (9:1)	0

[a]Reactions were performed by using **1a** (0.1 mmol), **2a** (0.2 mmol), Ni catalyst (10 mol%), ligand (30 mol%), hydride source (0.3 mmol), base (0.2 mmol) and solvent (0.5 mL) at 40 °C for 18 h in a silicon-septa capped test tube under Ar. [b]Yields were determined by ^{1}H NMR spectorscopy. γ-pdt:β-pdt ratio in the parenthesis. [c]**2a** (0.15 mmol), ligand (20 mol%) were used. [d]**2a** (0.25 mmol) was used.

IV. Control Experiments.

Control experiment for alkene isomerization

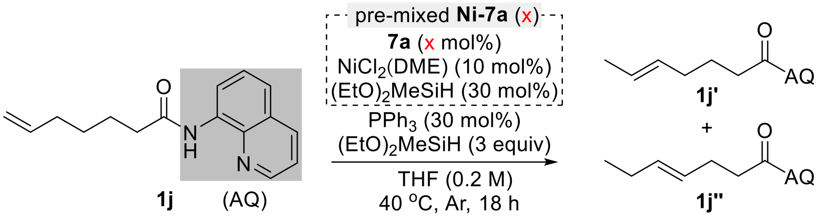

Pre-mixed solution: To a flame-dried 4 mL vial equipped with Teflon-coated magnetic bar was added *N*-(quinolin-8-yl)hex-5-enamide (**7a**) (x × 1.5 mmol). The vial was sealed with a PTFE/silicon septa cap, which was pierced by a 22-gauge needle. The sealed vial was placed into an argon-filled glovebox. In glovebox, $NiCl_2$(DME) (3.3 mg, 0.015 mmol) and were added to the vial. The reaction mixture was diluted with tetrahydrofuran (0.3 mL). After addition of $(EtO)_2$MeSiH (7.2 µL, 0.045 mmol), the reaction mixture was stirred at room temperature for 30 min.

Reaction: To a flame-dried 12 mL test tube equipped with a Teflon-coated magnetic bar were added *N*-(quinolin-8-yl)hept-6-enamide (**1j**) (25.4 mg, 0.10 mmol), PPh_3 (7.9 mg, 0.030 mmol). The test tube was sealed with a PTFE/silicon septa cap, which was pierced by a 22-gauge needle. The sealed test tube was placed into an argon-filled glovebox. In glovebox, The reaction mixture was diluted with tetrahydrofuran (0.3 mL), and **pre-mixed solution** (0.2 mL) was added stirred at room temperature for 5 min. After addition of $(EtO)_2$MeSiH (48.1 µL, 0.30 mmol), the reaction test tube was sealed with septa cap, and removed from the glovebox. The reaction mixture was stirred at 40 °C for 18 h. The reaction mixture was diluted with 50 mL of ethyl acetate and washed with aqueous $NaHCO_3$ (2 × 25 mL) and brine (25 mL). The combined organic layer was dried over Na_2SO_4. After removal of solvent, the crude mixture was analyzed by ^{1}H NMR.

Preparation of d_2-*N*-(quinolin-8-yl)hex-5-enamide (d_2-1a)

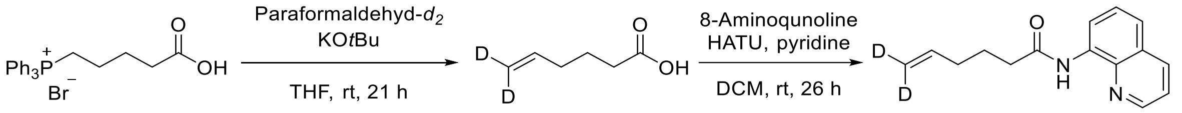

Wittig reaction[10]

To a suspension of (4-carboxybutyl)triphenylphosphonium bromide (4.65 g, 10.5 mmol) in THF (22 mL, 0.3 M) was added KO*t*Bu (2.36 g, 21.0 mmol) portionwise at 0 °C under N_2 atmosphere and the mixture is stirred for 30 min. paraformaldehyde-d_2 (0.224 g, 7.0 mmol) is added portionwise at 0 ºC. The reaction mixutre was allowed to warm to room temperature and stirred for 21 h. After the reaction mixture was acidified with 1 N HCl solution (40 mL) at 0 ºC and extracted with diethyl ether (3 × 40 mL). The combined organic layer was washed with water (2 × 50 mL) and brine (50 mL). The

combined organic layer was dried over Na_2SO_4 and concentrated under reduced pressure to give hex-5-enoic-6,6-d2 acid. The residue was used in the next step without further purification.

Amide bond formation[6]

To a solution of hex-5-enoic-6,6-d_2 acid (7.0 mmol) in DCM (28 mL, 0.25 M) were added 8-aminoquinoline (1.01 g, 7.0 mmol), pyridine (1.13 mL, 14.0 mmol) and HATU (3.99 g, 10.5 mmol). The reaction mixture was stirred at room temperature for 26 h. The reaction mixture was monitored by TLC using ethyl acetate:hexane = 1:9 as the mobile phase. After disappearance of starting material, the reaction mixture was concentrated under reduced pressure. The residue was diluted with ethyl acetate (70 mL) and washed with aqueous $NaHCO_3$ (3 × 100 mL) and brine (100 mL). The combined organic layer was dried over Na_2SO_4. After the removal of solvent, the residue was purified by flash chromatography on silica gel (ethyl acetate:hexane = 1:9) to give d_2-*N*-(quinolin-8-yl)hex-5-enamide as colorless oil (799 mg, 47% for 2 steps)

^{1}H NMR (400 MHz, $CDCl_3$) δ 9.80 (s, 1H), 8.97 – 8.63 (m, 2H), 8.15 (dd, *J* = 8.3, 1.7 Hz, 1H), 7.53 (dd, *J* = 8.3, 7.4 Hz, 1H), 7.49 (dd, *J* = 8.3, 1.7 Hz, 1H), 7.44 (dd, *J* = 8.3, 4.2 Hz, 1H), 5.92 – 5.69 (m, 1H), 2.57 (dd, *J* = 8.0, 7.1 Hz, 2H), 2.26 – 2.05 (m, 2H), 2.01 – 1.81 (m, 2H). HRMS (EI^+) m/z calcd. For $[C_{15}H_{14}D_2N_2O]^+$: 242.1388, found :242.1385.

Deuterium migration experiment

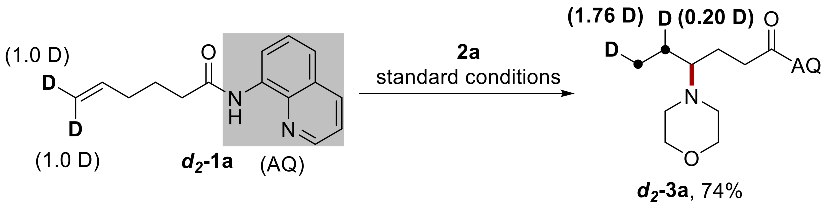

To a flame-dried 12 mL test tube equipped with a Teflon-coated magnetic bar were added d_2-*N*-(quinolin-8-yl)hex-5-enamide (24.0 mg, 0.10 mmol), morpholino benzoate (41.4 mg, 0.20 mmol), tris(4-methoxyphenyl)phosphine (10.6 mg, 0.030 mmol). The test tube was sealed with a PTFE/silicon septa cap, which was pierced by a 22-gauge needle. The sealed test tube was placed into an argon-filled glovebox. In glovebox, Cs_2CO_3 (0.20 mmol) and $NiCl_2$(DME) (2.2 mg, 0.010 mmol) and were added to the test tube. The reaction mixture was diluted with THF:DMA = 9:1 (0.5 mL, 0.2 M) and stirred at room temperature for 5 min. After addition of $(EtO)_2$MeSiH (48.1 µL, 0.30 mmol), the reaction test tube was sealed with septa cap, and removed from the glovebox. The reaction mixture was stirred at 40 °C for 18 h. The reaction mixture was monitored by TLC. After disappearance of starting material, the reaction mixture was diluted with 50 mL of ethyl acetate and washed with aqueous $NaHCO_3$ (2 × 25 mL) and brine (25 mL). The combined organic layer was dried over Na_2SO_4. After removal of solvent, the residue was purified by flash chromatography on silica gel (EA:Hx = 1:1 to MeOH:EA:Hx = 1:9:10) to give a corresponding desired product compound, d_2-4-morpholino-*N*-(quinolin-8-yl)hexanamid (***d*₂-**

3a) as colorless oil (24.5 mg, 74%).

^{1}H NMR (600 MHz, Acetonitrile-d_3) δ 9.85 (s, 1H), 8.83 (dd, J = 4.1, 1.7 Hz, 1H), 8.73 (dd, J = 7.2, 1.7 Hz, 1H), 8.27 (dd, J = 8.3, 1.7 Hz, 1H), 7.64 – 7.39 (m, 3H), 3.61 – 3.54 (m, 2H), 3.54 – 3.47 (m, 2H), 2.71 – 2.61 (m, 1H), 2.61 – 2.53 (m, 2H), 2.54 – 2.45 (m, 1H), 2.40 – 2.34 (m, 2H), 2.35 – 2.28 (m, 1H), 1.87 – 1.73 (m, 2H), 1.66 – 1.55 (m, 0.91H), 1.23 – 1.11 (m, 0.89H), 0.91 – 0.84 (m, 1.24H). HRMS (EI$^+$) m/z calcd. For $[C_{19}H_{23}D_2N_3O_2]^+$: 329.2072, found : 329.2069.

Crossover experiment

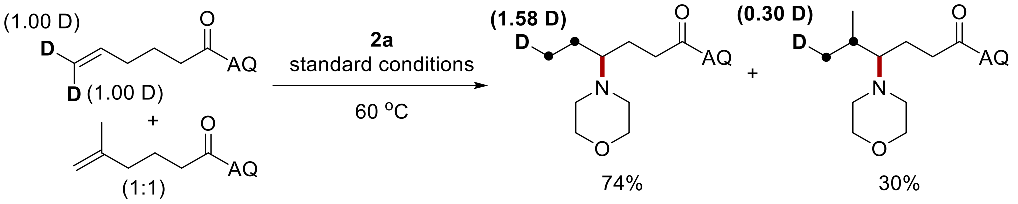

To a flame-dried 12 mL test tube equipped with a Teflon-coated magnetic bar were added d_2-*N*-(quinolin-8-yl)hex-5-enamide (24.2 mg, 0.10 mmol), 5-methyl-*N*-(quinolin-8-yl)hex-5-enamide (25.4 mg, 0.10 mmol), morpholino benzoate (103.6 mg, 0.50 mmol) and tris(4-methoxyphenyl)phosphine (21.1 mg, 0.060 mmol). The test tube was sealed with a PTFE/silicon septa cap, which was pierced by a 22-gauge needle. The sealed test tube was placed into an argon-filled glovebox. In glovebox, Cs_2CO_3 (130.3 mg, 0.40 mmol) and $NiCl_2$(DME) (4.4 mg, 0.020 mmol) and were added to the test tube. The reaction mixture was diluted with THF:DMA = 9:1 (1.0 mL, 0.2 M) and stirred at room temperature for 5 min. After addition of $(EtO)_2$MeSiH (96.1 μL, 0.60 mmol), the reaction test tube was sealed with septa cap, and removed from the glovebox. The reaction mixture was stirred at 60 °C for 18 h. The reaction mixture was monitored by TLC. After disappearance of starting material, the reaction mixture was diluted with 50 mL of ethyl acetate and washed with aqueous $NaHCO_3$ (2 × 25 mL) and brine (25 mL). The combined organic layer was dried over Na_2SO_4. After removal of solvent, the residue was purified by preparative TLC (EA:Hx = 1:3 (1% TEA)) to give a corresponding desired product compound, *d*-4-morpholino-*N*-(quinolin-8-yl)hexanamide (***d*-3a**) as reddish oil (24.3 mg, 74%) and d_2- 5-(methyl-d)-4-morpholino-*N*-(quinolin-8-yl)hexanamide (***d*-3d**) as reddish oil (10.2 mg, 30%).

***d*-3a:** ^{1}H NMR (600 MHz, $CDCl_3$) δ 9.87 (s, 1H), 8.85 – 8.72 (m, 2H), 8.16 (dd, J = 8.3, 1.7 Hz, 1H), 7.53 (t, J = 7.9 Hz, 1H), 7.49 (d, J = 8.1 Hz, 1H), 7.45 (dd, J = 8.3, 4.2 Hz, 1H), 3.70 (ddd, J = 9.8, 6.3, 2.8 Hz, 2H), 3.63 (ddd, J = 10.6, 6.4, 2.7 Hz, 2H), 2.71 (dt, J = 14.8, 7.5 Hz, 1H), 2.64 (ddt, J = 15.5, 11.8, 5.9 Hz, 2H), 2.58 (dd, J = 14.1, 6.7 Hz, 1H), 2.44 (ddd, J = 10.6, 6.5, 2.8 Hz, 2H), 2.36 (tt, J = 9.5, 4.7 Hz, 1H), 1.99 – 1.91 (m, 1H), 1.88 – 1.79 (m, 1H), 1.70 – 1.62 (m, 1H), 1.22 (td, J = 15.0, 7.7 Hz, 1H), 0.95 – 0.85 (m, 1.42H).

***d*-3d:** ^{1}H NMR (600 MHz, $CDCl_3$) δ 9.83 (s, 1H), 8.83 – 8.74 (m, 2H), 8.16 (dd, J = 8.3, 1.6 Hz, 1H), 7.54 (t, J = 7.9 Hz, 1H), 7.49 (d, J = 8.2 Hz, 1H), 7.45 (dd, J = 8.2, 4.2 Hz, 1H), 3.66 (ddd, J = 9.8, 6.3, 2.8 Hz, 2H), 3.59 (ddd, J = 10.5, 6.3, 2.8 Hz, 2H), 2.75 (ddd, J = 10.1, 6.4, 2.7 Hz, 2H), 2.72 – 2.60 (m,

2H), 2.56 (ddd, J = 10.8, 6.5, 2.8 Hz, 2H), 2.26 (dt, J = 9.8, 4.8 Hz, 1H), 2.01 – 1.81 (m, 3H), 0.95 (dd, J = 12.3, 6.8 Hz, 5.70H). HRMS (FAB$^+$) m/z calcd. For $[C_{20}H_{27}DN_3O_2]^+$: 343.2239, found : 343.2240.

Deutration with Ph_2SiD_2

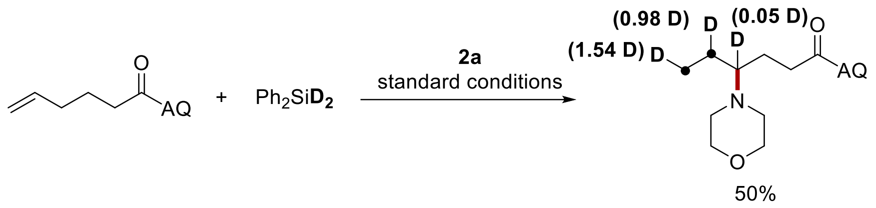

To a flame-dried 12 mL test tube equipped with a Teflon-coated magnetic bar were added *N*-(quinolin-8-yl)hex-5-enamide (24.0 mg, 0.10 mmol), morpholino benzoate (41.4 mg, 0.20 mmol), tris(4-methoxyphenyl)phosphine (10.6 mg, 0.030 mmol). The test tube was sealed with a PTFE/silicon septa cap, which was pierced by a 22-gauge needle. The sealed test tube was placed into an argon-filled glovebox. In glovebox, Cs_2CO_3 (0.20 mmol) and $NiCl_2$(DME) (2.2 mg, 0.010 mmol) and were added to the test tube. The reaction mixture was diluted with THF:DMA = 9:1 (0.5 mL, 0.2 M) and stirred at room temperature for 5 min. After addition of Ph_2SiD_2 (55.9 µL, 0.30 mmol), the reaction test tube was sealed with septa cap, and removed from the glovebox. The reaction mixture was stirred at 40 ºC for 18 h. The reaction mixture was monitored by TLC. After disappearance of starting material, the reaction mixture was diluted with 50 mL of ethyl acetate and washed with aqueous $NaHCO_3$ (2 × 25 mL) and brine (25 mL). The combined organic layer was dried over Na_2SO_4. After removal of solvent, the residue was purified by flash chromatography on silica gel (EA:Hx = 1:1 to MeOH:EA:Hx = 1:9:10) to give a corresponding desired product compound, d_3-4-morpholino-*N*-(quinolin-8-yl)hexanamid (***d*₃-3a**) as colorless oil (16.4 mg, 50%).

^{1}H NMR (600 MHz, Acetonitrile-d_3) 9.85 (s, 1H), 8.84 (dd, J = 4.2, 1.6 Hz, 1H), 8.73 (dd, J = 7.2, 1.7 Hz, 1H), 8.28 (dd, J = 8.4, 1.7 Hz, 1H), 7.60 – 7.51 (m, 3H), 3.61 – 3.54 (m, 2H), 3.54 – 3.46 (m, 2H), 2.74 – 2.63 (m, 1H), 2.61 – 2.54 (m, 2H), 2.55 – 2.40 (m, 1H), 2.41 – 2.35 (m, 2H), 1.88 – 1.74 (m, 2H), 1.67 – 1.57 (m, 0H), 1.23 – 1.14 (m, 1H), 0.95 – 0.84 (m, 1H). HRMS (EI$^+$) m/z calcd. For $[C_{19}H_{22}D_3N_3O_2]^+$: 330.2135, found : 330.2137 or $[C_{19}H_{21}D_4N_3O_2]^+$: 331.2198, found : 331.2195.

The use of *α,β*- and *β,γ*-Alkene substrates

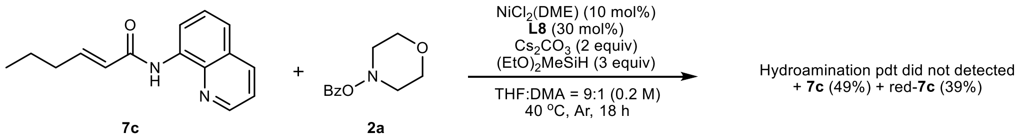

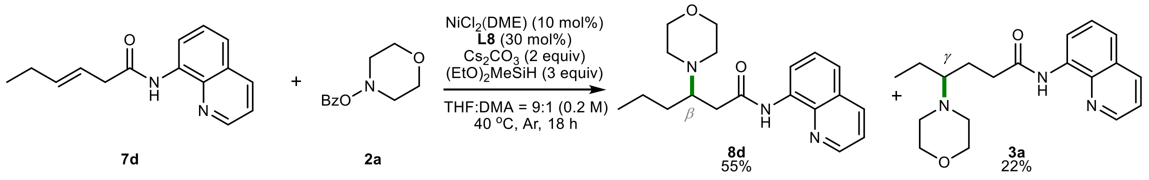

V. Kinetic Analysis.

Reaction set up & analysis (Time profiling)

To a flame-dried 25 mL 2-neck round bottom flask equipped with a Teflon-coated magnetic bar were added *N*-(quinolin-8-yl)hex-5-enamide (**1a**) (144.2 mg, 0.60 mmol), morpholino benzoate (**2a**) (248.7 mg, 1.2 mmol), tris(4-methoxyphenyl)phosphine (**L8**) (63.4 mg, 0.18 mmol) and dimethyl terephthalate (14.6 mg, 0.075 mmol) as internal standard. The test tube was sealed with a septum, which was pierced by a 22-gauge needle. The sealed flask was placed into an argon-filled glovebox. In glovebox, Cs_2CO_3 (390.7 mg, 1.2 mmol) and $NiCl_2$(DME) (13.2 mg, 0.060 mmol) was added to the flask. The reaction test tube was sealed with septum, and removed from the glovebox. The atmosphere was evacuated and backfilled with argon-filled balloon. The reaction mixture was diluted with THF:DMA = 9:1 (3.0 mL, 0.2 M) and stirred at room temperature for 5 min. After addition of $(EtO)_2MeSiH$ (288 μL, 1.8 mmol) and positioned at heat-block (40 ºC), the stopwatch was started. The aliquot (50 μL) of the reaction mixture were taken every 20 min for 300 min. an aliquot was immediately diluted with 3 mL of ethyl acetate and washed with aqueous $NaHCO_3$ (1 × 3 mL). The combined organic layer was dried over Na_2SO_4. After removal of solvent, the product composition was analyzed by ^1H NMR.

Reaction set up & analysis (Different excess experiment-standard condition)

To a flame-dried 25 mL 2-neck round bottom flask equipped with a Teflon-coated magnetic bar were added *N*-(quinolin-8-yl)hex-5-enamide (**1a**) (96.1 mg, 0.40 mmol), morpholino benzoate (**2a**) (165.8 mg, 0.8 mmol), tris(4-methoxyphenyl)phosphine (**L8**) (42.3 mg, 0.12 mmol) and dimethyl terephthalate (9.7 mg, 0.05 mmol) as internal standard. The test tube was sealed with a septum, which was pierced by a 22-gauge needle. The sealed flask was placed into an argon-filled glovebox. In glovebox, Cs_2CO_3 (260.7 mg, 0.8 mmol) and $NiCl_2$(DME) (8.8 mg, 0.040 mmol) was added to the flask. The reaction test tube was sealed with septum, and removed from the glovebox. The atmosphere was evacuated and backfilled with argon-filled balloon. The reaction mixture was diluted with THF:DMA = 9:1 (2.0 mL, 0.2 M) and stirred at room temperature for 5 min. After addition of $(EtO)_2MeSiH$ (192 μL, 1.2 mmol) and positioned at heat-block (40 ºC), the stopwatch was started. The aliquot (50 μL) of the reaction mixture were taken every 15 min for 120 min (+ further extra time progress). an aliquot was immediately diluted with 3 mL of ethyl acetate and washed with aqueous $NaHCO_3$ (3 mL). The combined organic layer was dried over Na_2SO_4. After removal of solvent, the product composition was analyzed by ^1H NMR.

Overall result

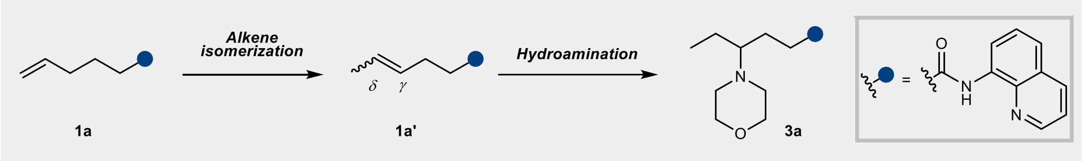

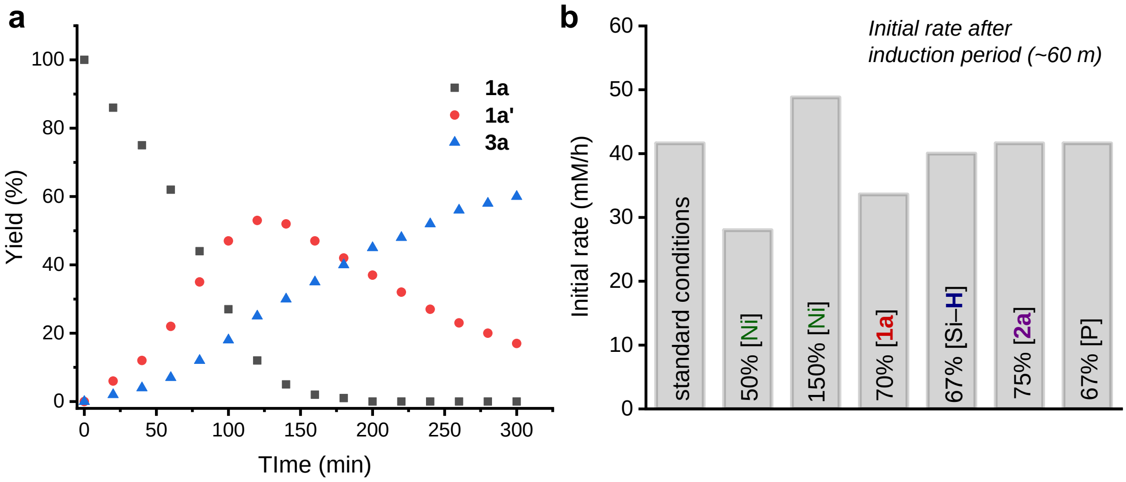

Supplementary Figure 1. Kinetic anaylsis. a Time profiling. **b** Different excess experiment.

Time profiling.

Supplementary Table 2. Experimental data of time profiling.

Time (min)	NMR Y (%)			Time (min)	NMR Y (%)		
	**1a**	**1a'**	**3a**		**1a**	**1a'**	**3a**
0	100	0	0	160	2	47	35
20	84	6	2	180	1	42	40
40	75	12	4	200	0	37	45
60	62	22	7	220	0	32	48
80	44	35	12	240	0	27	52
100	27	47	18	260	0	23	56
120	12	53	25	280	0	20	58
140	5	52	30	300	0	17	60

Different excess experiment

Supplementary Table 3. Detailed condition of different excess experiment.

	Standard Conditions	lower [Ni]	Higher [Ni]	Lower [1a]	Lower [Si–H]	Lower [2a]	Lower [L8]
[Ni]	0.020 M	0.010 M	0.030 M	0.020 M	0.020 M	0.020 M	0.020 M
[**1a**]	0.200 M	0.200 M	0.200 M	0.140 M	0.200 M	0.200 M	0.200 M
[**2a**]	0.400 M	0.400 M	0.400 M	0.400 M	0.400 M	0.300 M	0.400 M
[Si–H]	0.600 M	0.600 M	0.600 M	0.600 M	0.400 M	0.600 M	0.600 M
[**L8**]	0.060 M	0.060 M	0.060 M	0.060 M	0.060 M	0.060 M	0.040 M

Supplementary Table 4. Different excess experiment on $NiCl_2$(DME).

	Ni 5 mol%	Ni 10 mol%	Ni 15 mol%
Time (min)	[**3a**] (M)	[**3a**] (M)	[**3a**] (M)
0	0	0	0
15	0.002	0.002	0.002
30	0.004	0.004	0.004
45	0.006	0.008	0.008
60	0.012	0.014	0.014
75	0.018	0.024	0.024
90	0.024	0.034	0.036
105	0.032	0.044	0.05
120	0.04	0.056	0.062

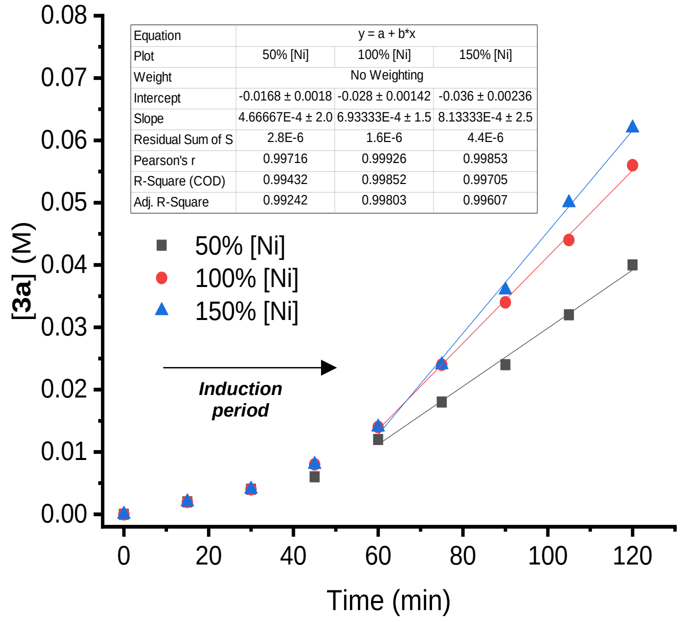

Supplementary Figure 2. Rate profile of different excess experiment on $NiCl_2$(DME).

Supplementary Table 5. Different excess experiment on **1a**.

	**1a** 1.0 equiv	**1a** 0.7 equiv
Time (min)	[**3a**] (M)	[**3a**] (M)
0	0	0
15	0.002	0.002
30	0.004	0.004
45	0.008	0.006
60	0.014	0.008
75	0.024	0.016
90	0.034	0.024
105	0.044	0.032
120	0.056	0.042

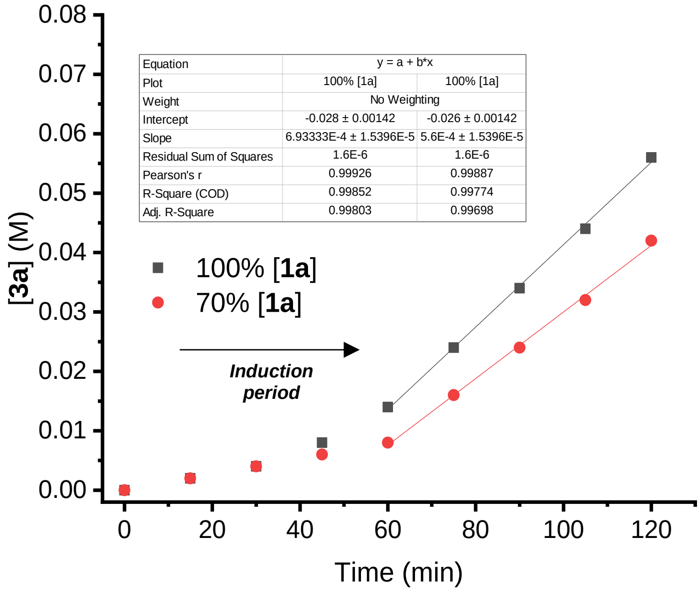

Supplementary Figure 3. Rate profile of different excess experiment on **1a**.

Supplementary Table 6. Different excess experiment on **2a**.

	**2a** 2.0 equiv	**2a** 1.5 equiv
Time (min)	[**3a**] (M)	[**3a**] (M)
0	0	0
15	0.002	0.002
30	0.004	0.006
45	0.008	0.012
60	0.014	0.022
75	0.024	0.032
90	0.034	0.042
105	0.044	0.052
120	0.056	0.064

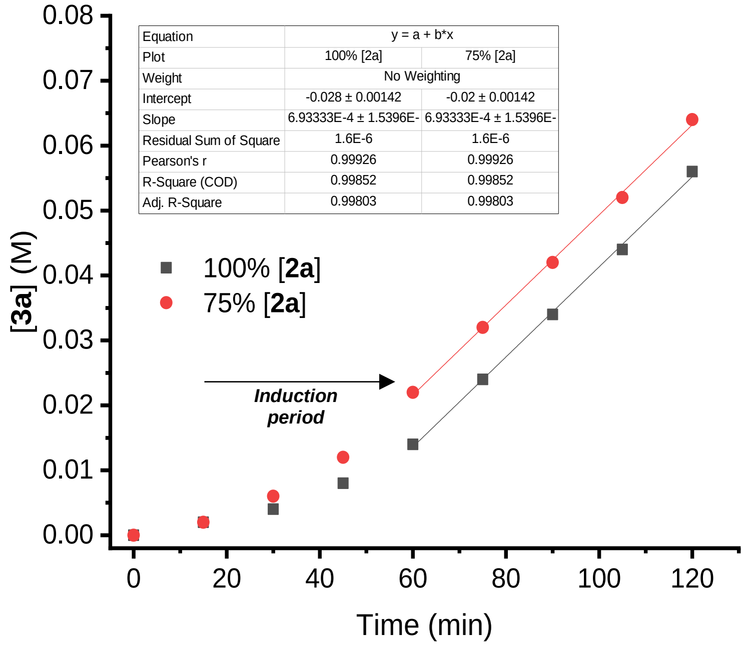

Supplementary Figure 4. Rate profile of different excess experiment on **2a**.

Supplementary Table 7. Different excess experiment on **L8**.

	[**L8**] 30 mol%	[**L8**] 20 mol%
Time (min)	[**3a**] (M)	[**3a**] (M)
0	0	0
15	0.002	0.002
30	0.004	0.006
45	0.008	0.01
60	0.014	0.018
75	0.024	0.028
90	0.034	0.038
105	0.044	0.048
120	0.056	0.06

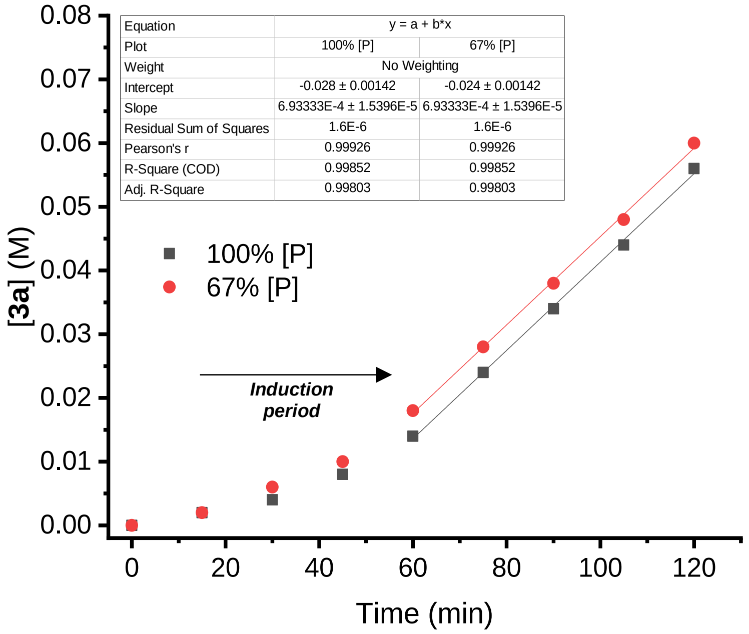

Supplementary Figure 5. Rate profile of different excess experiment on **L8**.

Supplementary Table 8. Different excess experiment on Si–H.

	Si–H 3.0 equiv	Si–H 2.0 equiv
Time (min)	[**3a**] (M)	[**3a**] (M)
0	0	0
15	0.002	0.002
30	0.004	0.004
45	0.008	0.006
60	0.014	0.01
75	0.024	0.016
90	0.034	0.024
105	0.044	0.032
120	0.056	0.044
135		0.056

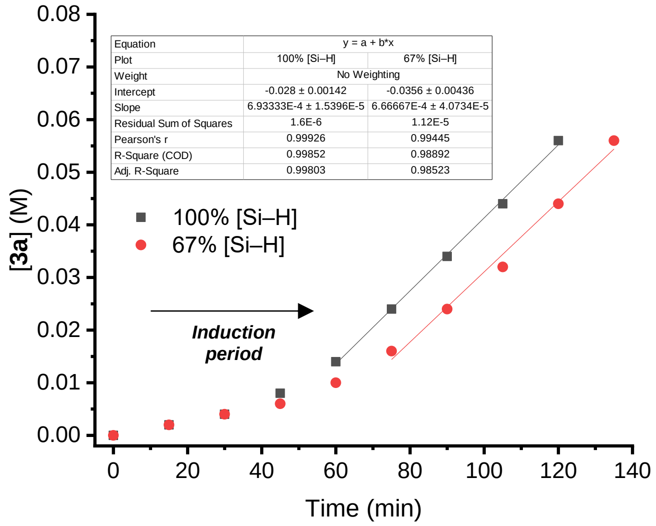

Supplementary Figure 6. Rate profile of different excess experiment on Si–H.

VI. EPR measurements.

A solution of each compound (0.2 M) in dry DMA (0.1 mL) was frozen in liquid nitrogen. X-Band EPR spectra of the frozen samples were obtained under following conditions: Temperature = 100 K, MW power = 2.00 mW, modulation amplitude = 4.0 G, modulation frequency = 100 kHz, and time constant = 0.01 ms.

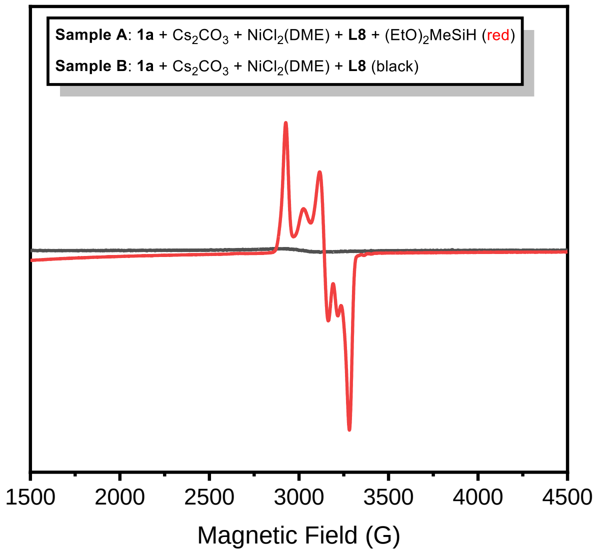

Supplementary Figure 7. Experimental X-band EPR spectrum of **Sample A** (MW frequency = 9.408 GHz) and **Sample B** (MW frequency = 9.413 GHz).

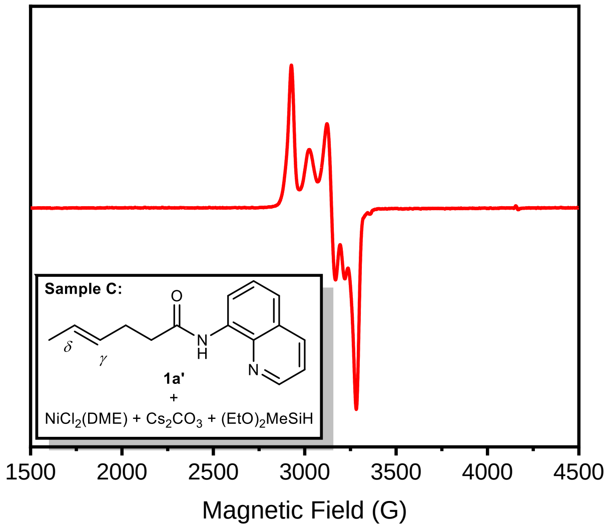

Supplementary Figure 8. Experimental X-band EPR spectrum of **Sample C** (MW frequency = 9.411 GHz).

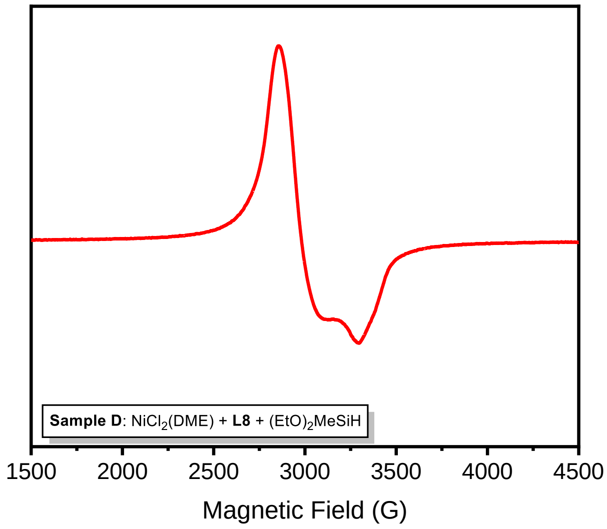

Supplementary Figure 9. Experimental X-band EPR spectrum of **Sample D** (MW frequency = 9.410 GHz).

VII. Computed Results.

Computational details.

All calculations except single point calculations were conducted using (DFT)[30] as implemented in the Jaguar 9.1 suite[31] of ab initio quantum chemistry programs with B3LYP-D3 levels of theory.[32] Geometry optimizations were proceeded using the LACVP** basis set. With the optimized geometries, single point energies were re-evaluated using triple-ζ quality of basis set, cc-pVTZ(-f),[33] where Ni and Cs center was computed with LACV3P** basis set. Analytical vibrational frequencies within the harmonic approximation were calculated using the LACVP** basis to confirm proper convergence to well-defined minima or saddle points on the potential energy surface. Solvation energies were calculated using a self-consistent reaction field (SCRF)[34-36] approach based on accurate numerical solutions of the Poisson-Boltzmann equation and were performed with the LACVP** basis at the optimized gas-phase geometry with the $\varepsilon = 7.6$ for tetrahydrofuran. The Gibbs free energies in solution phase G(sol) were computed with the following protocol.

$$G(sol) = G(gas) + G^{solv} \quad (1)$$

$$G(gas) = H(gas) - TS(gas) \quad (2)$$

$$H(gas) = E(SCF) + ZPE \quad (3)$$

$$\Delta E(SCF) = \Sigma E(SCF) \text{ for products} - \Sigma E(SCF) \text{ for reactants} \quad (4)$$

$$\Delta G(sol) = \Sigma G(sol) \text{ for products} - \Sigma G(sol) \text{ for reactants} \quad (5)$$

G(gas) is the free energy in gas phase; G^{solv} is the free energy of solvation; H(gas) is the enthalpy in gas phase; T is the temperature (313.15K); S(gas) is the entropy in gas phase; E(SCF) is “raw” electronic energy as computed from the SCF procedure which is the self-consistent field energy, and ZPE is the zero point energy. The entropy we refer is specifically vibrational/rotational/translational entropy of the solute(s), and the entropy of the solvent is implicitly comprised in the continuum solvation model.

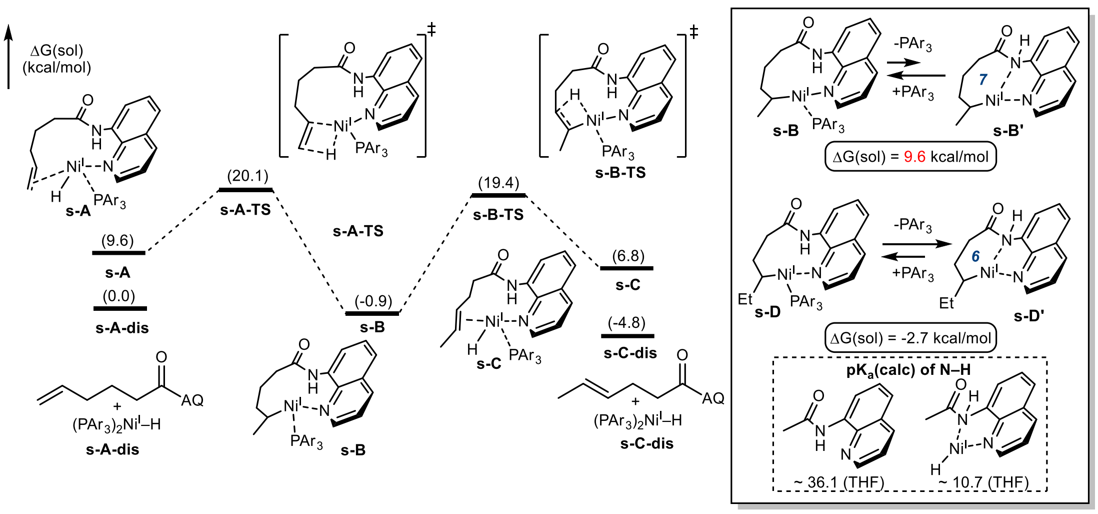

Supplementary Figure 10. Computed energy profile of NiH-catalyzed alkene isomerization.

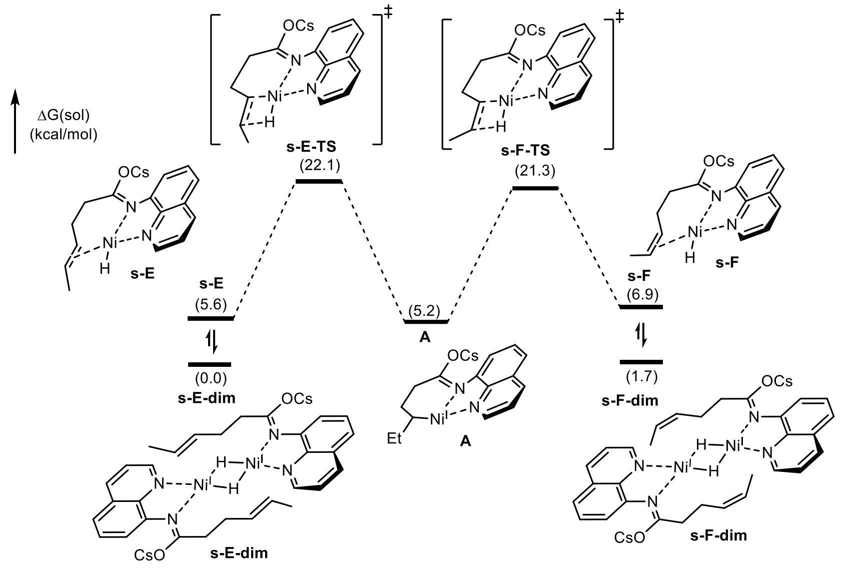

Supplementary Figure 11. Computed energy profile of thermodynamics of monomer-dimer equilibrium and migratory insertion step to formation of 6-membered cycle.

VIII. Compound Characterizations.

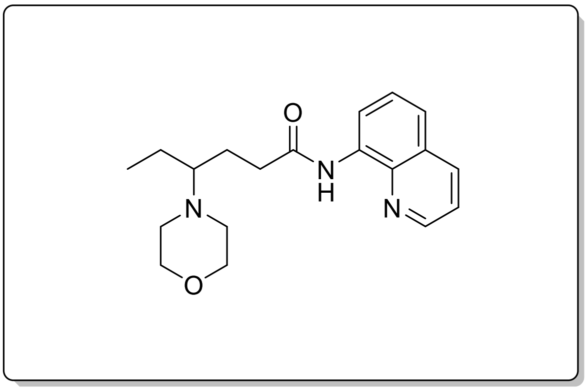

4-Morpholino-*N*-(quinolin-8-yl)hexanamide (3a).

Prepared according to GP3. Monitored by TLC using EA:Hx = 1:1 (Rf = 0.3) as the mobile phase and purified with flash column chromatography on silica gel (EA:Hx = 1:1 to MeOH:EA:Hx=1:9:10). From **1a** (24.0 mg, 0.1 mmol), compound **3a** (25.6 mg, 78%) was obtained. Yellowish oil. ^{1}H NMR (600 MHz, $CDCl_3$) δ 9.87 (s, 1H), 8.80 (m, *J* = 7.0 Hz, 2H), 8.15 (d, *J* = 8.2 Hz, 1H), 7.53 (t, *J* = 7.9 Hz, 1H), 7.49 (d, *J* = 8.2 Hz, 1H), 7.45 (dd, *J* = 8.3, 4.2 Hz, 1H), 3.70 (ddd, *J* = 9.7, 6.4, 2.8 Hz, 2H), 3.63 (ddd, *J* = 10.4, 6.3, 2.8 Hz, 2H), 2.71 (dt, *J* = 14.8, 7.6 Hz, 1H), 2.68 – 2.61 (m, 2H), 2.59 (dt, *J* = 13.9, 6.5 Hz, 1H), 2.44 (ddd, *J* = 10.4, 6.5, 2.7 Hz, 2H), 2.36 (tt, *J* = 9.4, 4.7 Hz, 1H), 2.01 – 1.90 (m, 1H), 1.88 – 1.79 (m, 1H), 1.72 – 1.61 (m, 1H), 1.24 – 1.18 (m, 1H), 0.92 (t, *J* = 7.5 Hz, 3H). ^{13}C NMR (150 MHz, $CDCl_3$) δ 172.5, 148.1, 138.5, 136.5, 134.9, 128.1, 127.6, 121.7, 121.4, 116.6, 67.7, 65.4, 48.8, 35.7, 26.0, 21.2, 12.0. HRMS (EI^+) m/z calcd. For $[C_{19}H_{25}N_3O_2]^+$: 327.1947, found : 327.1949.

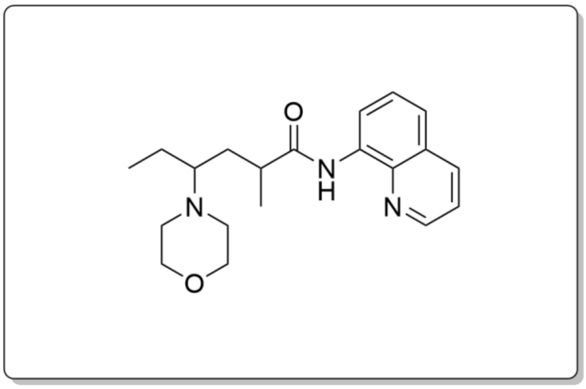

2-Methyl-4-morpholino-*N*-(quinolin-8-yl)hexanamide (3b) (diastereomer 5.4 : 1)

Prepared according to GP3. Monitored by TLC using EA:Hx = 1:3 (Rf = 0.3) as the mobile phase and purified with flash column chromatography on silica gel (EA:Hx = 1:3). From **1b** (25.4 mg, 0.1 mmol), compound **3b** (22.1 mg, 65%) was obtained. Pink soild. ^{1}H NMR (400 MHz, $CDCl_3$) δ 9.83 (s, 1H), 8.80 (d, *J* = 5.9 Hz, 2H), 8.16 (d, *J* = 8.3 Hz, 1H), 7.58 – 7.40 (m, 3H), 3.43 (td, *J* = 6.4, 3.2 Hz, 4H), 2.77 – 2.67 (m, 1H), 2.69 – 2.59 (m, 2H), 2.36 (tt, *J* = 9.6, 3.9 Hz, 3H), 2.02 (ddd, *J* = 14.0, 10.2, 7.6

Hz, 1H), 1.66 (dqd, J = 11.9, 7.6, 4.1 Hz, 1H), 1.49 (dt, J = 14.2, 4.7 Hz, 1H), 1.32 (d, J = 6.9 Hz, 3H), 1.24 – 1.11 (m, 1H), 0.92 (t, J = 7.4 Hz, 3H). ^{13}C NMR (100 MHz, $CDCl_3$) δ 176.0, 148.2, 138.5, 136.6, 135.1, 128.1, 127.7, 121.7, 121.2, 116.5, 77.5, 77.2, 76.8, 67.4, 65.9, 48.8, 41.8, 34.8, 21.1, 19.0, 12.3. HRMS (EI$^+$) m/z calcd. For $[C_{20}H_{27}N_3O_2]^+$: 341.2103, found : 341.2101.

2-Methyl-4-morpholino-*N*-(quinolin-8-yl)hexanamide (3b') (diastereomer 5.4 : 1)

Prepared according to GP3. Monitored by TLC using EA:Hx = 1:3 (Rf = 0.3) as the mobile phase and purified with flash column chromatography on silica gel (EA:Hx = 1:3). From **1b** (25.4 mg, 0.1 mmol), compound **3b'** (4.1 mg, 12%) was obtained. Pink soild. ^{1}H NMR (400 MHz, $CDCl_3$) δ 10.03 (s, 1H), 8.88 (dd, J = 7.4, 1.6 Hz, 1H), 8.78 (dd, J = 4.3, 1.7 Hz, 1H), 8.16 (dd, J = 8.2, 1.7 Hz, 1H), 7.57 – 7.48 (m, 2H), 7.45 (dd, J = 8.3, 4.2 Hz, 1H), 3.91 (ddd, J = 10.8, 6.4, 2.8 Hz, 2H), 3.71 (ddd, J = 10.8, 6.4, 2.8 Hz, 2H), 3.11 – 2.98 (m, 1H), 2.64 (ddd, J = 11.4, 6.4, 2.8 Hz, 2H), 2.39 (ddd, J = 10.4, 5.9, 2.8 Hz, 3H), 1.92 (ddd, J = 15.0, 11.1, 4.3 Hz, 1H), 1.67 (ddd, J = 13.3, 7.6, 3.9 Hz, 1H), 1.51 (ddd, J = 14.7, 11.5, 3.8 Hz, 1H), 1.29 (d, J = 6.8 Hz, 3H), 1.12 (ddt, J = 14.2, 9.2, 7.2 Hz, 1H), 0.85 (t, J = 7.5 Hz, 3H). ^{13}C NMR (100 MHz, $CDCl_3$) δ 175.9, 148.0, 138.6, 136.5, 134.9, 128.2, 127.7, 121.7, 121.4, 116.8, 77.5, 77.2, 76.8, 67.9, 63.4, 48.4, 38.9, 36.3, 20.6, 18.3, 12.0. HRMS (EI$^+$) m/z calcd. For $[C_{20}H_{27}N_3O_2]^+$: 341.2103, found : 341.2101.

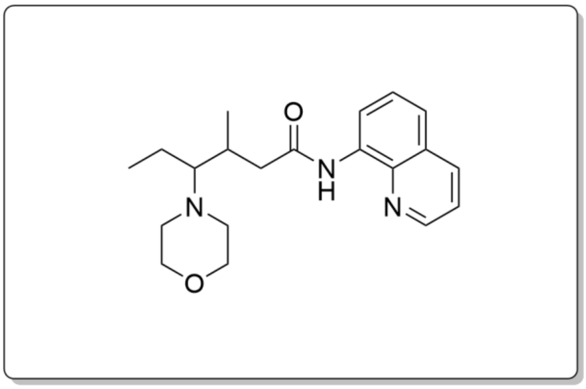

3-Methyl-4-morpholino-*N*-(quinolin-8-yl)hexanamide (3c) (diastereomer 2:1)

Prepared according to GP4. Monitored by TLC using EA:Hx = 1:3 (Rf = 0.3) as the mobile phase and purified with flash column chromatography on silica gel (EA:Hx = 1:3). From **1c** (25.4 mg, 0.1 mmol), compound **3c** (15.5 mg, 45%) was obtained. Colorless oil. ^{1}H NMR (400 MHz, $CDCl_3$) δ 9.84 (s, 1H), 8.81 (dd, J = 7.5, 1.5 Hz, 1H), 8.78 (dd, J = 4.3, 1.7 Hz, 1H), 8.16 (dd, J = 8.3, 1.7 Hz, 1H), 7.57 – 7.47 (m, 2H), 7.45 (dd, J = 8.3, 4.2 Hz, 1H), 3.59 (qdd, J = 10.8, 6.2, 3.0 Hz, 4H), 2.78 – 2.69 (m, 3H), 2.58 – 2.50 (m, 2H), 2.47 (dd, J = 14.1, 6.2 Hz, 1H), 2.40 – 2.28 (m, 1H), 2.14 (ddd, J = 9.3, 6.2, 4.4 Hz, 1H), 1.71 – 1.58 (m, 1H), 1.46 (dqd, J = 14.7, 7.4, 4.4 Hz, 1H), 1.08 (d, J = 6.8 Hz, 3H), 1.00 (t, J = 7.5 Hz, 3H). ^{13}C NMR (100 MHz, $CDCl_3$) δ 171.9, 148.1, 138.5, 136.5, 135.0, 128.2, 127.7, 121.7, 121.3, 116.5, 77.5, 77.2, 76.8, 71.0, 67.6, 49.7, 44.3, 33.1, 20.0, 18.3, 14.1. HRMS (EI$^+$) m/z calcd. For

$[C_{20}H_{27}N_3O_2]^+$: 341.2103, found : 341.2101.

3-Methyl-4-morpholino-*N*-(quinolin-8-yl)hexanamide (3c') (diastereomer 2:1)

Prepared according to GP3. Monitored by TLC using EA:Hx = 1:3 (Rf = 0.3) as the mobile phase and purified with flash column chromatography on silica gel (EA:Hx = 1:3). From **1c** (25.4 mg, 0.1 mmol), compound **3c** (7.5 mg, 22%) was obtained. Pinkish oil. ^{1}H NMR (400 MHz, $CDCl_3$) δ 9.84 (s, 1H), 8.86 – 8.76 (m, 2H), 8.16 (dd, *J* = 8.3, 1.7 Hz, 1H), 7.57 – 7.42 (m, 3H), 3.74 (ddd, *J* = 9.5, 6.2, 2.9 Hz, 2H), 3.69 – 3.62 (m, 2H), 2.85 (dd, *J* = 13.0, 5.5 Hz, 1H), 2.74 – 2.63 (m, 2H), 2.57 (dt, *J* = 12.4, 2.8 Hz, 2H), 2.48 – 2.34 (m, 2H), 2.31 – 2.19 (m, 1H), 1.70 – 1.59 (m, 1H), 1.55 – 1.43 (m, 1H), 1.05 (d, *J* = 6.6 Hz, 3H), 0.98 (t, *J* = 7.4 Hz, 3H). ^{13}C NMR (100 MHz, $CDCl_3$) δ 172.0, 148.3, 138.5, 136.5, 134.7, 128.1, 127.6, 121.8, 121.5, 116.6, 77.5, 77.2, 76.8, 69.0, 67.9, 51.4, 43.0, 33.4, 19.8, 16.8, 13.2. HRMS (EI$^+$) m/z calcd. For $[C_{20}H_{27}N_3O_2]^+$: 341.2103, found : 341.2106.

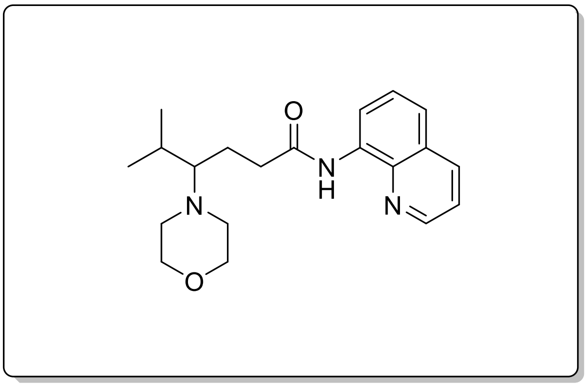

5-Methyl-4-morpholino-*N*-(quinolin-8-yl)hexanamide (3d).

Prepared according to GP4. Monitored by TLC using EA:Hx = 1:2 (Rf = 0.3) as the mobile phase and purified with flash column chromatography on silica gel (EA:Hx = 1:2). From **1d** (25.4 mg, 0.1 mmol), compound **3d** (22.5 mg, 66%) was obtained. Colorless oil. ^{1}H NMR (400 MHz, $CDCl_3$) δ 9.83 (s, 1H), 8.83 – 8.71 (m, 2H), 8.16 (dd, *J* = 8.2, 1.7 Hz, 1H), 7.54 (t, *J* = 7.8 Hz, 1H), 7.49 (dd, *J* = 8.3, 1.6 Hz, 1H), 7.45 (dd, *J* = 8.3, 4.2 Hz, 1H), 3.66 (ddd, *J* = 10.7, 6.2, 3.0 Hz, 2H), 3.59 (ddd, *J* = 10.8, 6.2, 2.9 Hz, 2H), 2.75 (ddd, *J* = 11.1, 6.1, 2.9 Hz, 2H), 2.72 – 2.58 (m, 2H), 2.56 (ddd, *J* = 11.3, 6.1, 3.0 Hz, 2H), 2.26 (dt, *J* = 9.7, 5.0 Hz, 1H), 2.02 – 1.81 (m, 3H), 1.01 – 0.67 (m, 6H). ^{13}C NMR (100 MHz, $CDCl_3$) δ 172.4, 148.2, 138.5, 136.5, 134.8, 128.1, 127.6, 121.7, 121.4, 116.5, 69.6, 67.9, 49.8, 36.6, 28.5, 23.3, 22.5, 20.1. HRMS (EI$^+$) m/z calcd. For $[C_{20}H_{27}N_3O_2]^+$: 341.2103, found : 341.2104.

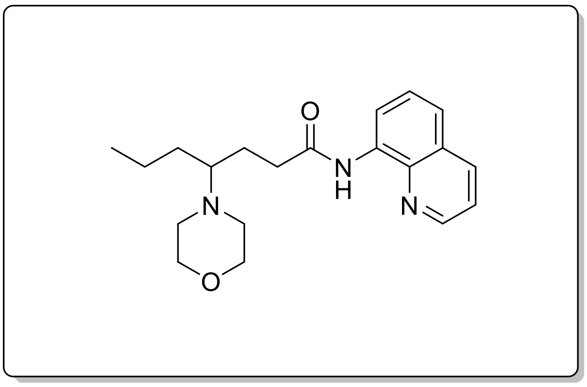

4-Morpholino-*N*-(quinolin-8-yl)heptanamide (3e).

Prepared according to GP4. Monitored by TLC using EA:Hx = 1:2 (Rf = 0.2) as the mobile phase and purified with flash column chromatography on silica gel (EA:Hx = 1:2 to MeOH:EA:Hx = 1:9:20). From **1e** (25.4 mg, 0.1 mmol), compound **3e** (18.7 mg, 55%) was obtained. Colorless oil. ^{1}H NMR (600 MHz, $CDCl_3$) δ 9.87 (s, 1H), 8.88 – 8.48 (m, 2H), 8.16 (dd, *J* = 8.2, 1.7 Hz, 1H), 7.53 (t, *J* = 7.9 Hz, 1H), 7.49 (d, *J* = 8.1 Hz, 1H), 7.45 (dd, *J* = 8.2, 4.2 Hz, 1H), 3.70 (ddd, *J* = 9.7, 6.4, 2.8 Hz, 2H), 3.62 (ddd, *J* = 10.4, 6.4, 2.8 Hz, 2H), 2.71 (dt, *J* = 14.8, 7.5 Hz, 1H), 2.63 (ddd, *J* = 9.8, 6.3, 2.7 Hz, 2H), 2.58 (dt, *J* = 13.9, 6.5 Hz, 1H), 2.49 – 2.39 (m, 3H), 1.97 – 1.80 (m, 2H), 1.63 – 1.51 (m, 1H), 1.47 – 1.33 (m, 1H), 1.34 – 1.23 (m, 1H), 1.22 – 1.15 (m, 1H), 0.91 (t, *J* = 7.3 Hz, 3H). ^{13}C NMR (150 MHz, $CDCl_3$) δ 172.5, 148.1, 138.5, 136.5, 134.8, 128.1, 127.6, 121.7, 121.4, 116.6, 67.7, 63.5, 48.7, 35.7, 30.7, 26.5, 20.7, 14.5. HRMS (EI^+) m/z calcd. For $[C_{20}H_{27}N_3O_2]^+$: 341.2103, found : 341.2105.

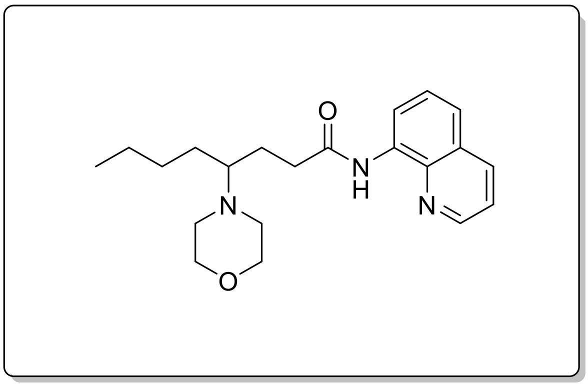

4-Morpholino-*N*-(quinolin-8-yl)octanamide (3f).

Prepared according to GP4. Monitored by TLC using EA:Hx = 1:2 (Rf = 0.2) as the mobile phase and purified with flash column chromatography on silica gel (EA:Hx = 1:2 to MeOH:EA:Hx = 1:9:20). From **1f** (26.8 mg, 0.1 mmol), compound **3f** (19.6 mg, 55%) was obtained. Colorless oil. ^{1}H NMR (600 MHz, $CDCl_3$) δ 9.87 (s, 1H), 8.91 – 8.72 (m, 2H), 8.16 (dd, *J* = 8.3, 1.7 Hz, 1H), 7.54 (t, *J* = 7.9 Hz, 1H), 7.49 (d, *J* = 8.1 Hz, 1H), 7.45 (dd, *J* = 8.2, 4.2 Hz, 1H), 3.70 (ddd, *J* = 9.8, 6.4, 2.8 Hz, 2H), 3.63 (ddd, *J* = 10.4, 6.4, 2.8 Hz, 2H), 2.71 (dt, *J* = 14.8, 7.5 Hz, 1H), 2.64 (ddd, *J* = 9.8, 6.5, 2.7 Hz, 2H), 2.59 (dt, *J* = 13.8, 6.5 Hz, 1H), 2.50 – 2.33 (m, 3H), 1.97 – 1.88 (m, 1H), 1.90 – 1.81 (m, 1H), 1.66 –

1.55 (m, 1H), 1.39 – 1.15 (m, 5H), 0.89 (t, *J* = 6.8 Hz, 3H). ^{13}C NMR (150 MHz, $CDCl_3$) δ 172.5, 148.1, 138.5, 136.5, 134.9, 128.1, 127.6, 121.7, 121.4, 116.6, 67.7, 63.7, 48.7, 35.7, 29.8, 28.2, 26.6, 23.1, 14.2. HRMS (EI^+) m/z calcd. For $[C_{21}H_{29}N_3O_2]^+$: 355.2260, found : 355.2261.

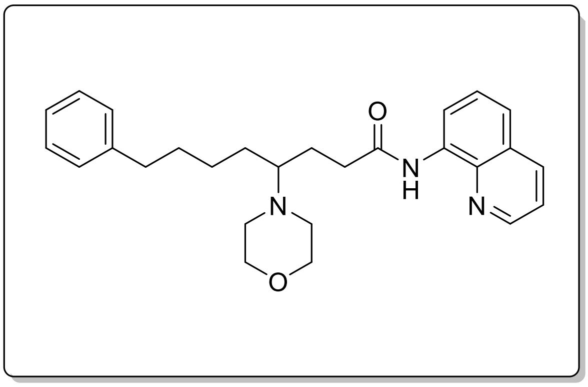

4-Morpholino-8-phenyl-*N*-(quinolin-8-yl)octanamide (3g).

Prepared according to GP4. Monitored by TLC using EA:Hx = 1:2 (Rf = 0.2) as the mobile phase and purified with flash column chromatography on silica gel (EA:Hx = 1:2). From **1g** (34.4 mg, 0.1 mmol), compound **3g** (24.1 mg, 56%) was obtained. Colorless oil. ^{1}H (400 MHz, CD_2Cl_2) δ 9.85 (s, 1H), 8.81 (dd, *J* = 4.2, 1.7 Hz, 1H), 8.77 (dd, *J* = 6.4, 2.6 Hz, 1H), 8.20 (dd, *J* = 8.2, 1.7 Hz, 1H), 7.58 – 7.48 (m, 2H), 7.48 (dd, *J* = 8.3, 4.2 Hz, 1H), 7.29 – 7.21 (m, 2H), 7.20 – 7.10 (m, 3H), 3.65 (ddd, *J* = 10.8, 6.3, 3.0 Hz, 2H), 3.58 (ddd, *J* = 10.8, 6.2, 2.9 Hz, 2H), 2.75 – 2.49 (m, 6H), 2.50 – 2.37 (m, 3H), 1.93 – 1.78 (m, 2H), 1.73 – 1.56 (m, 3H), 1.49 – 1.36 (m, 1H), 1.37 – 1.26 (m, 1H), 1.29 – 1.17 (m, 1H). ^{13}C NMR (100 MHz, CD_2Cl_2) δ 172.7, 148.7, 143.4, 138.9, 136.9, 135.5, 128.9, 128.7, 128.6, 127.8, 126.1, 122.2, 121.7, 116.6, 68.0, 64.0, 49.2, 36.4, 36.0, 32.3, 28.7, 27.6, 26.8. HRMS (EI^+) m/z calcd. For $[C_{27}H_{33}N_3O_2]^+$: 431.2573, found : 431.2570.

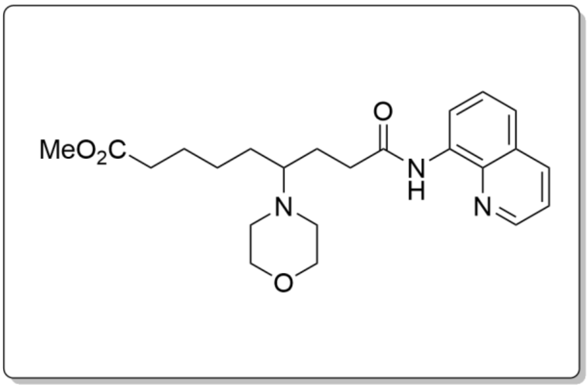

Methyl 6-morpholino-9-oxo-9-(quinolin-8-ylamino)nonanoate (3h)

Prepared according to GP4. Monitored by TLC using EA:Hx = 1:1 (Rf = 0.3) as the mobile phase and purified with flash column chromatography on silica gel (EA:Hx = 1:1). From **1h** (32.6 mg, 0.1 mmol), compound **3h** (24.9 mg, 61%) was obtained. Brown oil. ^{1}H NMR (600 MHz, $CDCl_3$) δ 9.86 (s, 1H),

8.79 (d, *J* = 5.8 Hz, 2H), 8.16 (d, *J* = 8.2 Hz, 1H), 7.56 – 7.47 (m, 2H), 7.45 (dd, *J* = 8.4, 4.2 Hz, 1H), 3.69 (td, *J* = 8.2, 6.1, 2.9 Hz, 2H), 3.65 (s, 3H), 3.65 – 3.59 (m, 2H), 2.70 (dt, *J* = 14.8, 7.5 Hz, 1H), 2.61 (d, *J* = 8.6 Hz, 2H), 2.57 (dd, *J* = 14.1, 6.7 Hz, 1H), 2.47 – 2.39 (m, 3H), 2.30 (t, *J* = 7.5 Hz, 2H), 1.93 – 1.82 (m, 2H), 1.68 – 1.55 (m, 3H), 1.44 – 1.34 (m, 1H), 1.33 – 1.26 (m, 1H), 1.25 – 1.14 (m, 1H). ^{13}C NMR (100 MHz, $CDCl_3$) δ 174.2, 172.3, 148.1, 138.4, 136.5, 134.8, 128.1, 127.6, 121.7, 121.4, 116.5, 77.5, 77.2, 76.8, 67.6, 63.5, 51.6, 48.7, 35.6, 34.1, 28.2, 27.0, 26.4, 25.3. HRMS (ESI$^+$) m/z calcd. For $[C_{23}H_{32}N_3O_4]^+$: 414.2387, found : 414.2388.

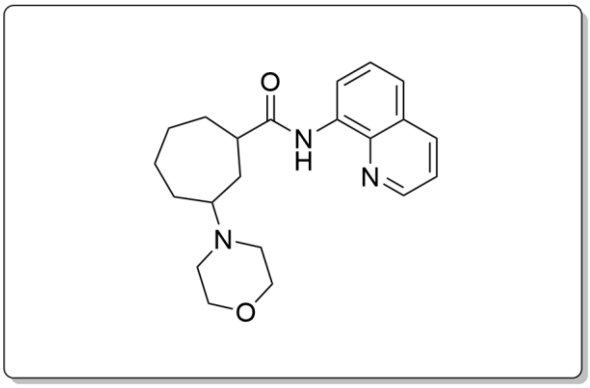

3-Morpholino-*N*-(quinolin-8-yl)cycloheptane-1-carboxamide (3i)

Prepared according to modified GP3 (Reaction was conducted at 60 °C with 2.5 equiv of morpholino benzoate) . Monitored by TLC using EA:Hx = 1:1 (1% TEA) (Rf = 0.3) as the mobile phase and purified with flash column chromatography on silica gel (EA:Hx = 1:1 (1% TEA)). From **1i** (26.6 mg, 0.1 mmol), compound **3i** (22.1 mg, 63%) was obtained. Yellow solid. ^{1}H NMR (400 MHz, CD_2Cl_2) δ 9.81 (s, 1H), 8.83 (dd, *J* = 4.2, 1.7 Hz, 1H), 8.73 (dd, *J* = 5.8, 3.2 Hz, 1H), 8.20 (dd, *J* = 8.3, 1.7 Hz, 1H), 7.56 – 7.51 (m, 2H), 7.48 (dd, *J* = 8.3, 4.2 Hz, 1H), 3.72 – 3.52 (m, 4H), 2.63 (ddt, *J* = 8.8, 4.6, 2.0 Hz, 1H), 2.58 (ddd, *J* = 6.7, 4.1, 2.3 Hz, 1H), 2.56 – 2.52 (m, 4H), 2.19 (dtt, *J* = 13.4, 2.7, 1.4 Hz, 1H), 2.09 – 1.99 (m, 1H), 1.94 – 1.88 (m, 1H), 1.87 – 1.84 (m, 1H), 1.84 – 1.81 (m, 1H), 1.81 – 1.76 (m, 1H), 1.77 – 1.67 (m, 1H), 1.67 – 1.62 (m, 1H), 1.62 – 1.58 (m, 1H), 1.58 – 1.51 (m, 1H). ^{13}C NMR (100 MHz, CD_2Cl_2) δ 175.9, 148.8, 139.0, 136.9, 135.4, 128.6, 127.8, 122.3, 121.8, 116.6, 68.0, 64.7, 54.5, 54.3, 54.0, 53.7, 53.5, 49.5, 47.5, 34.7, 32.5, 30.2, 26.1, 25.3.HRMS (EI$^+$) m/z calcd. For $[C_{21}H_{27}N_3O_2]^+$: 353.2103, found : 353.2106.

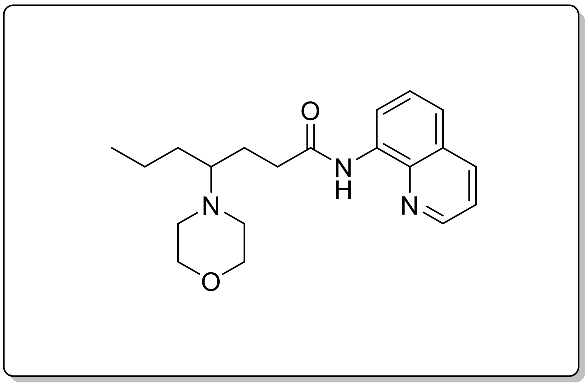

4-Morpholino-*N*-(quinolin-8-yl)heptanamide (3j).

Prepared according to GP4. Monitored by TLC using EA:Hx = 1:2 (Rf = 0.2) as the mobile phase and purified with flash column chromatography on silica gel (EA:Hx = 1:2 to MeOH:EA:Hx = 1:9:20). From **1j** (25.4 mg, 0.1 mmol), compound **3j** (22.1 mg, 65%) was obtained. Colorless oil. ^{1}H NMR (600 MHz, $CDCl_3$) δ 9.87 (s, 1H), 8.90 – 8.67 (m, 2H), 8.16 (dd, *J* = 8.2, 1.6 Hz, 1H), 7.53 (t, *J* = 7.9 Hz, 1H), 7.49 (d, *J* = 8.1 Hz, 1H), 7.45 (dd, *J* = 8.3, 4.2 Hz, 1H), 3.70 (ddd, *J* = 9.7, 6.3, 2.8 Hz, 2H), 3.62 (ddd, *J* = 10.4, 6.4, 2.7 Hz, 2H), 2.71 (dt, *J* = 14.8, 7.5 Hz, 1H), 2.63 (ddd, *J* = 9.9, 6.4, 2.7 Hz, 2H), 2.58 (dt, *J* = 13.9, 6.5 Hz, 1H), 2.49 – 2.39 (m, 3H), 1.95 – 1.89 (m, 1H), 1.88 – 1.81 (m, 1H), 1.62 – 1.54 (m, 1H), 1.45 – 1.35 (m, 1H), 1.33 – 1.26 (m, 1H), 1.23 – 1.13 (m, 1H), 0.91 (t, *J* = 7.3 Hz, 3H). ^{13}C NMR (150 MHz, $CDCl_3$) δ 172.5, 148.1, 138.5, 136.5, 134.8, 128.1, 127.6, 121.7, 121.4, 116.6, 67.7, 63.5, 48.7, 35.7, 30.7, 26.5, 20.7, 14.5. HRMS (EI^+) m/z calcd. For $[C_{20}H_{27}N_3O_2]^+$: 341.2103, found : 341.2105.

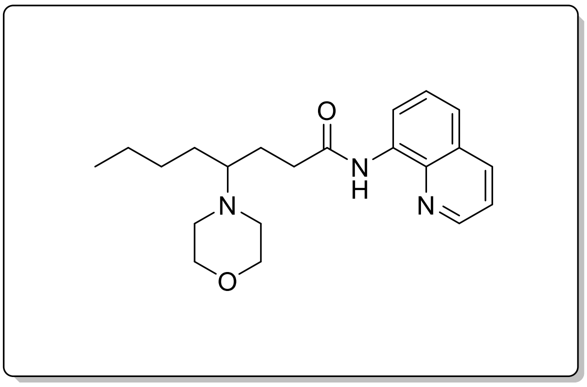

4-Morpholino-*N*-(quinolin-8-yl)octanamide (3k).

Prepared according to GP4. Monitored by TLC using EA:Hx = 1:2 (Rf = 0.2) as the mobile phase and purified with flash column chromatography on silica gel (EA:Hx = 1:2 to MeOH:EA:Hx = 1:9:20). From **1k** (26.8 mg, 0.1 mmol), compound **3k** (16.2 mg, 46%) was obtained. Colorless oil. ^{1}H NMR (400 MHz, $CDCl_3$) δ 9.87 (s, 1H), 8.92 – 8.74 (m, 2H), 8.16 (dd, *J* = 8.3, 1.7 Hz, 1H), 7.58 – 7.50 (m, 1H), 7.49 (dd, *J* = 8.3, 1.6 Hz, 1H), 7.45 (dd, *J* = 8.3, 4.2 Hz, 1H), 3.70 (ddd, *J* = 10.8, 6.3, 2.9 Hz, 2H), 3.63 (ddd, *J* = 10.8, 6.2, 2.9 Hz, 2H), 2.76 – 2.53 (m, 4H), 2.49 – 2.36 (m, 3H), 2.00 – 1.76 (m, 2H),

1.69 – 1.51 (m, 1H), 1.42 – 1.10 (m, 5H), 0.89 (t, *J* = 7.0 Hz, 3H). ^{13}C NMR (100 MHz, $CDCl_3$) δ 172.5, 148.1, 138.5, 136.5, 134.9, 128.1, 127.6, 121.7, 121.4, 116.6, 67.7, 63.7, 48.7, 35.7, 29.8, 28.1, 26.6, 23.1, 14.2. HRMS (EI$^+$) m/z calcd. For $[C_{21}H_{29}N_3O_2]^+$: 355.2260, found : 355.2258.

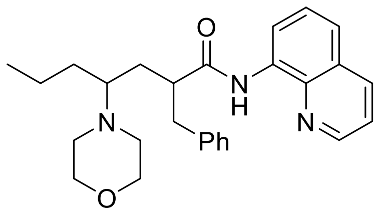

2-Benzyl-4-morpholino-*N*-(quinolin-8-yl)heptanamide (3l) (diastereomer 4.4 : 1).

Prepared according to GP4. Monitored by TLC using EA:Hx = 1:4 (Rf = 0.2) as the mobile phase and purified with flash column chromatography on silica gel (EA:Hx = 1:4) and sequentially purified with flash column chromatography on silica gel (Acetone:Hx = 1:5). From **1l** (34.4 mg, 0.1 mmol), compound **3l** (18.1 mg, 42%) was obtained. Colorless oil. ^{1}H NMR (400 MHz, $CDCl_3$) δ 9.65 (s, 1H), 8.76 (dd, *J* = 7.6, 1.5 Hz, 1H), 8.73 (dd, *J* = 4.2, 1.7 Hz, 1H), 8.13 (dd, *J* = 8.3, 1.7 Hz, 1H), 7.52 (t, *J* = 7.9 Hz, 1H), 7.46 (dd, *J* = 8.3, 1.5 Hz, 1H), 7.42 (dd, *J* = 8.3, 4.2 Hz, 1H), 7.28 – 7.19 (m, 4H), 7.14 – 7.08 (m, 1H), 3.47 – 3.32 (m, 4H), 3.19 – 3.05 (m, 1H), 2.91 – 2.78 (m, 2H), 2.60 (ddd, *J* = 11.4, 6.0, 3.3 Hz, 2H), 2.42 – 2.24 (m, 3H), 2.14 – 1.91 (m, 1H), 1.63 – 1.55 (m, 1H), 1.55 – 1.43 (m, 1H), 1.39 – 1.23 (m, 1H), 1.23 – 1.04 (m, 2H), 0.86 (t, *J* = 7.1 Hz, 3H). ^{13}C NMR (100 MHz, $CDCl_3$) δ 174.6, 148.1, 139.9, 138.4, 136.4, 134.9, 129.2, 128.5, 128.1, 127.6, 126.3, 121.6, 121.3, 116.5, 67.3, 64.2, 49.9, 48.7, 39.9, 33.4, 30.5, 20.8, 14.4. HRMS (EI$^+$) m/z calcd. For $[C_{27}H_{33}N_3O_2]^+$: 431.2573, found : 431.2577.

2-Benzyl-4-morpholino-*N*-(quinolin-8-yl)heptanamide (3l’) (diastereomer 4.4 : 1).

Prepared according to GP4. Monitored by TLC using EA:Hx = 1:4 (Rf = 0.5) as the mobile phase and purified with flash column chromatography on silica gel (EA:Hx = 1:4). From **1l** (34.4 mg, 0.1 mmol), compound **3l’** (4.2 mg, 10%) was obtained. Colorless oil. ^{1}H NMR (400 MHz, $CDCl_3$) δ 9.97 (s, 1H), 8.87 (dd, *J* = 7.4, 1.6 Hz, 1H), 8.75 (dd, *J* = 4.2, 1.7 Hz, 1H), 8.15 (dd, *J* = 8.3, 1.7 Hz, 1H), 7.58 – 7.50 (m, 1H), 7.49 (dd, *J* = 8.3, 1.6 Hz, 1H), 7.44 (dd, *J* = 8.2, 4.2 Hz, 1H), 7.30 – 7.19 (m, 4H), 7.22 – 7.11 (m, 1H), 3.89 (ddd, *J* = 10.7, 6.2, 2.8 Hz, 2H), 3.68 (ddd, *J* = 10.7, 6.4, 2.8 Hz, 2H), 3.32 – 3.17 (m, 2H), 2.83 – 2.72 (m, 1H), 2.54 – 2.35 (m, 3H), 2.34 – 2.25 (m, 2H), 1.93 – 1.81 (m, 1H), 1.57 – 1.45 (m, 2H), 1.39 – 1.24 (m, 1H), 1.18 – 1.05 (m, 1H), 1.07 – 0.92 (m, 1H), 0.82 (t, *J* = 7.2 Hz, 3H). ^{13}C NMR (100 MHz, $CDCl_3$) δ 174.8, 148.0, 140.2, 138.5, 136.5, 134.8, 129.2, 128.5, 128.1, 127.6, 126.3,

121.7, 121.5, 116.8, 67.8, 61.2, 48.2, 46.5, 38.8, 34.3, 29.9, 20.6, 14.5. HRMS (EI$^+$) m/z calcd. For $[C_{27}H_{33}N_3O_2]^+$: 431.2573, found : 431.2572.

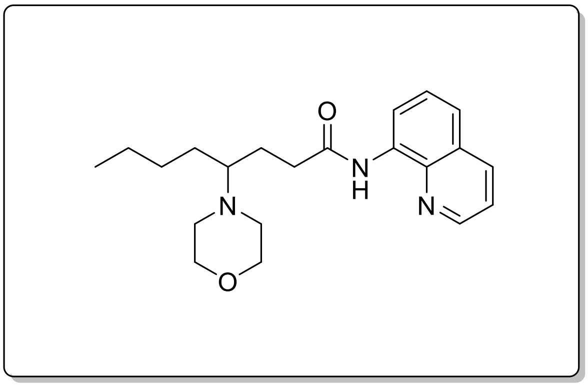

4-Morpholino-*N*-(quinolin-8-yl)octanamide (3m).

Prepared according to GP4. Monitored by TLC using EA:Hx = 1:2 (Rf = 0.2) as the mobile phase and purified with flash column chromatography on silica gel (EA:Hx = 1:2 to MeOH:EA:Hx = 1:9:20). From **1m** (26.8 mg, 0.1 mmol), compound **3m** (17.8 mg, 50%) was obtained. Colorless oil. ^{1}H NMR (400 MHz, $CDCl_3$) δ 9.87 (s, 1H), 9.09 – 8.49 (m, 2H), 8.16 (dd, *J* = 8.2, 1.7 Hz, 1H), 7.58 – 7.50 (m, 1H), 7.49 (dd, *J* = 8.3, 1.6 Hz, 1H), 7.45 (dd, *J* = 8.3, 4.2 Hz, 1H), 3.70 (ddd, *J* = 10.8, 6.3, 2.9 Hz, 2H), 3.63 (ddd, *J* = 10.8, 6.3, 2.9 Hz, 2H), 2.78 – 2.52 (m, 4H), 2.49 – 2.37 (m, 3H), 2.01 – 1.78 (m, 2H), 1.69 – 1.54 (m, 1H), 1.44 – 1.06 (m, 5H), 0.89 (t, *J* = 7.0 Hz, 2H). ^{13}C NMR (100 MHz, $CDCl_3$) δ 172.5, 148.1, 138.5, 136.5, 134.9, 128.1, 127.6, 121.7, 121.4, 116.6, 67.7, 63.7, 48.7, 35.7, 29.8, 28.1, 26.6, 23.2, 14.2. HRMS (EI$^+$) m/z calcd. For $[C_{21}H_{29}N_3O_2]^+$: 355.2260, found : 355.2257.

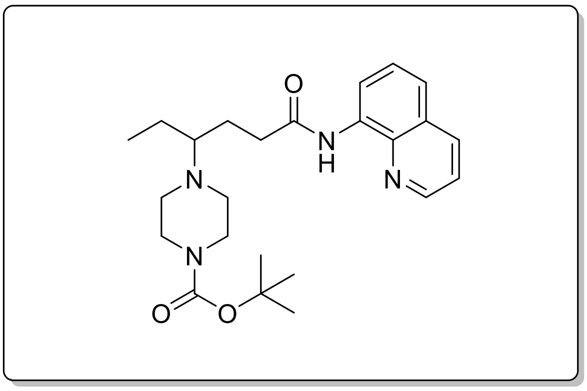

tert-Butyl 4-(6-oxo-6-(quinolin-8-ylamino)hexan-3-yl)piperazine-1-carboxylate (4a).

Prepared according to GP3. Monitored by TLC using EA:Hx = 1:2 (Rf = 0.3) as the mobile phase and purified with flash column chromatography on silica gel (EA:Hx = 1:2). From **1a** (24.0 mg, 0.1 mmol), compound **4a** (29.1 mg, 68%) was obtained. Colorless oil. ^{1}H NMR (400 MHz, CD_2Cl_2) δ 9.84 (s, 1H), 8.81 (dd, *J* = 4.3, 1.7 Hz, 1H), 8.76 (dd, *J* = 6.4, 2.6 Hz, 1H), 8.19 (dd, *J* = 8.3, 1.7 Hz, 1H), 7.57 – 7.50 (m, 2H), 7.48 (dd, *J* = 8.3, 4.2 Hz, 1H), 3.40 (ddd, *J* = 12.5, 6.6, 3.3 Hz, 2H), 3.32 (ddd, *J* = 12.2, 6.5,

3.2 Hz, 2H), 2.68 (ddd, J = 15.0, 8.1, 6.9 Hz, 1H), 2.63 – 2.50 (m, 3H), 2.47 – 2.29 (m, 3H), 1.98 – 1.86 (m, 1H), 1.86 – 1.75 (m, 1H), 1.69 – 1.56 (m, 1H), 1.42 (s, 9H), 1.25 – 1.14 (m, 1H), 0.91 (t, J = 7.5 Hz, 3H). ^{13}C NMR (100 MHz, CD_2Cl_2) δ 172.7, 155.1, 148.7, 138.9, 136.9, 135.5, 128.6, 127.8, 122.2, 121.7, 116.6, 79.5, 65.8, 48.6, 44.8, 36.0, 28.7, 26.5, 21.7, 12.3. HRMS (EI^+) m/z calcd. For $[C_{24}H_{34}N_4O_3]^+$: 426.2631, found : 426.2633.

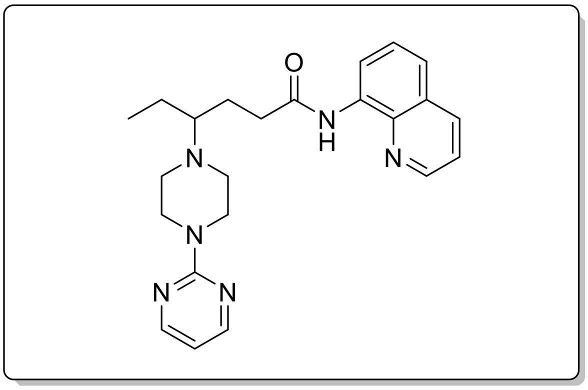

4-(4-(Pyrimidin-2-yl)piperazin-1-yl)-*N*-(quinolin-8-yl)hexanamide (4b).

Prepared according to GP3. Monitored by TLC using EA:Hx = 1:1 (Rf = 0.2) as the mobile phase and purified with flash column chromatography on silica gel (EA:Hx = 1:1). From **1a** (24.0 mg, 0.1 mmol), compound **4b** (31.4 mg, 78%) was obtained. Pale yellow oil. ^{1}H NMR (400 MHz, $CDCl_3$) δ 9.90 (s, 1H), 8.84 – 8.76 (m, 2H), 8.27 (d, J = 4.7 Hz, 2H), 8.14 (dd, J = 8.3, 1.7 Hz, 1H), 7.52 (t, J = 7.9 Hz, 1H), 7.47 (dd, J = 8.3, 1.6 Hz, 1H), 7.43 (dd, J = 8.3, 4.2 Hz, 1H), 6.43 (t, J = 4.7 Hz, 1H), 3.86 (ddd, J = 12.7, 6.8, 3.3 Hz, 2H), 3.76 (ddd, J = 12.7, 6.8, 3.2 Hz, 2H), 2.82 – 2.68 (m, 3H), 2.62 (dt, J = 14.3, 6.4 Hz, 1H), 2.55 – 2.42 (m, 3H), 2.06 – 1.92 (m, 1H), 1.91 – 1.79 (m, 1H), 1.72 – 1.58 (m, 1H), 1.26 – 1.13 (m, 1H), 0.91 (t, J = 7.4 Hz, 3H). ^{13}C NMR (100 MHz, $CDCl_3$) δ 172.6, 161.8, 157.8, 148.2, 138.5, 136.5, 134.8, 128.1, 127.6, 121.7, 121.4, 116.6, 109.6, 65.2, 48.1, 44.4, 35.7, 26.3, 21.2, 12.1. HRMS (EI^+) m/z calcd. For $[C_{23}H_{28}N_6O]^+$: 404.2325, found : 404.2325.

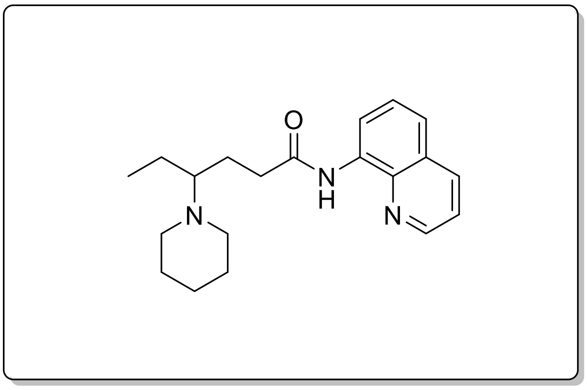

4-(Piperidin-1-yl)-*N*-(quinolin-8-yl)hexanamide (4c).

Prepared according to GP3. Monitored by TLC using MeOH:EA:Hx = 1:9:10 (Rf = 0.2) as the mobile

phase and purified with flash column chromatography on silica gel (EA:Hx = 1:1 to EA:Hx = 1:1 (1% TEA)). From **1a** (24.0 mg, 0.1 mmol), compound **4c** (24.1 mg, 74%) was obtained. Yellowish oil. ^{1}H NMR (400 MHz, $CDCl_3$) δ 9.88 (s, 1H), 8.86 – 8.73 (m, 2H), 8.15 (dd, J = 8.2, 1.7 Hz, 1H), 7.56 – 7.50 (m, 1H), 7.48 (dd, J = 8.2, 1.5 Hz, 1H), 7.44 (dd, J = 8.3, 4.2 Hz, 1H), 2.69 (dt, J = 14.2, 7.9 Hz, 1H), 2.64 – 2.53 (m, 3H), 2.43 – 2.28 (m, 3H), 1.99 – 1.87 (m, 1H), 1.86 – 1.75 (m, 1H), 1.73 – 1.65 (m, 1H), 1.65 – 1.56 (m, 2H), 1.55 – 1.45 (m, 2H), 1.44 – 1.33 (m, 2H), 1.25 – 1.13 (m, 1H), 0.89 (t, J = 7.5 Hz, 3H). ^{13}C NMR (100 MHz, $CDCl_3$) δ 173.0, 148.1, 138.6, 136.4, 135.0, 128.1, 127.6, 121.6, 121.3, 116.6, 65.6, 49.5, 35.8, 26.9, 26.5, 25.3, 21.3, 12.2. HRMS (EI$^+$) m/z calcd. For $[C_{20}H_{27}N_3O]^+$: 325.2154, found : 325.2156.

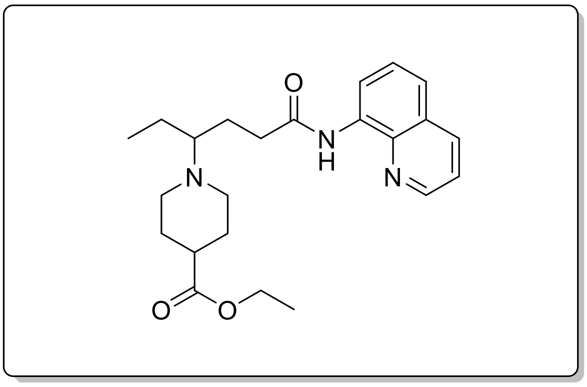

Ethyl 1-(6-oxo-6-(quinolin-8-ylamino)hexan-3-yl)piperidine-4-carboxylate (4d).

Prepared according to GP3. Monitored by TLC using EA:Hx = 1:1 (Rf = 0.3) as the mobile phase and purified with flash column chromatography on silica gel (EA:Hx = 1:1 to MeOH:EA:Hx = 1:9:10). From **1a** (24.0 mg, 0.1 mmol), compound **4d** (28.9 mg, 73%) was obtained. Pale yellow oil. ^{1}H NMR (400 MHz, $CDCl_3$) δ 9.88 (s, 1H), 8.95 – 8.56 (m, 2H), 8.14 (dd, J = 8.3, 1.7 Hz, 1H), 7.57 – 7.48 (m, 1H), 7.47 (dd, J = 8.2, 1.6 Hz, 1H), 7.44 (dd, J = 8.3, 4.2 Hz, 1H), 4.10 (q, J = 7.1 Hz, 2H), 2.86 – 2.76 (m, 1H), 2.74 – 2.62 (m, 2H), 2.62 – 2.51 (m, 1H), 2.53 – 2.43 (m, 1H), 2.46 – 2.34 (m, 1H), 2.28 – 2.10 (m, 2H), 2.01 – 1.73 (m, 5H), 1.76 – 1.56 (m, 2H), 1.27 – 1.11 (m, 4H), 0.89 (t, J = 7.4 Hz, 3H). ^{13}C NMR (100 MHz, $CDCl_3$) δ 175.6, 172.7, 148.2, 138.5, 136.4, 134.9, 128.1, 127.5, 121.7, 121.3, 116.5, 65.1, 60.3, 50.4, 45.6, 42.0, 35.6, 29.1, 29.0, 26.5, 21.3, 14.4, 12.1. HRMS (EI$^+$) m/z calcd. For $[C_{23}H_{31}N_3O_3]^+$: 397.2365, found : 397.2361.

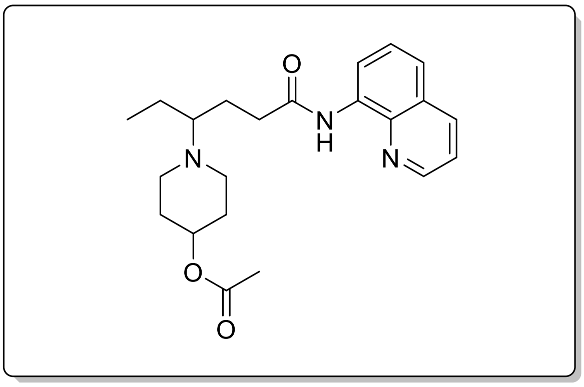

1-(6-Oxo-6-(quinolin-8-ylamino)hexan-3-yl)piperidin-4-yl acetate (4e).

Prepared according to GP3. Monitored by TLC using MeOH:EA:Hx = 1:9:10 (Rf = 0.5) as the mobile phase and purified with flash column chromatography on silica gel (EA:Hx = 1:1 to MeOH:EA:Hx = 1:9:10). From **1a** (24.0 mg, 0.1 mmol), compound **4e** (27.5 mg, 72%) was obtained. Yellowish oil. ^{1}H NMR (400 MHz, $CDCl_3$) δ 9.86 (s, 1H), 8.84 – 8.67 (m, 2H), 8.15 (dd, *J* = 8.2, 1.7 Hz, 1H), 7.57 – 7.51 (m, 1H), 7.48 (dd, *J* = 8.3, 1.6 Hz, 1H), 7.44 (dd, *J* = 8.3, 4.2 Hz, 1H), 4.71 (tt, *J* = 8.6, 4.2 Hz, 1H), 2.78 (dddd, *J* = 11.3, 5.6, 3.7, 1.4 Hz, 1H), 2.72 – 2.51 (m, 4H), 2.41 (dq, *J* = 13.5, 4.8 Hz, 1H), 2.29 (ddd, *J* = 11.8, 9.2, 3.2 Hz, 1H), 2.01 (s, 3H), 1.99 – 1.75 (m, 4H), 1.75 – 1.52 (m, 3H), 1.24 – 1.14 (m, 1H), 0.90 (t, *J* = 7.4 Hz, 3H). ^{13}C NMR (100 MHz, $CDCl_3$) δ 172.7, 170.7, 148.1, 138.5, 136.5, 134.9, 128.1, 127.6, 121.7, 121.4, 116.6, 71.3, 65.1, 47.0, 44.5, 35.7, 31.7, 31.5, 26.6, 21.5, 21.4, 12.1. HRMS (EI^+) m/z calcd. For $[C_{22}H_{29}N_3O_3]^+$: 383.2209, found : 383.2209.

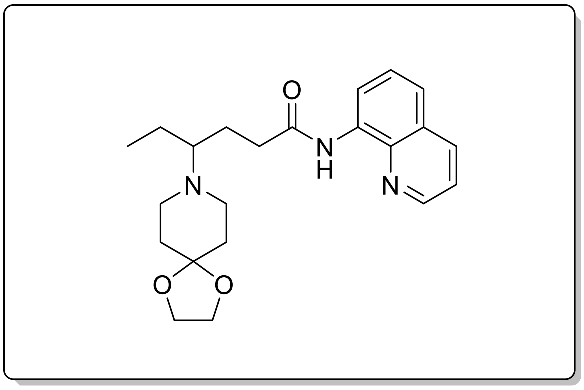

***N*-(Quinolin-8-yl)-4-(1,4-dioxa-8-azaspiro[4.5]decan-8-yl)hexanamide (4f).**

Prepared according to GP4. Monitored by TLC using EA:Hx = 1:1 (1% TEA) (Rf = 0.3) as the mobile phase and purified with flash column chromatography on silica gel (EA:Hx = 1:1 to EA:Hx = 1:1 (1% TEA)). From **1a** (24.0 mg, 0.1 mmol), compound **4f** (21.6 mg, 56%) was obtained. Pale yellow oil. ^{1}H NMR (400 MHz, $CDCl_3$) δ 9.88 (s, 1H), 8.90 – 8.45 (m, 2H), 8.15 (dd, *J* = 8.3, 1.7 Hz, 1H), 7.56 – 7.51 (m, 1H), 7.48 (dd, *J* = 8.3, 1.6 Hz, 1H), 7.44 (dd, *J* = 8.3, 4.2 Hz, 1H), 3.94 (s, 4H), 2.78 – 2.65 (m, 3H), 2.60 (ddd, *J* = 14.2, 7.1, 5.6 Hz, 1H), 2.54 – 2.39 (m, 3H), 2.03 – 1.90 (m, 1H), 1.86 – 1.74 (m, 3H), 1.74 – 1.58 (m, 3H), 1.28 – 1.13 (m, 1H), 0.90 (t, *J* = 7.4 Hz, 3H). ^{13}C NMR (100 MHz, $CDCl_3$) δ 172.7,

148.2, 138.5, 136.4, 134.9, 128.1, 127.6, 121.7, 121.4, 116.6, 108.0, 64.7, 64.3, 46.1, 35.7, 35.7, 26.7, 21.5, 12.1. HRMS (EI+) m/z calcd. For $[C_{22}H_{29}N_3O_3]^+$: 383.2209, found : 383.2206.

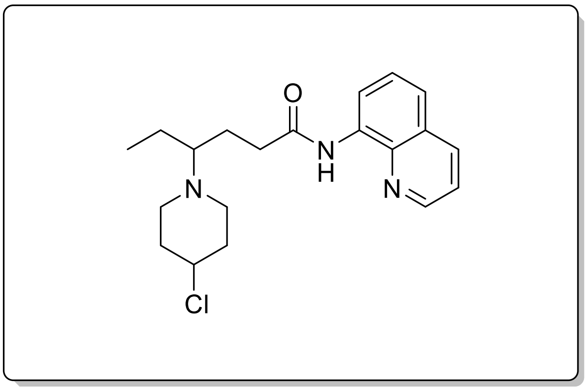

4-(4-Chloropiperidin-1-yl)-*N*-(quinolin-8-yl)hexanamide (4g).

Prepared according to GP3. Monitored by TLC using EA:Hx = 1:2 (Rf = 0.2) as the mobile phase and purified with flash column chromatography on silica gel (EA:Hx = 1:2 to EA:Hx = 1:1). From **1a** (24.0 mg, 0.1 mmol), compound **4g** (26.3 mg, 73%) was obtained. Yellowish oil. ^{1}H NMR (400 MHz, $CDCl_3$) δ 9.87 (s, 1H), 8.88 – 8.69 (m, 2H), 8.15 (dd, *J* = 8.3, 1.7 Hz, 1H), 7.57 – 7.51 (m, 1H), 7.49 (dd, *J* = 8.3, 1.6 Hz, 1H), 7.45 (dd, *J* = 8.3, 4.2 Hz, 1H), 3.95 (dp, *J* = 8.9, 4.0 Hz, 1H), 2.86 (dq, *J* = 10.7, 3.8 Hz, 1H), 2.68 (ddd, *J* = 14.8, 8.5, 6.7 Hz, 2H), 2.60 – 2.46 (m, 2H), 2.41 (tt, *J* = 9.4, 4.8 Hz, 1H), 2.26 (ddd, *J* = 12.0, 9.1, 3.0 Hz, 1H), 2.14 – 2.05 (m, 1H), 2.04 – 1.88 (m, 2H), 1.87 – 1.73 (m, 2H), 1.70 – 1.56 (m, 1H), 1.24 – 1.13 (m, 1H), 0.90 (t, *J* = 7.4 Hz, 3H). ^{13}C NMR (100 MHz, $CDCl_3$) δ 172.6, 148.2, 138.5, 136.5, 134.8, 128.1, 127.6, 121.7, 121.4, 116.6, 65.1, 58.6, 47.7, 45.5, 36.5, 36.4, 35.7, 26.5, 21.5, 12.1. HRMS (EI+) m/z calcd. For $[C_{20}H_{26}ClN_3O]^+$: 359.1764, found : 359.1761.

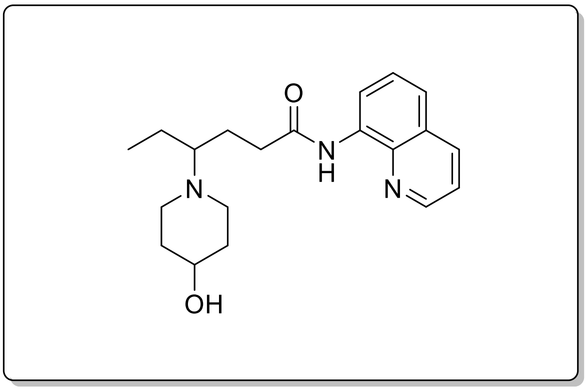

4-(4-Hydroxypiperidin-1-yl)-*N*-(quinolin-8-yl)hexanamide (4h).

Prepared according to GP3. Monitored by TLC using EA:Hx = 4:1 (1% TEA) (Rf = 0.1) as the mobile phase and purified with flash column chromatography on silica gel (EA:Hx = 4:1 to EA:Hx = 4:1 (1% TEA)). From **1a** (24.0 mg, 0.1 mmol), compound **4h** (19.8 mg, 58%) was obtained. Colorless oil. ^{1}H NMR (400 MHz, $CDCl_3$) δ 9.87 (s, 1H), 9.02 – 8.64 (m, 2H), 8.16 (d, *J* = 8.2 Hz, 1H), 7.57 – 7.51 (m,

1H), 7.51 – 7.47 (m, 1H), 7.45 (dd, *J* = 8.2, 4.2 Hz, 1H), 3.75 – 3.44 (m, 1H), 2.88 – 2.74 (m, 1H), 2.76 – 2.35 (m, 5H), 2.31 – 2.15 (m, 1H), 2.01 – 1.73 (m, 4H), 1.73 – 1.42 (m, 4H), 1.31 – 1.13 (m, 1H), 0.91 (t, *J* = 7.4 Hz, 3H). ^{13}C NMR (100 MHz, $CDCl_3$) δ 172.7, 148.2, 138.5, 136.5, 134.9, 128.1, 127.6, 121.7, 121.4, 116.6, 69.1, 65.0, 47.8, 44.3, 35.8, 35.5, 35.4, 26.6, 21.5, 12.2. HRMS (ESI$^+$) m/z calcd. For $[C_{20}H_{28}N_3O_2]^+$: 342.2176, found : 342.2173.

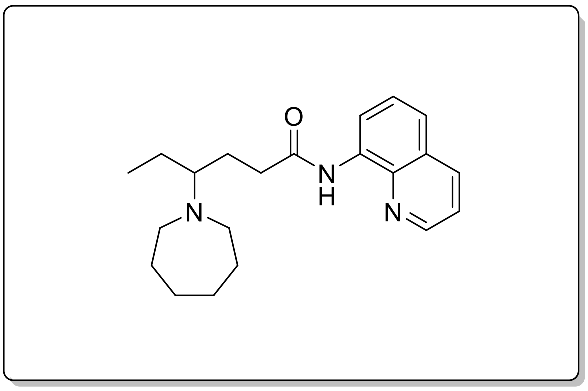

4-(Azepan-1-yl)-*N*-(quinolin-8-yl)hexanamide (4i).

Prepared according to GP3. Monitored by TLC using EA:Hx = 1:1 (1% TEA) (Rf = 0.3) as the mobile phase and purified with flash column chromatography on silica gel (EA:Hx = 1:1 to EA:Hx = 1:1 (TEA 1%). From **1a** (24.0 mg, 0.1 mmol), compound **4i** (24.7 mg, 73%) was obtained. Yellowish oil. ^{1}H NMR (400 MHz, $CDCl_3$) δ 9.85 (s, 1H), 8.90 – 8.57 (m, 2H), 8.15 (dd, *J* = 8.3, 1.7 Hz, 1H), 7.53 (t, *J* = 7.9 Hz, 1H), 7.48 (dd, *J* = 8.3, 1.6 Hz, 1H), 7.44 (dd, *J* = 8.3, 4.2 Hz, 1H), 2.81 – 2.62 (m, 4H), 2.62 – 2.50 (m, 2H), 2.51 – 2.37 (m, 1H), 1.97 – 1.85 (m, 1H), 1.82 – 1.68 (m, 1H), 1.68 – 1.49 (m, 9H), 1.35 – 1.14 (m, 1H), 0.91 (t, *J* = 7.4 Hz, 3H). ^{13}C NMR (100 MHz, $CDCl_3$) δ 172.8, 148.1, 138.5, 136.4, 134.9, 128.1, 127.6, 121.7, 121.3, 116.5, 66.7, 51.3, 35.9, 30.3, 27.7, 27.2, 22.9, 12.3. HRMS (EI$^+$) m/z calcd. For $[C_{21}H_{29}N_3O]^+$: 339.2311, found : 339.2313.

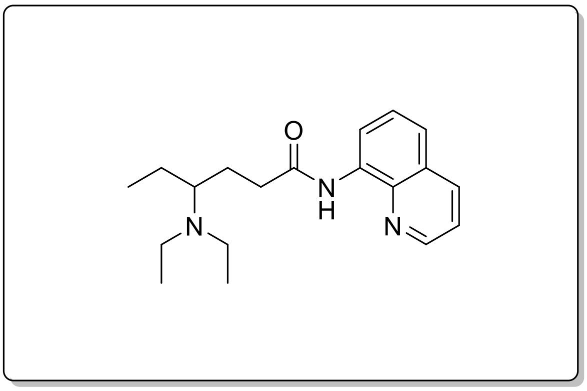

4-(Diethylamino)-*N*-(quinolin-8-yl)hexanamide (4j).

Prepared according to GP3. Monitored by TLC using MeOH:EA:Hx = 1:9:10 (Rf = 0.3) as the mobile phase and purified with flash column chromatography on silica gel (EA:Hx = 1:1 to EA:Hx = 1:1 (TEA

1%). From **1a** (24.0 mg, 0.1 mmol), compound **4j** (20.4 mg, 65%) was obtained. Brown oil. ^{1}H NMR (400 MHz, $CDCl_3$) δ 9.85 (s, 1H), 8.87 – 8.63 (m, 2H), 8.15 (dd, *J* = 8.3, 1.7 Hz, 1H), 7.56 – 7.50 (m, 1H), 7.48 (dd, *J* = 8.2, 1.6 Hz, 1H), 7.44 (dd, *J* = 8.3, 4.2 Hz, 1H), 2.70 (ddd, *J* = 14.5, 8.5, 5.8 Hz, 1H), 2.65 – 2.47 (m, 4H), 2.46 – 2.31 (m, 2H), 2.01 – 1.86 (m, 1H), 1.83 – 1.72 (m, 1H), 1.65 – 1.51 (m, 1H), 1.24 – 1.13 (m, 1H), 1.00 (t, *J* = 7.1 Hz, 6H), 0.92 (t, *J* = 7.4 Hz, 3H). ^{13}C NMR (100 MHz, $CDCl_3$) δ 172.8, 148.2, 138.5, 136.4, 134.9, 128.1, 127.6, 121.7, 121.3, 116.5, 60.9, 43.4, 35.9, 26.6, 22.2, 15.0, 12.3. HRMS (EI$^+$) m/z calcd. For $[C_{19}H_{27}N_3O]^+$: 313.2154, found : 313.2153.

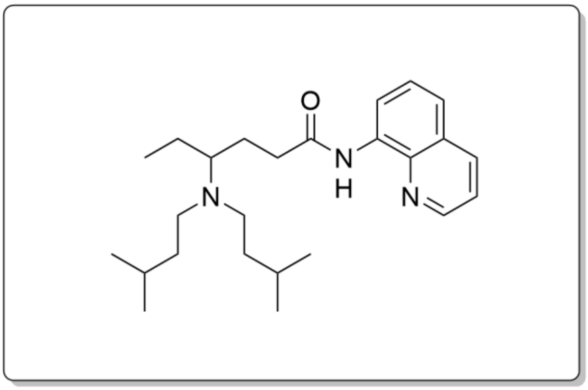

4-(diisopentylamino)-N-(quinolin-8-yl)hexanamide (4k).

Prepared according to GP3. Monitored by TLC using EA:Hx = 1:5 (Rf = 0.3) as the mobile phase and purified with flash column chromatography on silica gel (EA:Hx = 1:5 to EA:Hx=1:1). From **1a** (24.0 mg, 0.1 mmol), compound **4k** (19.1 mg, 48%) was obtained. Yellowish oil. ^{1}H NMR (400 MHz, $CDCl_3$) δ 9.84 (s, 1H), 8.80 (dt, J = 6.1, 1.6 Hz, 2H), 8.15 (dd, J = 8.2, 1.7 Hz, 1H), 7.59 – 7.40 (m, 3H), 2.70 (ddd, J = 14.6, 8.8, 5.6 Hz, 1H), 2.58 (ddd, J = 15.3, 8.8, 7.0 Hz, 1H), 2.46 (dt, J = 12.3, 7.8 Hz, 3H), 2.32 (dt, J = 12.8, 7.3 Hz, 2H), 1.98 – 1.85 (m, 1H), 1.83 – 1.69 (m, 1H), 1.66 – 1.49 (m, 4H), 1.32 – 1.17 (m, 4H), 0.93 (t, J = 7.4 Hz, 3H), 0.83 (dd, J = 6.6, 2.5 Hz, 12H). ^{13}C NMR (100 MHz, $CDCl_3$) δ 172.7, 148.2, 138.5, 136.5, 134.9, 128.1, 127.6, 121.7, 121.3, 116.5, 77.5, 77.2, 76.8, 61.5, 48.2, 38.8, 36.0, 26.6, 26.3, 23.1, 22.8, 22.1, 12.4. HRMS (EI$^+$) m/z calcd. For $[C_{25}H_{39}N_3O_1]^+$: 398.3166, found : 398.3166.

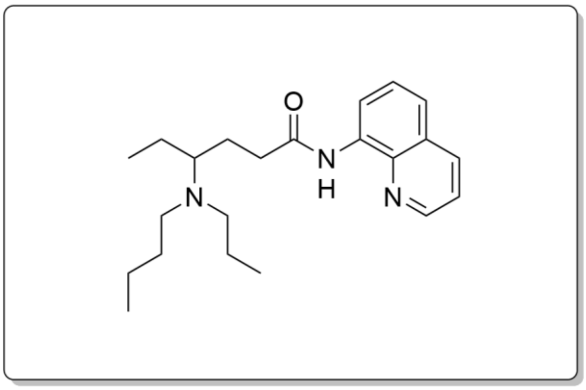

4-(butyl(propyl)amino)-N-(quinolin-8-yl)hexanamide (4l).

Prepared according to GP3. Monitored by TLC using EA:Hx = 1:5 (Rf = 0.3) as the mobile phase and purified with flash column chromatography on silica gel (EA:Hx = 1:5 to EA:Hx=1:1). From **1a** (24.0 mg, 0.1 mmol), compound **4l** (17.0 mg, 48%) was obtained. Colorless oil. ^{1}H NMR (400 MHz, $CDCl_3$) δ 9.83 (s, 1H), 8.84 – 8.75 (m, 2H), 8.15 (dd, J = 8.3, 1.7 Hz, 1H), 7.58 – 7.40 (m, 3H), 2.73 (ddd, J = 14.6, 8.9, 5.5 Hz, 1H), 2.59 (ddd, J = 15.2, 9.0, 6.9 Hz, 1H), 2.43 (dddd, J = 14.3, 8.6, 6.7, 1.9 Hz, 3H), 2.39 – 2.26 (m, 2H), 1.90 (dddd, J = 13.9, 8.9, 7.0, 5.0 Hz, 1H), 1.77 (ddt, J = 13.5, 8.8, 4.6 Hz, 1H), 1.57 (dtd, J = 12.5, 7.3, 6.4, 3.4 Hz, 2H), 1.50 – 1.09 (m, 6H), 0.92 (t, J = 7.4 Hz, 3H), 0.85 (d, J = 7.3 Hz, 6H).^{13}C NMR (100 MHz, $CDCl_3$) δ 172.8, 148.2, 138.5, 136.5, 134.9, 128.1, 127.6, 121.7, 121.3, 116.5, 77.5, 77.2, 76.8, 61.8, 52.3, 50.1, 36.0, 32.0, 26.7, 22.7, 22.2, 20.8, 14.3, 12.4, 12.1. HRMS (EI^+) m/z calcd. For $[C_{25}H_{39}N_3O_1]^+$: 356.2696, found : 356.2696.

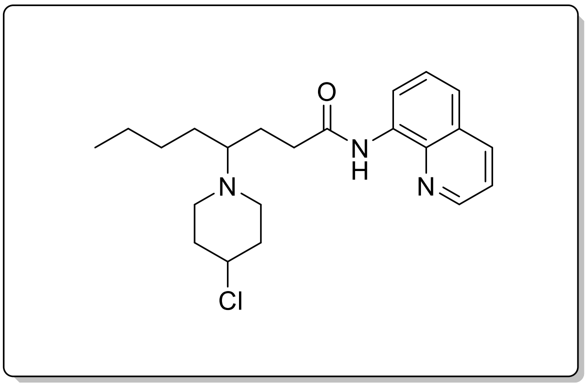

4-(4-Chloropiperidin-1-yl)-*N*-(quinolin-8-yl)octanamide (4m).

Prepared according to GP4. Monitored by TLC using EA:Hx = 1:3 (Rf = 0.25) as the mobile phase and purified with preparative TLC on silica gel (EA:Hx = 1:3). From **1f** (26.8 mg, 0.1 mmol), compound **4m** (15.7 mg, 40%) was obtained. Colorless oil. ^{1}H NMR (600 MHz, $CDCl_3$) δ 9.86 (s, 1H), 8.80 (d, J = 8.0 Hz, 2H), 8.16 (d, J = 8.1 Hz, 1H), 7.54 (t, J = 7.8 Hz, 1H), 7.49 (d, J = 8.1 Hz, 1H), 7.45 (dd, J = 7.8, 3.9 Hz, 1H), 4.09 – 3.86 (m, 1H), 2.90 – 2.79 (m, 1H), 2.72 – 2.62 (m, 2H), 2.59 – 2.42 (m, 3H), 2.32 – 2.20 (m, 1H), 2.15 – 1.88 (m, 4H), 1.87 – 1.73 (m, 2H), 1.65 – 1.48 (m, 1H), 1.37 – 1.11 (m, 5H), 0.88 (t, J = 6.2 Hz, 3H). ^{13}C NMR (100 MHz, $CDCl_3$) δ 172.6, 148.2, 138.5, 136.5, 134.8, 128.1, 127.6, 121.7, 121.4, 116.6, 63.4, 58.6, 45.5, 36.5, 36.4, 35.8, 29.8, 28.4, 27.1, 23.1, 14.2. HRMS (EI^+) m/z calcd. For $[C_{22}H_{30}ClN_3O]^+$: 387.2077, found : 387.2078.

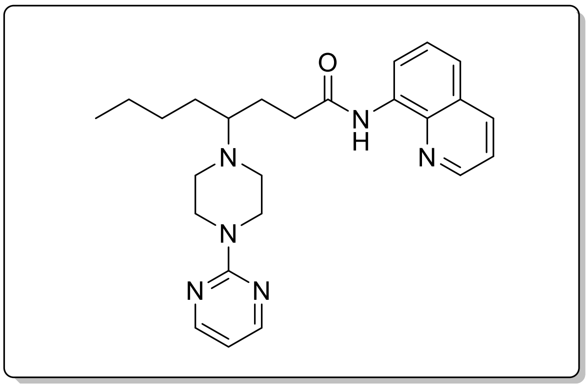

4-(4-(Pyrimidin-2-yl)piperazin-1-yl)-*N*-(quinolin-8-yl)octanamide (4n).

Prepared according to GP4. Monitored by TLC using EA:Hx = 1:2 (Rf = 0.25) as the mobile phase and purified with preparative TLC on silica gel (EA:Hx = 1:3 and EA:Hx = 1:2). From **1m** (26.8 mg, 0.1 mmol), compound **4n** (18.7 mg, 43%) was obtained. Colorless oil. ^{1}H NMR (600 MHz, $CDCl_3$) δ 9.89 (s, 1H), 8.85 – 8.74 (m, 2H), 8.28 (d, *J* = 4.5 Hz, 2H), 8.14 (d, *J* = 8.2 Hz, 1H), 7.53 (t, *J* = 7.9 Hz, 1H), 7.48 (d, *J* = 8.1 Hz, 1H), 7.43 (dd, *J* = 8.0, 4.1 Hz, 1H), 6.43 (t, *J* = 4.4 Hz, 1H), 3.91 – 3.82 (m, 2H), 3.81 – 3.70 (m, 2H), 2.81 – 2.67 (m, 3H), 2.66 – 2.59 (m, 1H), 2.58 – 2.45 (m, 3H), 2.01 – 1.92 (m, 1H), 1.92 – 1.80 (m, 1H), 1.65 – 1.54 (m, 1H), 1.40 – 1.25 (m, 3H), 1.25 – 1.13 (m, 2H), 0.87 (t, *J* = 6.8 Hz, 3H). ^{13}C NMR (100 MHz, $CDCl_3$) δ 172.6, 161.8, 157.8, 148.2, 138.5, 136.5, 134.8, 128.1, 127.6, 121.7, 121.4, 116.6, 109.6, 63.5, 48.1, 44.4, 35.7, 29.8, 28.2, 26.9, 23.1, 14.2. HRMS (EI^+) m/z calcd. For $[C_{25}H_{32}N_6O]^+$: 432.2638, found : 432.2639.

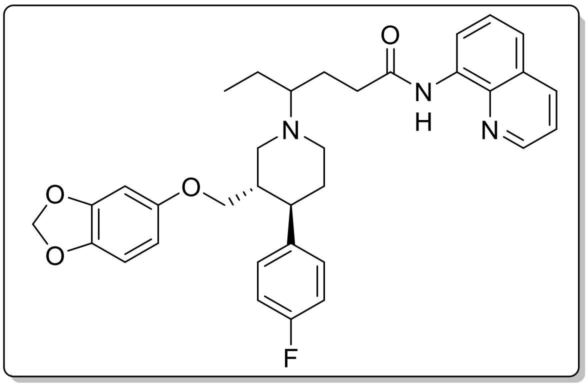

4-((3*S*,4*R*)-3-((Benzo[*d*][1,3]dioxol-5-yloxy)methyl)-4-(4-fluorophenyl)piperidin-1-yl)-*N*-(quinolin-8-yl)hexanamide (4o) (diastereomer 1.1 : 1).

Prepared according to GP3. Monitored by TLC using EA:Hx = 1:3 (Rf = 0.2) as the mobile phase and purified with flash column chromatography on alumina basic (EA:Hx = 1:15 to EA:Hx = 1:9) and sequentially purified with preparative TLC on silica gel (EA:Hx = 1:3). From **1a** (24.0 mg, 0.1 mmol), compound **4o** (35.9 mg, 66%) was obtained. Colorless oil. ^{1}H NMR (400 MHz, $CDCl_3$) δ 9.92 (s, 1H), 8.91 – 8.82 (m, 1H), 8.81 – 8.71 (m, 1H), 8.21 – 8.11 (m, 1H), 7.61 – 7.48 (m, 2H), 7.47 – 7.36 (m, 1H), 6.91 – 6.70 (m, 4H), 6.65 – 6.53 (m, 1H), 6.37 – 6.18 (m, 1H), 6.17 – 5.96 (m, 1H), 5.90 – 5.81 (m,

2H), 3.57 – 3.21 (m, 2H), 3.15 – 2.95 (m, 1H), 2.94 – 2.67 (m, 2H), 2.67 – 2.44 (m, 3H), 2.43 – 2.12 (m, 2H), 2.08 – 1.87 (m, 3H), 1.83 – 1.51 (m, 3H), 1.36 – 1.22 (m, 1H), 0.95 (t, *J* = 7.4 Hz, 3H). ^{13}C NMR (100 MHz, $CDCl_3$) δ 172.8, 172.7, 162.7 – 160.1 (m), 154.7, 154.6, 148.3, 148.2, 148.2, 141.6, 141.5, 140.4, 140.3, 140.2, 140.2, 138.5, 136.5, 136.4, 135.1, 135.1, 129.3 – 128.5 (m), 128.2, 128.2, 127.7, 127.7, 121.7, 121.3, 121.2, 116.6, 116.6, 115.6 – 114.5 (m), 107.9, 107.9, 105.8, 105.5, 101.1, 98.2, 98.0, 70.0, 69.9, 66.6, 66.1, 56.4, 52.3, 49.5, 45.6, 44.9, 44.7, 42.8, 42.5, 36.5, 36.3, 35.2, 34.6, 26.4, 26.3, 21.3, 21.0, 12.3, 12.3. ^{19}F NMR (376 MHz, $CDCl_3$) δ -117.1, -117.1. HRMS (EI$^+$) m/z calcd. For $[C_{34}H_{36}FN_3O_4]^+$: 569.2690, found : 569.2689.

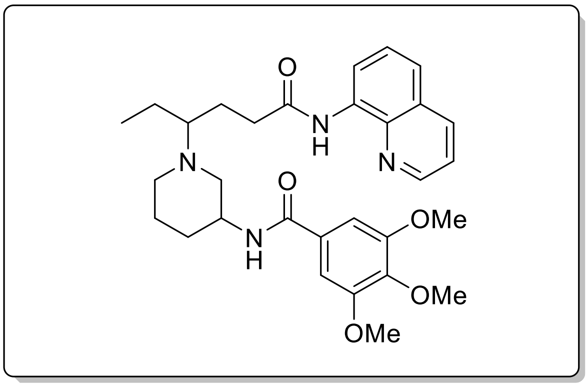

3,4,5-Trimethoxy-*N*-(1-(6-oxo-6-(quinolin-8-ylamino)hexan-3-yl)piperidin-3-yl)benzamide (4p) (diastereomer 2 : 1).

Prepared according to GP3. Monitored by TLC using MeOH:EA:Hx = 1:9:10 (Rf = 0.2) as the mobile phase and purified with flash column chromatography on silica gel (EA:Hx = 1:1 to MeOH:EA:Hx = 1:9:10) and sequentially purified with preparative reverse TLC on silica gel (MeOH:H_2O). From **1a** (24.0 mg, 0.1 mmol), compound **4p** (27.4 mg, 51%) was obtained. Yellowish oil. ^{1}H NMR (400 MHz, $CDCl_3$) δ 10.06 – 9.75 (m, 1H), 8.88 – 8.79 (m, 1H), 8.78 – 8.69 (m, 1H), 8.29 – 8.14 (m, 1H), 7.58 – 7.43 (m, 3H), 7.38 – 7.12 (m, 2H), 4.49 – 4.20 (m, 1H), 4.02 – 3.81 (m, 9H), 2.94 – 2.33 (m, 7H), 2.09 – 1.81 (m, 3H), 1.78 – 1.45 (m, 4H), 1.42 – 1.32 (m, 2H), 1.10 – 0.96 (m, 3H). ^{13}C NMR (100 MHz, $CDCl_3$) δ 172.3, 172.0, 166.4, 166.3, 153.2, 153.1, 148.3, 140.7, 140.6, 138.4, 138.4, 136.6, 136.5, 134.5, 134.5, 130.5, 128.1, 128.0, 127.5, 127.5, 121.8, 121.6, 121.5, 116.7, 116.4, 104.7, 104.5, 66.0, 65.9, 61.0, 56.4, 56.3, 55.0, 53.5, 49.4, 48.0, 46.4, 46.0, 36.5, 36.2, 29.8, 29.4, 29.3, 26.6, 25.8, 22.1, 22.0, 21.9, 21.5, 12.2, 12.2. HRMS (FAB$^+$) m/z calcd. For $[C_{30}H_{39}N_4O_5]^+$: 535.2915, found : 535.2917.

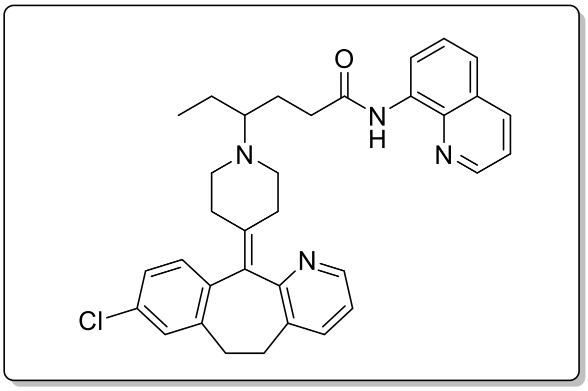

4-(4-(8-Chloro-5,6-dihydro-11*H*-benzo[5,6]cyclohepta[1,2-*b*]pyridin-11-ylidene)piperidin-1-yl)-*N*-(quinolin-8-yl)hexanamide (4q) (mixture of rotamers).

Prepared according to GP3. Monitored by TLC using MeOH:CH_2Cl_2 = 1:19 (Rf = 0.25) as the mobile phase and purified with flash column chromatography on silica gel (MeOH:CH_2Cl_2 = 1:29 to MeOH:CH_2Cl_2 = 1:19). From **1a** (24.0 mg, 0.1 mmol), compound **4q** (32.8 mg, 59%) was obtained. Yellowish oil. ^{1}H NMR (400 MHz, $CDCl_3$) δ 9.88 (s, 1H), 8.86 – 8.72 (m, 2H), 8.40 (d, *J* = 4.2 Hz, 1H), 8.14 (dt, *J* = 8.3, 1.3 Hz, 1H), 7.58 – 7.37 (m, 4H), 7.17 – 7.02 (m, 4H), 3.48 – 3.25 (m, 2H), 2.89 – 2.16 (m, 13H), 1.99 – 1.73 (m, 2H), 1.71 – 1.49 (m, 1H), 1.23 – 1.11 (m, 1H), 0.89 (t, *J* = 7.4 Hz, 3H). ^{13}C NMR (100 MHz, $CDCl_3$) δ 172.6, 158.0, 157.9, 148.2, 148.1, 146.7, 146.7, 140.2, 139.6, 139.6, 138.5, 138.1, 138.0, 137.3, 137.3, 136.4, 136.4, 134.8, 134.8, 133.6, 133.5, 132.6, 132.2, 131.1, 131.0, 129.1, 129.0, 128.1, 127.6, 127.5, 126.1, 126.1, 122.1, 121.7, 121.4, 116.6, 116.5, 65.1, 64.9, 51.0, 50.8, 48.9, 48.6, 35.7, 35.6, 32.4 – 31.1 (m), 26.4, 26.3, 21.6, 12.1, 12.1. HRMS (EI$^+$) m/z calcd. For $[C_{34}H_{35}ClN_4O]^+$: 550.2499, found : 550.2498.

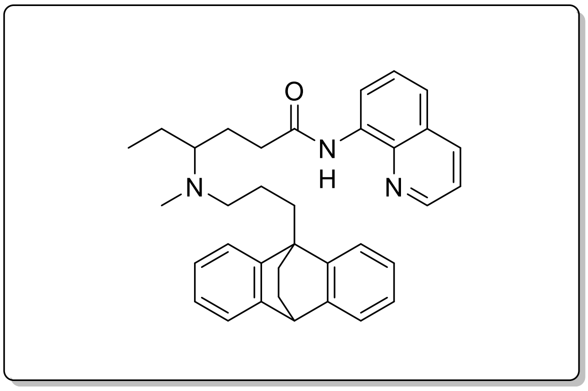

4-((3-(9,10-Ethanoanthracen-9(10*H*)-yl)propyl)(methyl)amino)-*N*-(quinolin-8-yl)hexanamide (4r).

Prepared according to GP3. Monitored by TLC using MeOH:EA:Hx = 1:3 (1% TEA) (Rf = 0.2) as the mobile phase and purified with flash column chromatography on silica gel (EA:Hx = 1:3 to EA:Hx = 1:3 (1% TEA)). From **1a** (24.0 mg, 0.1 mmol), compound **4r** (30.9 mg, 60%) was obtained. Yellowish oil. ^{1}H NMR (600 MHz, CD_2Cl_2) δ 9.93 (s, 1H), 8.77 (d, *J* = 7.0 Hz, 1H), 8.67 (d, *J* = 3.8 Hz, 1H), 8.15

(d, *J* = 8.2 Hz, 1H), 7.55 – 7.46 (m, 2H), 7.40 (dd, *J* = 8.1, 4.1 Hz, 1H), 7.30 – 7.20 (m, 4H), 7.12 – 6.99 (m, 4H), 4.28 – 4.20 (m, 1H), 2.86 – 2.76 (m, 1H), 2.75 – 2.66 (m, 3H), 2.62 – 2.53 (m, 1H), 2.53 – 2.39 (m, 2H), 2.33 (s, 3H), 2.01 – 1.94 (m, 1H), 1.94 – 1.84 (m, 3H), 1.82 – 1.66 (m, 3H), 1.52 – 1.41 (m, 2H), 1.38 – 1.30 (m, 1H), 1.01 (t, *J* = 7.3 Hz, 3H). ^{13}C NMR (100 MHz, CD_2Cl_2) δ 172.7, 148.7, 146.4, 145.7, 145.7, 138.9, 136.8, 135.5, 128.6, 127.8, 125.7, 125.6, 123.7, 123.7, 122.1, 121.9, 121.6, 116.5, 65.3, 55.3, 45.4, 45.1, 37.0, 36.2, 30.1, 29.3, 28.2, 26.8, 24.3, 21.9, 12.5. HRMS (EI^+) m/z calcd. For $[C_{35}H_{39}N_3O]^+$: 517.3093, found : 517.3093.

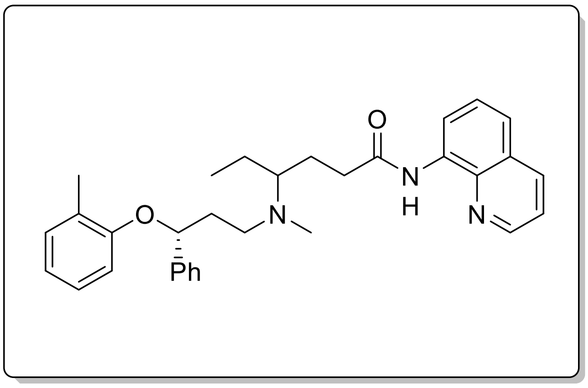

4-(Methyl((*R*)-3-phenyl-3-(*o*-tolyloxy)propyl)amino)-*N*-(quinolin-8-yl)hexanamide (4s) (diastereomer 1 : 1).

Prepared according to GP3. Monitored by TLC using EA:Hx = 1:3 (Rf = 0.2) as the mobile phase and purified with preparative TLC on silica gel (EA:Hx = 1:3). From **1a** (24.0 mg, 0.1 mmol), compound **4s** (31.0 mg, 60%) was obtained. Yellowish oil. ^{1}H NMR (600 MHz, CD_2Cl_2) δ 9.93 – 9.69 (m, 1H), 8.83 – 8.69 (m, 2H), 8.19 (d, *J* = 7.7 Hz, 1H), 7.56 – 7.49 (m, 2H), 7.46 (dd, *J* = 8.1, 4.1 Hz, 1H), 7.37 – 7.26 (m, 4H), 7.22 (t, *J* = 7.1 Hz, 1H), 7.13 – 7.03 (m, 1H), 6.98 – 6.82 (m, 1H), 6.77 – 6.57 (m, 2H), 5.36 – 5.27 (m, 1H), 2.82 – 2.52 (m, 3H), 2.51 – 2.35 (m, 2H), 2.33 – 2.27 (m, 3H), 2.26 – 2.20 (m, 3H), 2.19 – 2.08 (m, 1H), 2.05 – 1.95 (m, 1H), 1.93 – 1.69 (m, 2H), 1.67 – 1.54 (m, 1H), 1.26 – 1.20 (m, 1H), 0.91 (t, *J* = 7.3 Hz, 3H). ^{13}C NMR (100 MHz, CD_2Cl_2) δ 172.7, 172.6, 156.8, 156.6, 148.7, 148.7, 143.4, 143.3, 138.9, 136.8, 135.5, 135.5, 131.1, 131.0, 129.0, 128.6, 127.8, 127.5, 127.5, 127.0, 126.9, 126.4, 126.4, 122.2, 121.6, 121.6, 120.6, 120.5, 116.5, 116.5, 113.2, 113.1, 77.9, 77.7, 65.6, 64.9, 51.2, 50.0, 38.4, 38.3, 37.0, 36.0, 36.0, 35.7, 26.6, 26.5, 21.9, 21.6, 16.8, 16.8, 12.4, 12.3. HRMS (EI^+) m/z calcd. For $[C_{32}H_{37}N_3O_2]^+$: 495.2886, found : 495.2882.

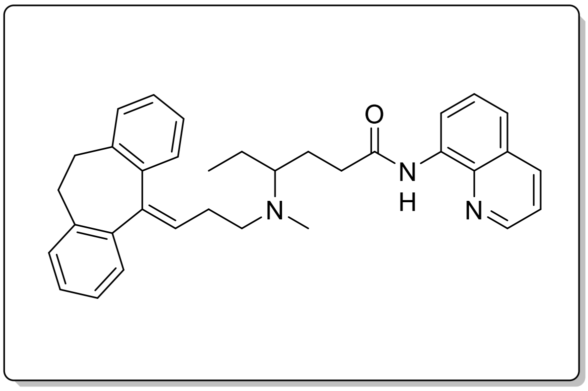

4-((3-(10,11-Dihydro-5*H*-dibenzo[*a*,*d*][7]annulen-5-ylidene)propyl)(methyl)amino)-*N*-(quinolin-8-yl)hexanamide (4t).

Prepared according to GP3. Monitored by TLC using MeOH:EA:Hx = 1:9:10 (Rf = 0.25) as the mobile phase and purified with flash column chromatography on silica gel (EA:Hx = 1:2 to MeOH:EA:Hx = 1:9:10) and sequentially purified with flash column chromatography on silica gel (MeOH:CH_2Cl_2 = 1:29 to MeOH:CH_2Cl_2 = 1:19). From **1a** (24.0 mg, 0.1 mmol), compound **4t** (23.9 mg, 47%) was obtained. Yellowish oil. ^{1}H NMR (400 MHz, $CDCl_3$) δ 9.86 (s, 1H), 8.84 – 8.70 (m, 2H), 8.15 (dd, *J* = 8.3, 1.6 Hz, 1H), 7.58 – 7.47 (m, 2H), 7.43 (dd, *J* = 8.3, 4.2 Hz, 1H), 7.29 – 7.23 (m, 1H), 7.20 – 7.11 (m, 4H), 7.10 – 7.04 (m, 2H), 7.02 – 6.94 (m, 1H), 5.89 (t, *J* = 7.4 Hz, 1H), 3.54 – 3.16 (m, 2H), 3.06 – 2.71 (m, 2H), 2.69 – 2.26 (m, 7H), 2.21 (s, 3H), 1.99 – 1.73 (m, 2H), 1.68 – 1.46 (m, 1H), 1.29 – 1.16 (m, 1H), 0.91 (t, *J* = 7.2 Hz, 3H). ^{13}C NMR (100 MHz, $CDCl_3$) δ 172.4, 148.2, 143.6, 141.4, 140.2, 139.4, 138.5, 137.1, 136.4, 134.9, 130.0, 129.8, 128.7, 128.3, 128.1, 128.1, 127.6, 127.5, 127.0, 126.1, 125.8, 121.7, 121.4, 116.5, 64.4, 53.4, 36.8, 35.5, 33.9, 32.2, 28.6, 26.1, 21.5, 12.0. HRMS (FAB$^+$) m/z calcd. For $[C_{34}H_{38}N_3O]^+$: 504.3015, found : 504.3018.

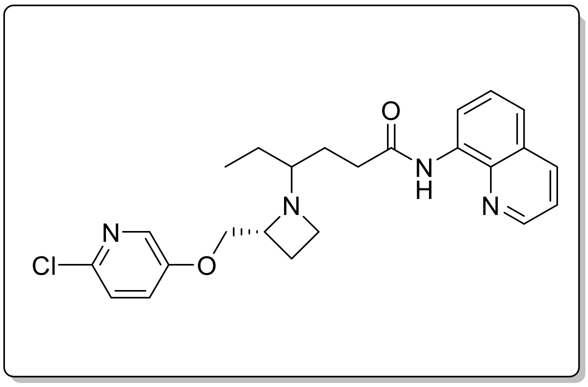

4-((*R*)-2-(((6-Chloropyridin-3-yl)oxy)methyl)azetidin-1-yl)-*N*-(quinolin-8-yl)hexanamide (4u) (diastereomer 1.6 : 1).

Prepared according to GP3. Monitored by TLC using MeOH:CH_2Cl_2 = 1:19 (Rf = 0.2) as the mobile phase and purified with preparative TLC on silica gel (MeOH:CH_2Cl_2 = 1:19) and sequentially purified with flash column chromatography on alumina basic (EA:Hx = 1:3). From **1a** (24.0 mg, 0.1 mmol),

compound **4u** (26.8 mg, 61%) was obtained. Yellowish oil. ^{1}H NMR (600 MHz, $CDCl_3$) δ 9.89 – 9.60 (m, 0H), 8.84 – 8.62 (m, 1H), 8.21 – 8.09 (m, 1H), 8.07 – 7.85 (m, 0H), 7.58 – 7.36 (m, 1H), 7.21 – 6.68 (m, 1H), 4.10 – 3.88 (m, 1H), 3.67 – 3.53 (m, 1H), 3.51 – 3.31 (m, 1H), 3.07 – 2.92 (m, 0H), 2.73 – 2.57 (m, 1H), 2.55 – 2.43 (m, 1H), 2.42 – 2.33 (m, 1H), 2.16 – 1.79 (m, 2H), 1.63 – 1.49 (m, 1H), 1.47 – 1.21 (m, 1H), 0.96 – 0.83 (m, 1H). ^{13}C NMR (100 MHz, $CDCl_3$) δ 172.1, 171.9, 154.4, 154.2, 148.2, 148.1, 142.6, 142.2, 138.4, 138.1, 136.8, 136.5, 136.4, 136.3, 134.7, 134.5, 128.0, 127.9, 127.5, 127.4, 125.0, 124.9, 124.4, 123.9, 121.7, 121.5, 121.5, 116.4, 116.3, 73.3, 72.9, 66.3, 66.2, 62.5, 62.2, 50.1, 48.9, 33.1, 32.1, 24.7, 24.1, 22.3, 21.4, 20.3, 19.7, 10.3, 9.2. HRMS (EI$^+$) m/z calcd. For $[C_{24}H_{27}ClN_4O_2]^+$: 438.1823, found : 438.1826.

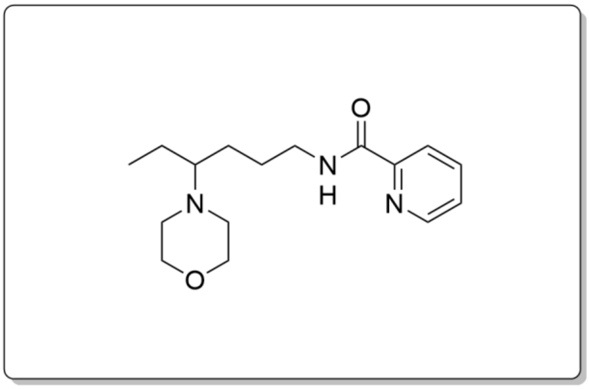

N-(4-Morpholinohexyl)picolinamide (6a)

Prepared according to GP5. Monitored by TLC using MeOH:EA:Hx = 1:9:20 (Rf = 0.3) as the mobile phase and purified with flash column chromatography on silica gel (MeOH:EA:Hx = 1:9:20). From **5a** (20.4 mg, 0.1 mmol), compound **6a** (18.0 mg, 62%) was obtained. Reddish oil. ^{1}H NMR (400 MHz, CD_2Cl_2) δ 8.54 (ddd, *J* = 4.8, 1.8, 0.9 Hz, 1H), 8.14 (dt, *J* = 7.8, 1.1 Hz, 1H), 8.10 (s, 1H), 7.85 (td, *J* = 7.7, 1.7 Hz, 1H), 7.43 (ddd, *J* = 7.6, 4.8, 1.3 Hz, 1H), 3.68 – 3.57 (m, 4H), 3.51 – 3.35 (m, 2H), 2.57 – 2.49 (m, 2H), 2.49 – 2.42 (m, 2H), 2.26 (tt, *J* = 7.6, 5.9 Hz, 1H), 1.72 – 1.67 (m, 1H), 1.66 – 1.44 (m, 3H), 1.43 – 1.34 (m, 1H), 1.27 – 1.21 (m, 1H), 0.89 (t, *J* = 7.4 Hz, 3H). ^{13}C NMR (100 MHz, CD_2Cl_2) δ 164.5, 150.9, 148.6, 137.9, 126.5, 122.4, 68.1, 66.1, 54.5, 54.3, 54.0, 53.7, 53.5, 49.4, 40.1, 27.5, 27.4, 22.4, 12.0.HRMS (EI$^+$) m/z calcd. For $[C_{16}H_{25}N_3O_2]^+$: 291.1947, found : 291.1950.

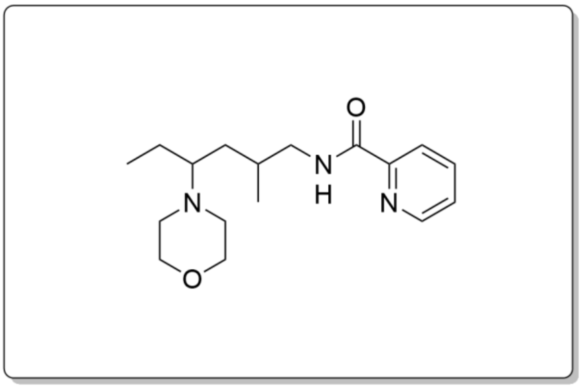

***N*-(2-Methyl-4-morpholinohexyl)picolinamide (6b) (diasteromer 2.6:1)**

Prepared according to GP5. Monitored by TLC using Hx:Acetone = 3:1 (Rf = 0.3) as the mobile phase and purified with preparative TLC (Hx:Acetone = 3:1). From **5b** (21.8 mg, 0.1 mmol), compound **6b** (14.7 mg, 48%) was obtained. Reddish oil. ^{1}H NMR (600 MHz, $CDCl_3$) δ 8.53 (dd, J = 8.9, 4.6 Hz, 1H), 8.33 – 8.15 (m, 2H), 7.84 (t, J = 7.8 Hz, 1H), 7.41 (dd, J = 7.6, 4.8 Hz, 1H), 3.76 – 3.61 (m, 4H), 3.49 – 3.36 (m, 1H), 3.35 – 3.25 (m, 1H), 2.64 – 2.54 (m, 2H), 2.53 – 2.40 (m, 2H), 2.40 – 2.28 (m, 1H), 2.05 – 1.93 (m, 1H), 1.60 (dtt, J = 26.9, 13.4, 5.9 Hz, 1H), 1.54 – 1.40 (m, 1H), 1.38 – 1.17 (m, 1H), 1.17 – 1.10 (m, 1H), 0.99 (dd, J = 11.8, 6.7 Hz, 3H), 0.92 – 0.83 (m, 3H). ^{13}C NMR (100 MHz, $CDCl_3$) δ 164.5, 164.4, 150.3, 150.2, 148.1, 148.0, 137.4, 137.4, 126.1, 126.1, 122.3, 122.3, 77.5, 77.2, 76.8, 67.7, 67.7, 63.9, 63.2, 48.8, 48.7, 45.5, 45.5, 35.2, 34.8, 31.3, 30.4, 21.7, 21.7, 19.0, 18.3, 12.0, 11.8. HRMS (ESI$^+$) m/z calcd. For $[C_{17}H_{28}N_3O_2]^+$: 306.2176, found : 306.2175.

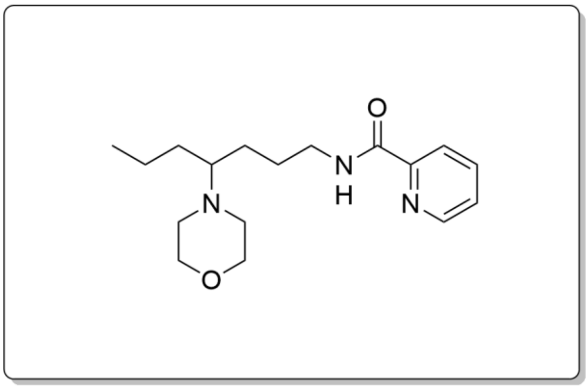

***N*-(4-Morpholinoheptyl)picolinamide (6c)**

Prepared according to modified GP5 (Reaction was conducted at 60 °C with 2.5 equiv of morpholino benzoate) . Monitored by TLC using MeOH:EA:Hx = 1:9:20 (Rf = 0.3) as the mobile phase and purified with flash column chromatography on silica gel (MeOH:EA:Hx = 1:9:20). From **5c** (21.8 mg, 0.1 mmol), compound **6c** (9.1 mg, 30%) was obtained. Reddish oil. ^{1}H NMR (400 MHz, $CDCl_3$) δ 8.54 (dt, *J* = 4.8, 1.3 Hz, 1H), 8.20 (dt, *J* = 7.8, 1.2 Hz, 1H), 8.11 (s, 1H), 7.84 (td, *J* = 7.7, 1.7 Hz, 1H), 7.42 (ddd, *J* = 7.7, 4.8, 1.3 Hz, 1H), 3.68 (t, *J* = 4.7 Hz, 4H), 3.55 – 3.38 (m, 2H), 2.53 (t, *J* = 16.4 Hz, 4H), 2.35 (s,

1H), 1.83 – 1.68 (m, 2H), 1.58 – 1.47 (m, 2H), 1.44 – 1.37 (m, 1H), 1.32 (dt, *J* = 15.1, 7.1 Hz, 2H), 1.20 (q, *J* = 7.3, 6.6 Hz, 1H), 0.89 (t, *J* = 7.2 Hz, 3H). ^{13}C NMR (100 MHz, $CDCl_3$) δ 164.4, 150.2, 148.1, 137.5, 126.2, 122.3, 77.5, 77.2, 76.8, 67.8, 63.7, 48.9, 39.7, 31.6, 27.6, 27.2, 20.5, 14.4. HRMS (EI$^+$) m/z calcd. For $[C_{17}H_{27}N_3O_2]^+$: 305.2103, found : 305.2106.

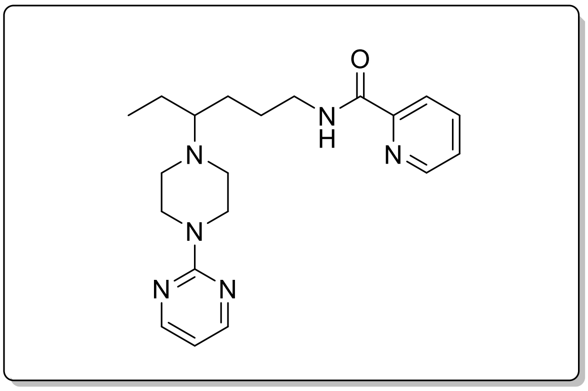

***N*-(4-(4-(Pyrimidin-2-yl)piperazin-1-yl)hexyl)picolinamide (6d).**

Prepared according to GP5. Monitored by TLC using EA:Hx = 1:1 (Rf = 0.15) as the mobile phase and purified with preparative TLC on silica gel (EA:Hx = 1:1 and EA:Hx = 1:1 (1% TEA). From **5a** (20.4 mg, 0.1 mmol), compound **6d** (21.9 mg, 60%) was obtained. Pale orange oil. ^{1}H NMR (400 MHz, $CDCl_3$) δ 8.53 (dt, *J* = 4.7, 1.4 Hz, 1H), 8.28 (d, *J* = 4.8 Hz, 2H), 8.19 (dt, *J* = 7.8, 1.1 Hz, 1H), 8.12 (s, 1H), 7.83 (td, *J* = 7.7, 1.8 Hz, 1H), 7.41 (ddd, *J* = 7.6, 4.8, 1.2 Hz, 1H), 6.44 (t, *J* = 4.7 Hz, 1H), 3.89 – 3.71 (m, 4H), 3.57 – 3.35 (m, 2H), 2.62 (dt, *J* = 10.5, 5.0 Hz, 2H), 2.53 (dt, *J* = 10.9, 5.1 Hz, 2H), 2.42 – 2.29 (m, 1H), 1.83 – 1.64 (m, 2H), 1.64 – 1.47 (m, 2H), 1.50 – 1.37 (m, 1H), 1.32 – 1.15 (m, 1H), 0.89 (t, *J* = 7.4 Hz, 3H). ^{13}C NMR (100 MHz, $CDCl_3$) δ 164.4, 161.8, 157.8, 150.2, 148.1, 137.5, 126.2, 122.3, 109.7, 65.6, 48.3, 44.5, 39.8, 27.4, 27.2, 22.2, 12.0. HRMS (EI$^+$) m/z calcd. For $[C_{21}H_{29}N_5O]^+$: 368.2325, found : 368.2322.

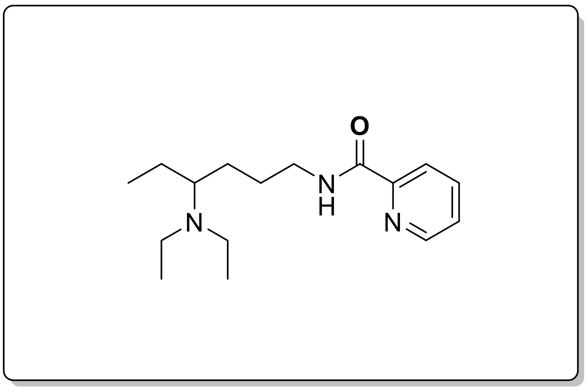

***N*-(4-(Diethylamino)hexyl)picolinamide (6e).**

Prepared according to GP5. Monitored by TLC using EA:Hx = 1:1 (1% TEA) (Rf = 0.3) as the mobile

phase and purified with preparative TLC on silica gel (EA:Hx = 1:1 and EA:Hx = 1:1 (1% TEA). From **5a** (20.4 mg, 0.1 mmol), compound **6e** (14.1 mg, 51%, mixtures with *β*-pdt) was obtained. Colorless oil. ^{1}H NMR (400 MHz, CD_2Cl_2) δ 8.54 (ddd, *J* = 4.8, 1.7, 0.9 Hz, 1H), 8.21 – 8.07 (m, 1H), 8.08 (s, 1H), 7.85 (td, *J* = 7.7, 1.7 Hz, 1H), 7.43 (ddd, *J* = 7.6, 4.8, 1.3 Hz, 1H), 3.47 – 3.29 (m, 2H), 2.55 – 2.30 (m, 5H), 1.82 – 1.55 (m, 2H), 1.56 – 1.30 (m, 3H), 1.28 – 1.10 (m, 1H), 0.98 (t, *J* = 7.1 Hz, 6H), 0.87 (t, *J* = 7.4 Hz, 3H). ^{13}C NMR (100 MHz, CD_2Cl_2) δ 164.4, 150.9, 148.6, 137.9, 126.5, 122.4, 61.5, 43.8, 40.2, 28.3, 28.1, 23.2, 15.3, 12.5. HRMS (EI$^+$) m/z calcd. For $[C_{16}H_{27}N_3O]^+$: 277.2154, found : 277.2151.

Appendix I

Spectral Copies of ^{1}H-, ^{13}C-, and ^{19}F-NMR Data Obtained in this Study

^{1}H NMR 600 MHz, CDCl$_3$

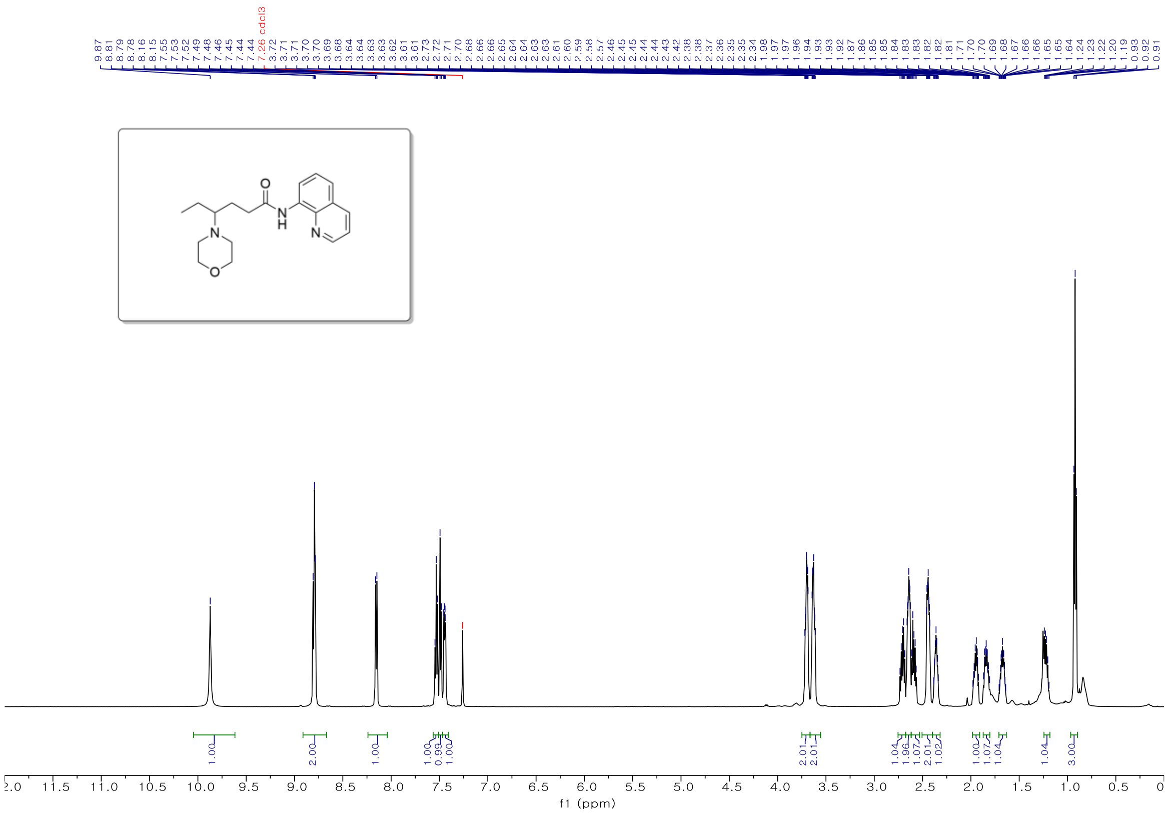

^{13}C NMR 150 MHz, CDCl$_3$

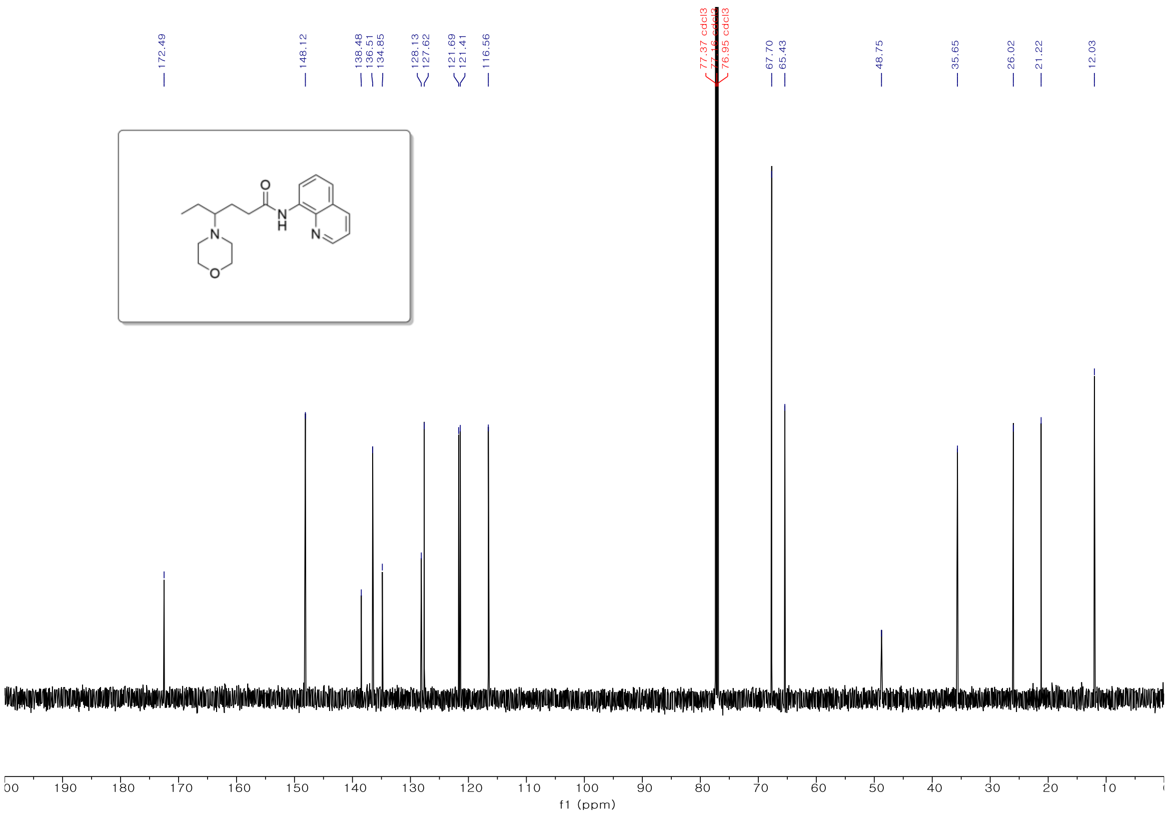

Supplementary Figure 12. ^{1}H and ^{13}C NMR of **3a**

^{1}H NMR 400 MHz, $CDCl_3$

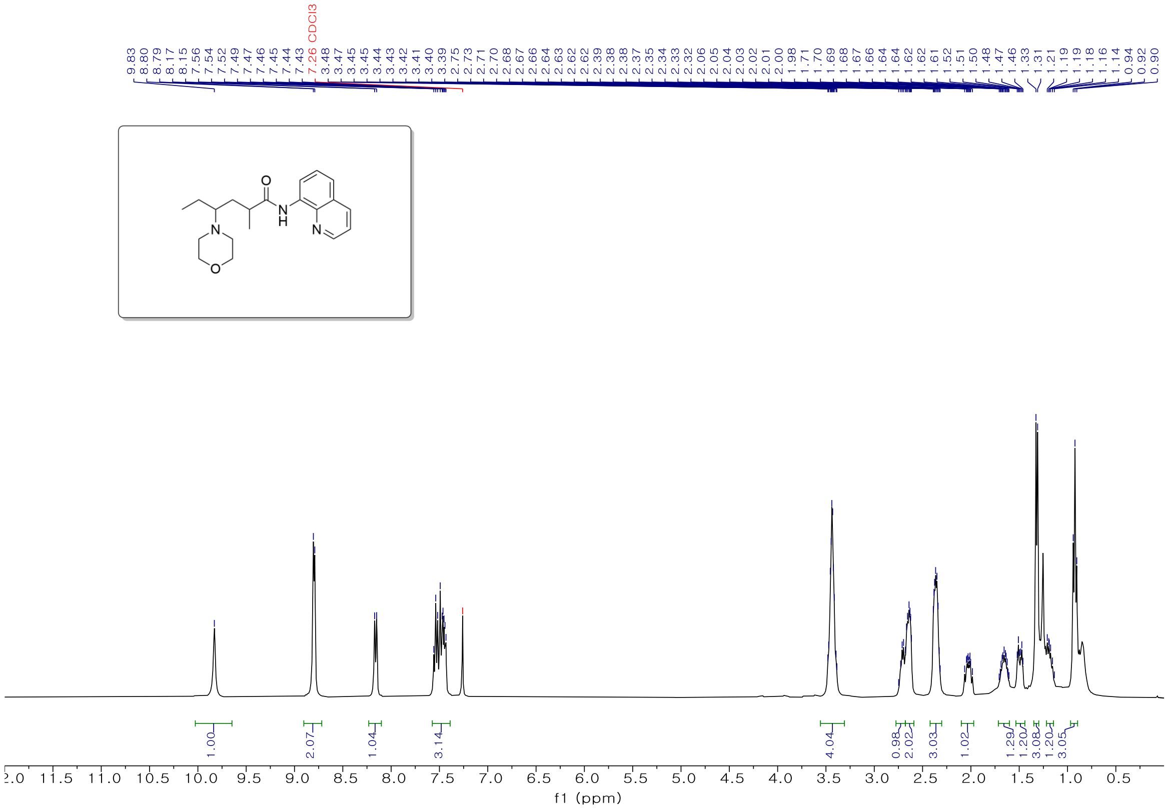

^{13}C NMR 100 MHz, $CDCl_3$

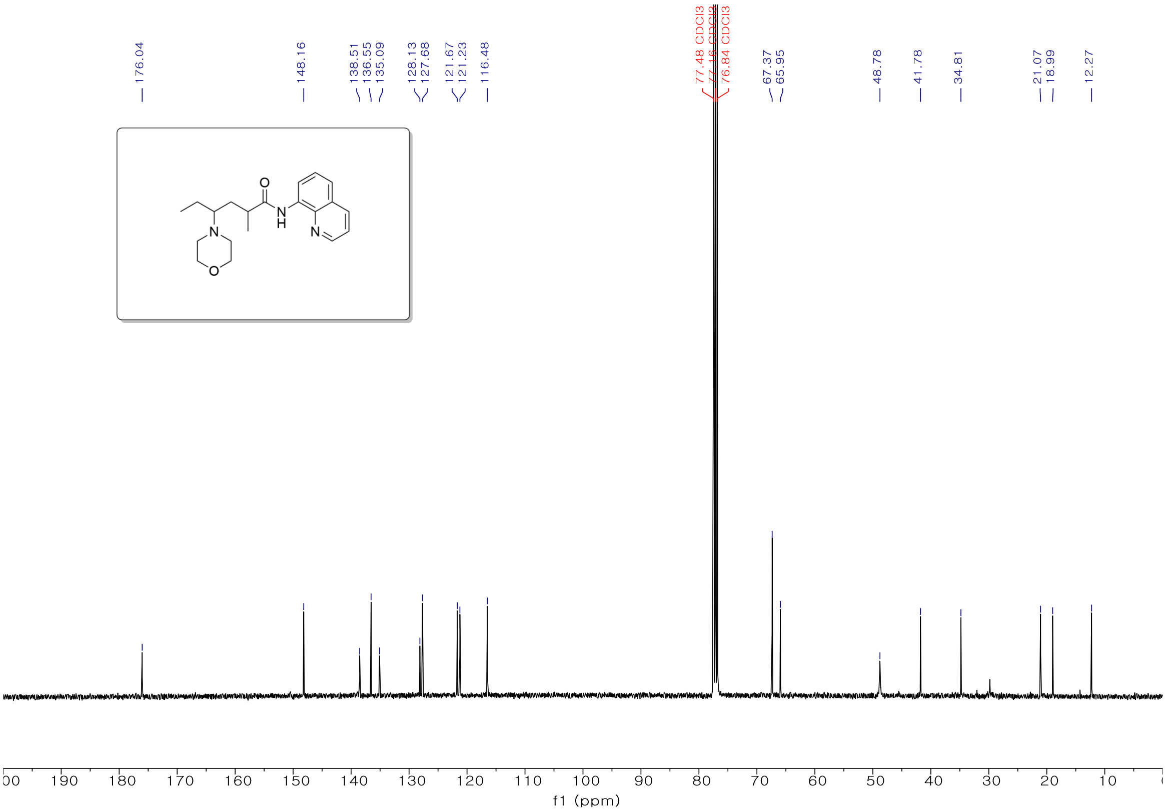

Supplementary Figure 13. ^{1}H and ^{13}C NMR of **3b** (diastereomer 5.4 : 1)

^{1}H NMR 400 MHz, $CDCl_3$

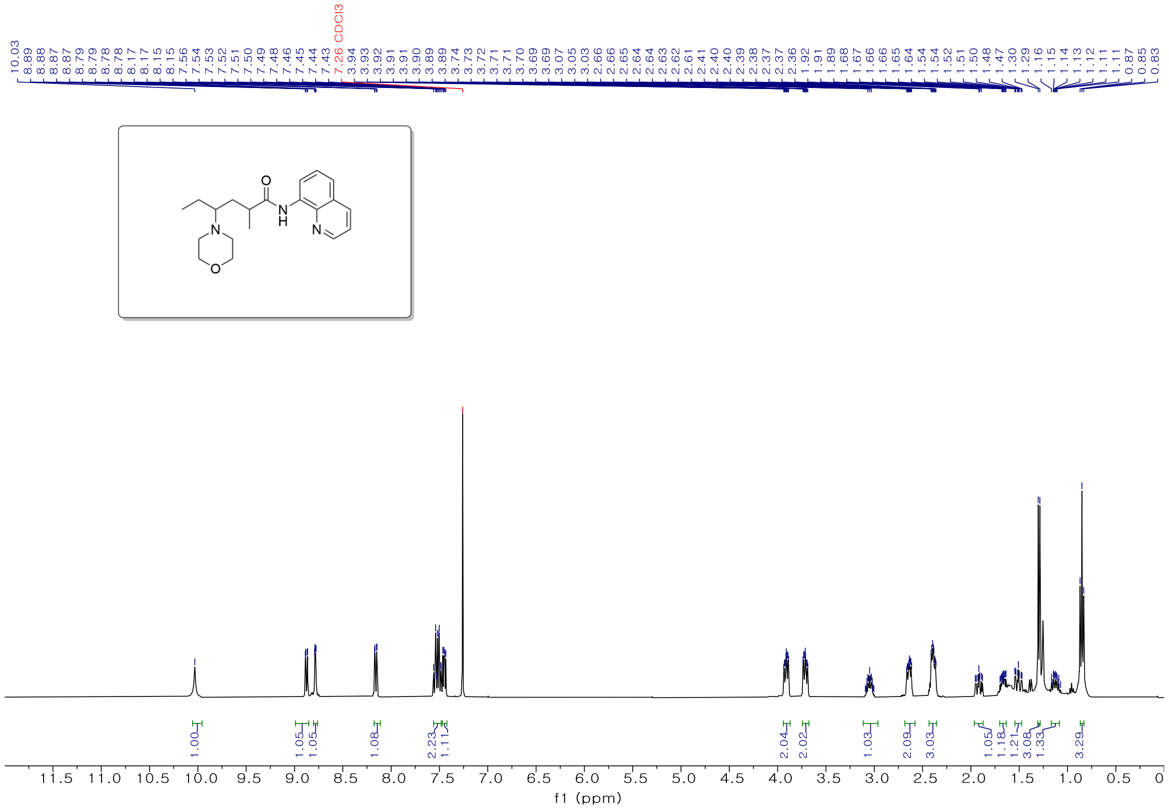

^{13}C NMR 100 MHz, $CDCl_3$

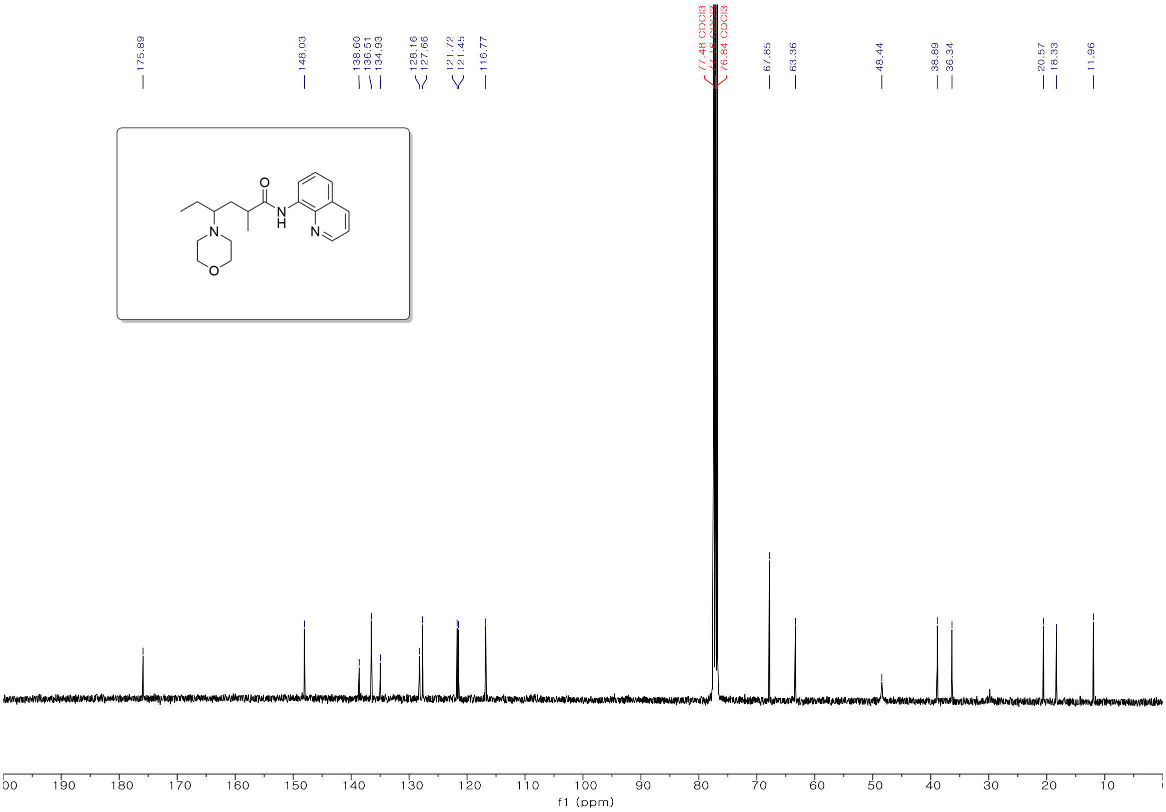

Supplementary Figure 14. ^{1}H and ^{13}C NMR of **3b'** (diastereomer 5.4 : 1)

^{1}H NMR 400 MHz, $CDCl_3$

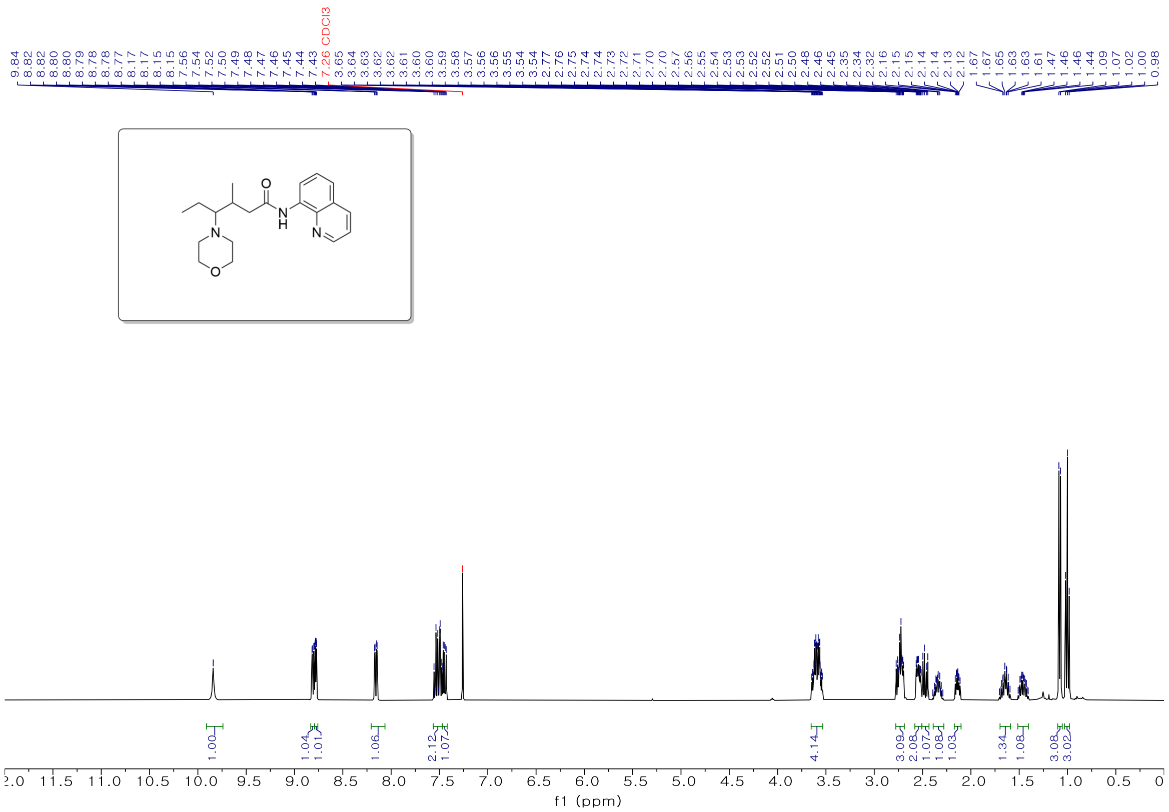

^{13}C NMR 100 MHz, $CDCl_3$

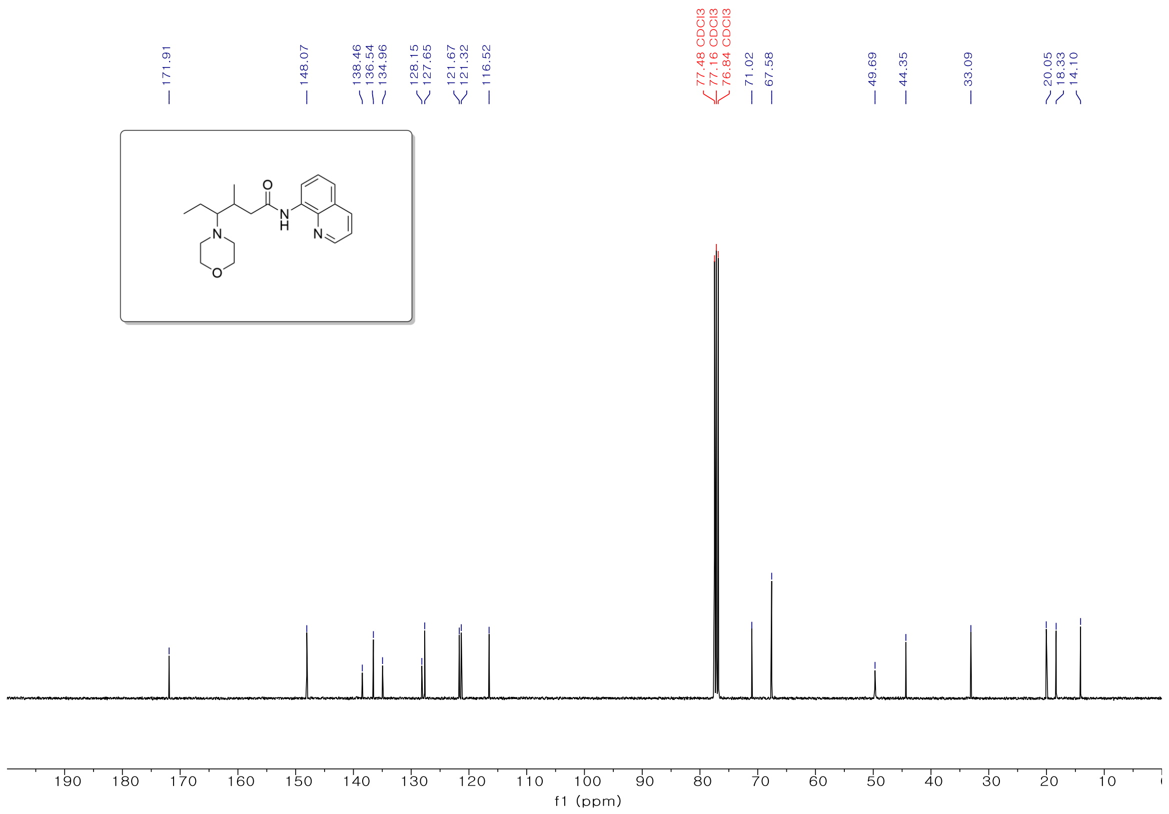

Supplementary Figure 15. ^{1}H and ^{13}C NMR of **3c** (diastereomer 2:1)

^{1}H NMR 400 MHz, $CDCl_3$

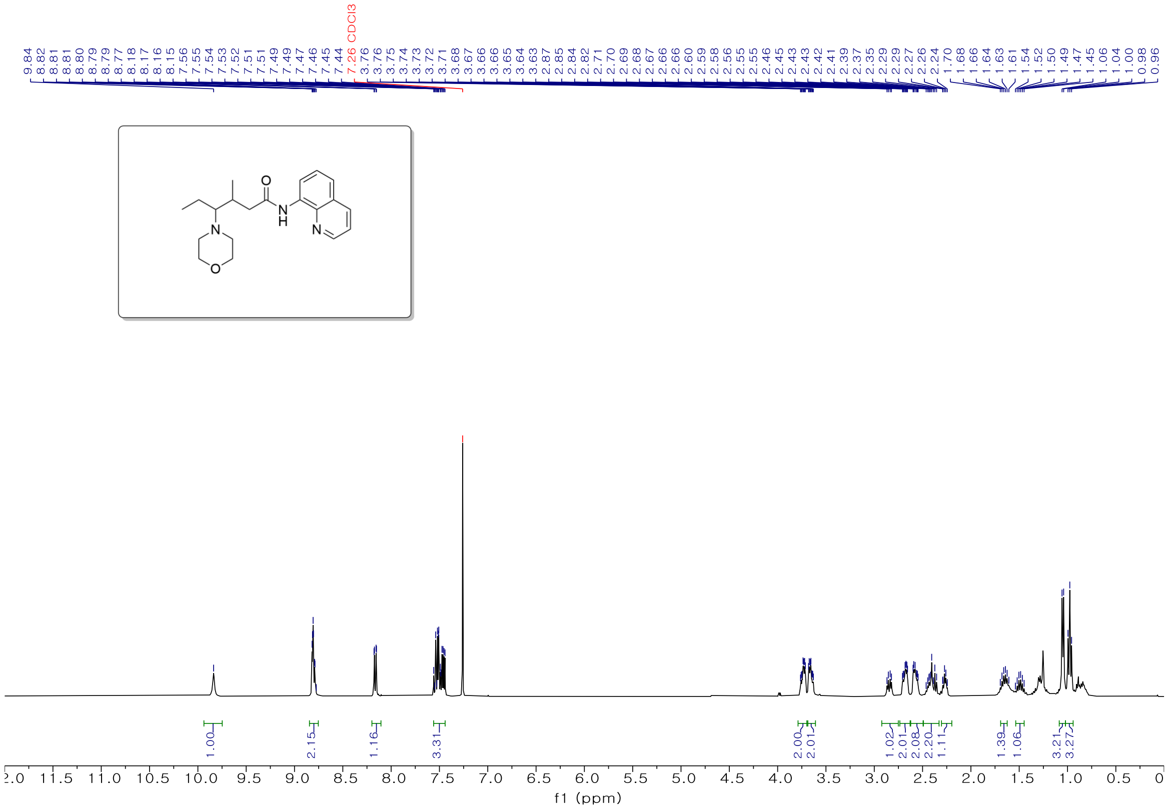

^{13}C NMR 100 MHz, $CDCl_3$

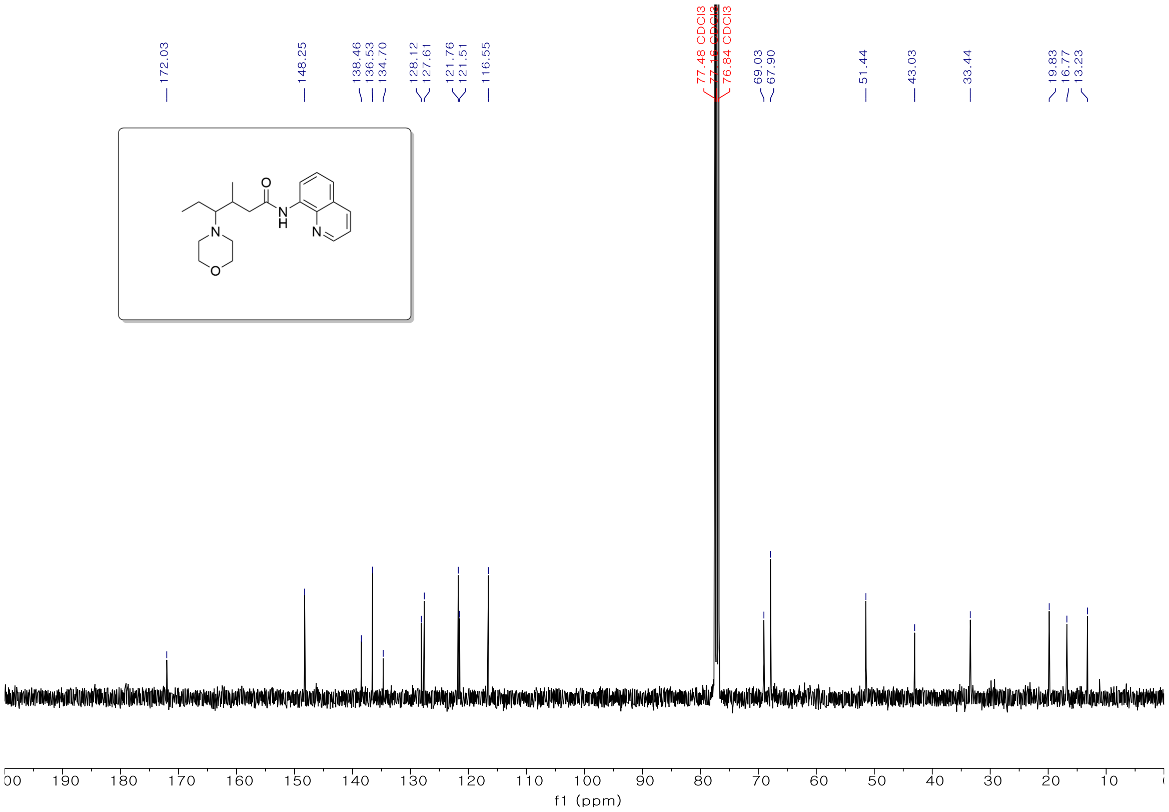

Supplementary Figure 16. ^{1}H and ^{13}C NMR of **3c'** (diastereomer 2:1)

^{1}H NMR 400 MHz, $CDCl_3$

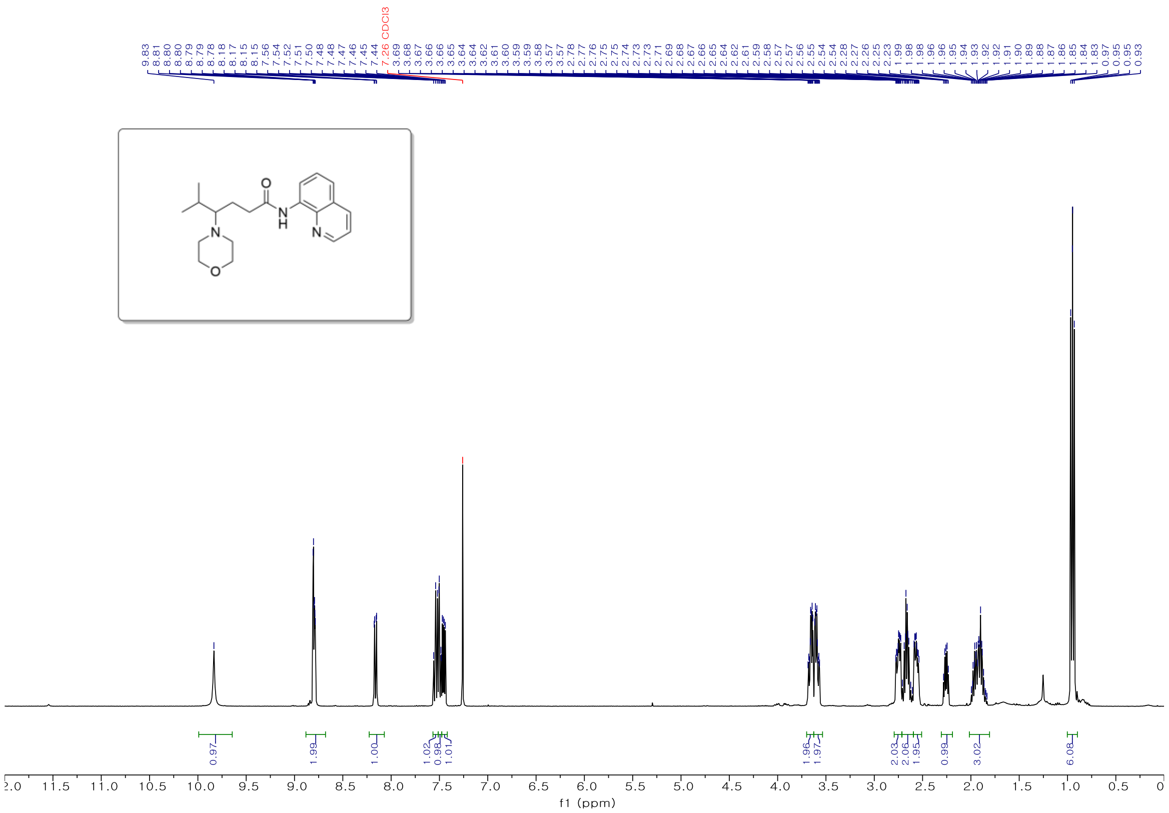

^{13}C NMR 100 MHz, $CDCl_3$

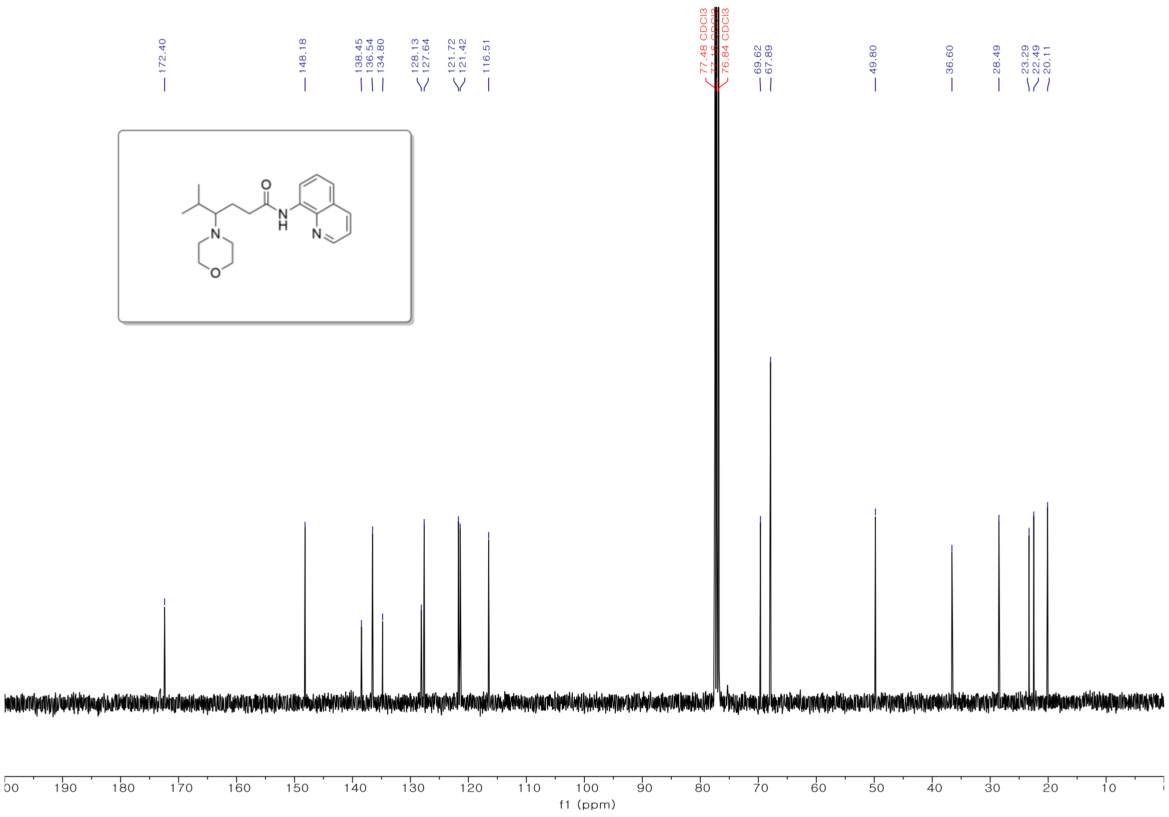

Supplementary Figure 17. ^{1}H and ^{13}C NMR of **3d**

^{1}H NMR 600 MHz, $CDCl_3$

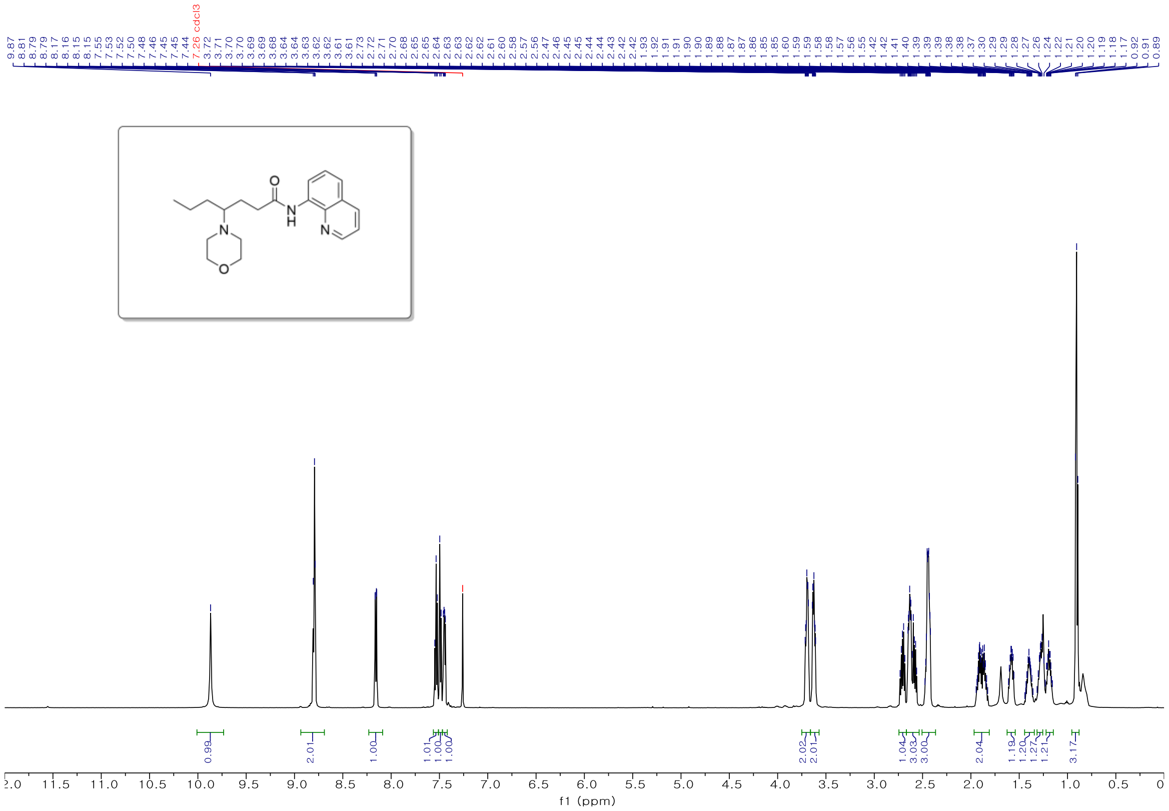

^{13}C NMR 150 MHz, $CDCl_3$

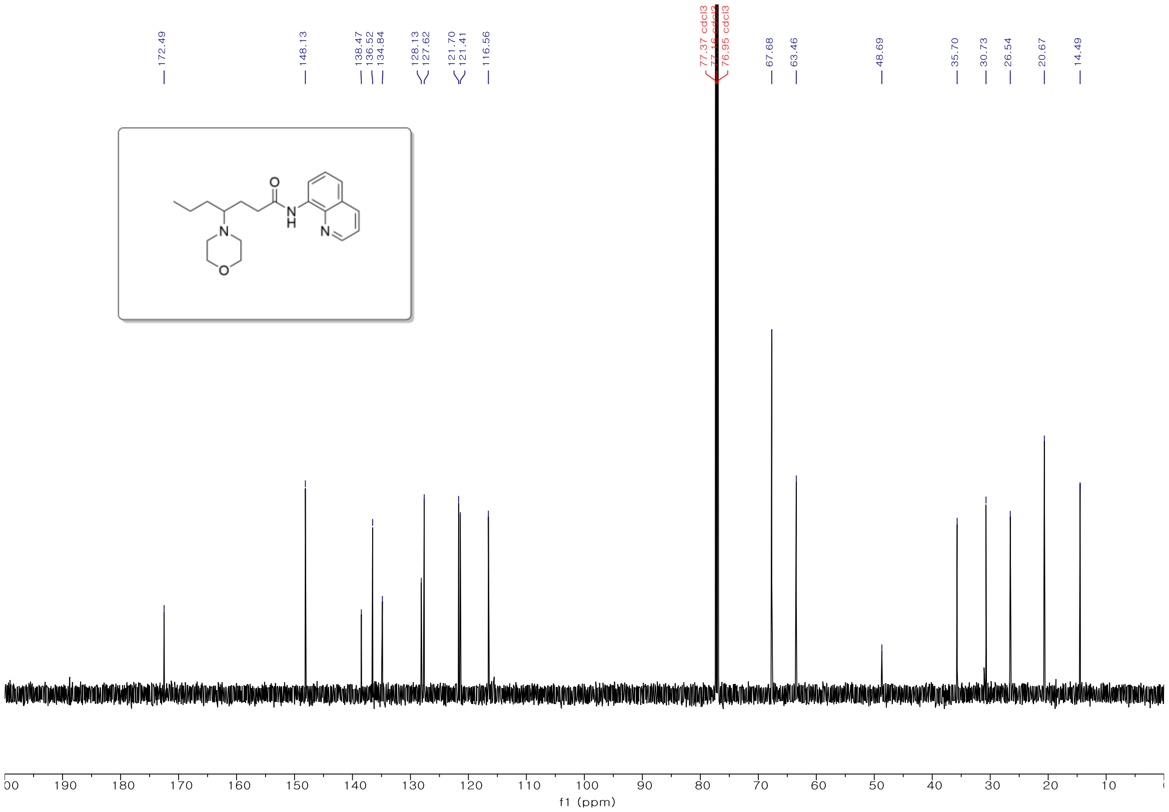

Supplementary Figure 18. ^{1}H and ^{13}C NMR of **3e**

^{1}H NMR 600 MHz, CDCl$_3$

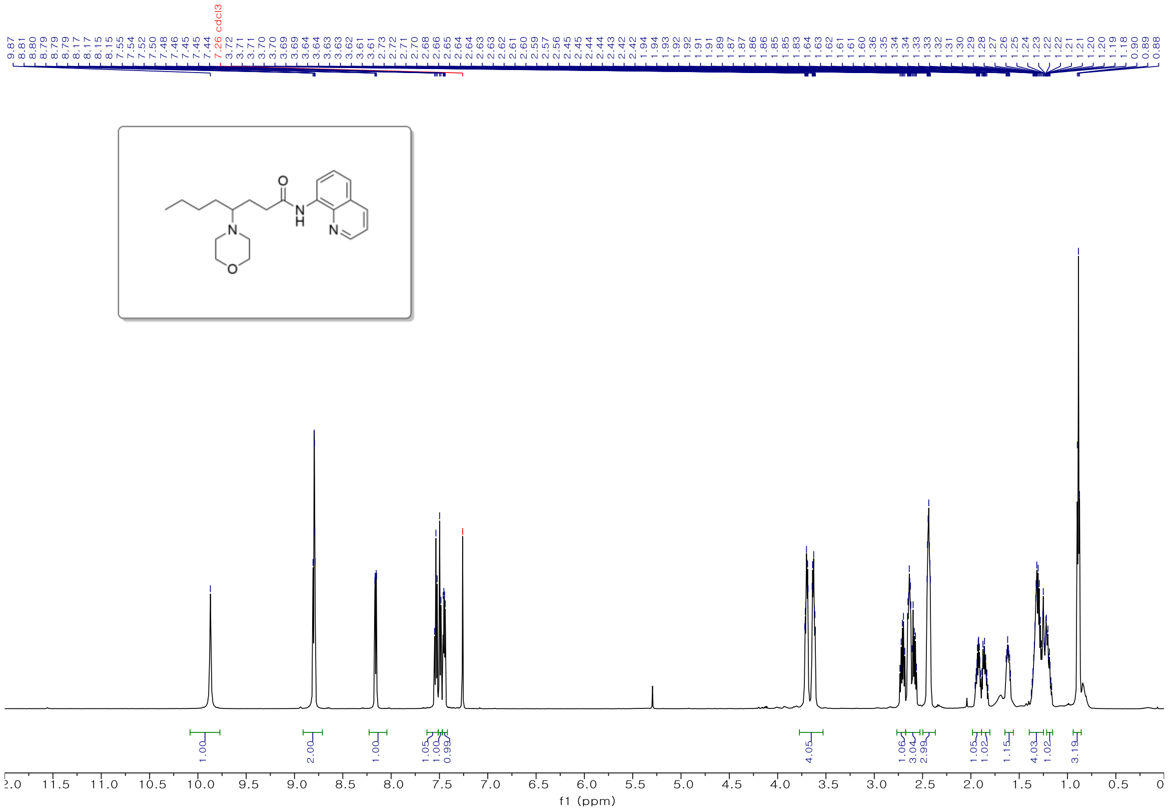

^{13}C NMR 150 MHz, CDCl$_3$

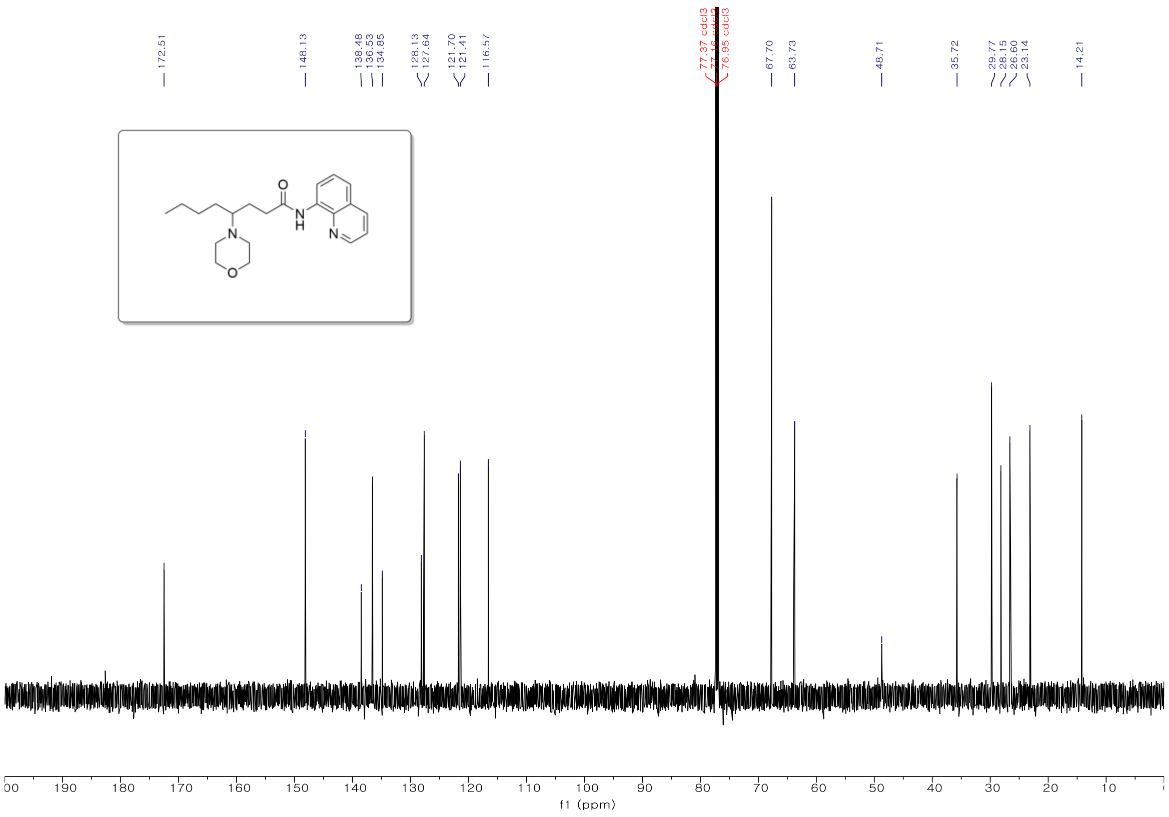

Supplementary Figure 19. ^{1}H and ^{13}C NMR of **3f**

^{1}H NMR 400 MHz, CD_2Cl_2

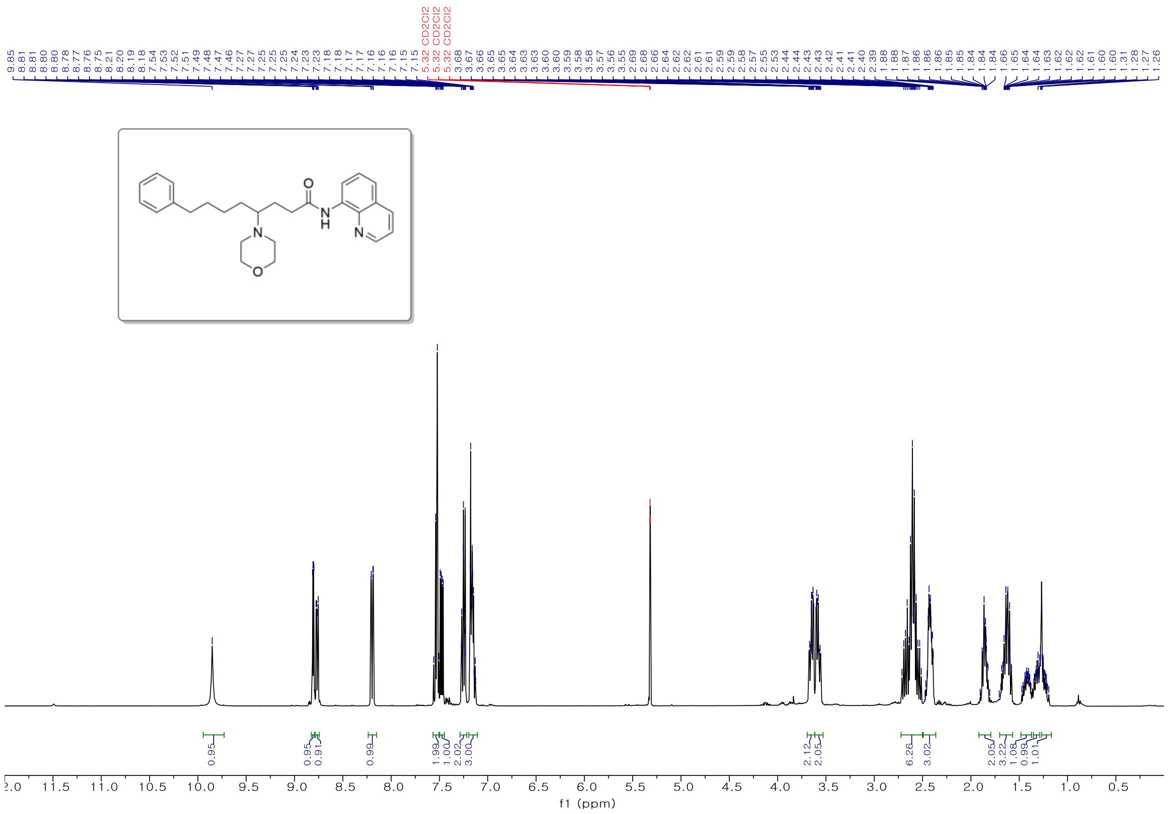

^{13}C NMR 100 MHz, CD_2Cl_2

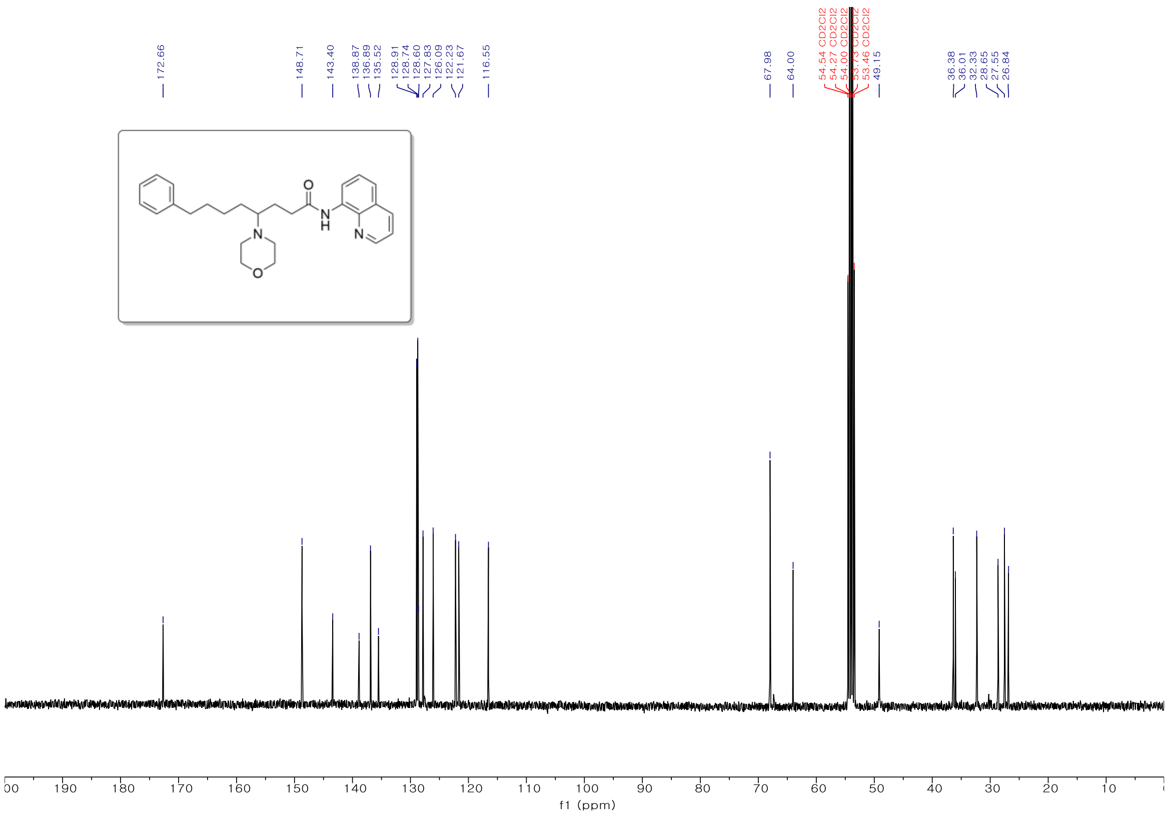

Supplementary Figure 20. ^{1}H and ^{13}C NMR of **3g**

^{1}H NMR 600 MHz, $CDCl_3$

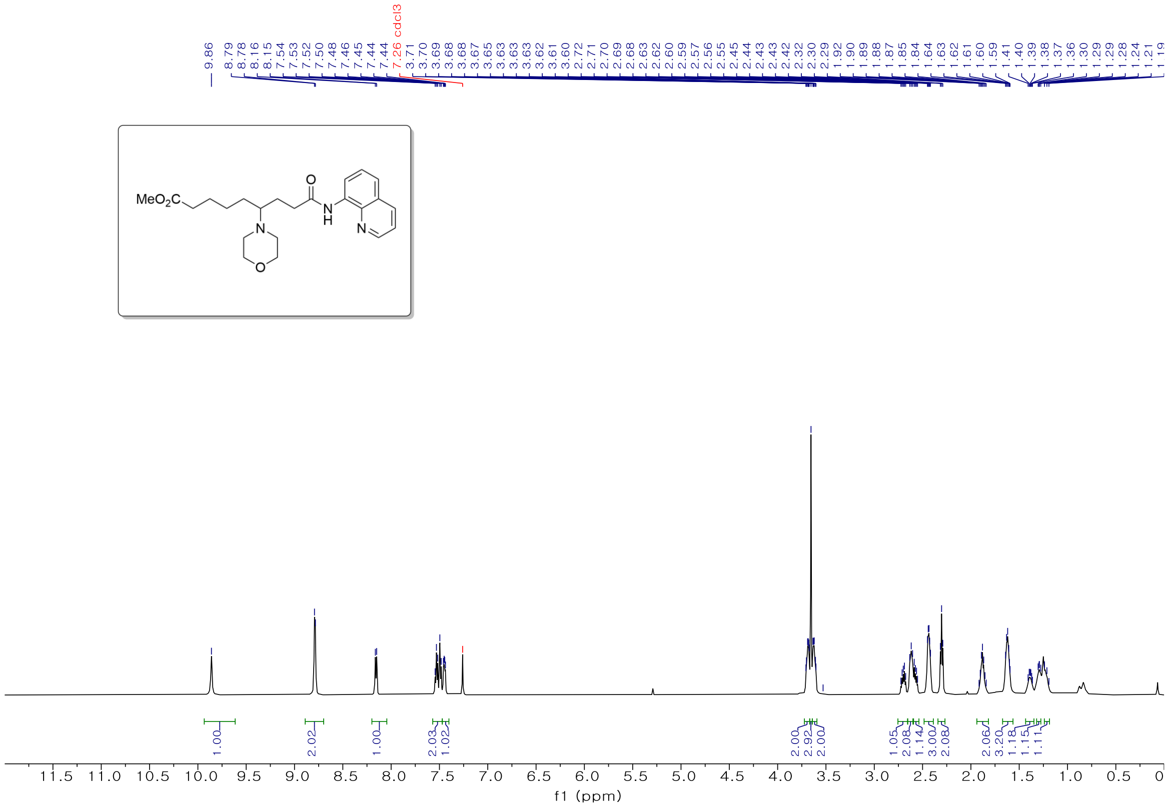

^{13}C NMR 100 MHz, $CDCl_3$

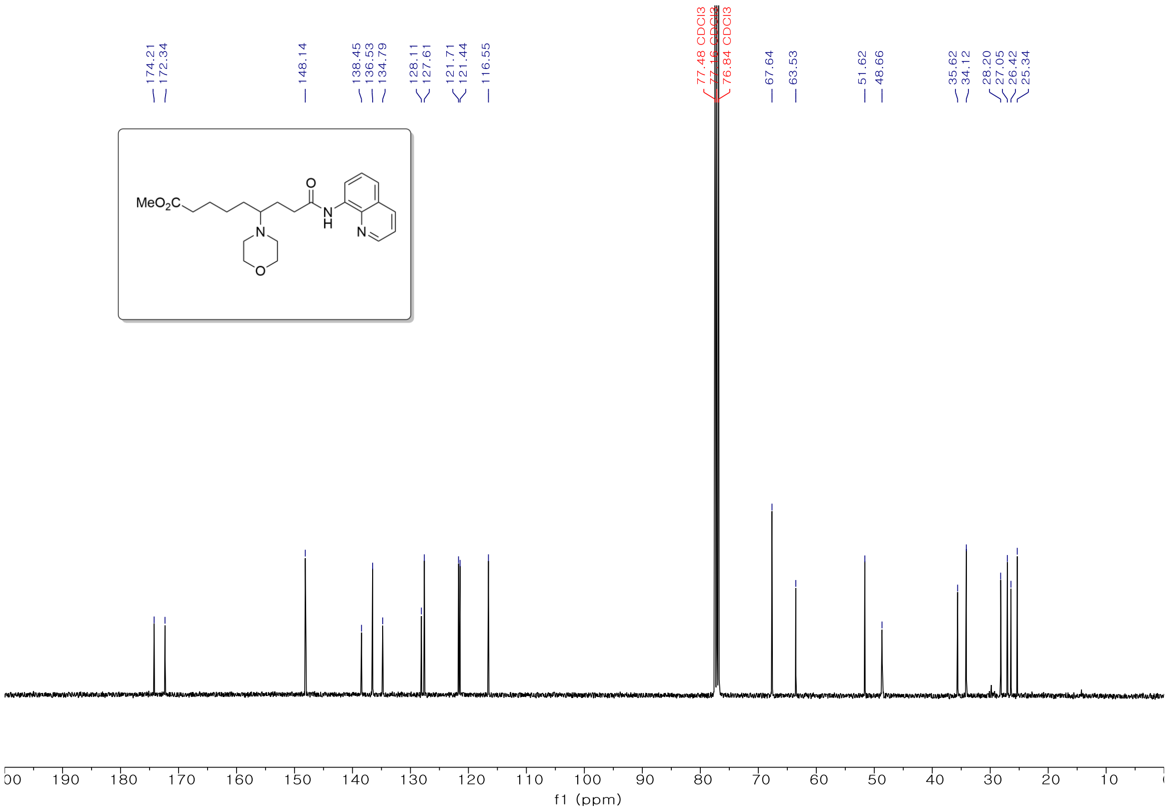

Supplementary Figure 21. ^{1}H and ^{13}C NMR of **3h**

^{1}H NMR 400 MHz, CD_2Cl_2

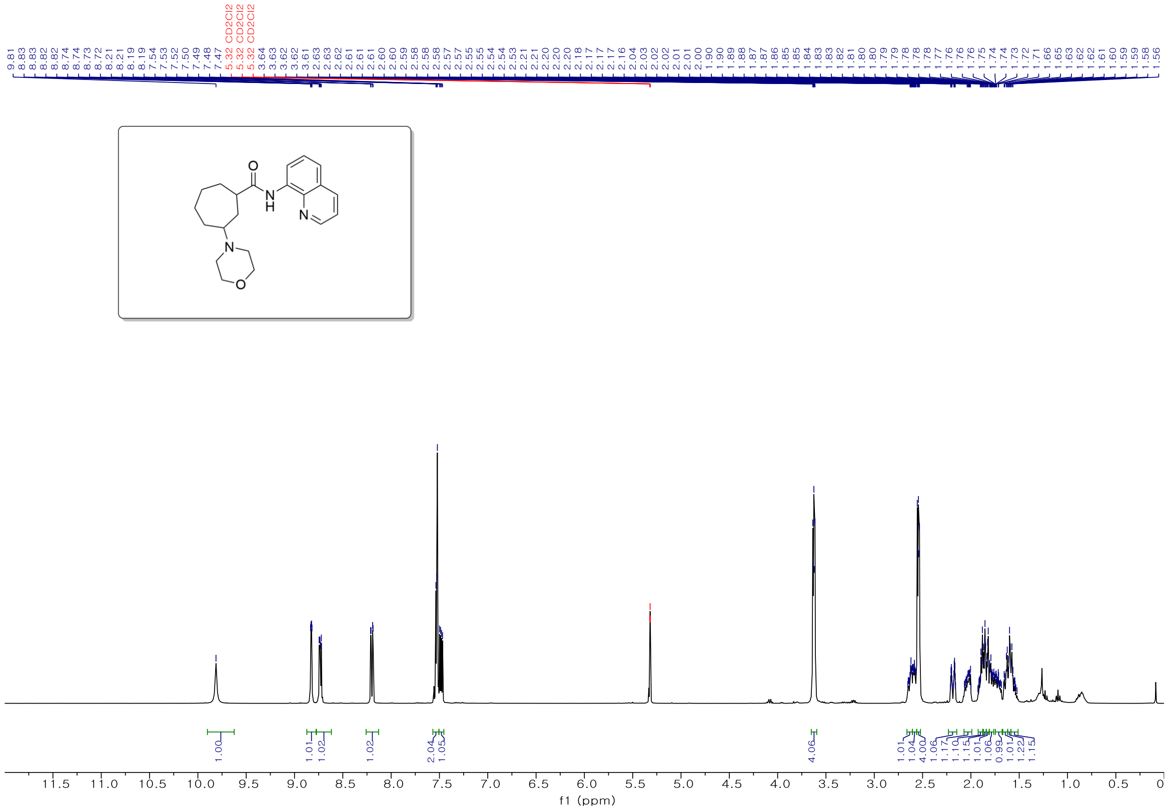

^{13}C NMR 100 MHz, CD_2Cl_2

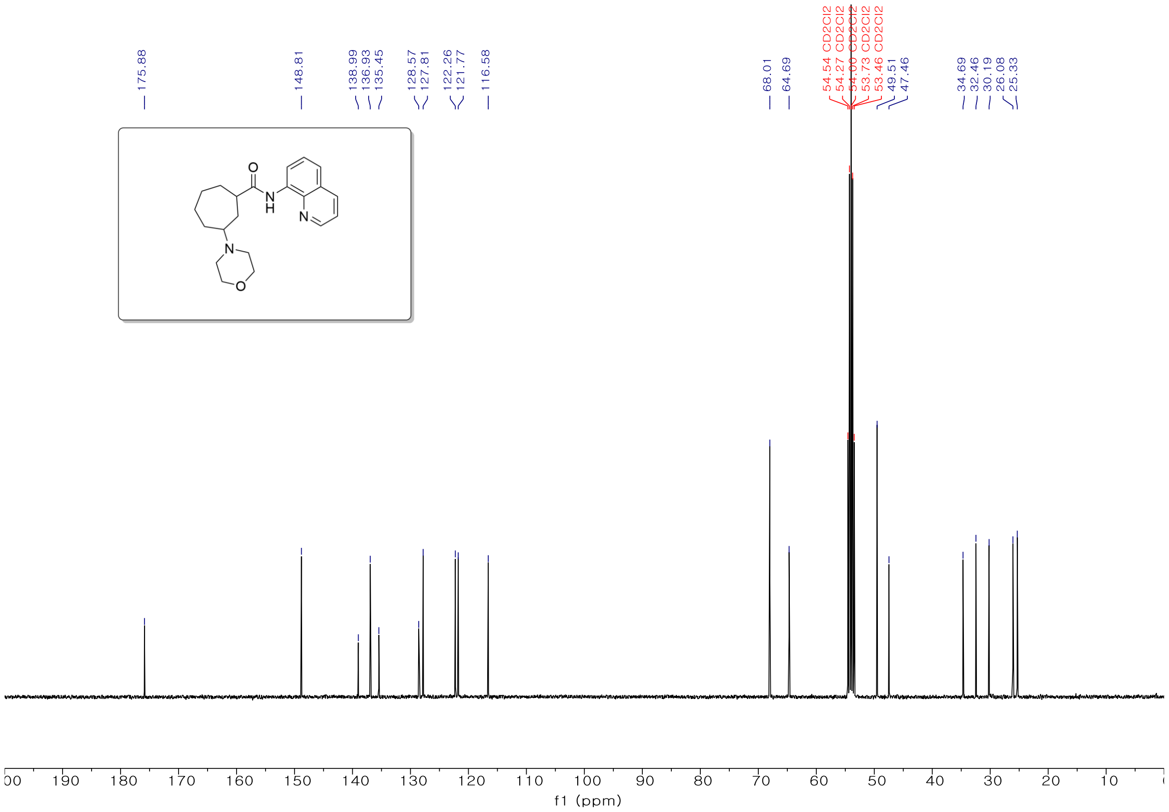

Supplementary Figure 22. ^{1}H and ^{13}C NMR of **3i**

^{1}H NMR 600 MHz, $CDCl_3$

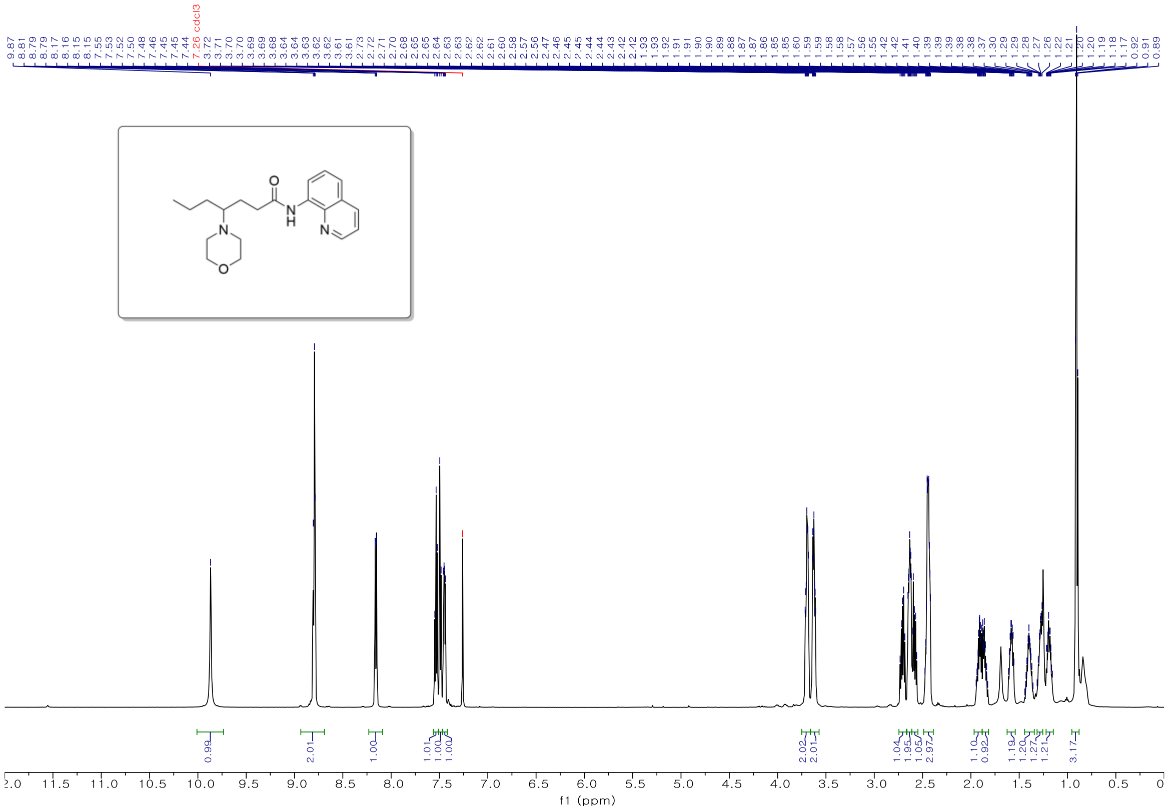

^{13}C NMR 150 MHz, $CDCl_3$

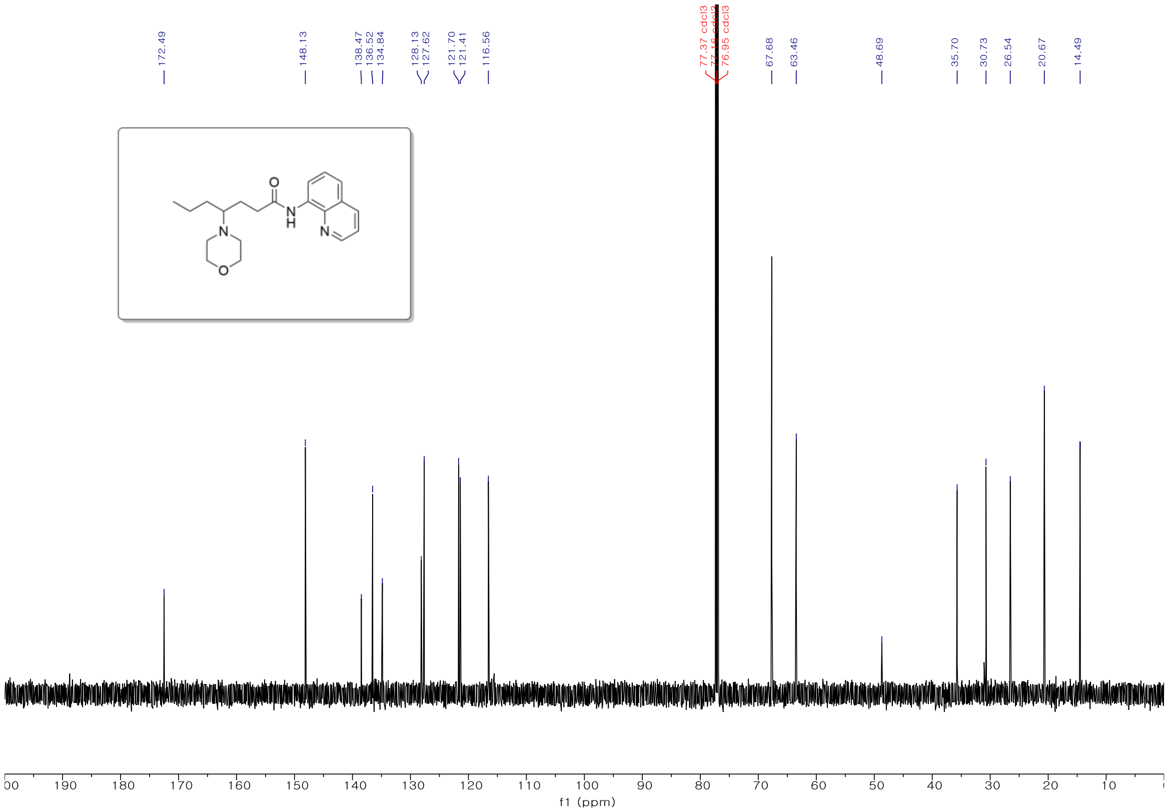

Supplementary Figure 23. ^{1}H and ^{13}C NMR of **3j**

^{1}H NMR 400 MHz, $CDCl_3$

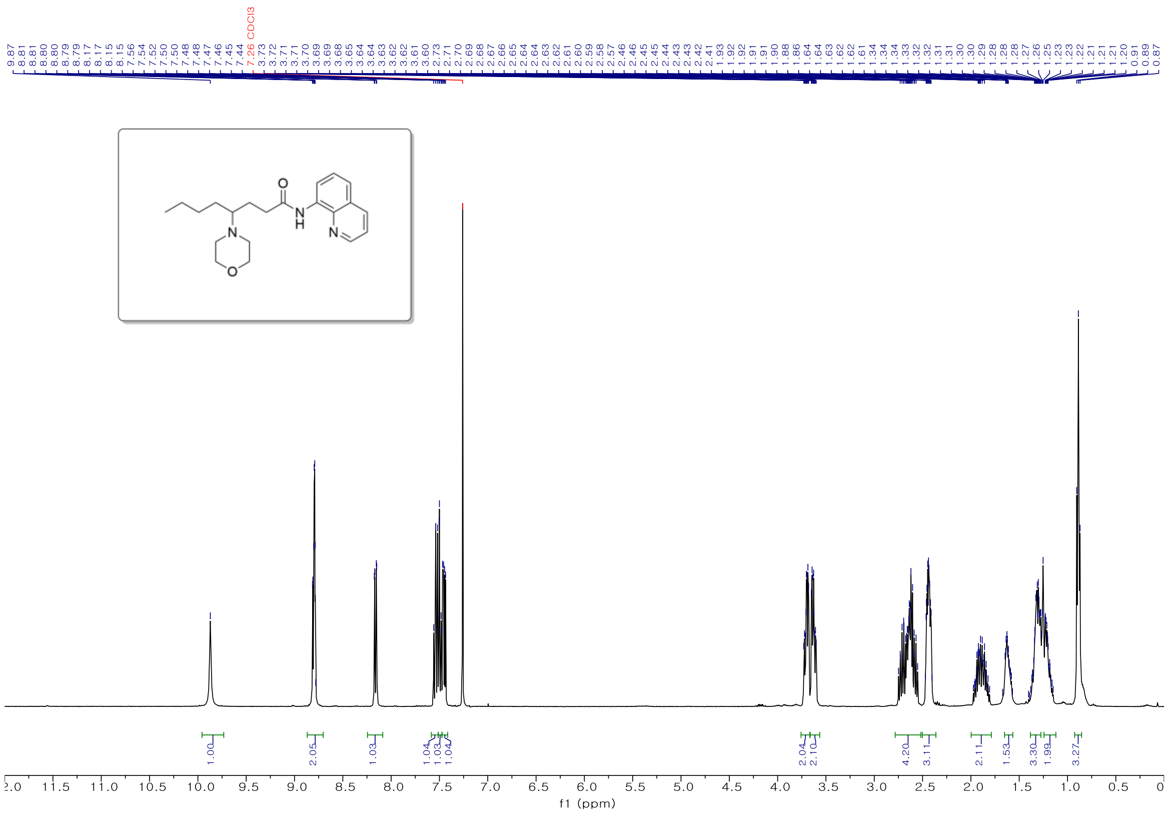

^{13}C NMR 100 MHz, $CDCl_3$

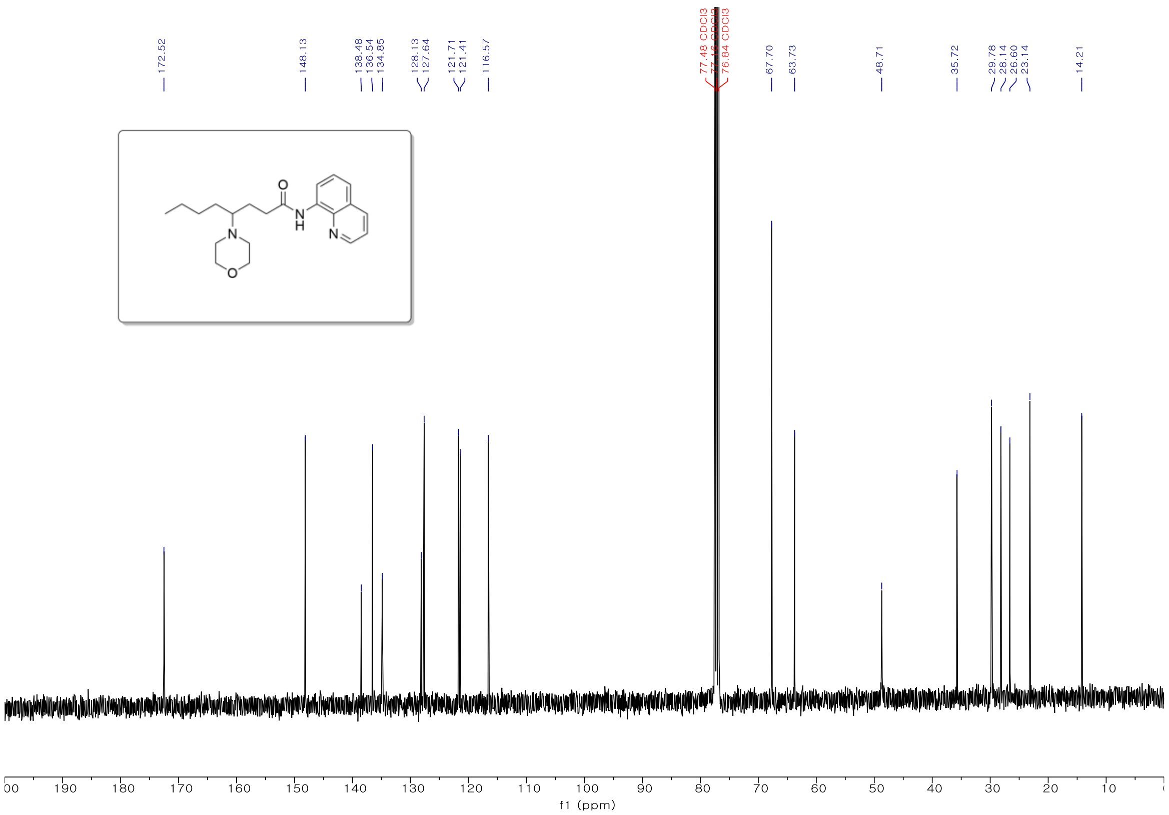

Supplementary Figure 24. ^{1}H and ^{13}C NMR of **3k**

^{1}H NMR 400 MHz, CDCl$_3$

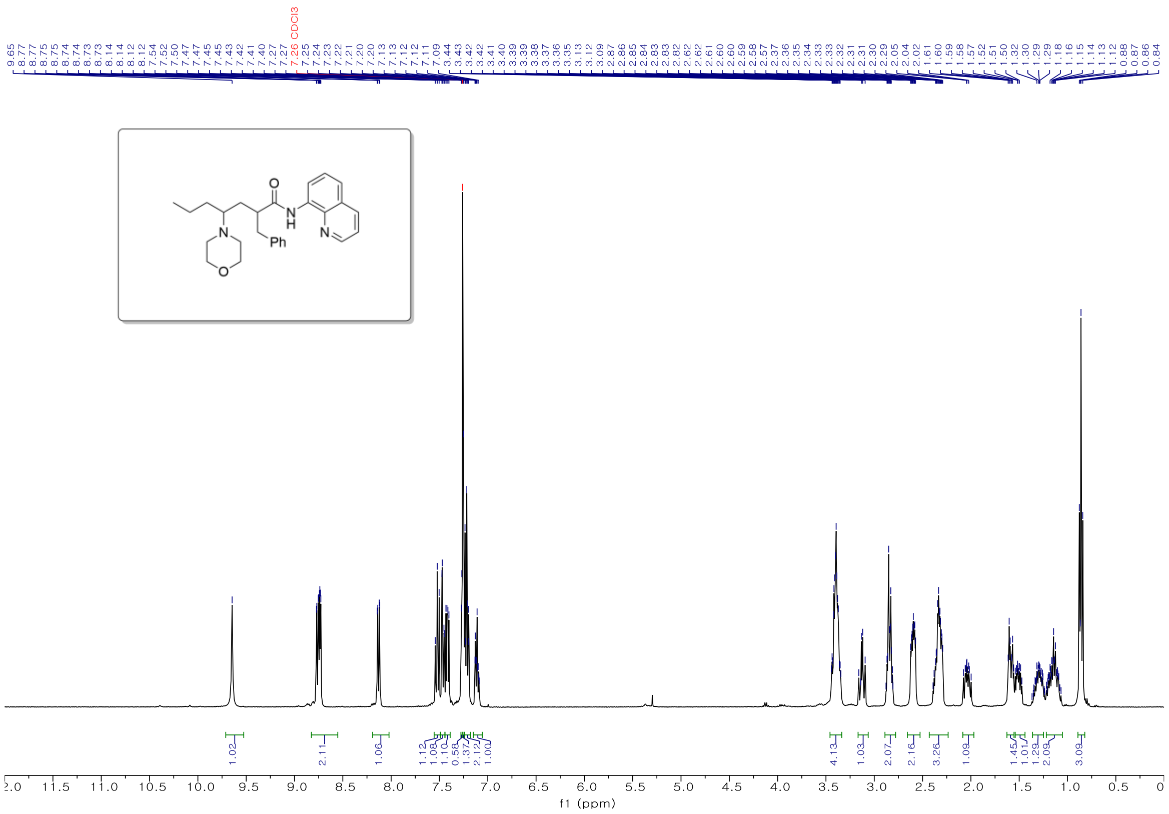

^{13}C NMR 100 MHz, CDCl$_3$

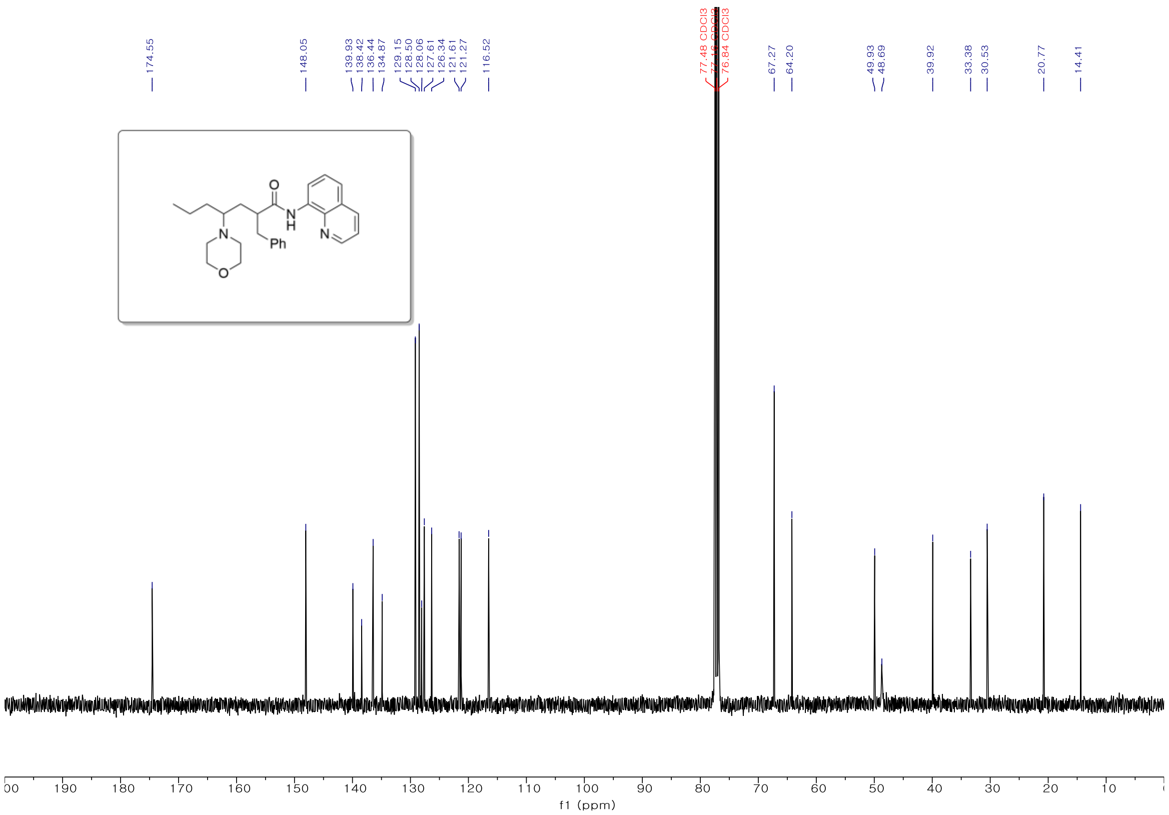

Supplementary Figure 25. ^{1}H and ^{13}C NMR of **3l** (diastereomer 4.4 : 1)

^{1}H NMR 400 MHz, $CDCl_3$

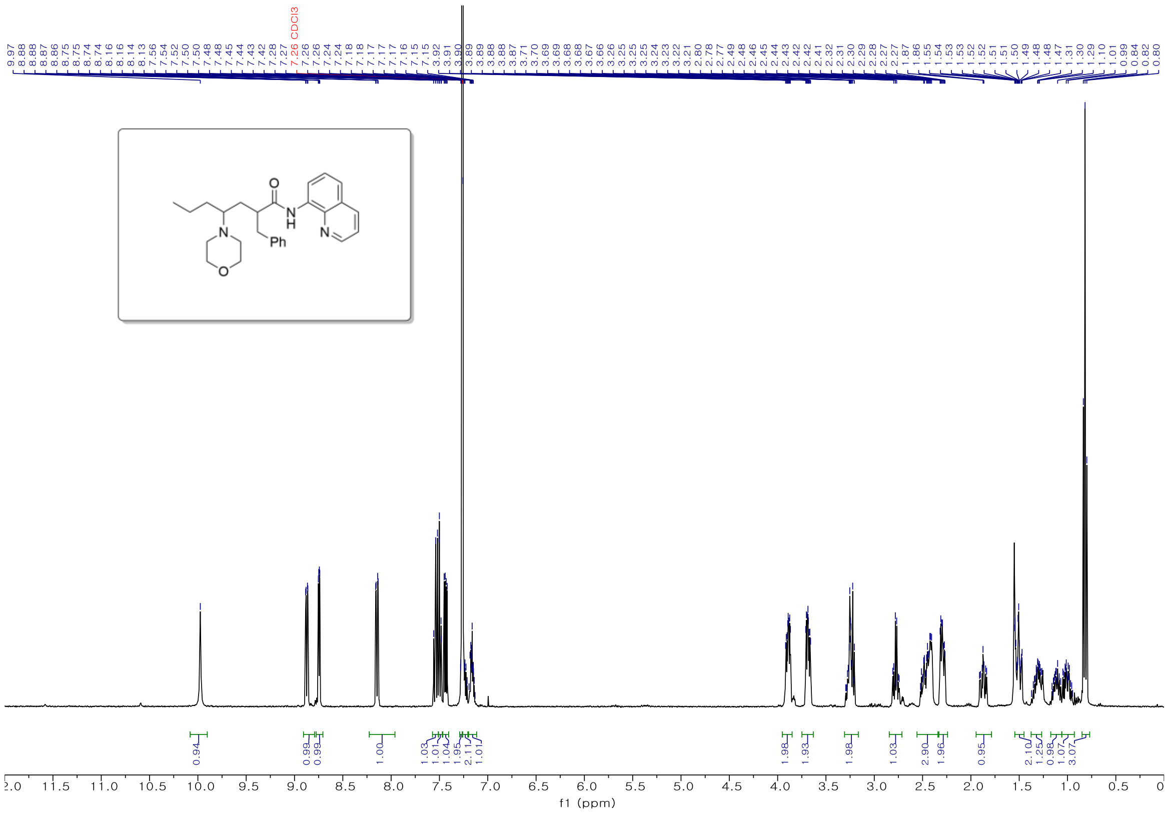

^{13}C NMR 100 MHz, $CDCl_3$

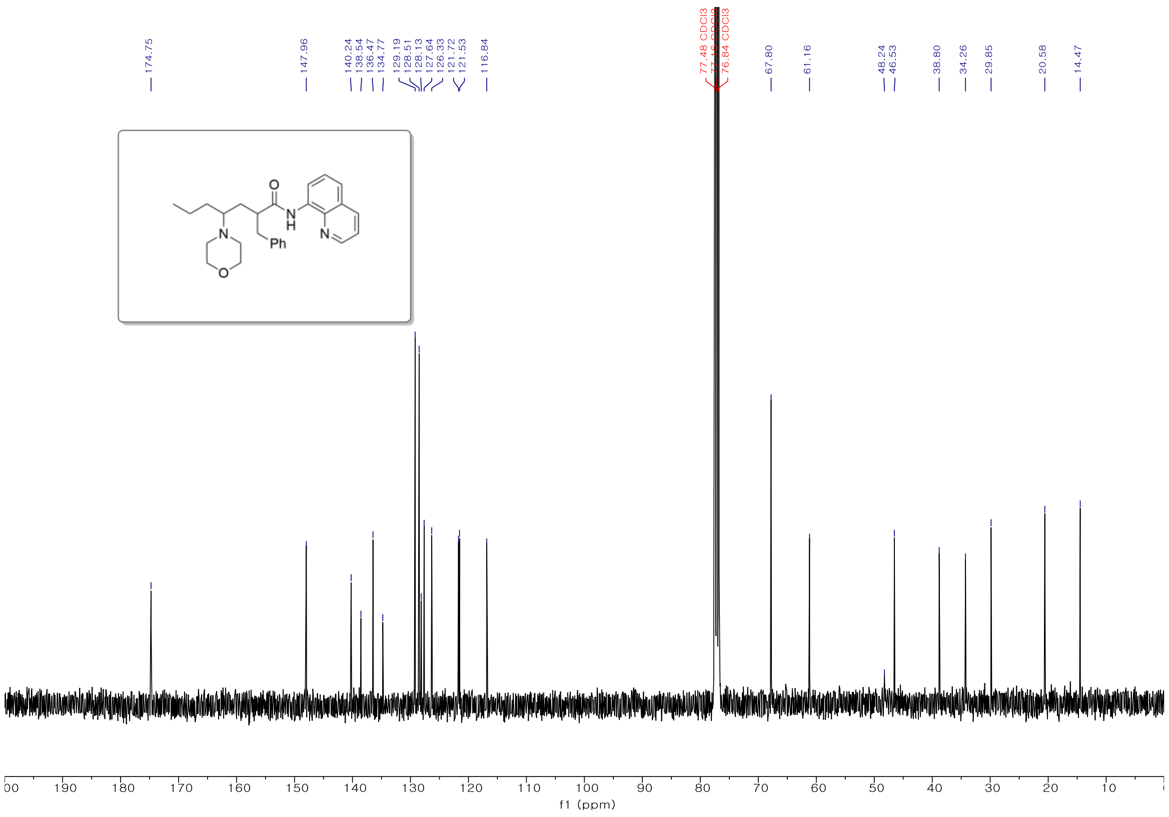

Supplementary Figure 26. ^{1}H and ^{13}C NMR of **3l’** (diastereomer 4.4 : 1)

^{1}H NMR 400 MHz, CDCl$_3$

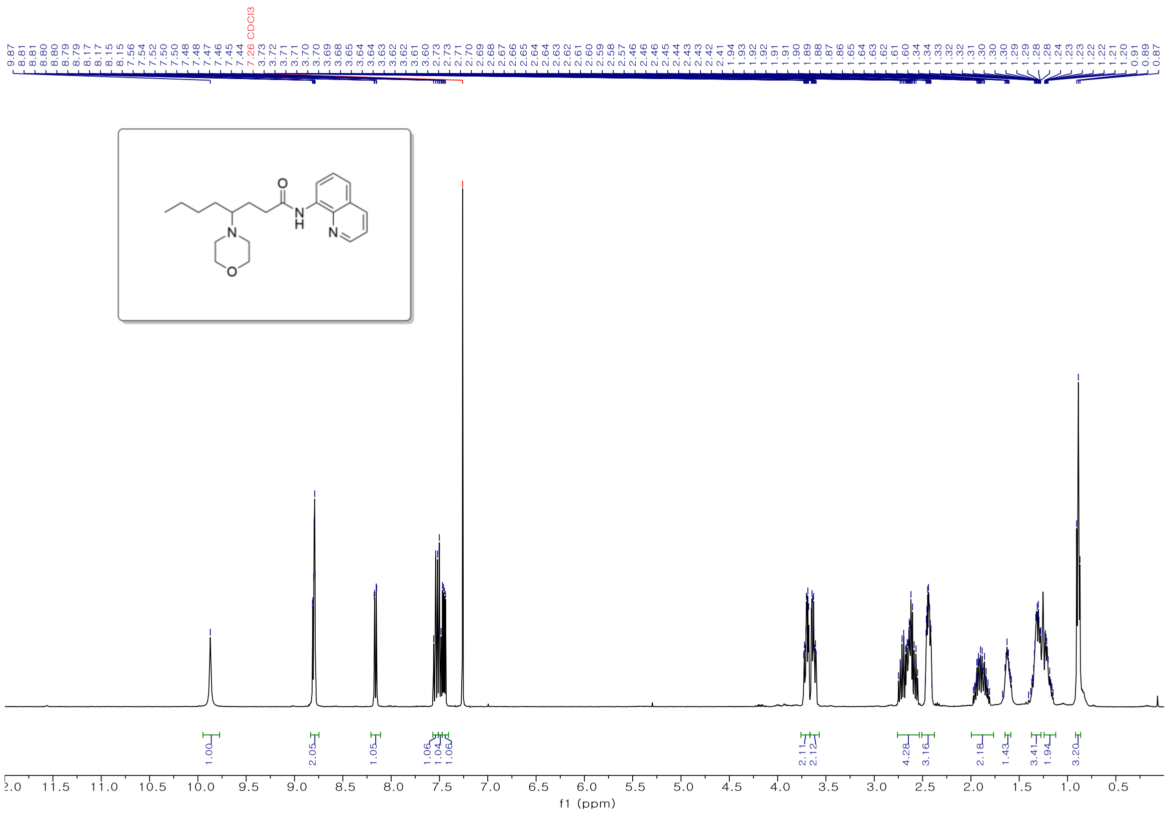

^{13}C NMR 100 MHz, CDCl$_3$

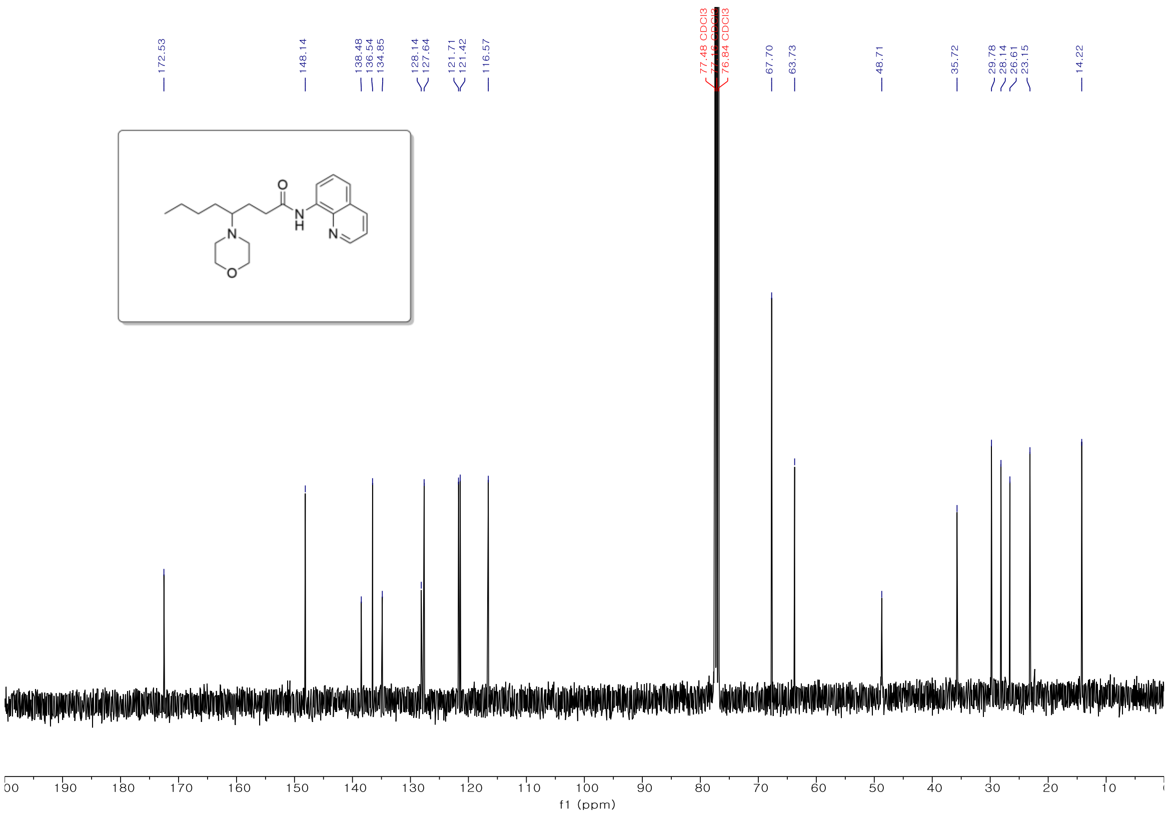

Supplementary Figure 27. ^{1}H and ^{13}C NMR of **3m**

^{1}H NMR 400 MHz, CD_2Cl_2

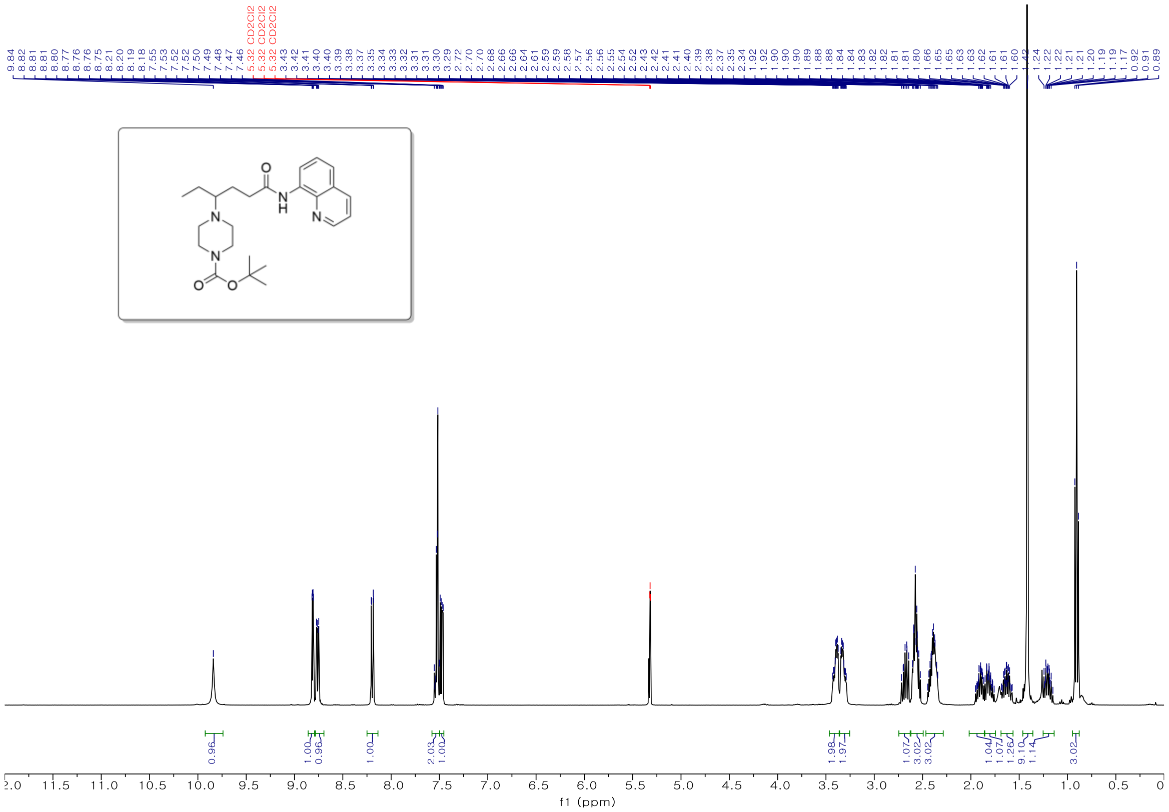

^{13}C NMR 100 MHz, CD_2Cl_2

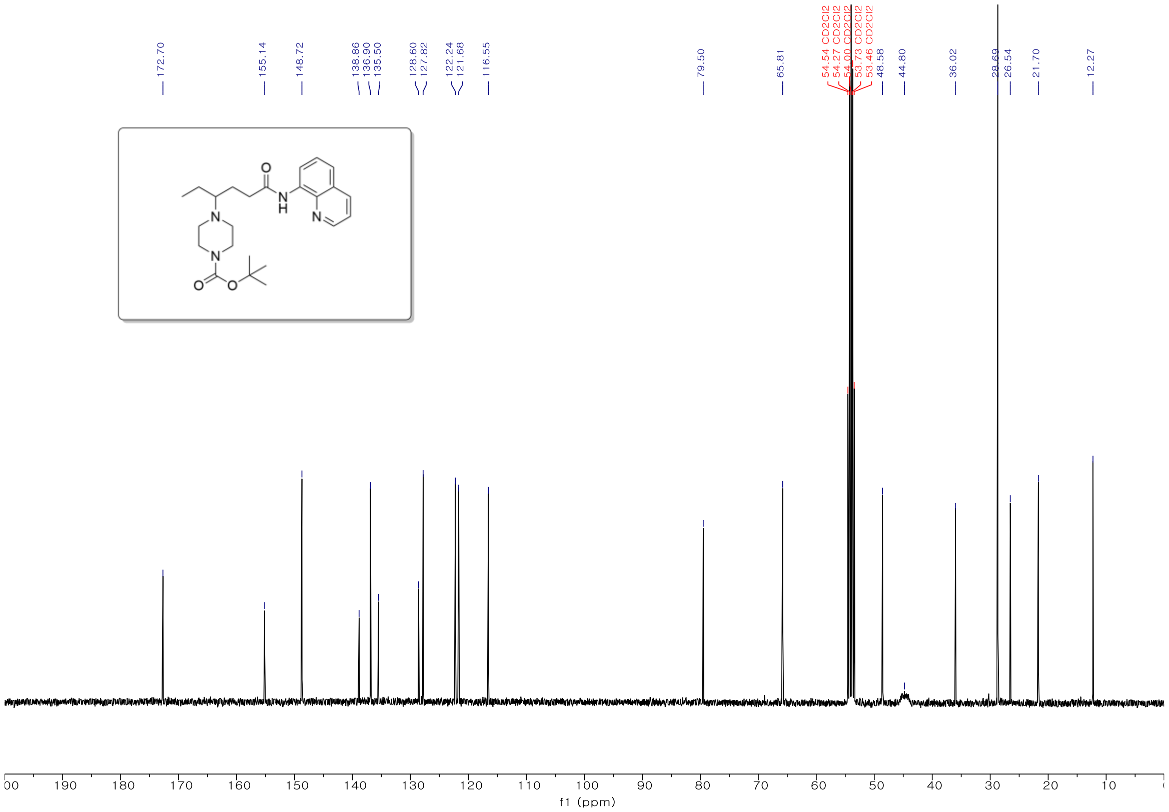

Supplementary Figure 28. ^{1}H and ^{13}C NMR of **4a**

^{1}H NMR 400 MHz, CDCl$_3$

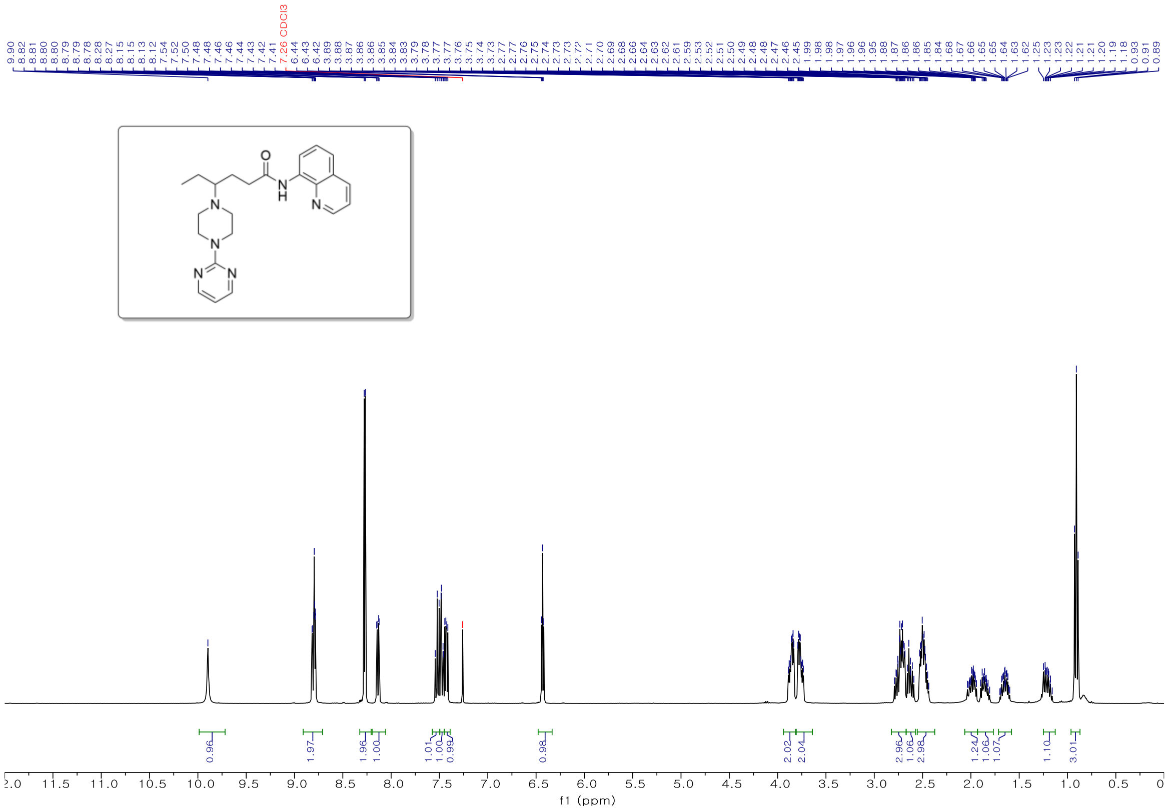

^{13}C NMR 100 MHz, CDCl$_3$

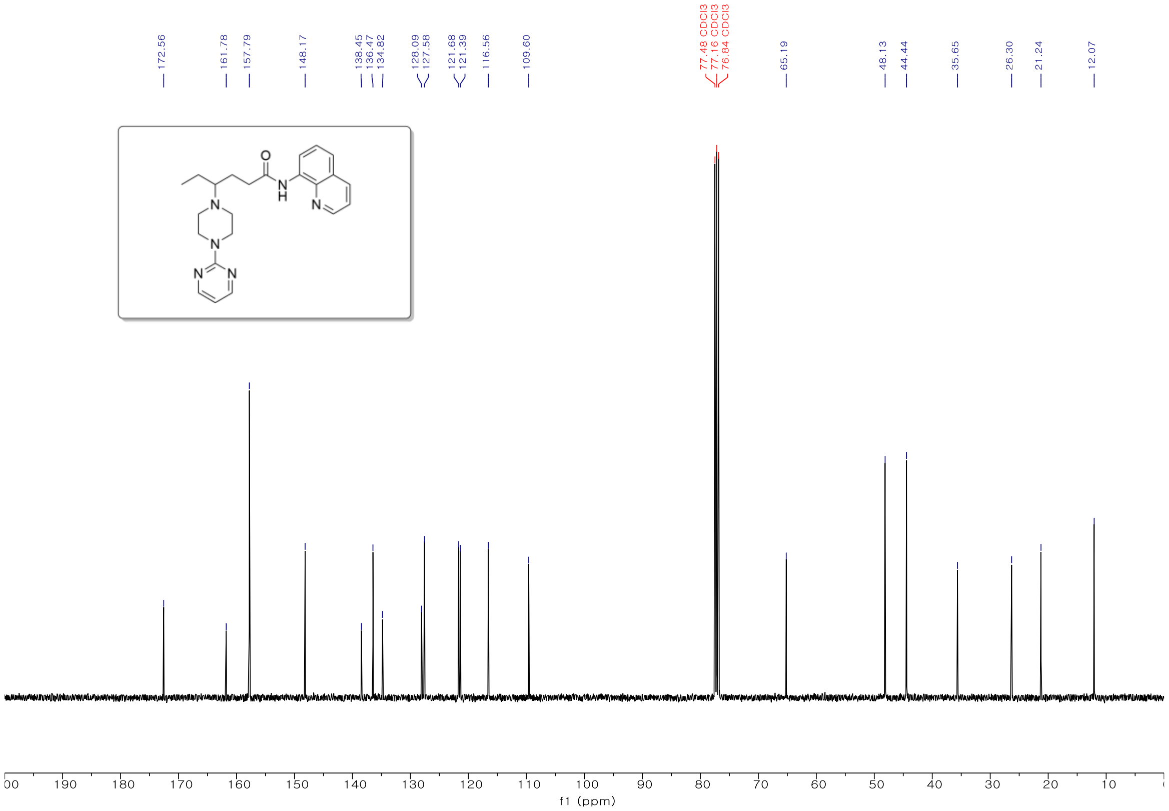

Supplementary Figure 29. ^{1}H and ^{13}C NMR of **4b**

^{1}H NMR 400 MHz, $CDCl_3$

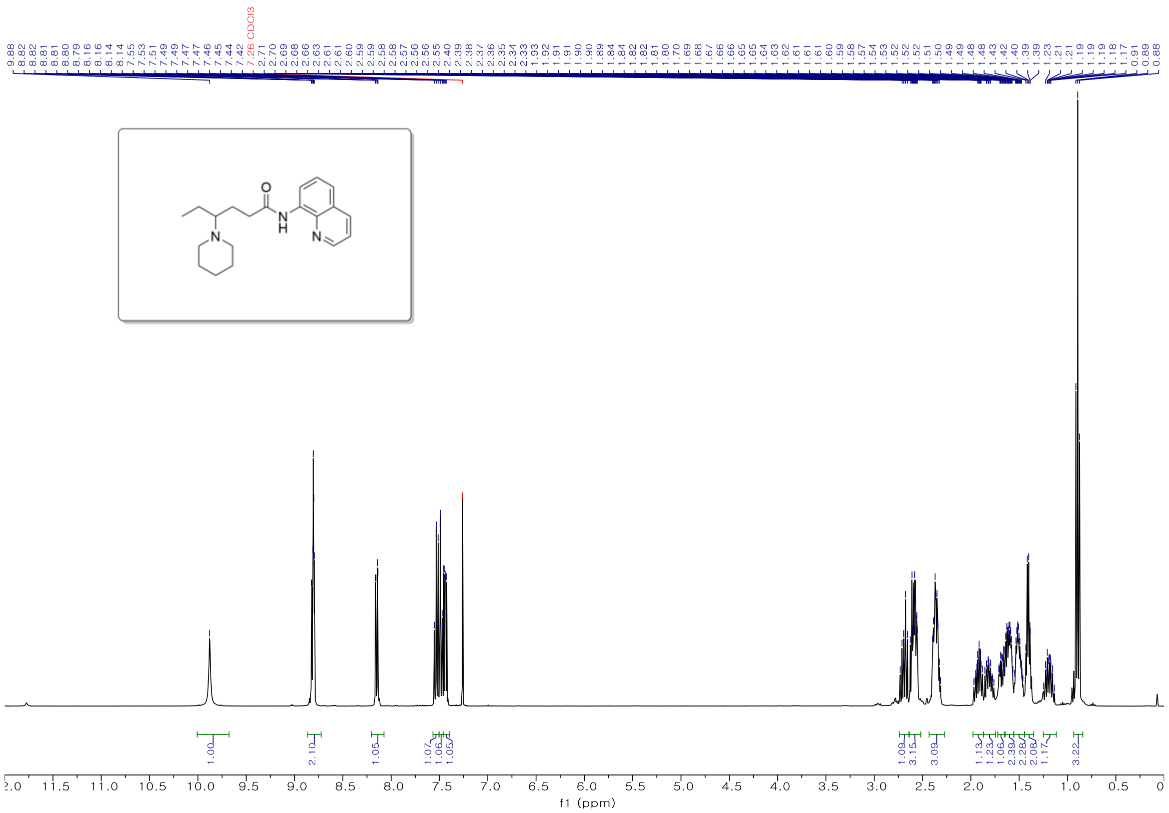

^{13}C NMR 100 MHz, $CDCl_3$

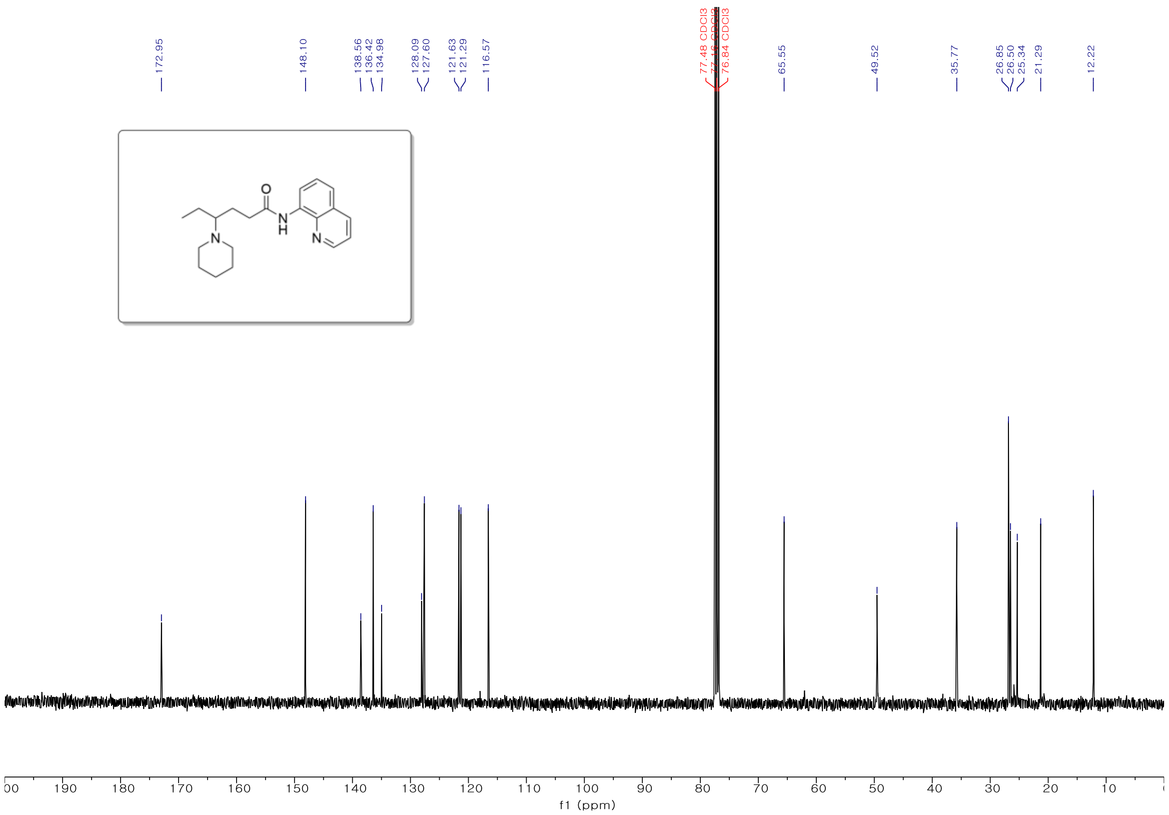

Supplementary Figure 30. ^{1}H and ^{13}C NMR of **4c**

^{1}H NMR 400 MHz, CDCl$_3$

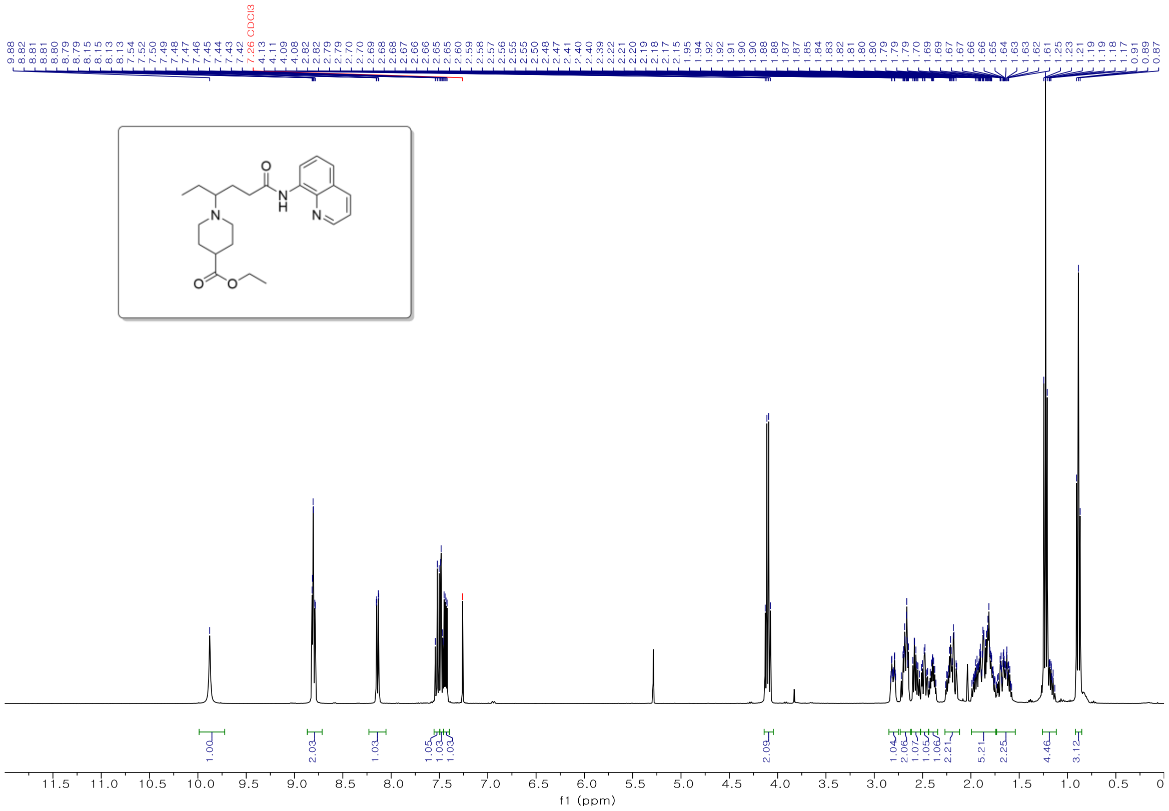

^{13}C NMR 100 MHz, CDCl$_3$

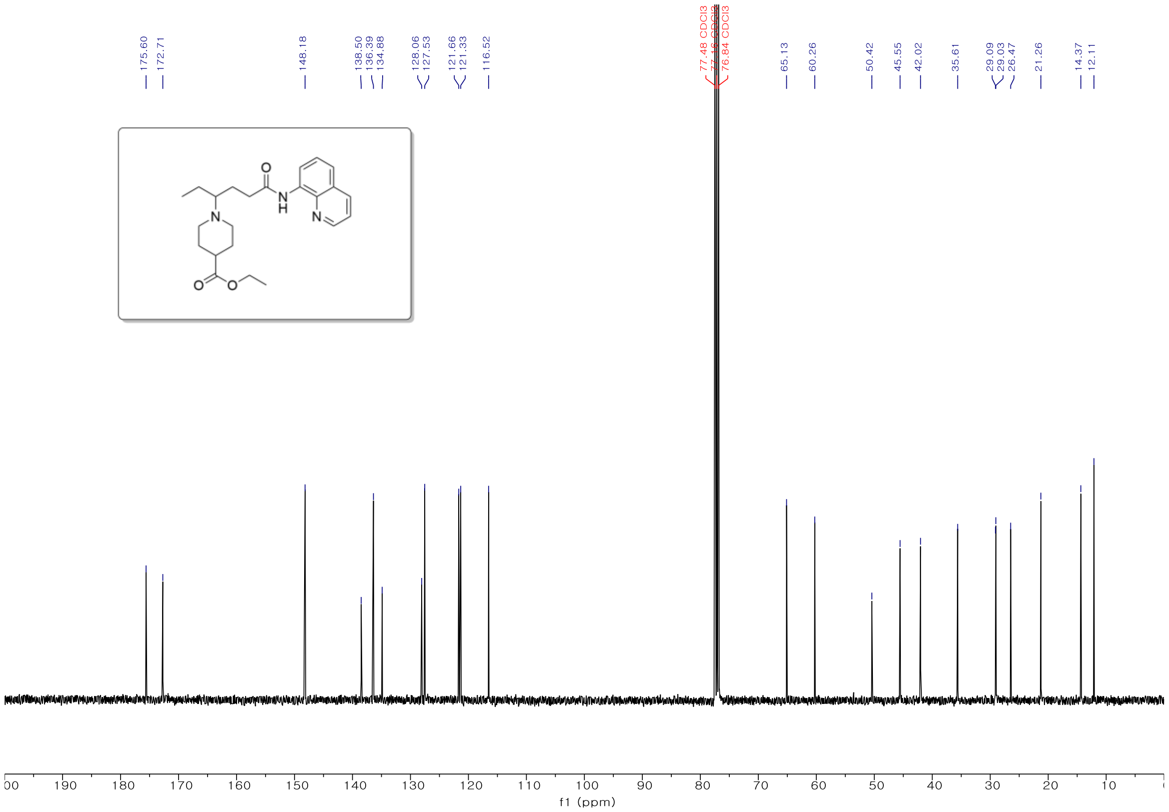

Supplementary Figure 31. ^{1}H and ^{13}C NMR of **4d**

^{1}H NMR 400 MHz, $CDCl_3$

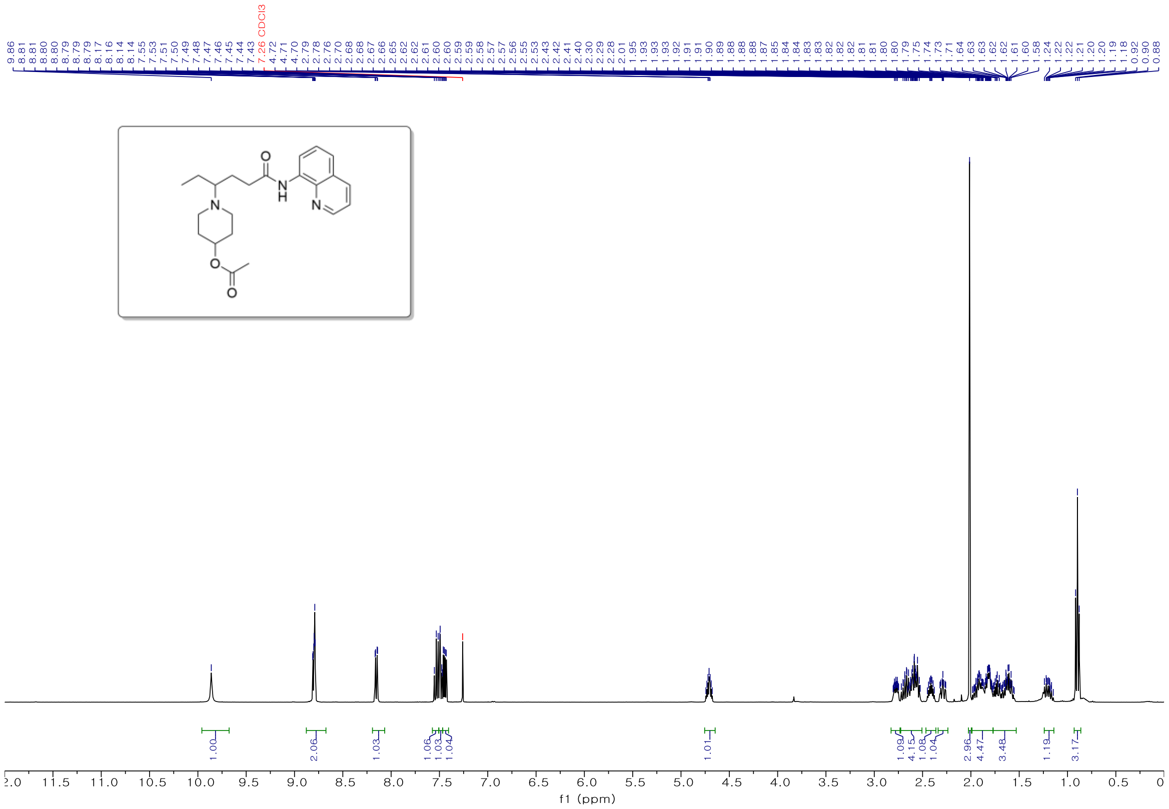

^{13}C NMR 100 MHz, $CDCl_3$

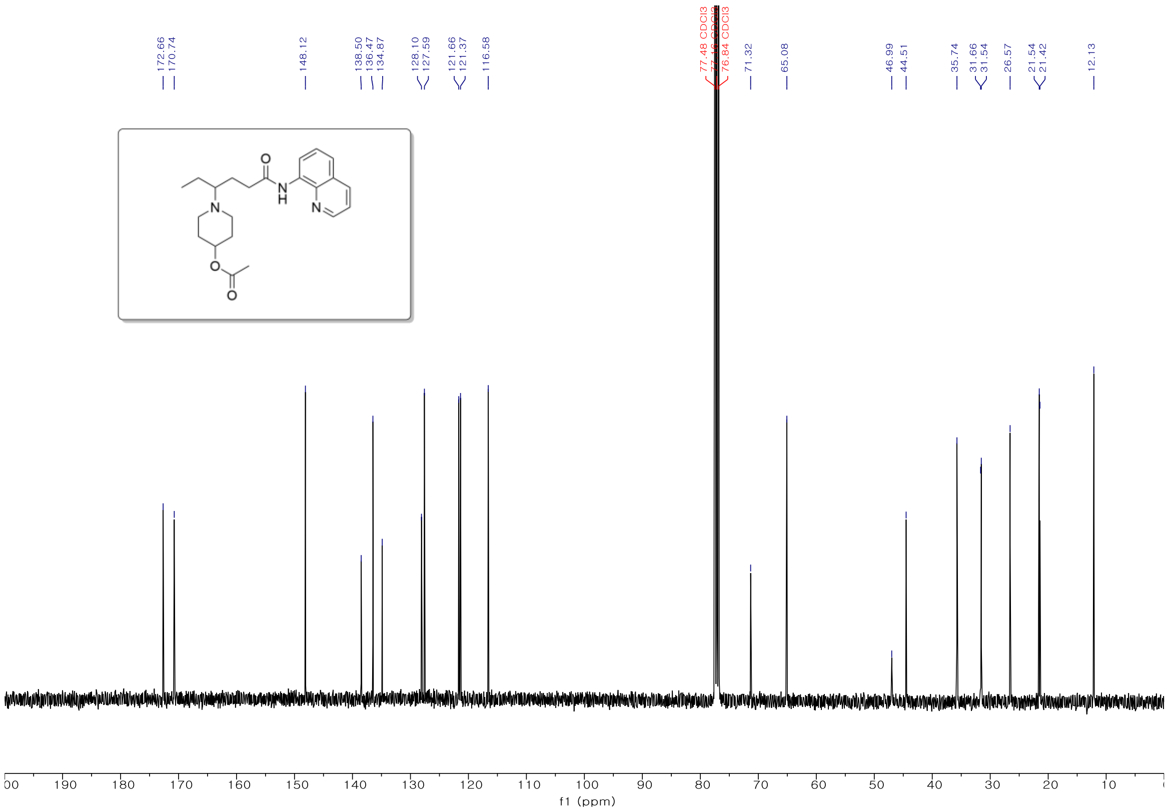

Supplementary Figure 32. ^{1}H and ^{13}C NMR of **4e**

^{1}H NMR 400 MHz, CDCl$_3$

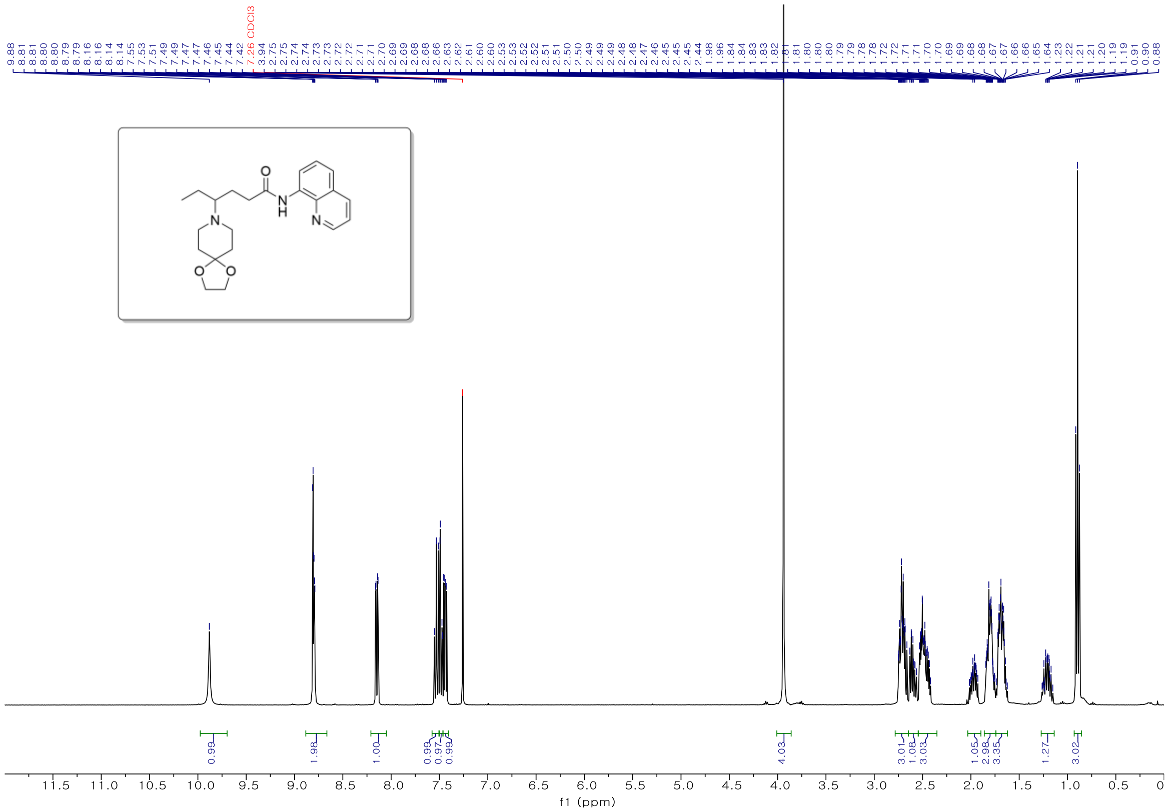

^{13}C NMR 100 MHz, CDCl$_3$

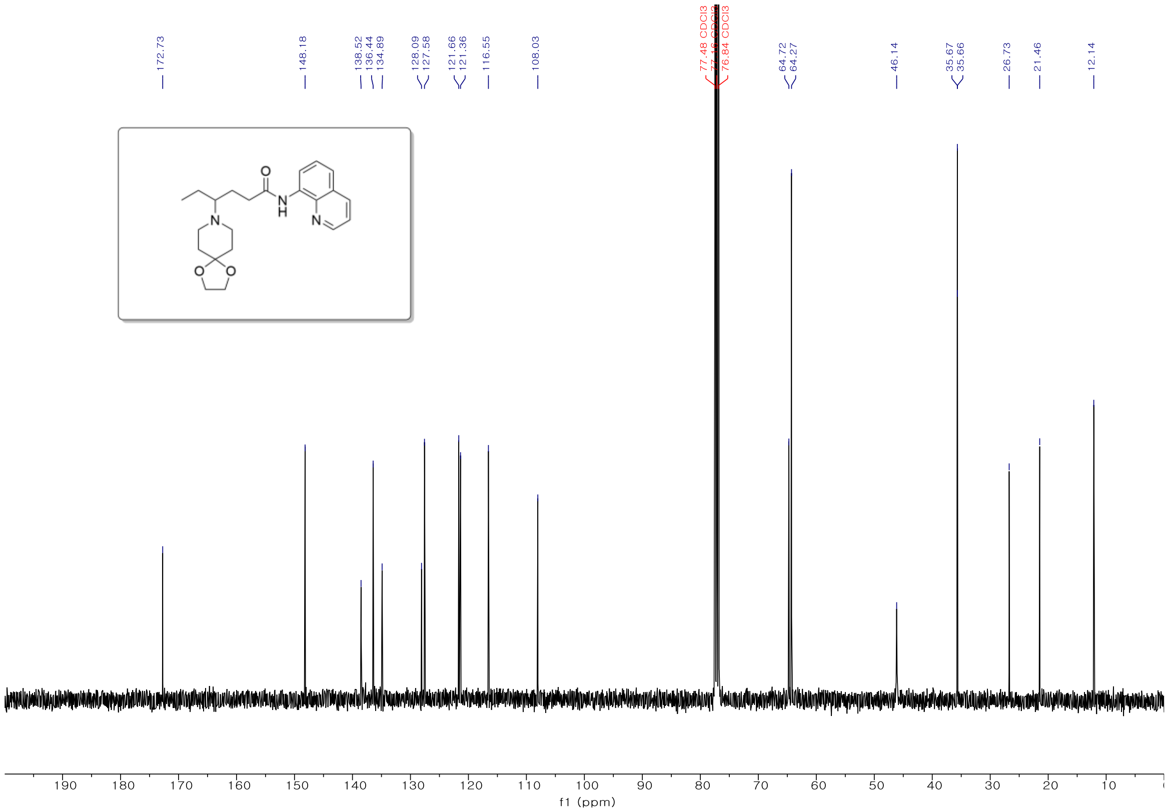

Supplementary Figure 33. ^{1}H and ^{13}C NMR of **4f**

^{1}H NMR 400 MHz, $CDCl_3$

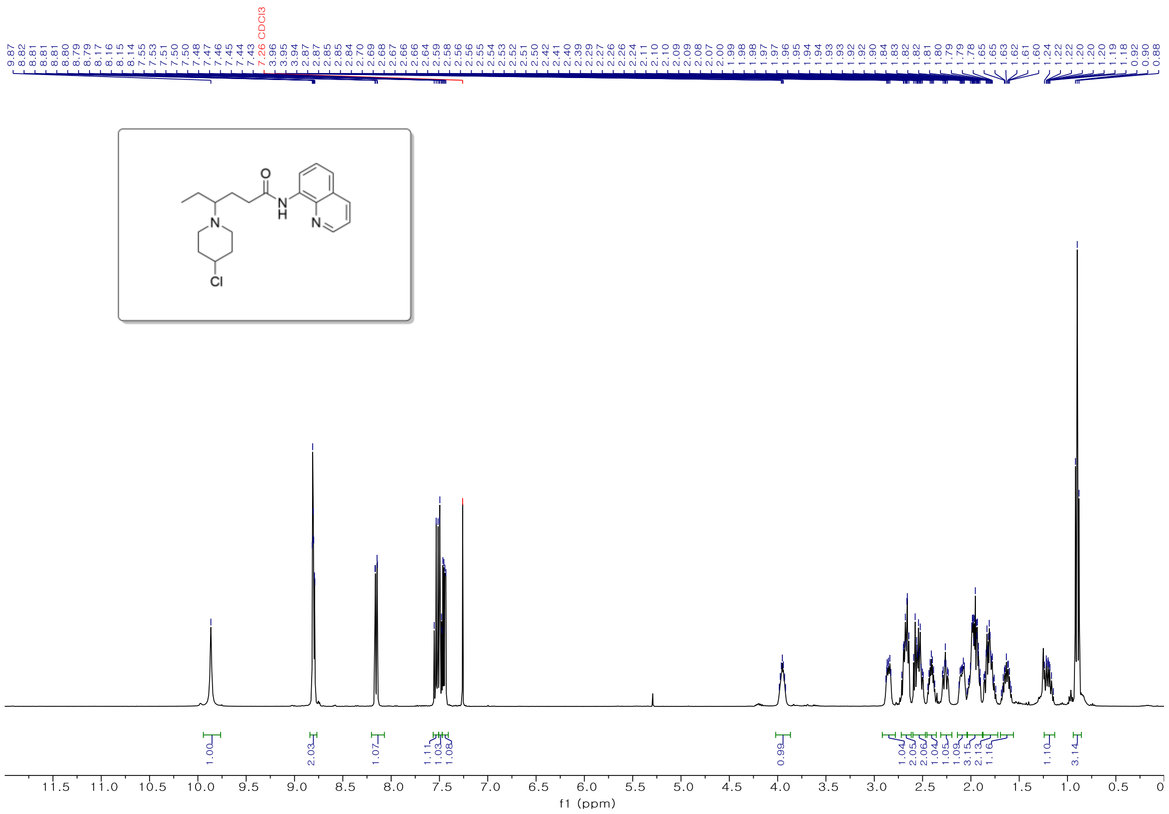

^{13}C NMR 100 MHz, $CDCl_3$

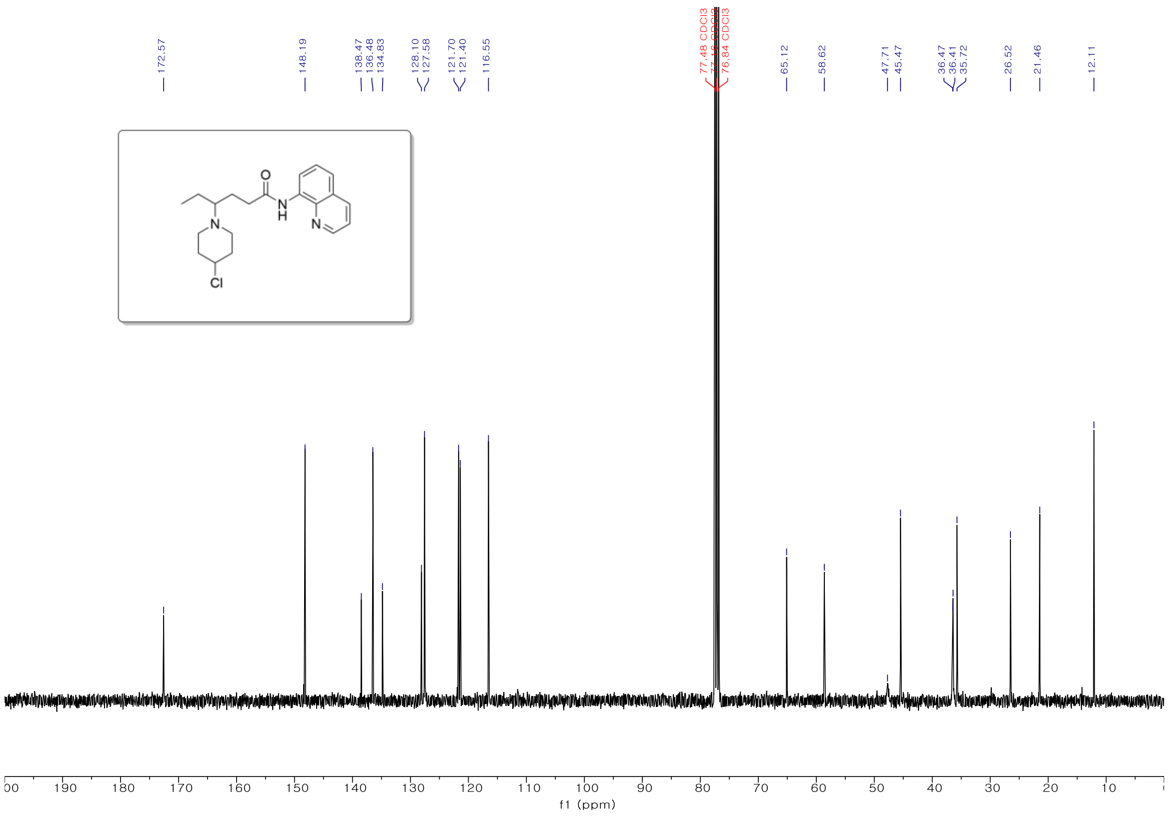

Supplementary Figure 34. ^{1}H and ^{13}C NMR of **4g**

^{1}H NMR 400 MHz, $CDCl_3$

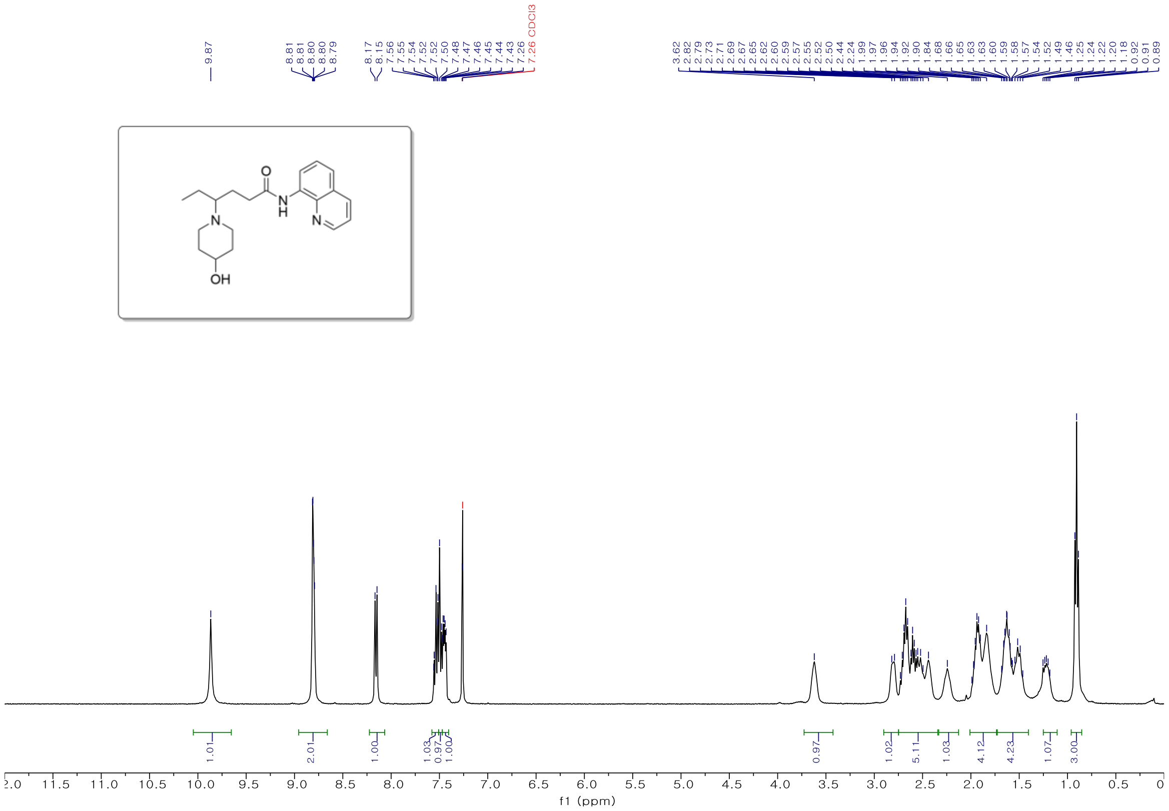

^{13}C NMR 100 MHz, $CDCl_3$

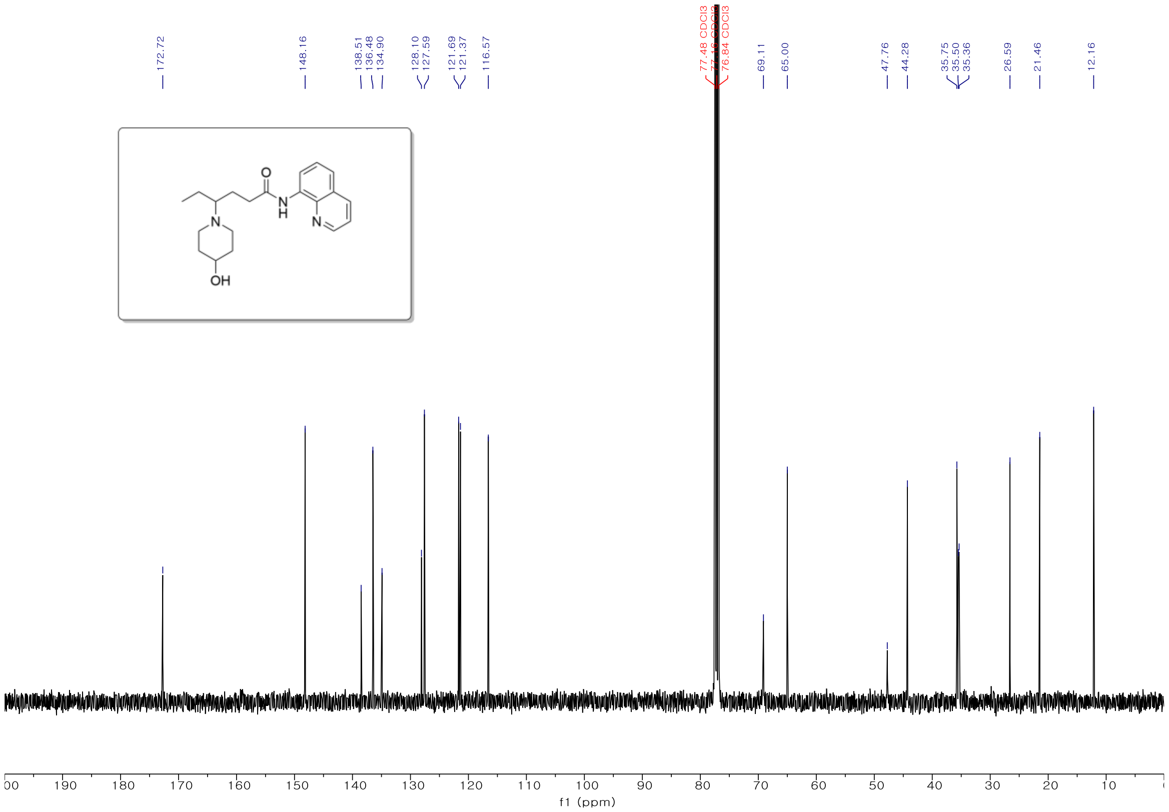

Supplementary Figure 35. ^{1}H and ^{13}C NMR of **4h**

^{1}H NMR 400 MHz, $CDCl_3$

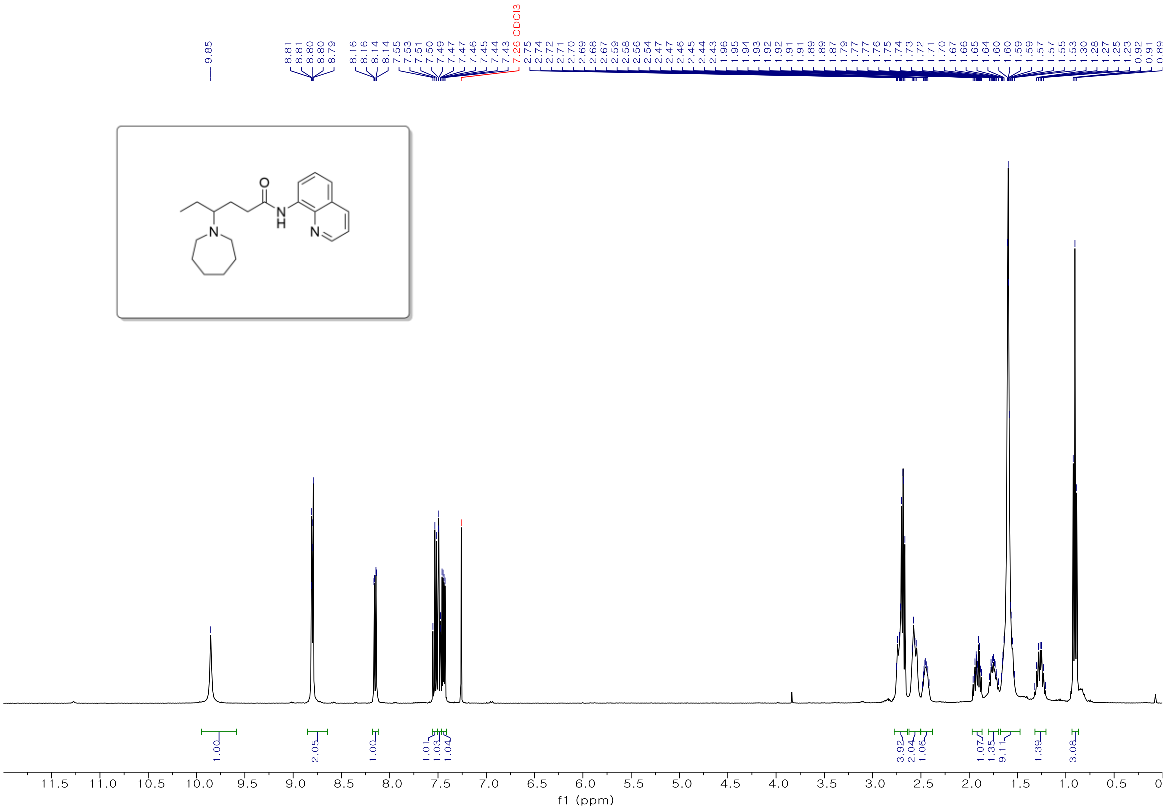

^{13}C NMR 100 MHz, $CDCl_3$

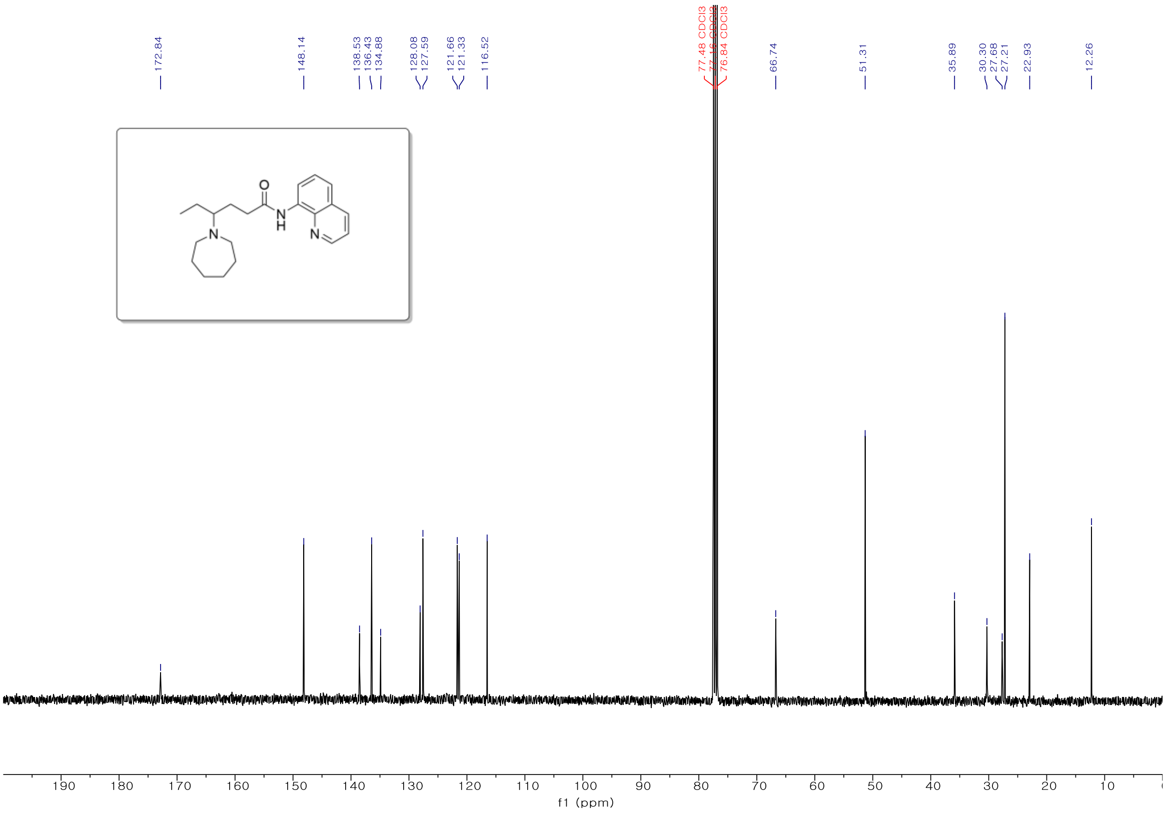

Supplementary Figure 36. ^{1}H and ^{13}C NMR of **4i**

^{1}H NMR 400 MHz, CDCl3

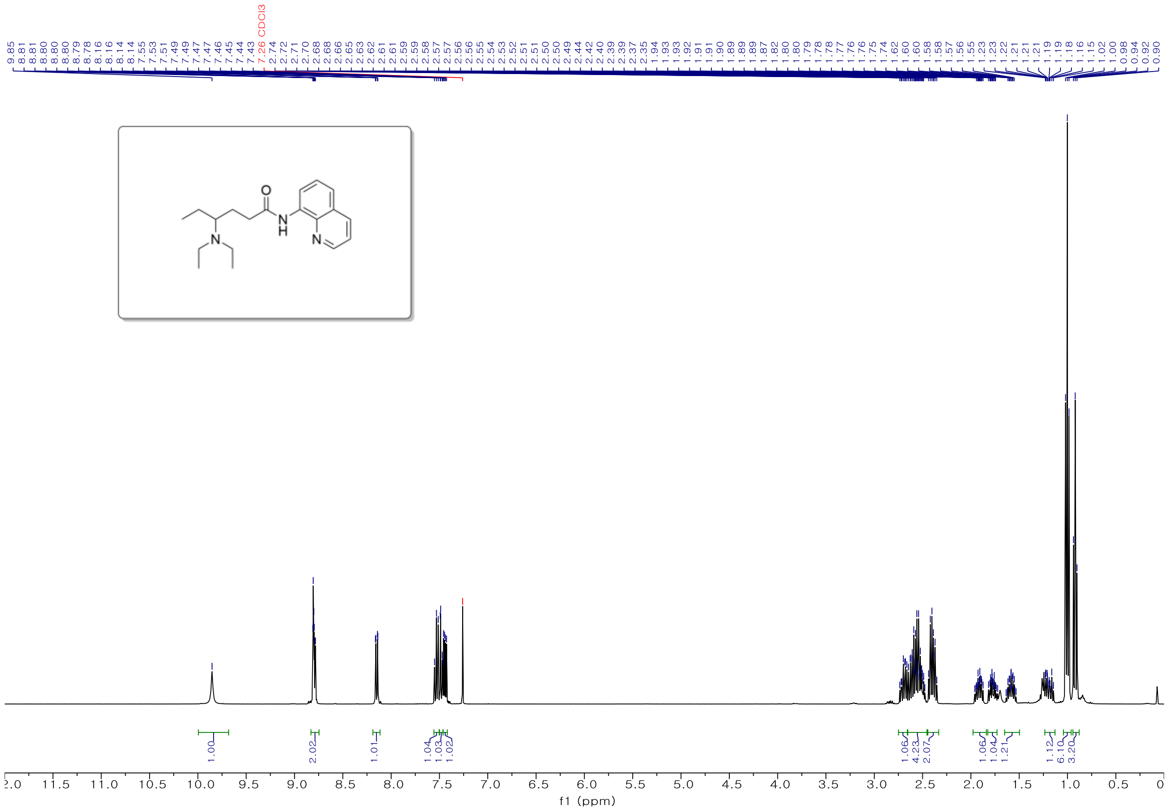

^{13}C NMR 100 MHz, CDCl3

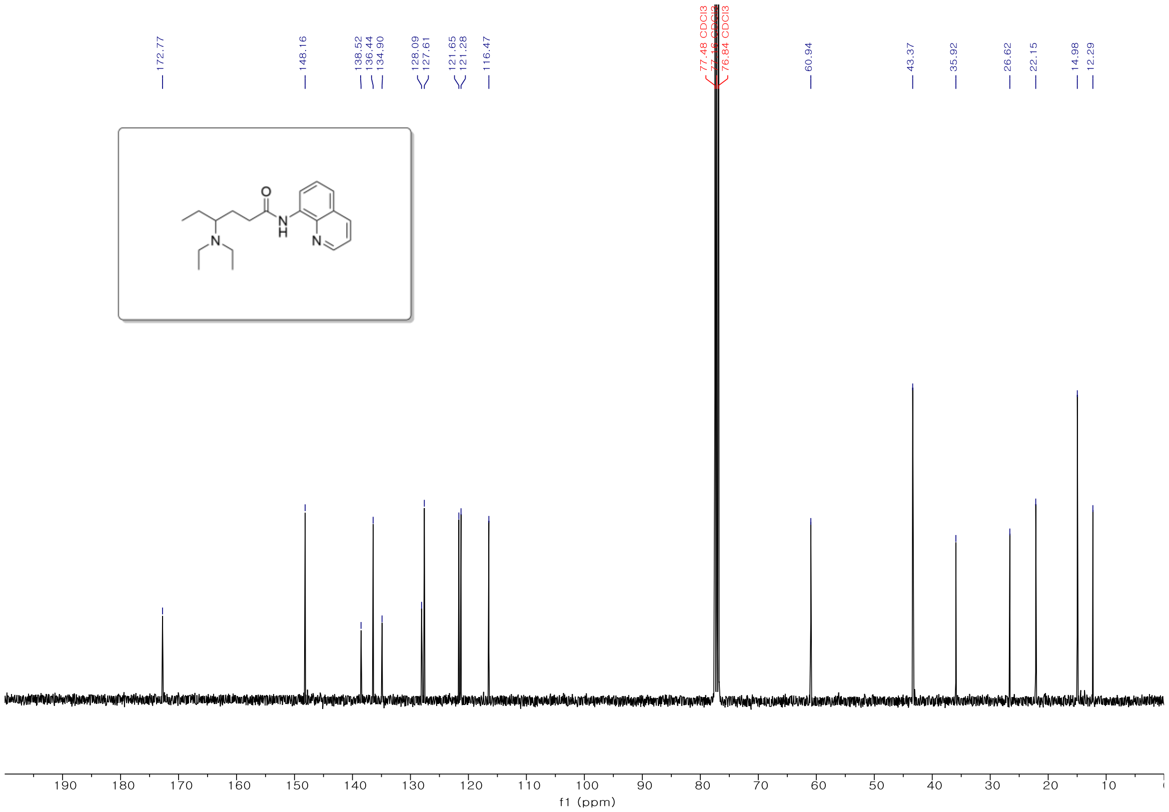

Supplementary Figure 37. ^{1}H and ^{13}C NMR of **4j**

^{1}H NMR 400 MHz, CDCl$_3$

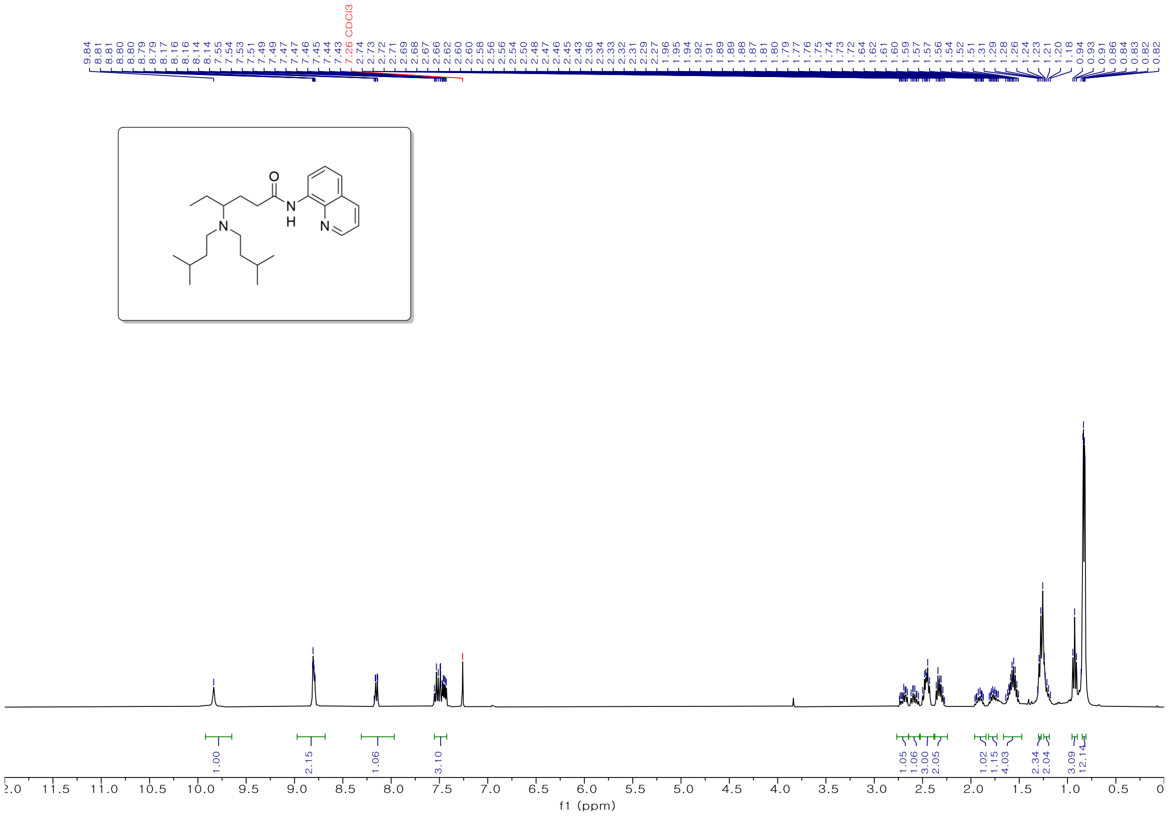

^{13}C NMR 100 MHz, CDCl$_3$

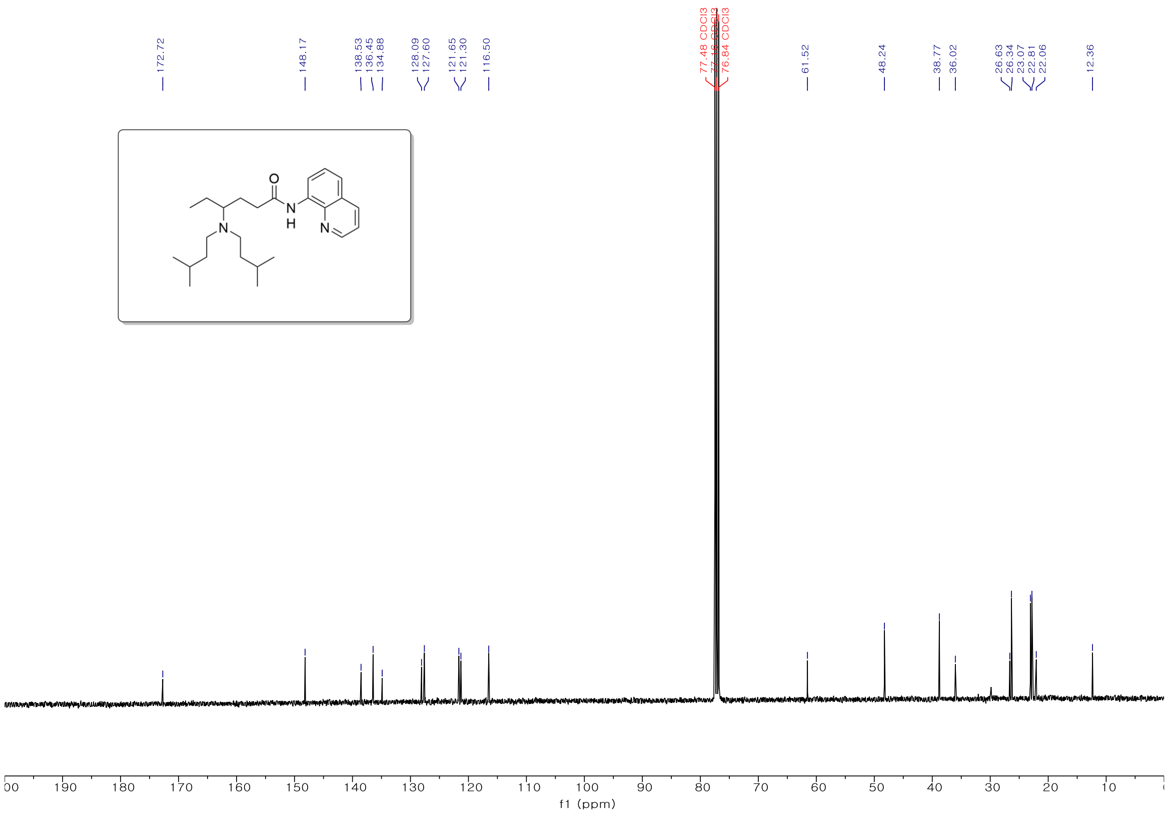

Supplementary Figure 38. ^{1}H and ^{13}C NMR of **4k**

^{1}H NMR 400 MHz, $CDCl_3$

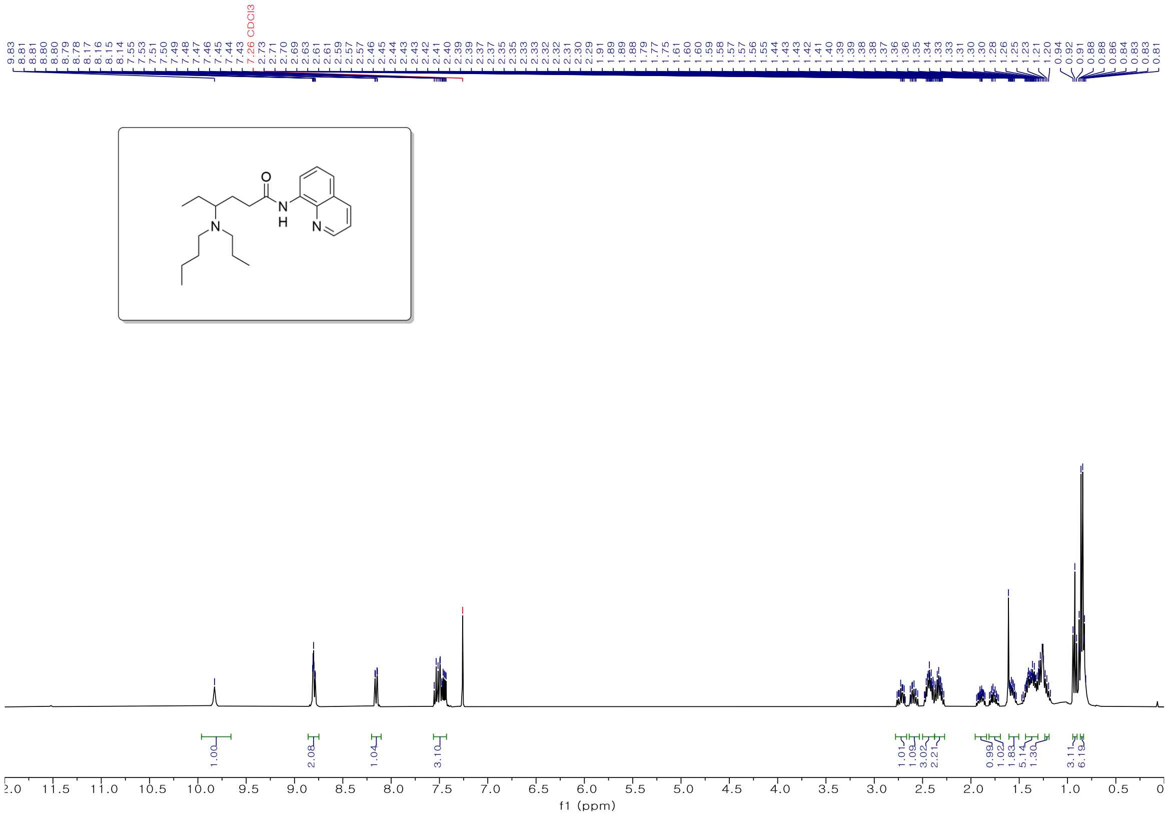

^{13}C NMR 100 MHz, $CDCl_3$

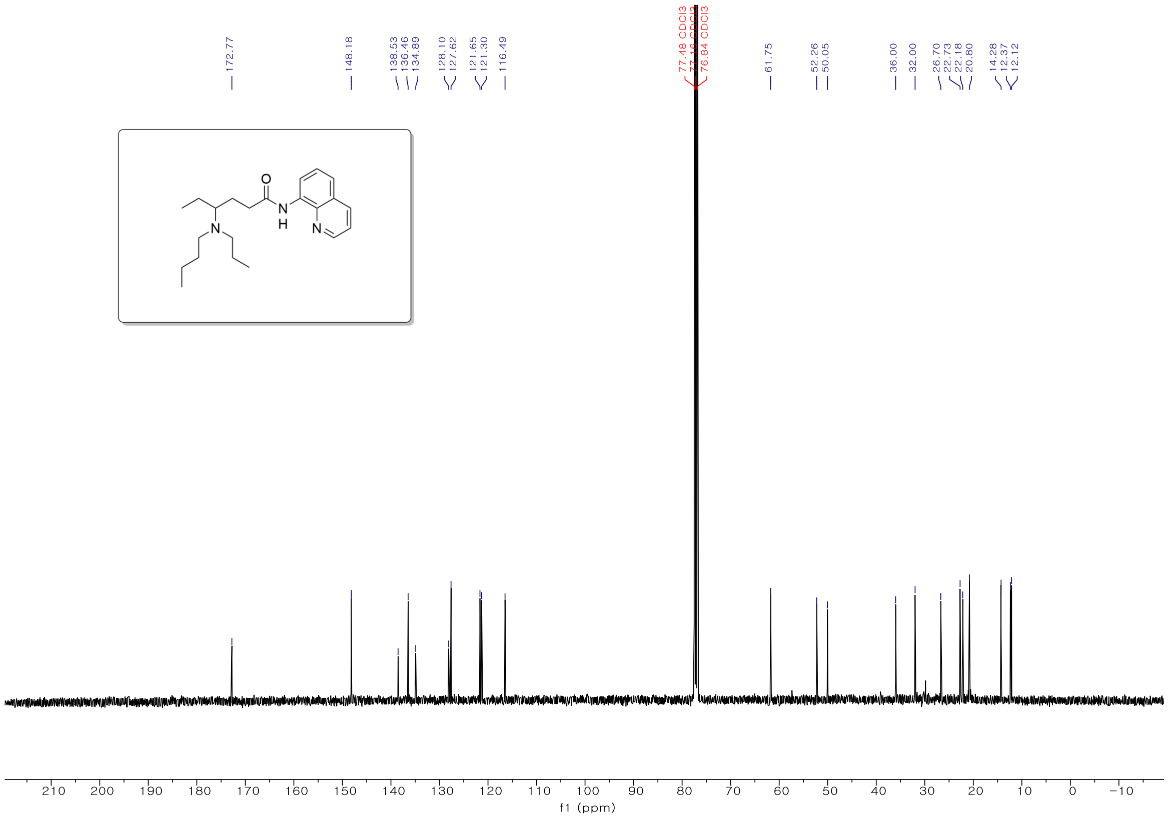

Supplementary Figure 39. ^{1}H and ^{13}C NMR of **4l**

^{1}H NMR 600 MHz, $CDCl_3$

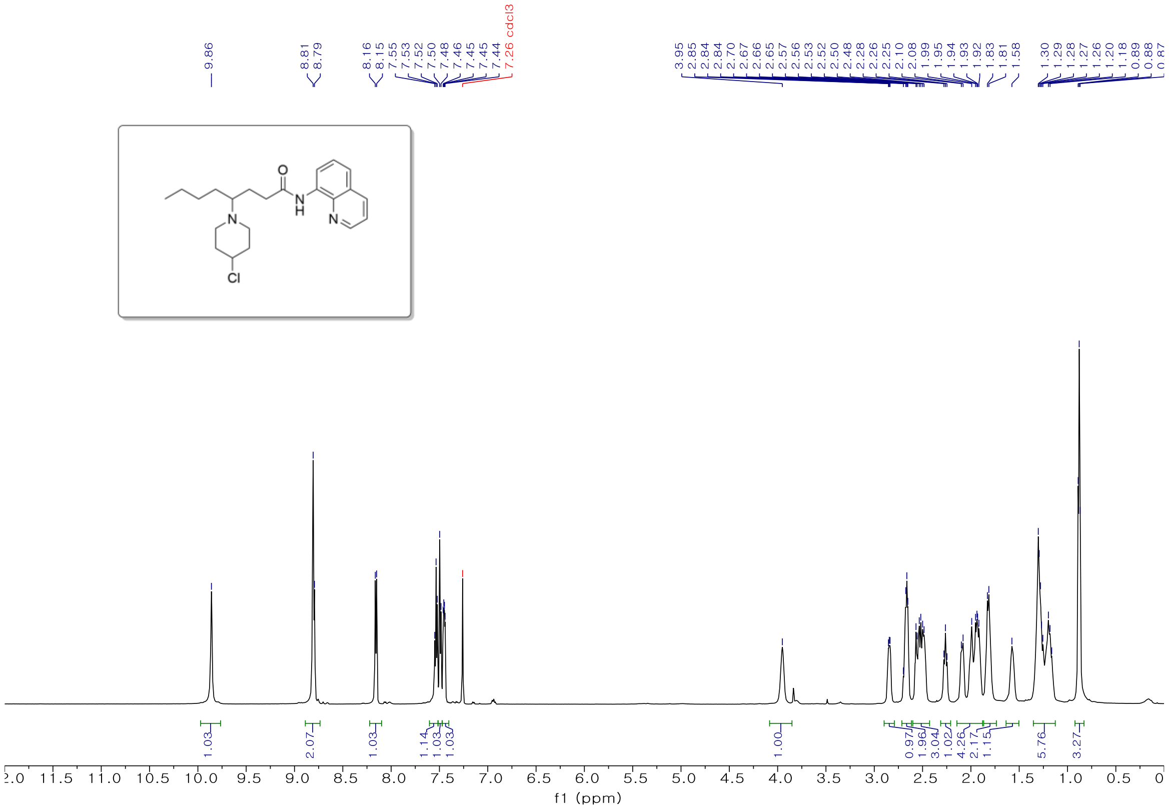

^{13}C NMR 100 MHz, $CDCl_3$

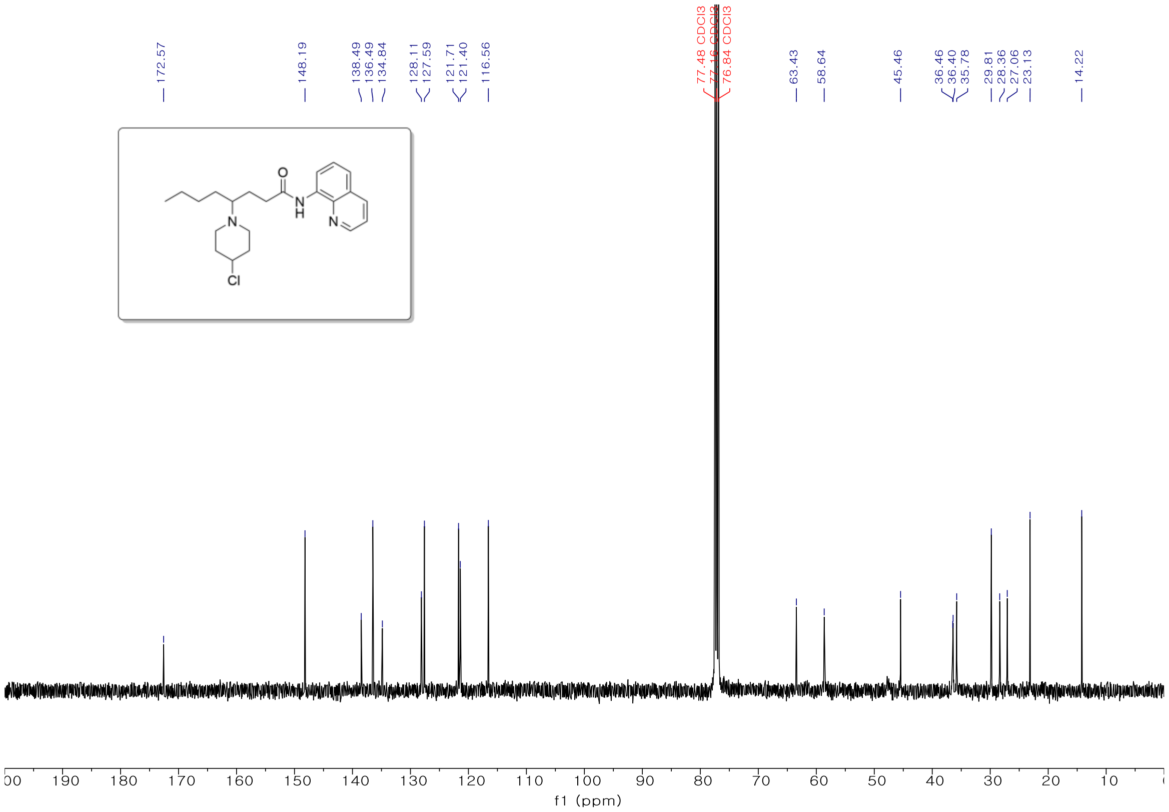

Supplementary Figure 40. ^{1}H and ^{13}C NMR of **4m**

^{1}H NMR 600 MHz, CDCl$_3$

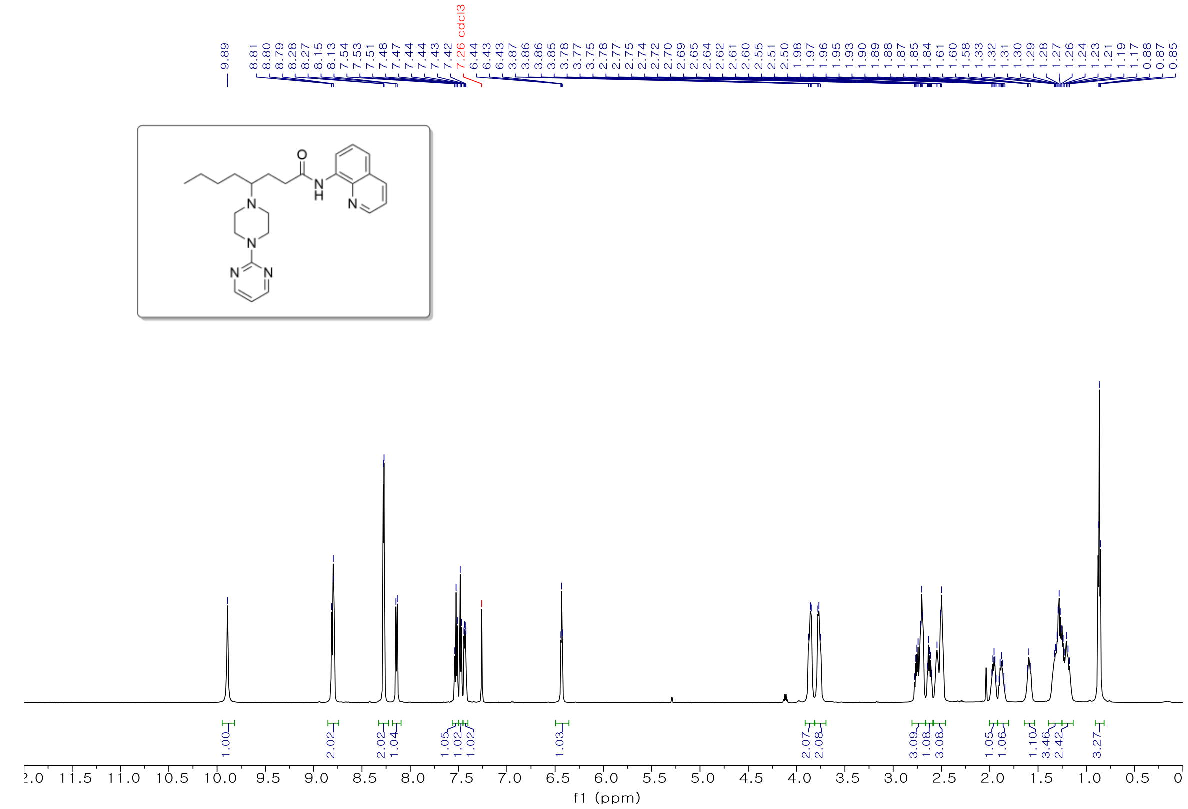

^{13}C NMR 100 MHz, CDCl$_3$

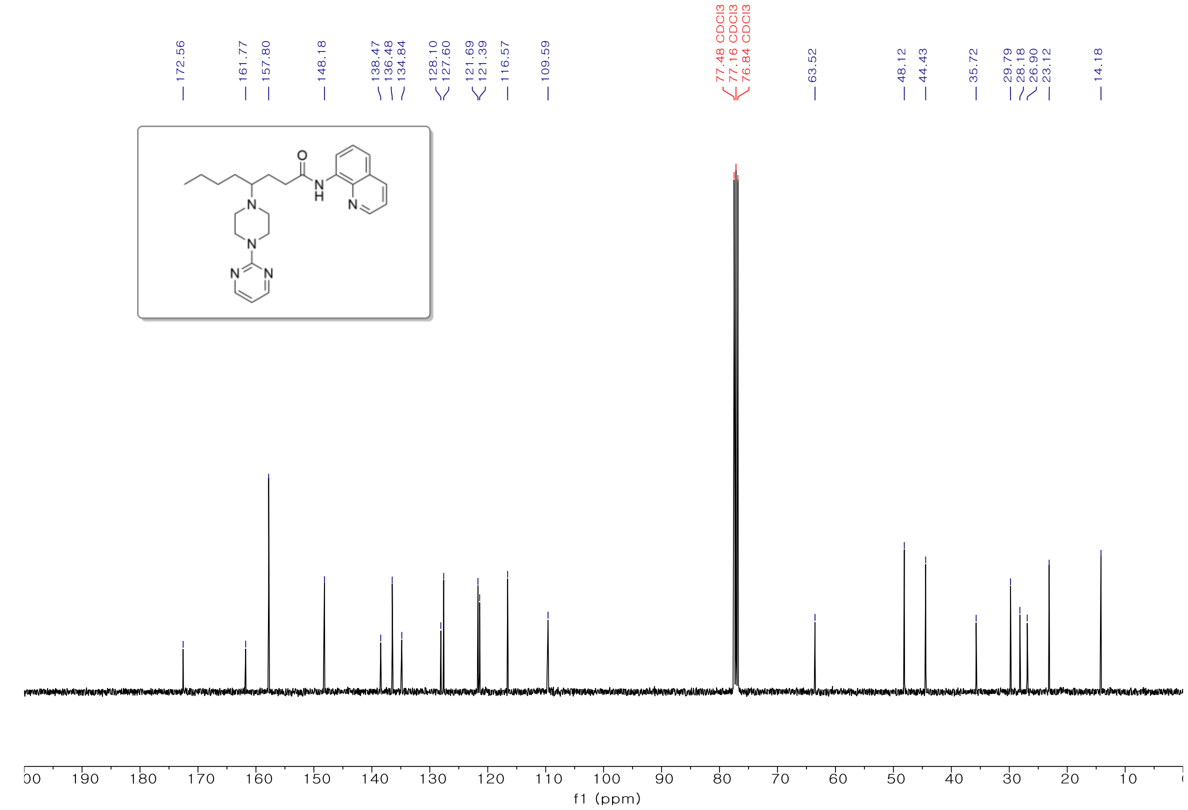

Supplementary Figure 41. ^{1}H and ^{13}C NMR of **4n**

^{1}H NMR 400 MHz, $CDCl_3$

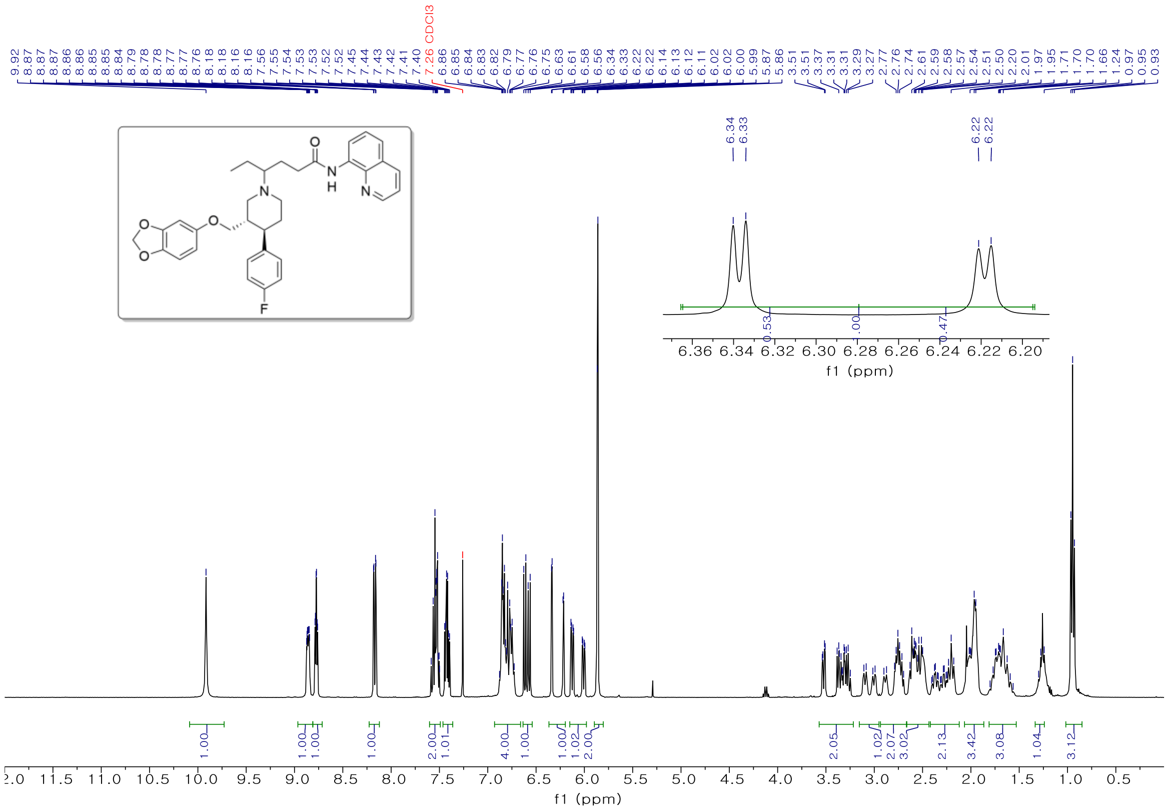

^{13}C NMR 100 MHz, $CDCl_3$

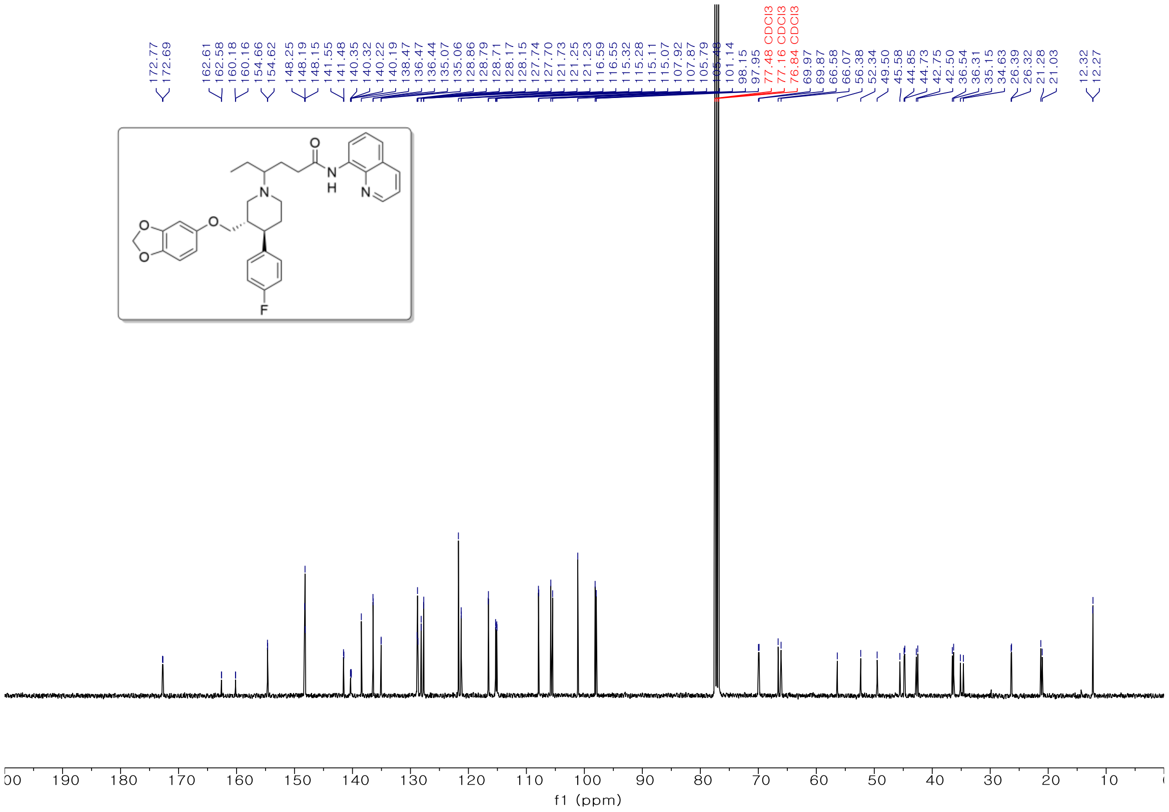

^{19}F NMR 376 MHz, CDCl$_3$

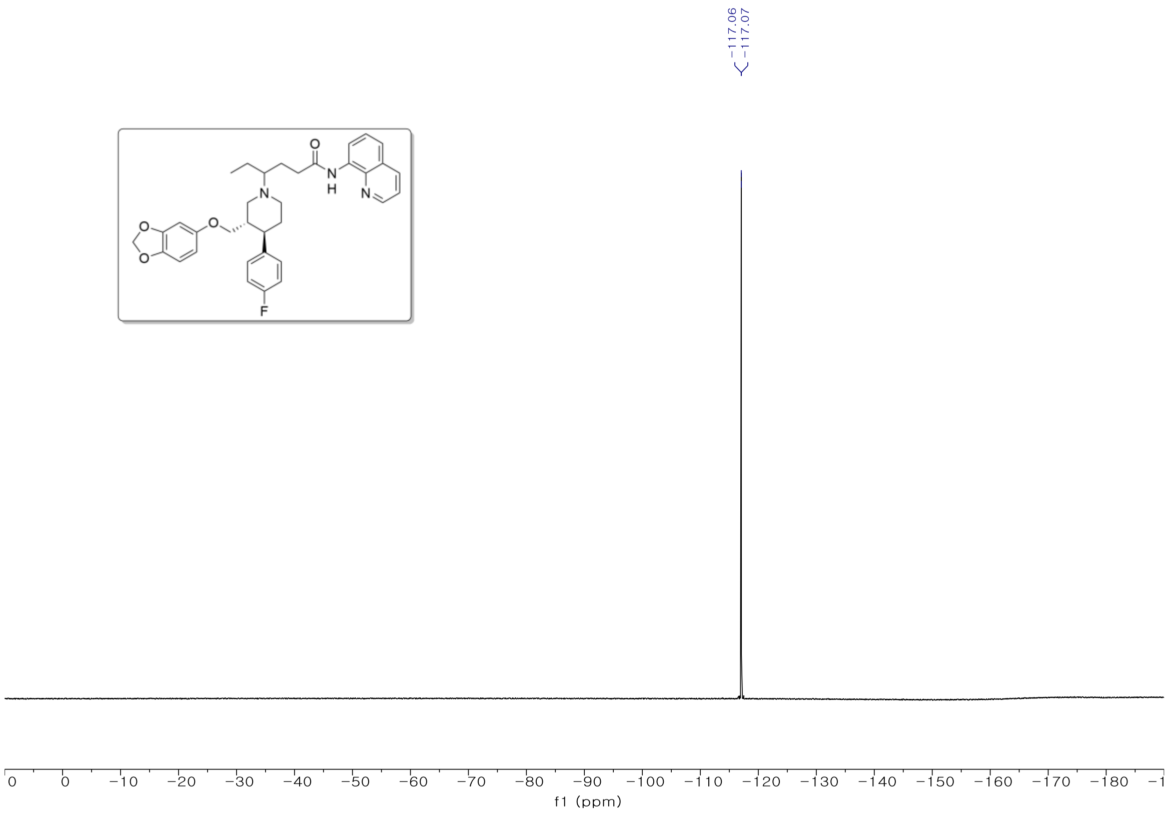

Supplementary Figure 42. ^{1}H, ^{13}C, and ^{19}F NMR of **4o** (diastereomer 1.1 : 1)

^{1}H NMR 400 MHz, $CDCl_3$

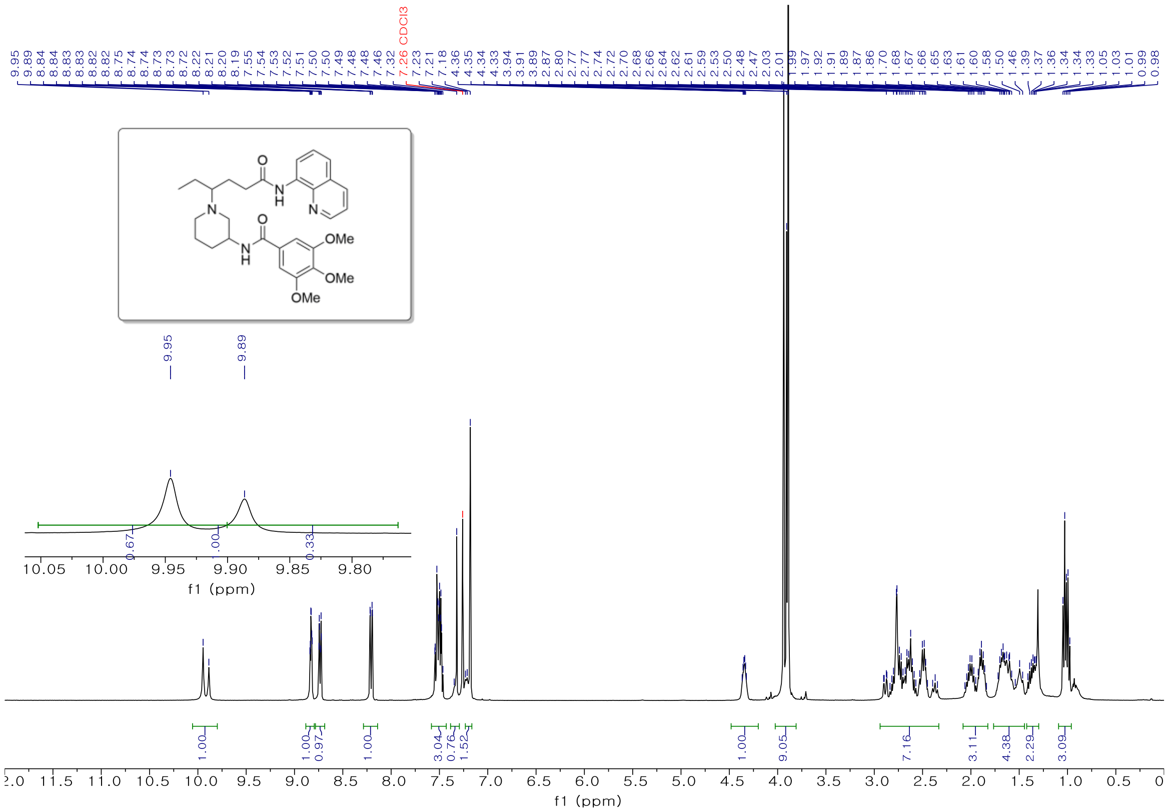

^{13}C NMR 100 MHz, $CDCl_3$

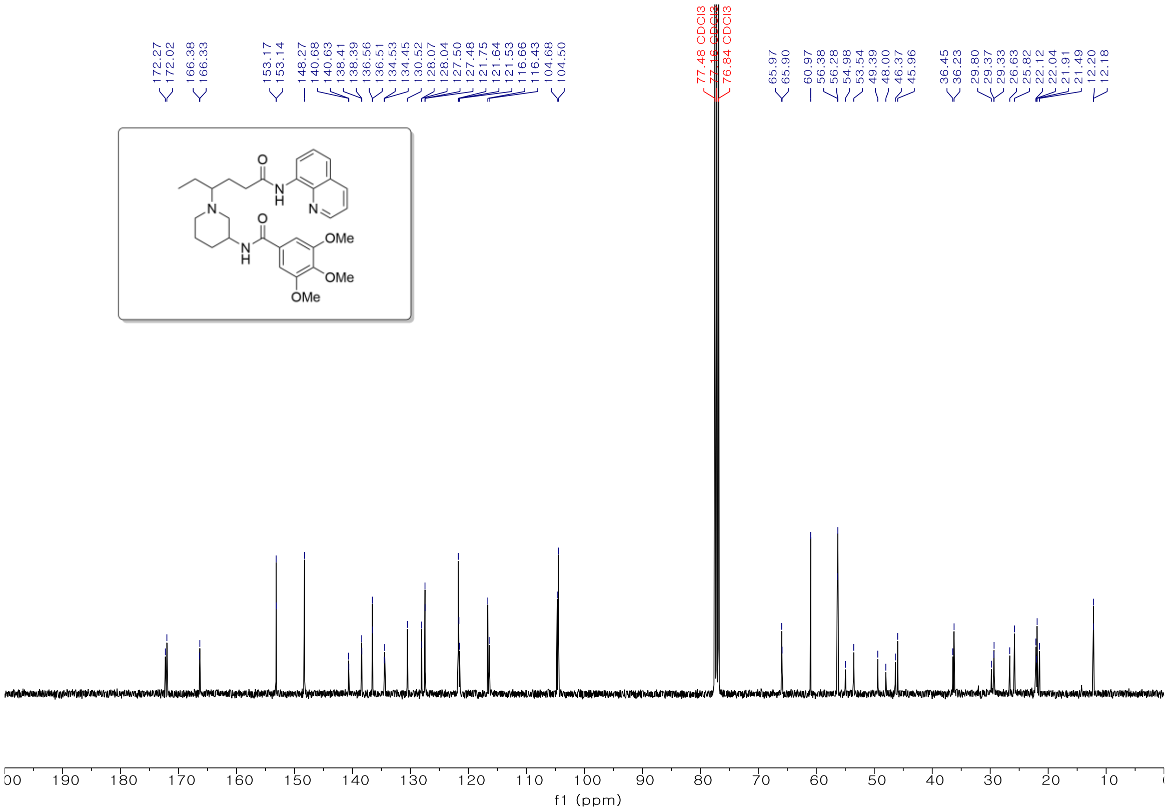

Supplementary Figure 43. ^{1}H and ^{13}C NMR of **4p** (diastereomer 2 : 1)

^{1}H NMR 400 MHz, $CDCl_3$

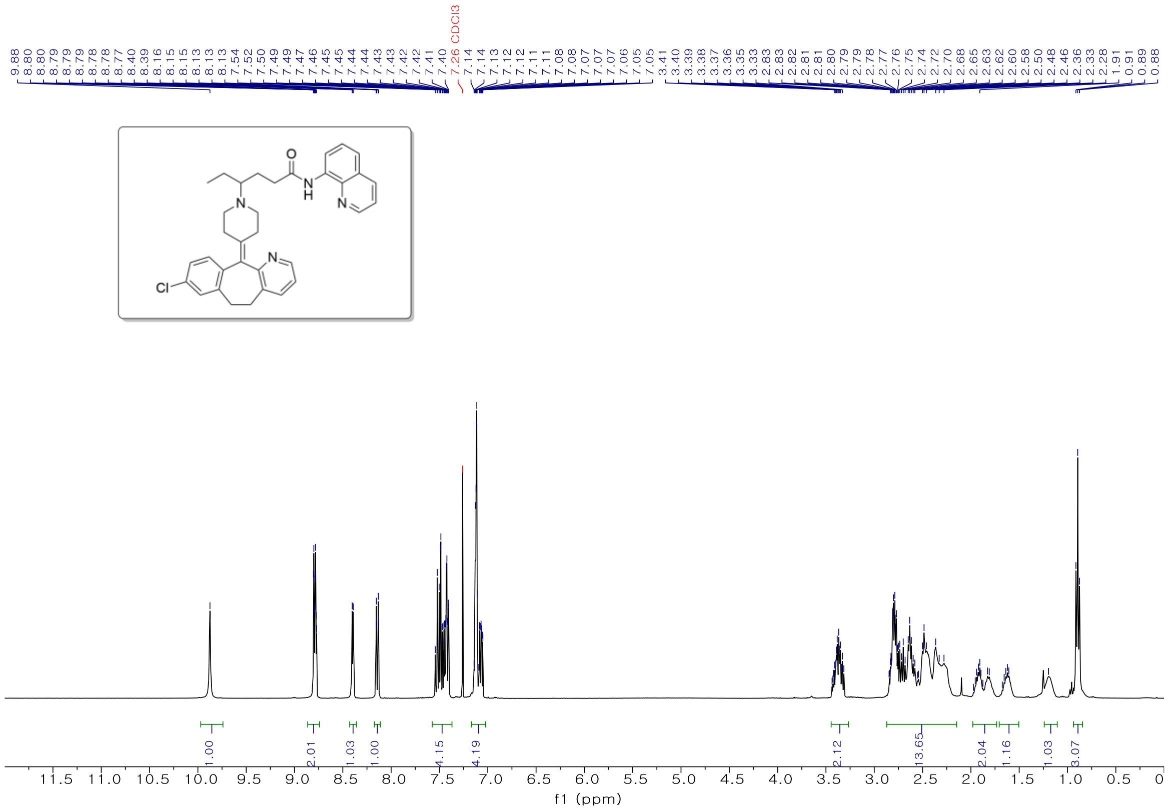

^{13}C NMR 100 MHz, $CDCl_3$

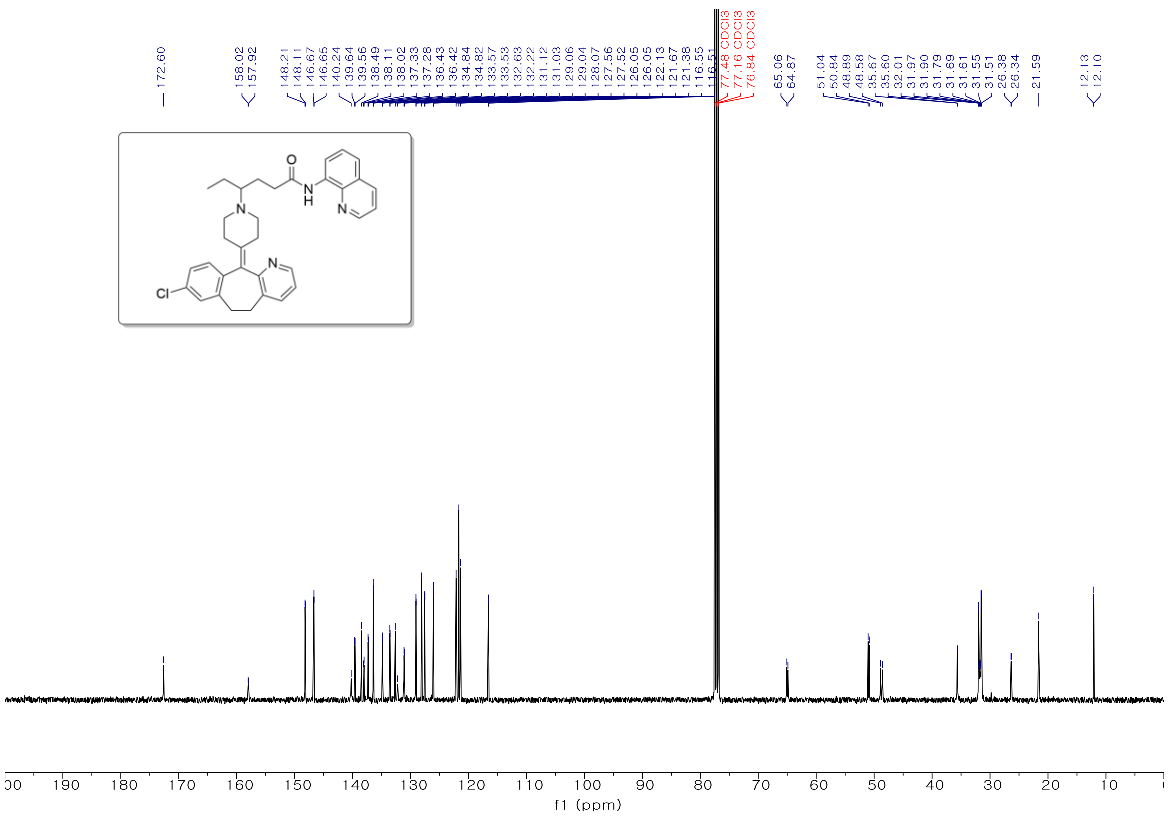

Supplementary Figure 44. ^{1}H and ^{13}C NMR of **4q** (mixture of rotamers)

^{1}H NMR 600 MHz, CD_2Cl_2

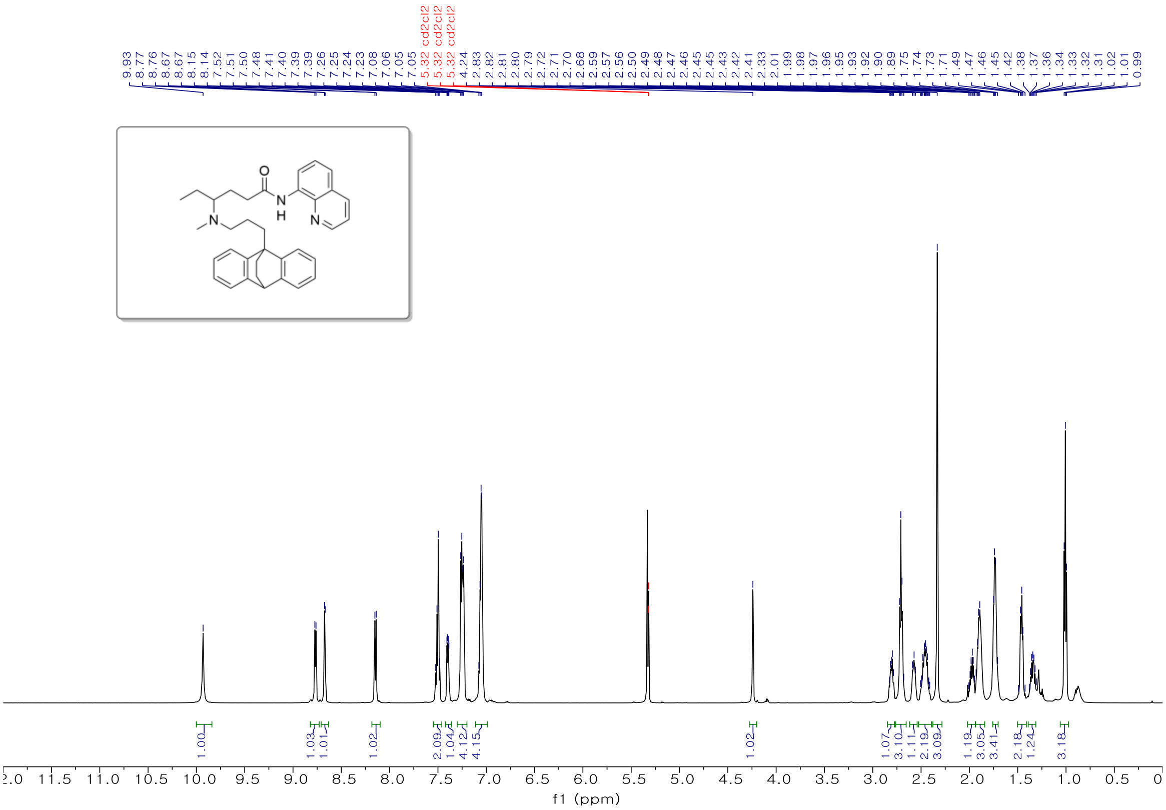

^{13}C NMR 100 MHz, CD_2Cl_2

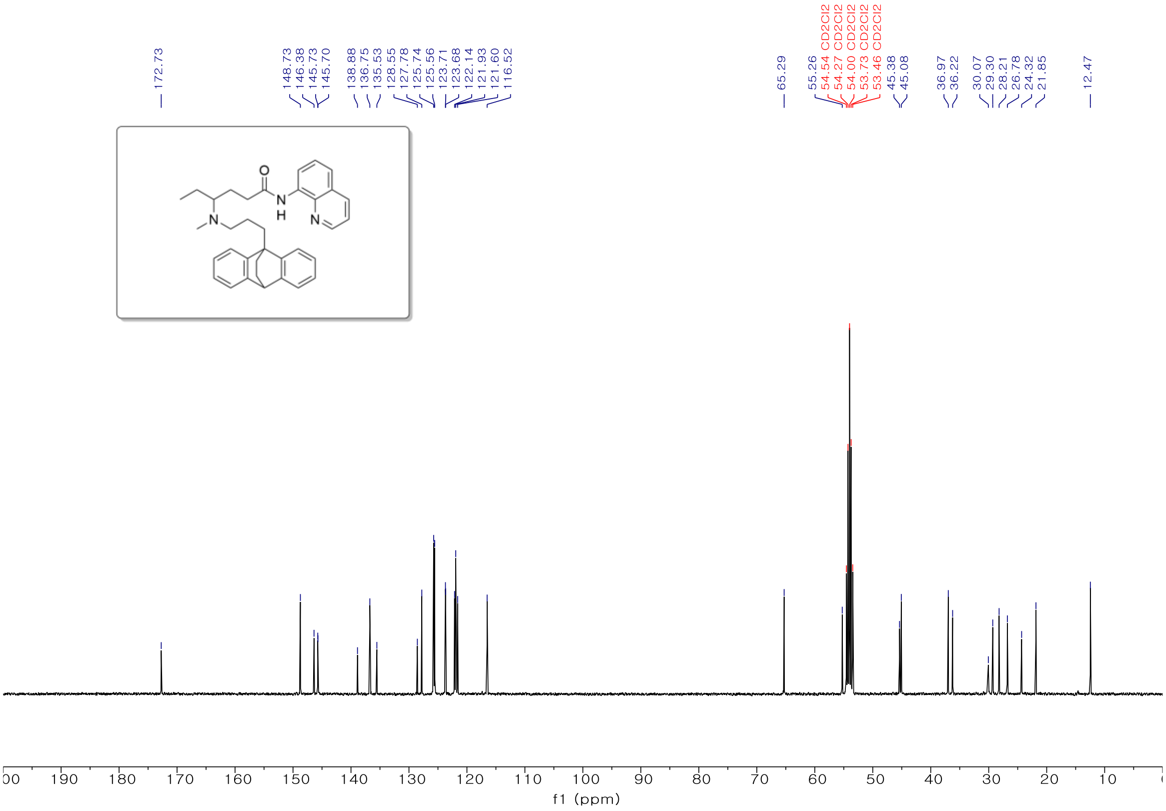

Supplementary Figure 45. ^{1}H and ^{13}C NMR of **4r**

^{1}H NMR 600 MHz, CD_2Cl_2

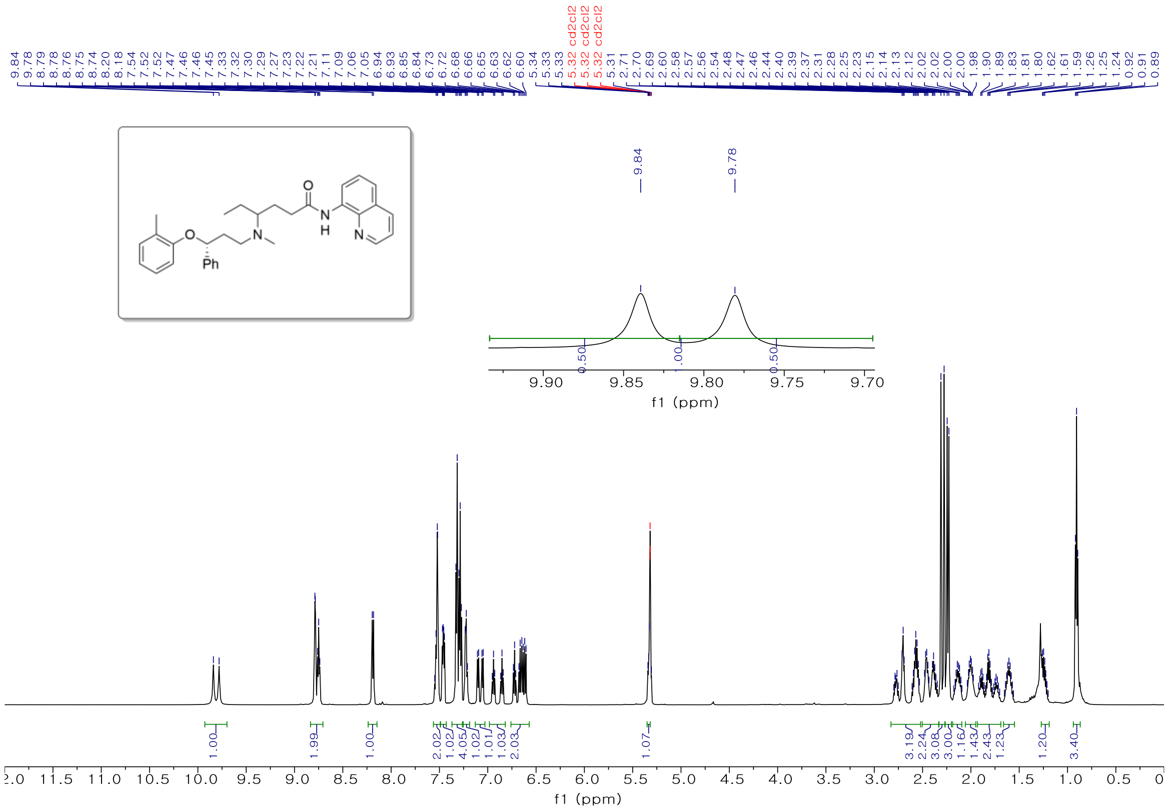

^{13}C NMR 100 MHz, CD_2Cl_2

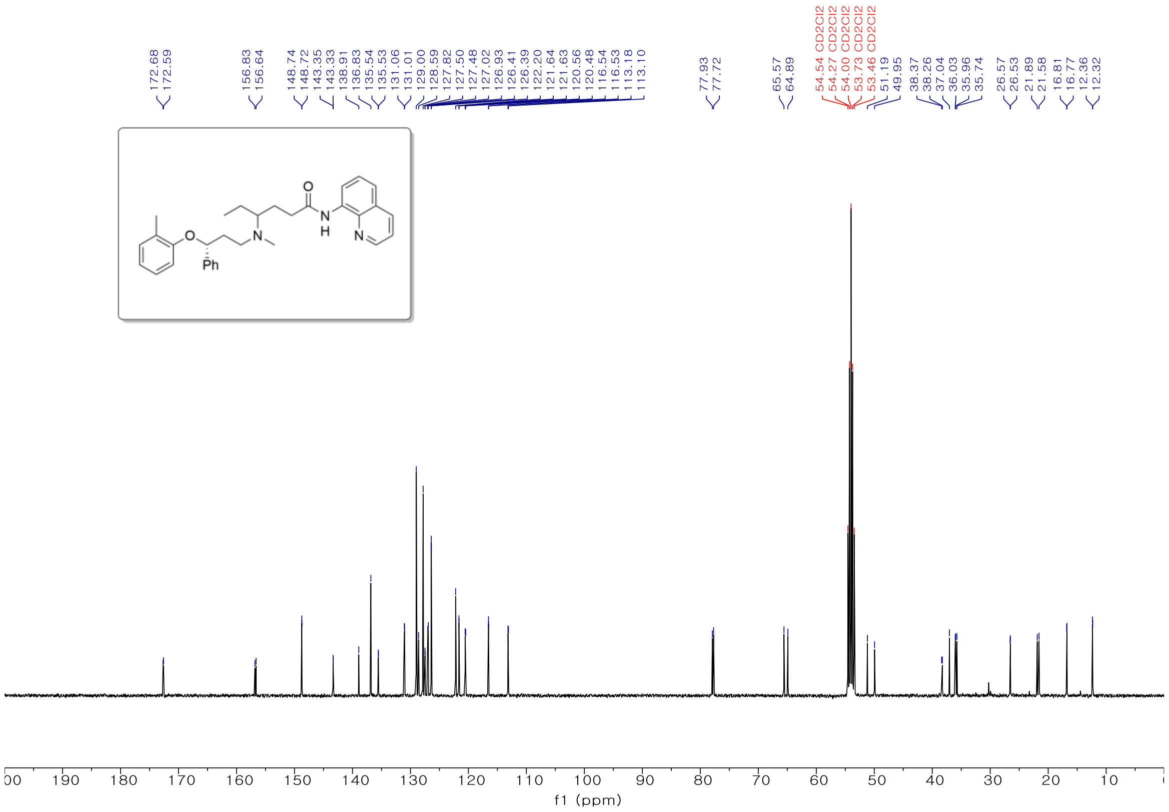

Supplementary Figure 46. ^{1}H and ^{13}C NMR of **4s** (diastereomer 1 : 1)

^{1}H NMR 400 MHz, CDCl$_3$

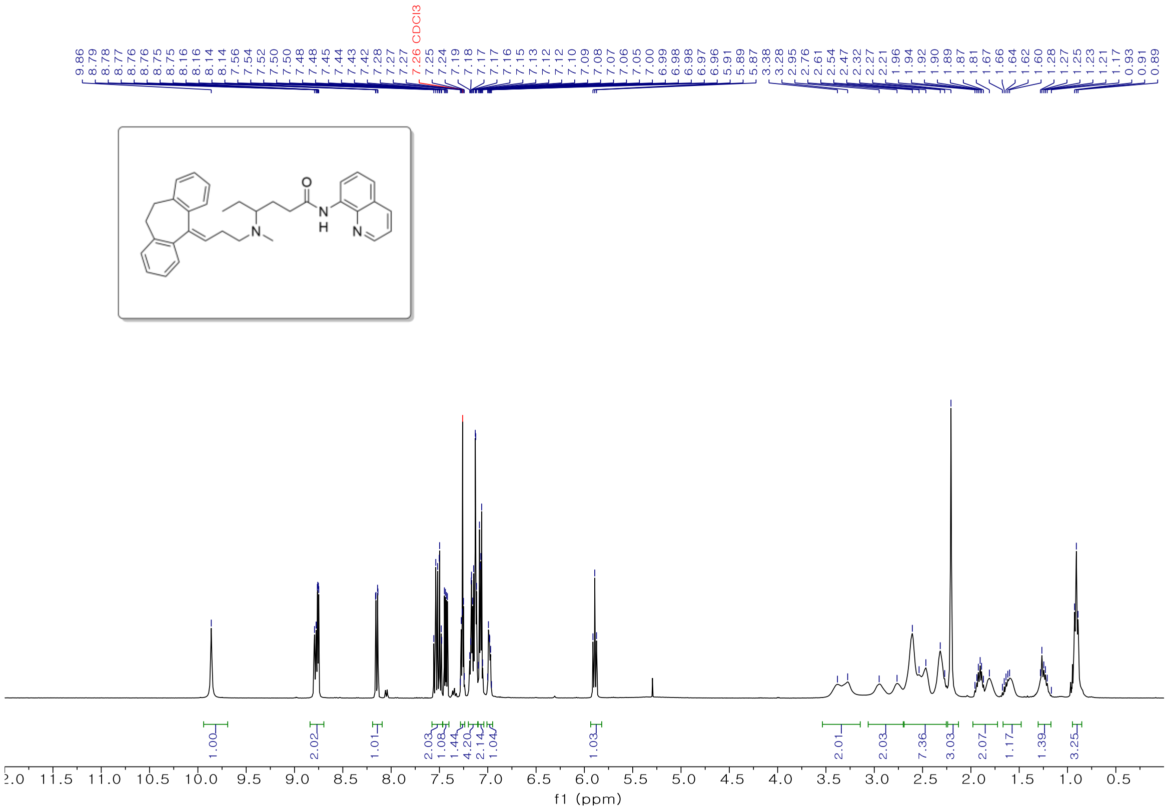

^{13}C NMR 100 MHz, CDCl$_3$

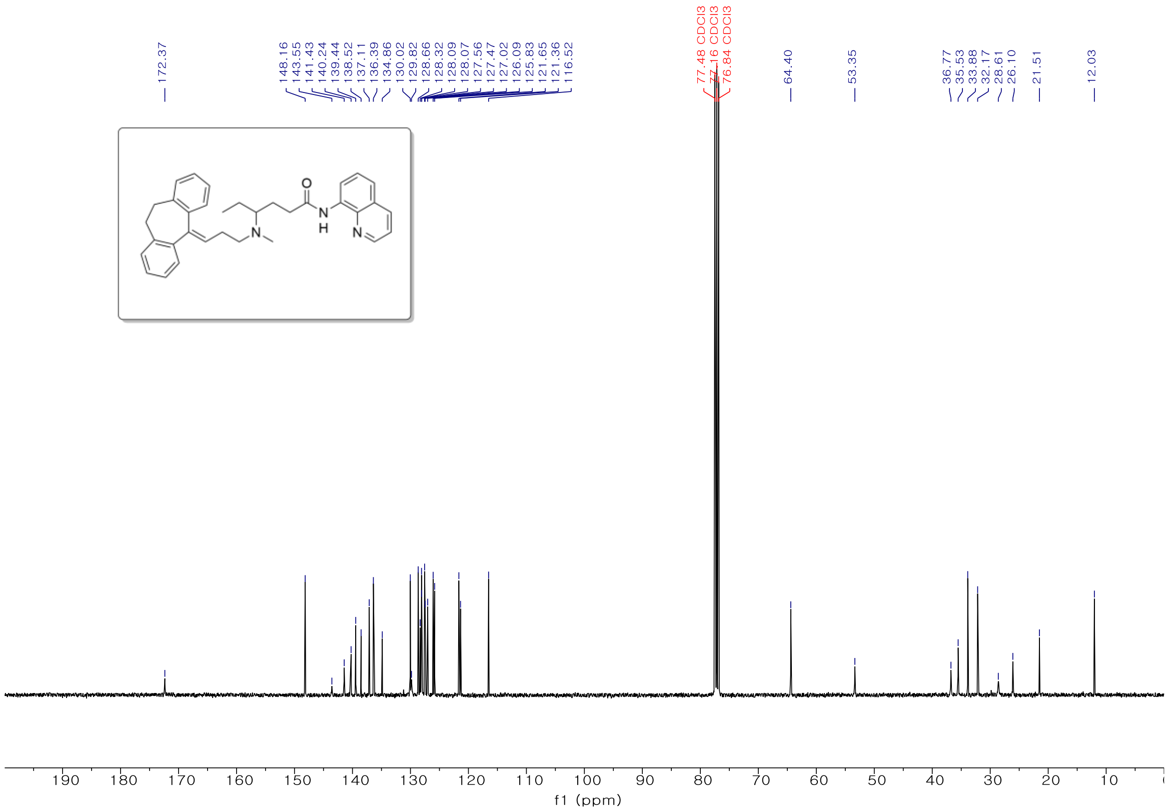

Supplementary Figure 47. ^{1}H and ^{13}C NMR of **4t**

^{1}H NMR 600 MHz, $CDCl_3$

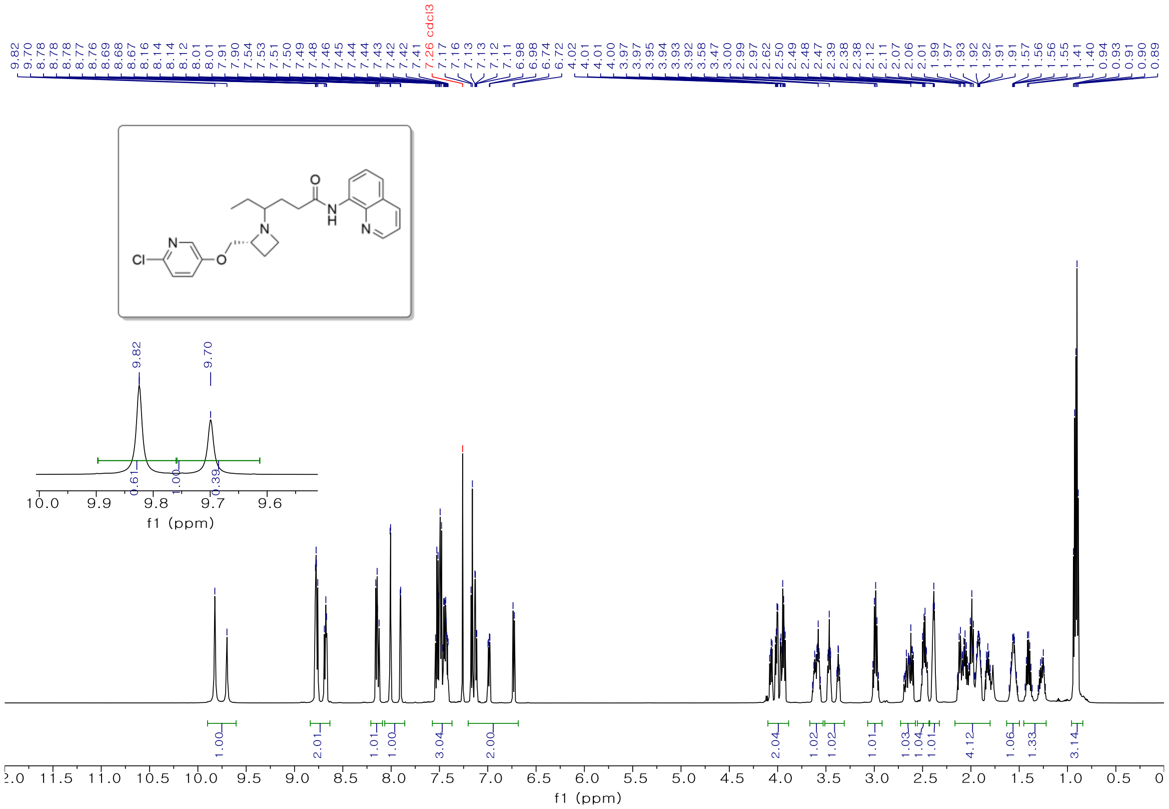

^{13}C NMR 100 MHz, $CDCl_3$

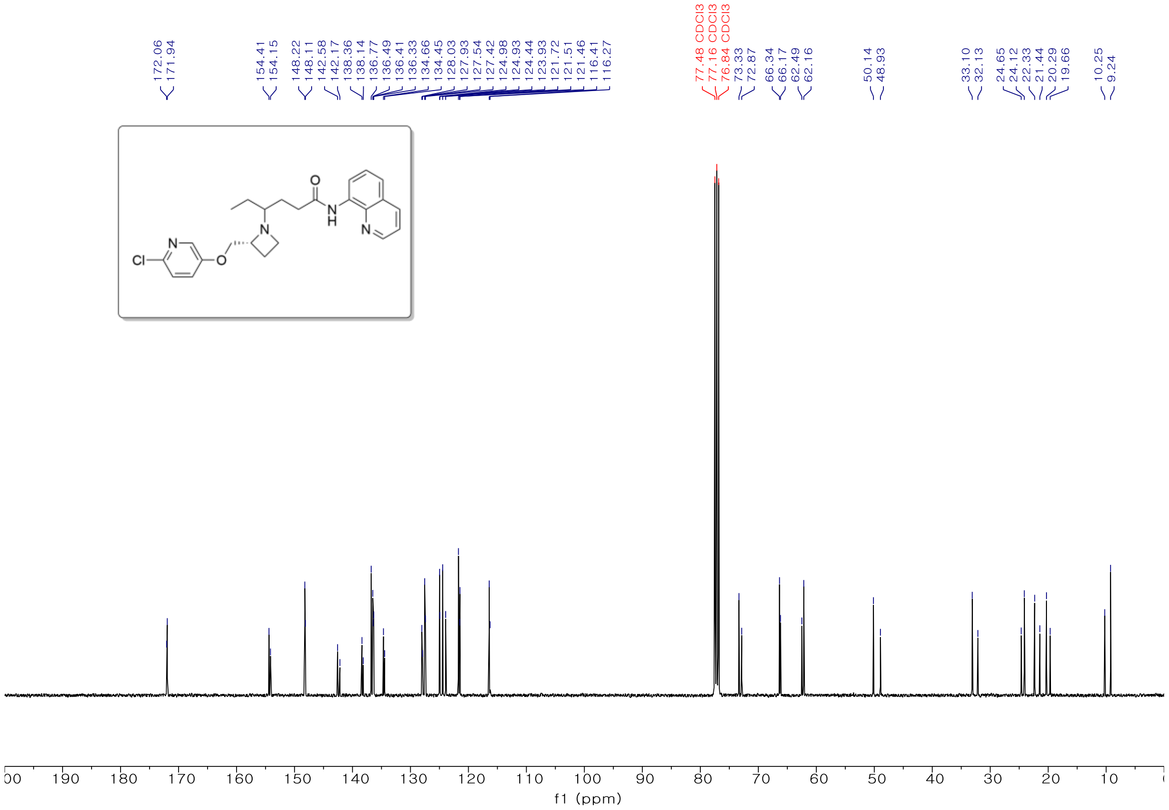

Supplementary Figure 48. ^{1}H and ^{13}C NMR of **4u**

^{1}H NMR 600 MHz, CD_2Cl_2

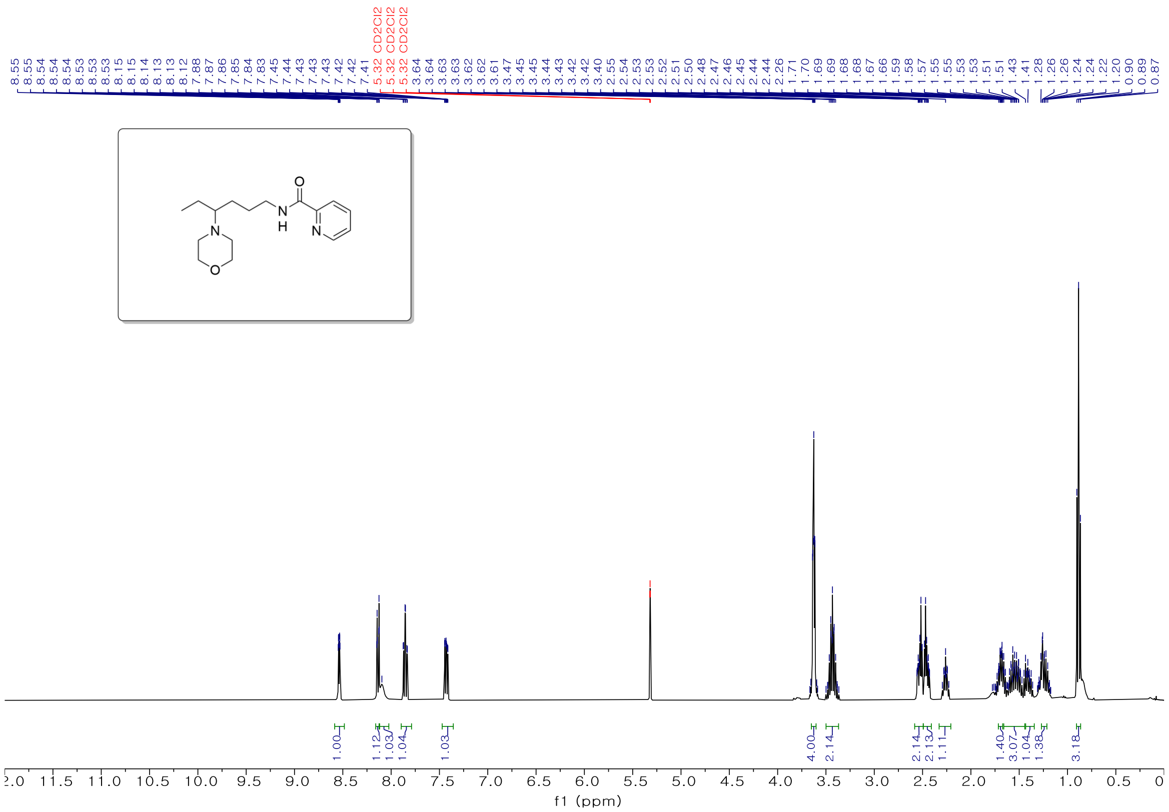

^{13}C NMR 100 MHz, CD_2Cl_2

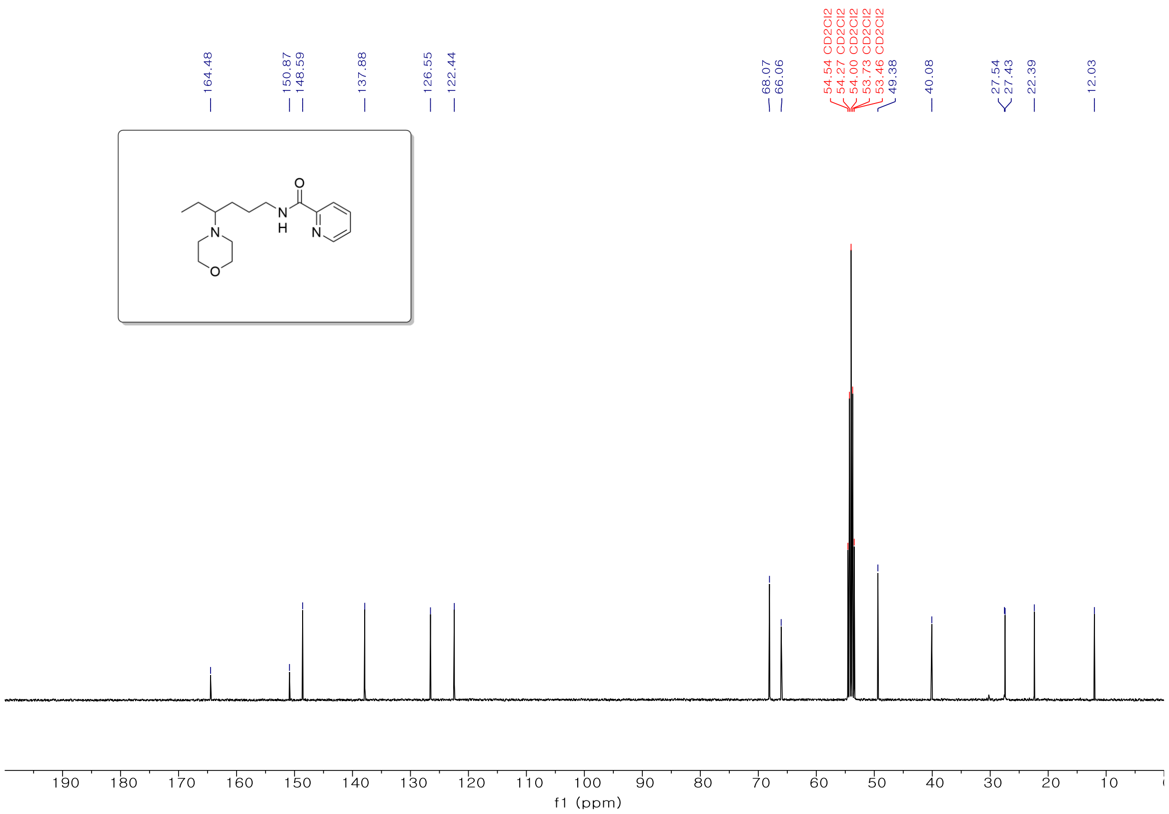

Supplementary Figure 49. ^{1}H and ^{13}C NMR of **6a**

^{1}H NMR 600 MHz, $CDCl_3$

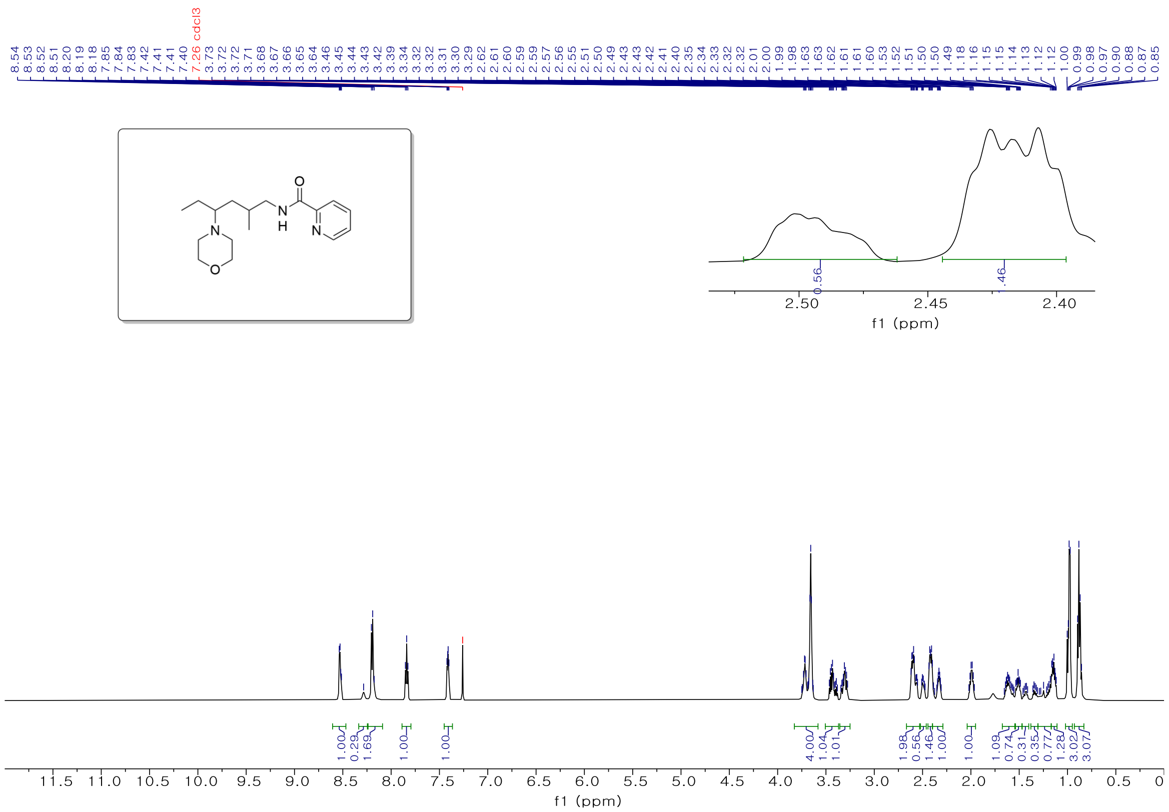

^{13}C NMR 100 MHz, $CDCl_3$

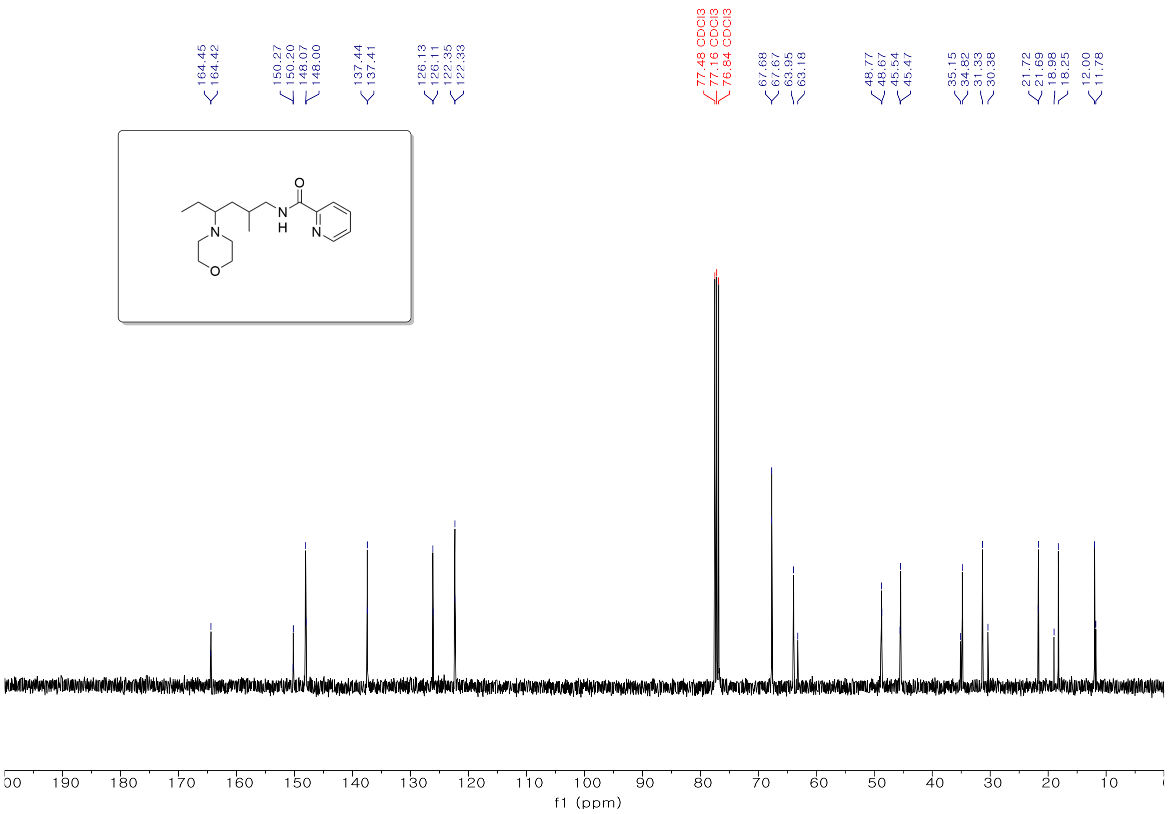

Supplementary Figure 50. ^{1}H and ^{13}C NMR of **6b** (diasteromer 2.6:1)

^{1}H NMR 400 MHz, $CDCl_3$

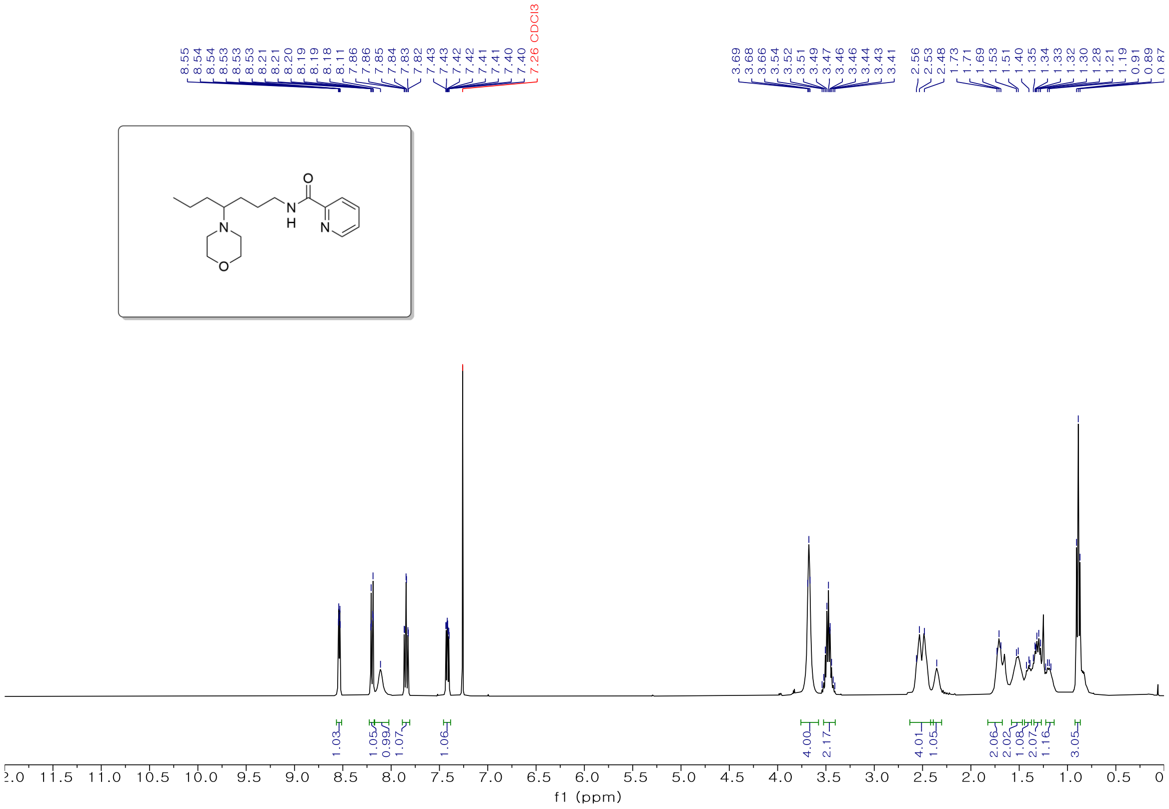

^{13}C NMR 100 MHz, $CDCl_3$

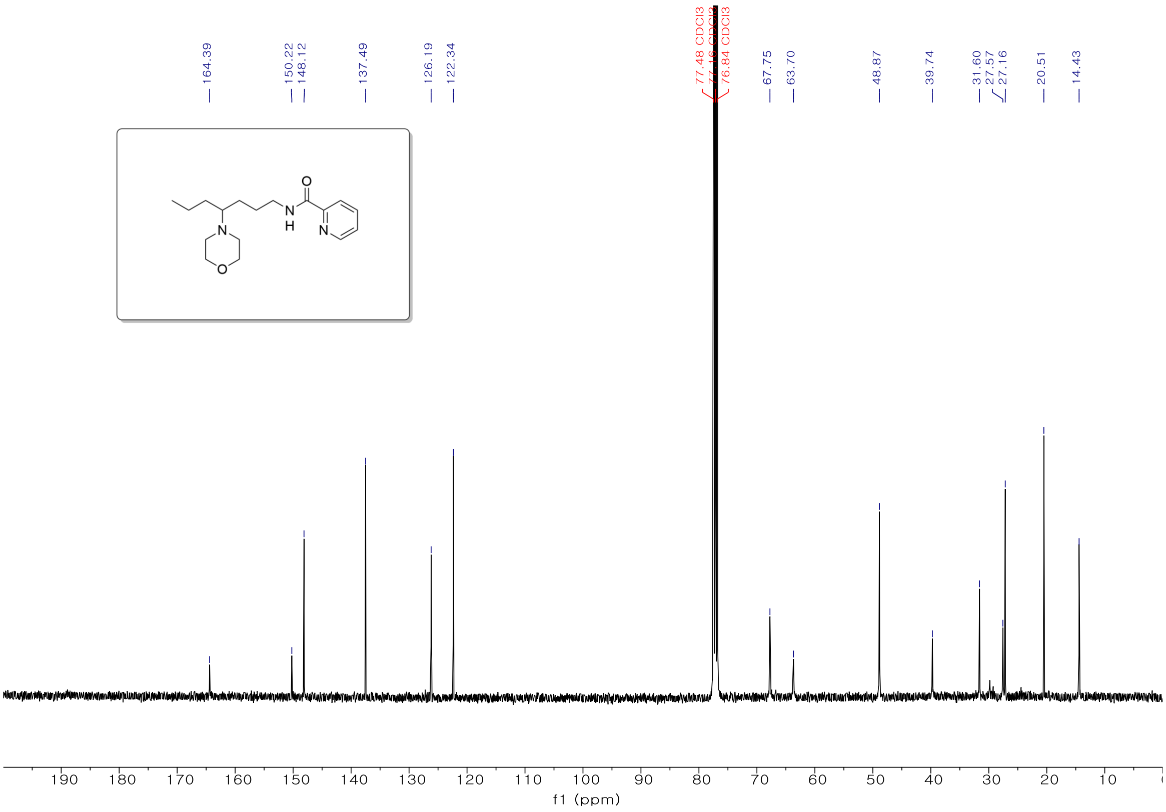

Supplementary Figure 51. ^{1}H and ^{13}C NMR of **6c**

^{1}H NMR 600 MHz, $CDCl_3$

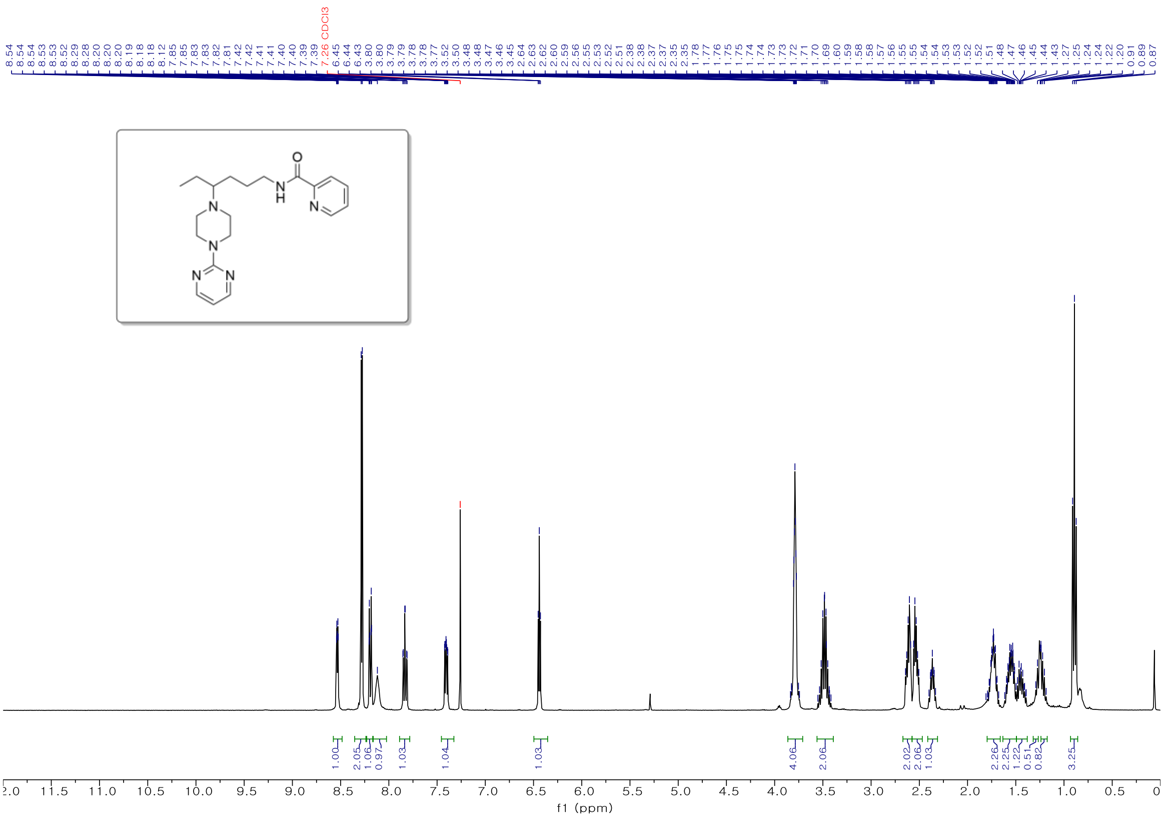

^{13}C NMR 100 MHz, $CDCl_3$

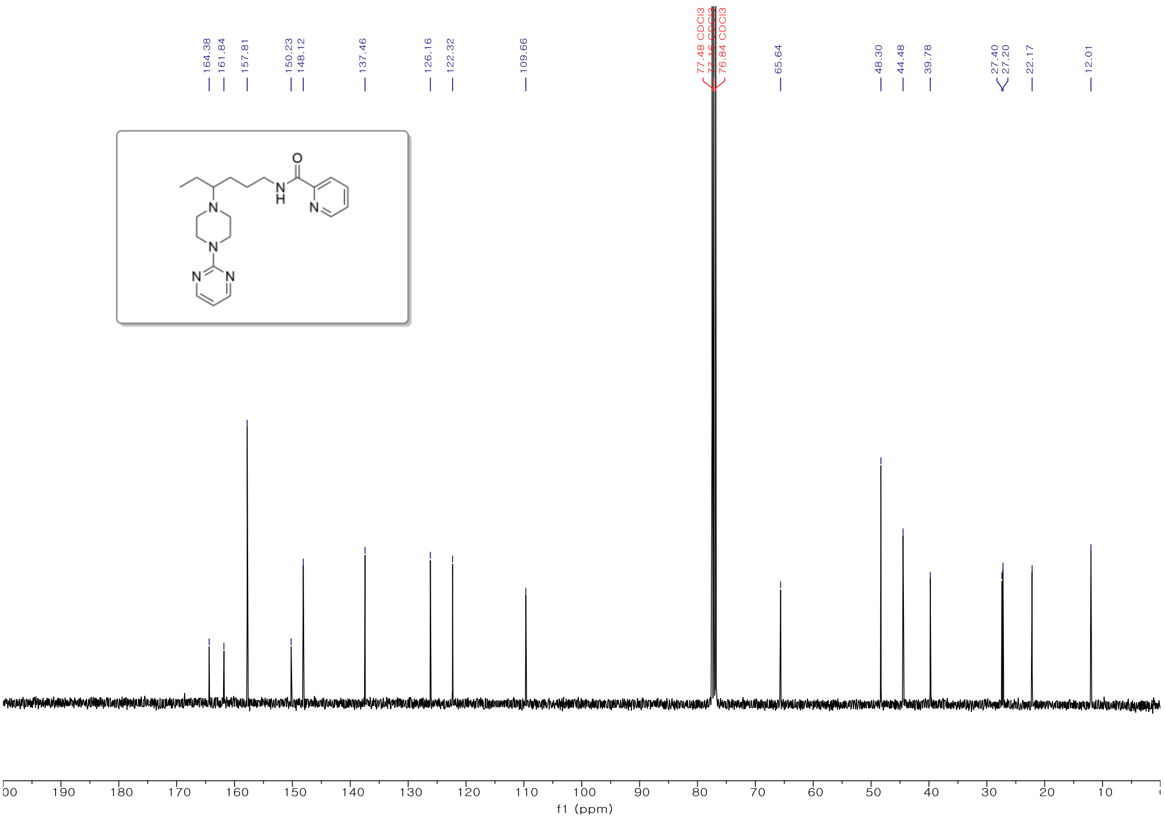

Supplementary Figure 52. ^{1}H and ^{13}C NMR of **6d**

^{1}H NMR 400 MHz, CD_2Cl_2

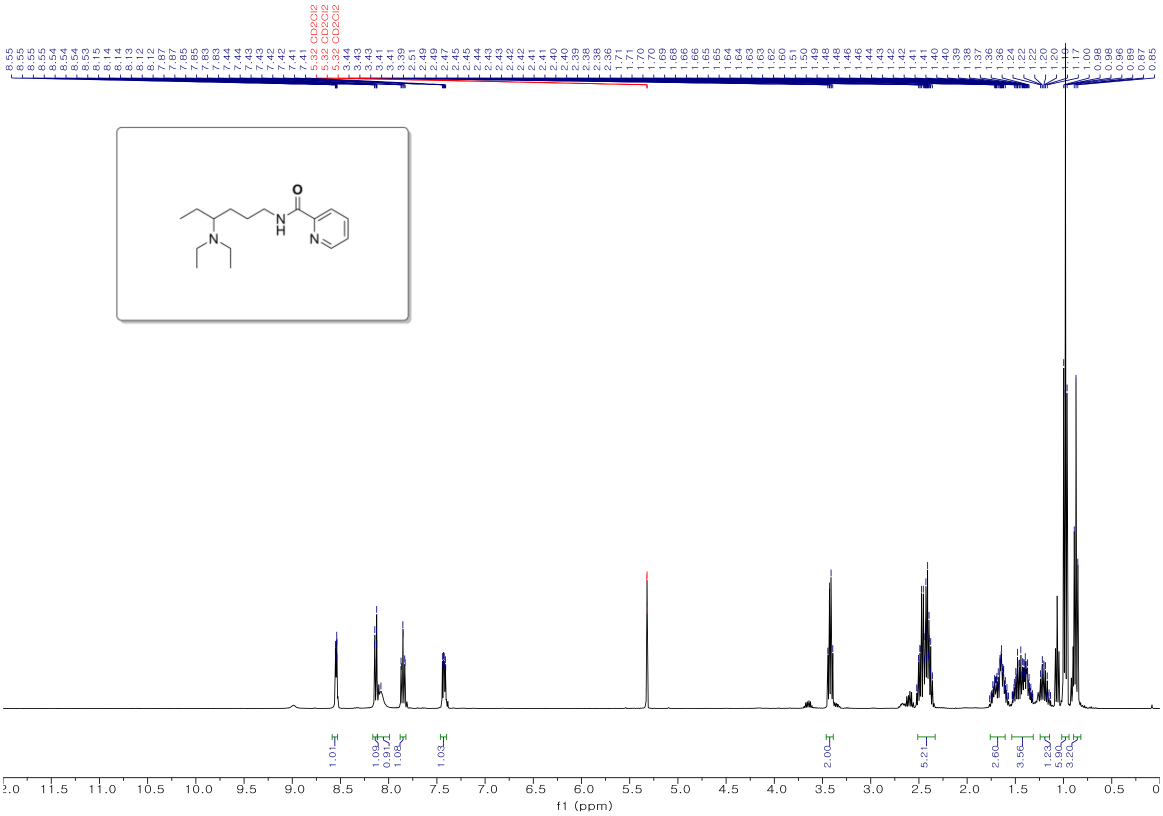

^{13}C NMR 100 MHz, CD_2Cl_2

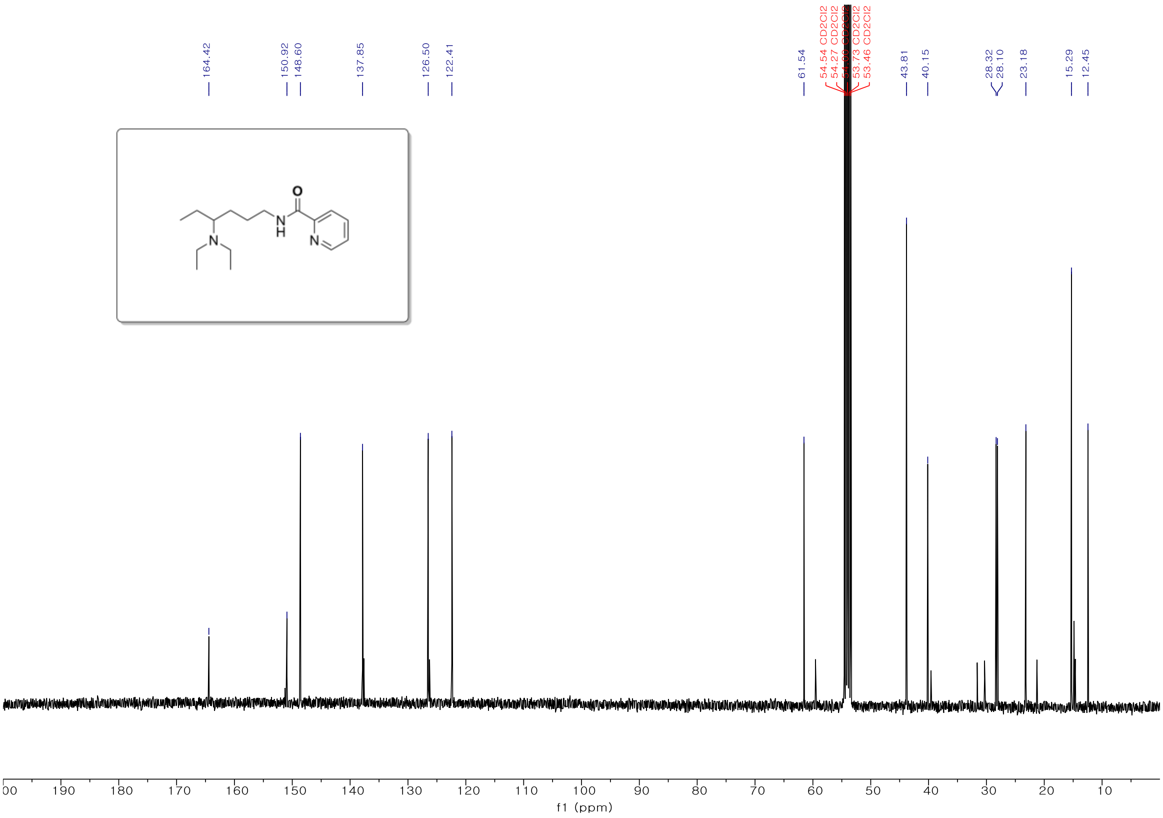

Supplementary Figure 53. ^{1}H and ^{13}C NMR of **6e**

^{1}H NMR 600 MHz, Acetonitrile-d_3

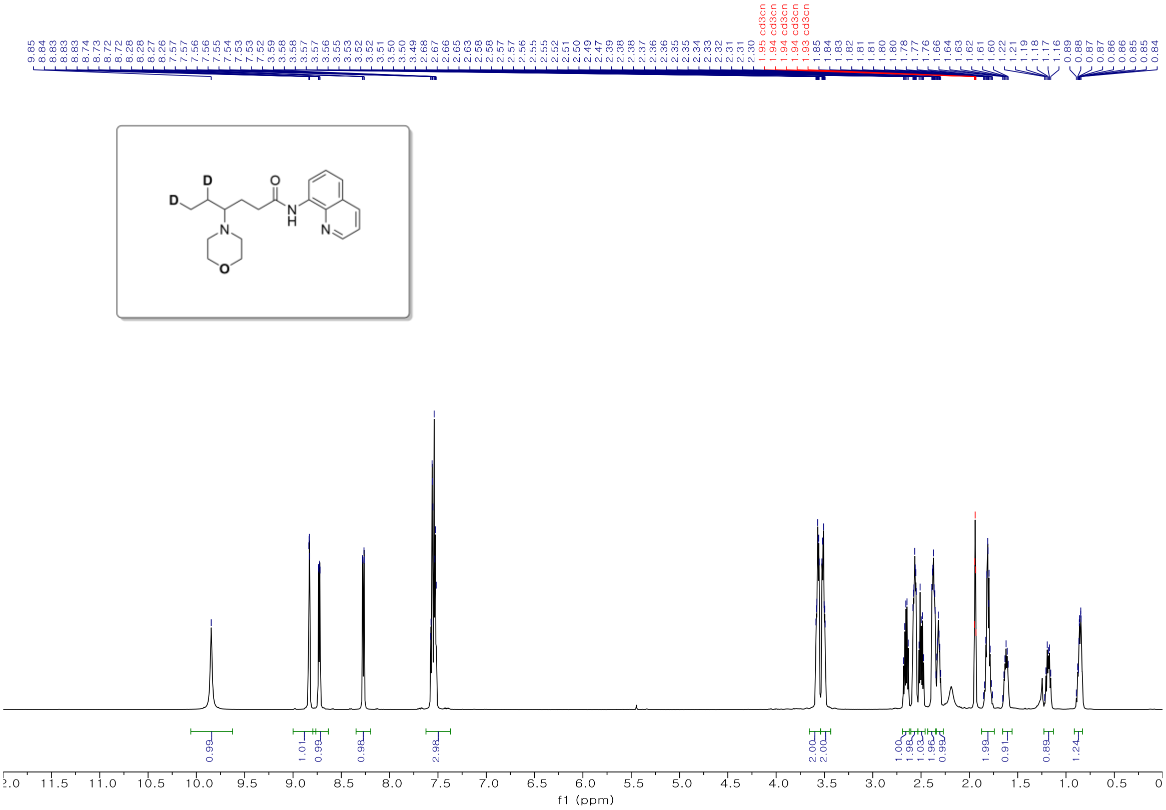

Supplementary Figure 54. ^{1}H NMR of ***d_2*-3a**

^{1}H NMR 600 MHz, Acetonitrile-*d_3*

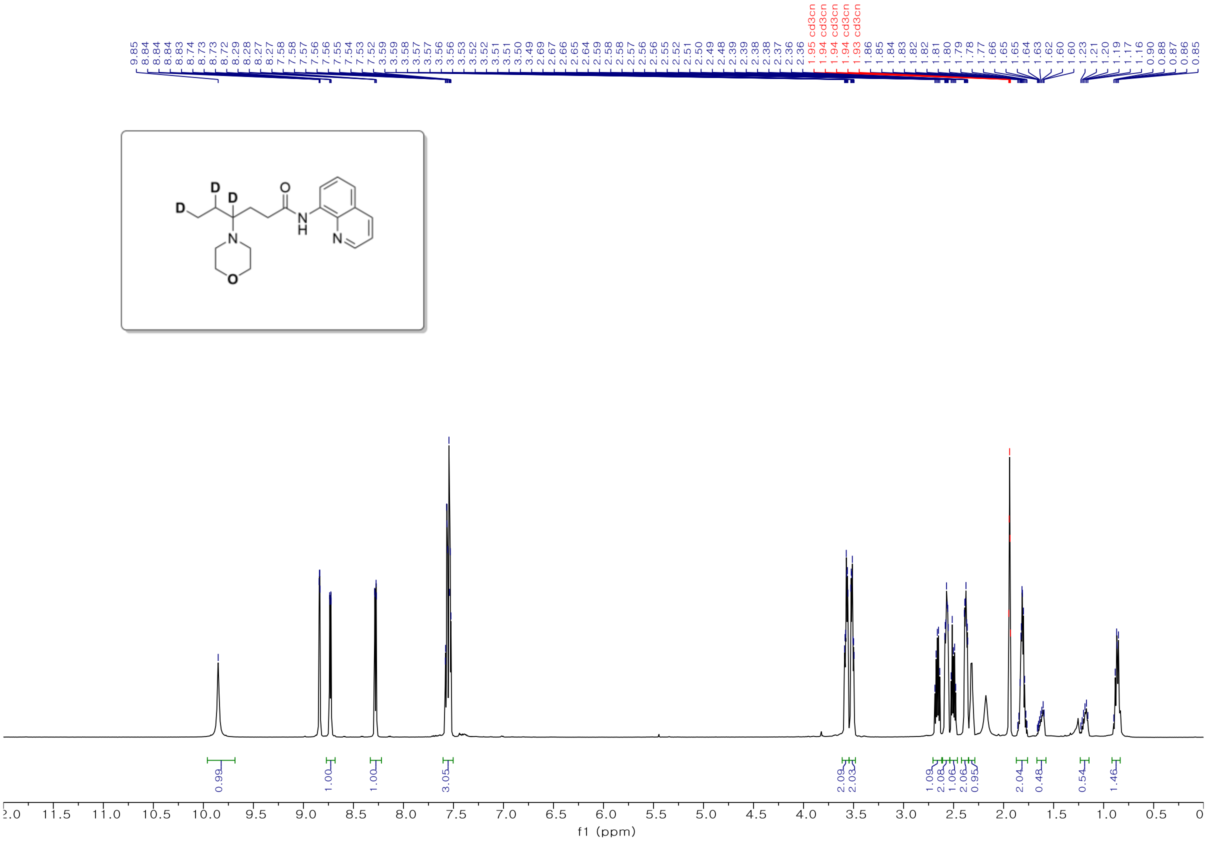

Supplementary Figure 55. ^{1}H NMR of ***d_3*-3a**

^{1}H NMR 600 MHz, $CDCl_3$

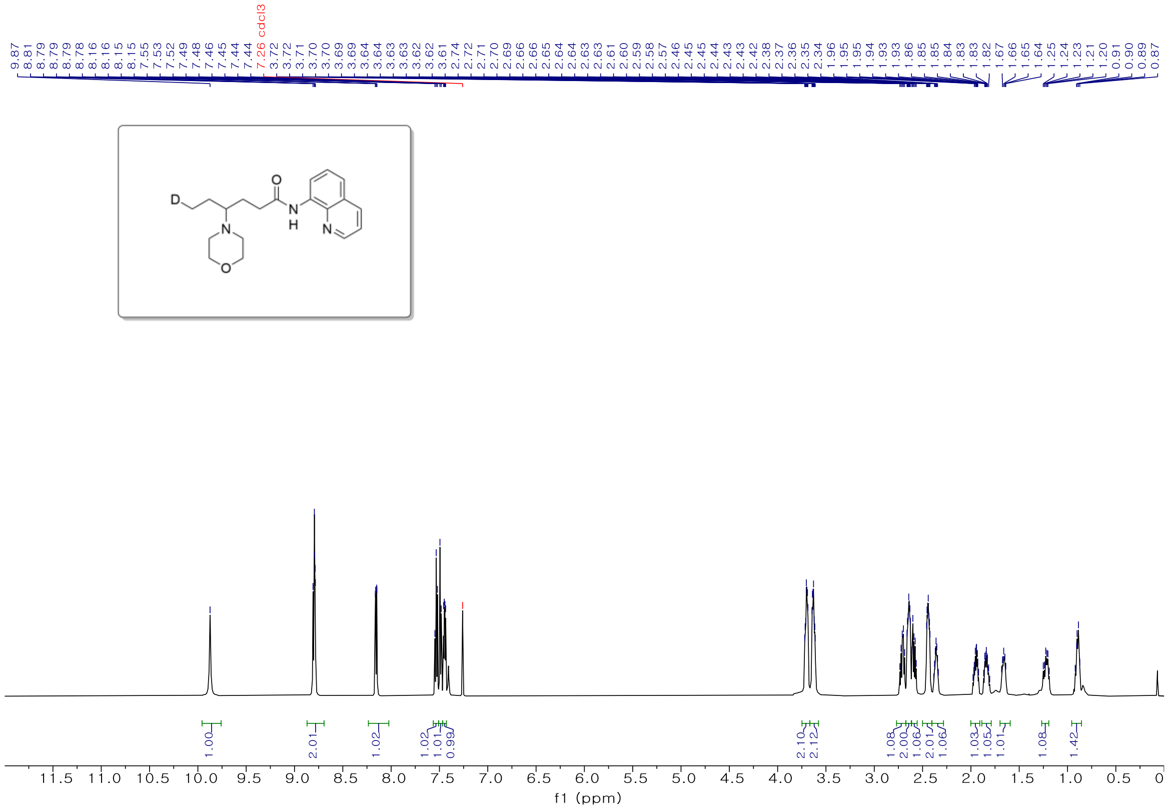

Supplementary Figure 56. ^{1}H NMR of ***d*-3a**

^{1}H NMR 600 MHz, CDCl$_3$

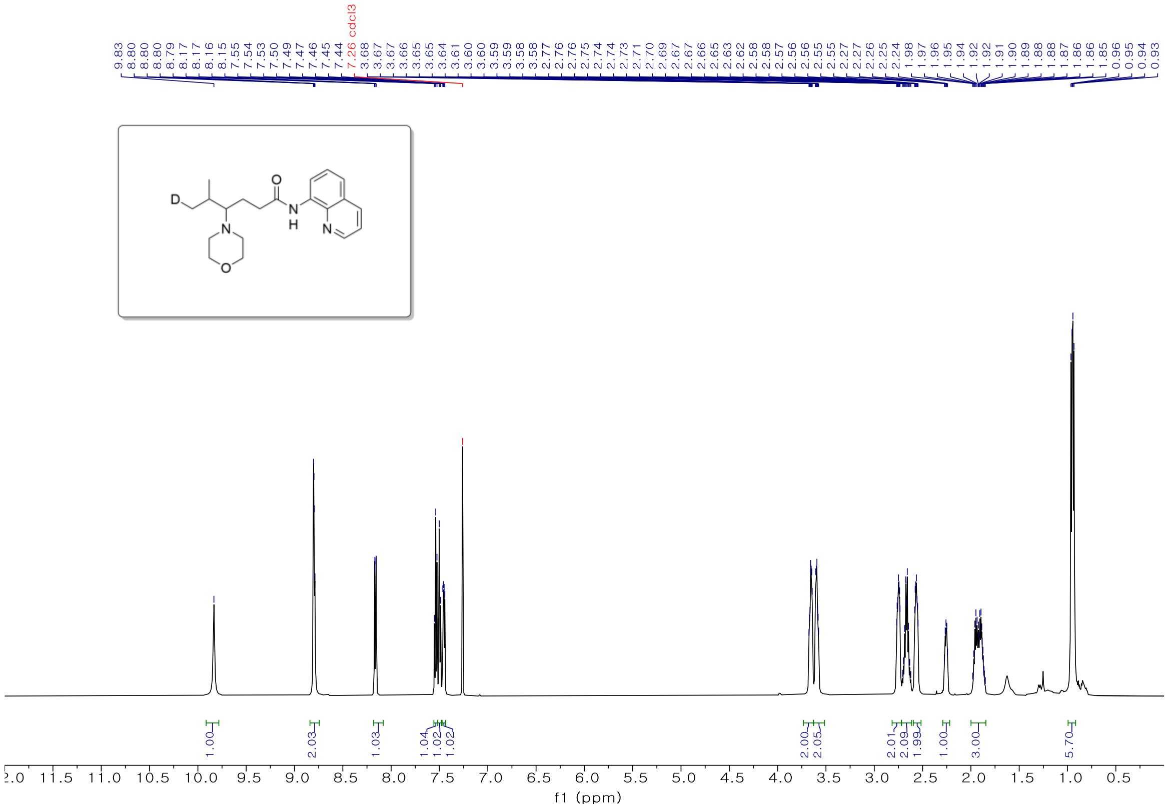

Supplementary Figure 57. ^{1}H NMR of ***d*-3d**

Appendix II

Crystallographic Data for 3i

Crystallographic Data for 3i (CCDC : 2071903)

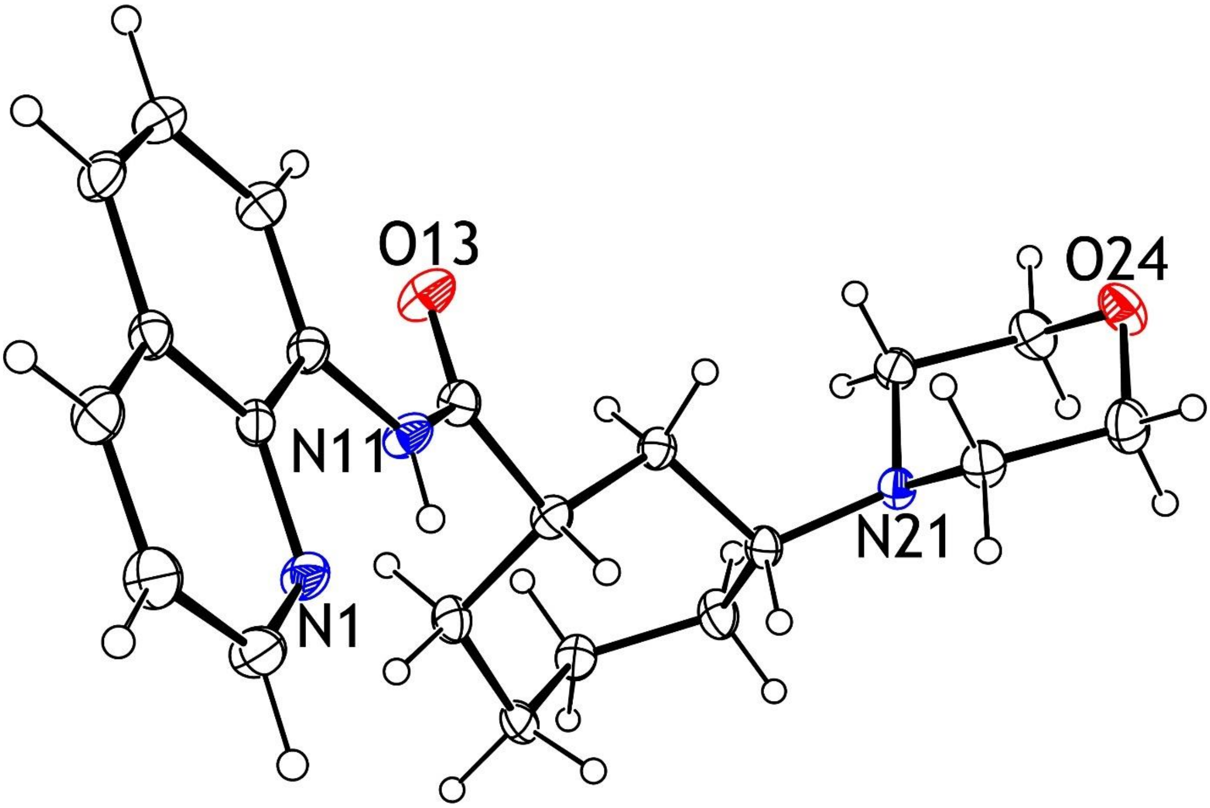

ORTEP representation (50% probability) of the crystal structure of **3i**

Supplementary Table 9. Crystal data and structure refinement for 3i.

Empirical formula	$C_{21} H_{27} N_3 O_2$	
Formula weight	353.45	
Temperature	173(2) K	
Wavelength	0.71073 Å	
Crystal system	Monoclinic	
Space group	$P2_1/c$	
Unit cell dimensions	a = 12.2984(7) Å	α = 90°
	b = 12.3285(6) Å	β = 100.4467(16)°
	c = 24.9341(13) Å	γ = 90°
Volume	3717.9(3) $Å^3$	
Z	8	
Density (calculated)	1.263 Mg/m^3	
Absorption coefficient	0.082 mm^{-1}	
F(000)	1520	
Crystal size	0.143 x 0.071 x 0.034 mm^3	
Theta range for data collection	2.571 to 26.364°.	

Index ranges	–13<=h<=15, –15<=k<=15, –31<=l<=31
Reflections collected	41225
Independent reflections	7566 [R(int) = 0.0888]
Completeness to theta = 25.242°	99.5 %
Absorption correction	Semi-empirical from equivalents
Max. and min. transmission	0.7454 and 0.6231
Refinement method	Full-matrix least-squares on F^2
Data / restraints / parameters	7566 / 0 / 487
Goodness-of-fit on F^2	1.073
Final R indices [I>2sigma(I)]	R1 = 0.0654, wR2 = 0.1174
R indices (all data)	R1 = 0.1034, wR2 = 0.1306
Largest diff. peak and hole	0.337 and –0.225 e·$Å^{-3}$

Supplementary Table 10. Atomic coordinates (x 10^4) and equivalent isotropic displacement parameters ($Å^2$ x 10^3) for 3i. U(eq) is defined as one third of the trace of the orthogonalized U^{ij} tensor.

	x	y	z	U(eq)
N(1)	9341(1)	7412(2)	7558(1)	28(1)
C(2)	9878(2)	6848(2)	7970(1)	33(1)
C(3)	11037(2)	6790(2)	8107(1)	36(1)
C(4)	11652(2)	7372(2)	7808(1)	32(1)
C(5)	11126(2)	8003(2)	7364(1)	25(1)
C(6)	9953(2)	7982(2)	7249(1)	23(1)
C(7)	9378(2)	8569(2)	6792(1)	25(1)
C(8)	9964(2)	9165(2)	6477(1)	29(1)
C(9)	11123(2)	9196(2)	6606(1)	32(1)
C(10)	11700(2)	8634(2)	7032(1)	30(1)
N(11)	8221(2)	8452(2)	6687(1)	30(1)
C(12)	7456(2)	8964(2)	6308(1)	28(1)
O(13)	7705(1)	9639(1)	5992(1)	41(1)
C(14)	6258(2)	8667(2)	6326(1)	26(1)
C(15)	5890(2)	9409(2)	6755(1)	31(1)
C(16)	4664(2)	9363(2)	6785(1)	31(1)
C(17)	3925(2)	9862(2)	6289(1)	32(1)
C(18)	3529(2)	9067(2)	5825(1)	35(1)
C(19)	4388(2)	8245(2)	5710(1)	26(1)
C(20)	5559(2)	8719(2)	5750(1)	26(1)
N(21)	3954(2)	7687(1)	5196(1)	25(1)
C(22)	3971(2)	8314(2)	4702(1)	31(1)
C(23)	3346(2)	7712(2)	4217(1)	38(1)
O(24)	3802(2)	6662(1)	4161(1)	41(1)
C(25)	3812(2)	6056(2)	4648(1)	40(1)
C(26)	4448(2)	6631(2)	5140(1)	32(1)
N(31)	5596(1)	2886(2)	2400(1)	26(1)
C(32)	5135(2)	2315(2)	1972(1)	31(1)
C(33)	3992(2)	2216(2)	1791(1)	33(1)
C(34)	3297(2)	2754(2)	2068(1)	30(1)
C(35)	3738(2)	3393(2)	2528(1)	24(1)
C(36)	4902(2)	3418(2)	2682(1)	23(1)
C(37)	5390(2)	4042(2)	3146(1)	25(1)

C(38)	4731(2)	4634(2)	3428(1)	27(1)
C(39)	3575(2)	4612(2)	3259(1)	30(1)
C(40)	3078(2)	4008(2)	2826(1)	28(1)
N(41)	6551(2)	3980(2)	3282(1)	29(1)
C(42)	7250(2)	4462(2)	3702(1)	28(1)
O(43)	6931(1)	5064(1)	4029(1)	37(1)
C(44)	8465(2)	4196(2)	3719(1)	30(1)
C(45)	8929(2)	4990(2)	3343(1)	36(1)
C(46)	10171(2)	4915(2)	3359(1)	37(1)
C(47)	10863(2)	5240(2)	3905(1)	35(1)
C(48)	11141(2)	4341(2)	4329(1)	35(1)
C(49)	10183(2)	3580(2)	4386(1)	28(1)
C(50)	9078(2)	4172(2)	4308(1)	30(1)
N(51)	10389(2)	2996(2)	4911(1)	35(1)
C(52)	11509(2)	2576(2)	5072(1)	47(1)
C(53)	11649(3)	2019(3)	5617(1)	68(1)
O(54)	10897(2)	1142(2)	5622(1)	67(1)
C(55)	9802(3)	1513(3)	5450(1)	62(1)
C(56)	9642(2)	2077(2)	4909(1)	45(1)

Supplementary Table 11. Bond lengths [Å] and angles [°] for 3i.

N(1)-C(2)	1.315(3)	C(18)-H(18B)	0.9900
N(1)-C(6)	1.366(3)	C(19)-N(21)	1.468(3)
C(2)-C(3)	1.405(3)	C(19)-C(20)	1.540(3)
C(2)-H(2)	0.9500	C(19)-H(19)	1.01(2)
C(3)-C(4)	1.359(3)	C(20)-H(20A)	0.9900
C(3)-H(3)	0.9500	C(20)-H(20B)	0.9900
C(4)-C(5)	1.411(3)	N(21)-C(26)	1.454(3)
C(4)-H(4)	0.9500	N(21)-C(22)	1.456(3)
C(5)-C(10)	1.415(3)	C(22)-C(23)	1.505(3)
C(5)-C(6)	1.419(3)	C(22)-H(22A)	0.9900
C(6)-C(7)	1.425(3)	C(22)-H(22B)	0.9900
C(7)-C(8)	1.372(3)	C(23)-O(24)	1.427(3)
C(7)-N(11)	1.406(3)	C(23)-H(23A)	0.9900
C(8)-C(9)	1.404(3)	C(23)-H(23B)	0.9900
C(8)-H(8)	0.9500	O(24)-C(25)	1.423(3)
C(9)-C(10)	1.356(3)	C(25)-C(26)	1.509(3)
C(9)-H(9)	0.9500	C(25)-H(25A)	0.9900
C(10)-H(10)	0.9500	C(25)-H(25B)	0.9900
N(11)-C(12)	1.362(3)	C(26)-H(26A)	0.9900
N(11)-H(11)	0.84(2)	C(26)-H(26B)	0.9900
C(12)-O(13)	1.223(3)	N(31)-C(32)	1.318(3)
C(12)-C(14)	1.526(3)	N(31)-C(36)	1.368(3)
C(14)-C(20)	1.535(3)	C(32)-C(33)	1.402(3)
C(14)-C(15)	1.536(3)	C(32)-H(32)	0.9500
C(14)-H(14)	0.99(2)	C(33)-C(34)	1.364(3)
C(15)-C(16)	1.524(3)	C(33)-H(33)	0.9500
C(15)-H(15A)	0.9900	C(34)-C(35)	1.414(3)
C(15)-H(15B)	0.9900	C(34)-H(34)	0.9500
C(16)-C(17)	1.525(3)	C(35)-C(36)	1.414(3)
C(16)-H(16A)	0.9900	C(35)-C(40)	1.416(3)
C(16)-H(16B)	0.9900	C(36)-C(37)	1.428(3)
C(17)-C(18)	1.528(3)	C(37)-C(38)	1.375(3)
C(17)-H(17A)	0.9900	C(37)-N(41)	1.409(3)
C(17)-H(17B)	0.9900	C(38)-C(39)	1.408(3)
C(18)-C(19)	1.528(3)	C(38)-H(38)	0.9500
C(18)-H(18A)	0.9900	C(39)-C(40)	1.362(3)

C(39)-H(39)	0.9500	C(56)-H(56A)	0.9900
C(40)-H(40)	0.9500	C(56)-H(56B)	0.9900
N(41)-C(42)	1.364(3)		
N(41)-H(41)	0.87(2)	C(2)-N(1)-C(6)	117.56(19)
C(42)-O(43)	1.219(3)	N(1)-C(2)-C(3)	124.1(2)
C(42)-C(44)	1.524(3)	N(1)-C(2)-H(2)	117.9
C(44)-C(50)	1.525(3)	C(3)-C(2)-H(2)	117.9
C(44)-C(45)	1.536(3)	C(4)-C(3)-C(2)	118.7(2)
C(44)-H(44)	1.03(3)	C(4)-C(3)-H(3)	120.6
C(45)-C(46)	1.524(3)	C(2)-C(3)-H(3)	120.6
C(45)-H(45A)	0.9900	C(3)-C(4)-C(5)	120.0(2)
C(45)-H(45B)	0.9900	C(3)-C(4)-H(4)	120.0
C(46)-C(47)	1.523(3)	C(5)-C(4)-H(4)	120.0
C(46)-H(46A)	0.9900	C(4)-C(5)-C(10)	123.8(2)
C(46)-H(46B)	0.9900	C(4)-C(5)-C(6)	117.0(2)
C(47)-C(48)	1.527(3)	C(10)-C(5)-C(6)	119.3(2)
C(47)-H(47A)	0.9900	N(1)-C(6)-C(5)	122.6(2)
C(47)-H(47B)	0.9900	N(1)-C(6)-C(7)	117.94(18)
C(48)-C(49)	1.532(3)	C(5)-C(6)-C(7)	119.43(19)
C(48)-H(48A)	0.9900	C(8)-C(7)-N(11)	124.6(2)
C(48)-H(48B)	0.9900	C(8)-C(7)-C(6)	119.61(19)
C(49)-N(51)	1.477(3)	N(11)-C(7)-C(6)	115.78(19)
C(49)-C(50)	1.524(3)	C(7)-C(8)-C(9)	120.0(2)
C(49)-H(49)	1.00(2)	C(7)-C(8)-H(8)	120.0
C(50)-H(50A)	0.9900	C(9)-C(8)-H(8)	120.0
C(50)-H(50B)	0.9900	C(10)-C(9)-C(8)	122.0(2)
N(51)-C(56)	1.458(3)	C(10)-C(9)-H(9)	119.0
N(51)-C(52)	1.458(3)	C(8)-C(9)-H(9)	119.0
C(52)-C(53)	1.504(4)	C(9)-C(10)-C(5)	119.6(2)
C(52)-H(52A)	0.9900	C(9)-C(10)-H(10)	120.2
C(52)-H(52B)	0.9900	C(5)-C(10)-H(10)	120.2
C(53)-O(54)	1.425(4)	C(12)-N(11)-C(7)	129.0(2)
C(53)-H(53A)	0.9900	C(12)-N(11)-H(11)	118.3(17)
C(53)-H(53B)	0.9900	C(7)-N(11)-H(11)	112.6(17)
O(54)-C(55)	1.413(4)	O(13)-C(12)-N(11)	122.8(2)
C(55)-C(56)	1.498(4)	O(13)-C(12)-C(14)	122.5(2)
C(55)-H(55A)	0.9900	N(11)-C(12)-C(14)	114.70(19)
C(55)-H(55B)	0.9900	C(12)-C(14)-C(20)	110.12(18)

C(12)-C(14)-C(15)	106.56(18)	C(14)-C(20)-H(20B)	108.8
C(20)-C(14)-C(15)	116.11(18)	C(19)-C(20)-H(20B)	108.8
C(12)-C(14)-H(14)	110.3(14)	H(20A)-C(20)-H(20B)	107.7
C(20)-C(14)-H(14)	106.7(14)	C(26)-N(21)-C(22)	108.91(18)
C(15)-C(14)-H(14)	107.0(14)	C(26)-N(21)-C(19)	114.19(17)
C(16)-C(15)-C(14)	115.53(18)	C(22)-N(21)-C(19)	115.54(17)
C(16)-C(15)-H(15A)	108.4	N(21)-C(22)-C(23)	109.49(19)
C(14)-C(15)-H(15A)	108.4	N(21)-C(22)-H(22A)	109.8
C(16)-C(15)-H(15B)	108.4	C(23)-C(22)-H(22A)	109.8
C(14)-C(15)-H(15B)	108.4	N(21)-C(22)-H(22B)	109.8
H(15A)-C(15)-H(15B)	107.5	C(23)-C(22)-H(22B)	109.8
C(15)-C(16)-C(17)	113.26(19)	H(22A)-C(22)-H(22B)	108.2
C(15)-C(16)-H(16A)	108.9	O(24)-C(23)-C(22)	111.92(19)
C(17)-C(16)-H(16A)	108.9	O(24)-C(23)-H(23A)	109.2
C(15)-C(16)-H(16B)	108.9	C(22)-C(23)-H(23A)	109.2
C(17)-C(16)-H(16B)	108.9	O(24)-C(23)-H(23B)	109.2
H(16A)-C(16)-H(16B)	107.7	C(22)-C(23)-H(23B)	109.2
C(16)-C(17)-C(18)	114.7(2)	H(23A)-C(23)-H(23B)	107.9
C(16)-C(17)-H(17A)	108.6	C(25)-O(24)-C(23)	109.58(18)
C(18)-C(17)-H(17A)	108.6	O(24)-C(25)-C(26)	111.7(2)
C(16)-C(17)-H(17B)	108.6	O(24)-C(25)-H(25A)	109.3
C(18)-C(17)-H(17B)	108.6	C(26)-C(25)-H(25A)	109.3
H(17A)-C(17)-H(17B)	107.6	O(24)-C(25)-H(25B)	109.3
C(17)-C(18)-C(19)	115.50(19)	C(26)-C(25)-H(25B)	109.3
C(17)-C(18)-H(18A)	108.4	H(25A)-C(25)-H(25B)	107.9
C(19)-C(18)-H(18A)	108.4	N(21)-C(26)-C(25)	109.50(19)
C(17)-C(18)-H(18B)	108.4	N(21)-C(26)-H(26A)	109.8
C(19)-C(18)-H(18B)	108.4	C(25)-C(26)-H(26A)	109.8
H(18A)-C(18)-H(18B)	107.5	N(21)-C(26)-H(26B)	109.8
N(21)-C(19)-C(18)	108.63(18)	C(25)-C(26)-H(26B)	109.8
N(21)-C(19)-C(20)	115.02(18)	H(26A)-C(26)-H(26B)	108.2
C(18)-C(19)-C(20)	113.92(19)	C(32)-N(31)-C(36)	117.14(19)
N(21)-C(19)-H(19)	105.2(14)	N(31)-C(32)-C(33)	124.4(2)
C(18)-C(19)-H(19)	107.2(14)	N(31)-C(32)-H(32)	117.8
C(20)-C(19)-H(19)	106.2(14)	C(33)-C(32)-H(32)	117.8
C(14)-C(20)-C(19)	113.85(18)	C(34)-C(33)-C(32)	118.6(2)
C(14)-C(20)-H(20A)	108.8	C(34)-C(33)-H(33)	120.7
C(19)-C(20)-H(20A)	108.8	C(32)-C(33)-H(33)	120.7

C(33)-C(34)-C(35)	119.8(2)	H(45A)-C(45)-H(45B)	107.5
C(33)-C(34)-H(34)	120.1	C(47)-C(46)-C(45)	113.8(2)
C(35)-C(34)-H(34)	120.1	C(47)-C(46)-H(46A)	108.8
C(34)-C(35)-C(36)	117.0(2)	C(45)-C(46)-H(46A)	108.8
C(34)-C(35)-C(40)	123.4(2)	C(47)-C(46)-H(46B)	108.8
C(36)-C(35)-C(40)	119.6(2)	C(45)-C(46)-H(46B)	108.8
N(31)-C(36)-C(35)	123.0(2)	H(46A)-C(46)-H(46B)	107.7
N(31)-C(36)-C(37)	117.77(19)	C(46)-C(47)-C(48)	116.5(2)
C(35)-C(36)-C(37)	119.2(2)	C(46)-C(47)-H(47A)	108.2
C(38)-C(37)-N(41)	124.8(2)	C(48)-C(47)-H(47A)	108.2
C(38)-C(37)-C(36)	120.1(2)	C(46)-C(47)-H(47B)	108.2
N(41)-C(37)-C(36)	115.13(19)	C(48)-C(47)-H(47B)	108.2
C(37)-C(38)-C(39)	119.5(2)	H(47A)-C(47)-H(47B)	107.3
C(37)-C(38)-H(38)	120.2	C(47)-C(48)-C(49)	115.36(19)
C(39)-C(38)-H(38)	120.2	C(47)-C(48)-H(48A)	108.4
C(40)-C(39)-C(38)	122.2(2)	C(49)-C(48)-H(48A)	108.4
C(40)-C(39)-H(39)	118.9	C(47)-C(48)-H(48B)	108.4
C(38)-C(39)-H(39)	118.9	C(49)-C(48)-H(48B)	108.4
C(39)-C(40)-C(35)	119.4(2)	H(48A)-C(48)-H(48B)	107.5
C(39)-C(40)-H(40)	120.3	N(51)-C(49)-C(50)	110.56(19)
C(35)-C(40)-H(40)	120.3	N(51)-C(49)-C(48)	111.67(19)
C(42)-N(41)-C(37)	129.2(2)	C(50)-C(49)-C(48)	112.2(2)
C(42)-N(41)-H(41)	118.1(16)	N(51)-C(49)-H(49)	107.2(14)
C(37)-N(41)-H(41)	112.7(16)	C(50)-C(49)-H(49)	107.4(14)
O(43)-C(42)-N(41)	123.0(2)	C(48)-C(49)-H(49)	107.5(14)
O(43)-C(42)-C(44)	123.1(2)	C(49)-C(50)-C(44)	113.88(19)
N(41)-C(42)-C(44)	114.0(2)	C(49)-C(50)-H(50A)	108.8
C(42)-C(44)-C(50)	110.03(19)	C(44)-C(50)-H(50A)	108.8
C(42)-C(44)-C(45)	108.45(19)	C(49)-C(50)-H(50B)	108.8
C(50)-C(44)-C(45)	115.29(19)	C(44)-C(50)-H(50B)	108.8
C(42)-C(44)-H(44)	110.0(14)	H(50A)-C(50)-H(50B)	107.7
C(50)-C(44)-H(44)	104.1(14)	C(56)-N(51)-C(52)	106.8(2)
C(45)-C(44)-H(44)	108.9(14)	C(56)-N(51)-C(49)	111.66(18)
C(46)-C(45)-C(44)	115.25(19)	C(52)-N(51)-C(49)	114.7(2)
C(46)-C(45)-H(45A)	108.5	N(51)-C(52)-C(53)	111.0(2)
C(44)-C(45)-H(45A)	108.5	N(51)-C(52)-H(52A)	109.4
C(46)-C(45)-H(45B)	108.5	C(53)-C(52)-H(52A)	109.4
C(44)-C(45)-H(45B)	108.5	N(51)-C(52)-H(52B)	109.4

C(53)-C(52)-H(52B)	109.4
H(52A)-C(52)-H(52B)	108.0
O(54)-C(53)-C(52)	112.7(3)
O(54)-C(53)-H(53A)	109.0
C(52)-C(53)-H(53A)	109.0
O(54)-C(53)-H(53B)	109.0
C(52)-C(53)-H(53B)	109.0
H(53A)-C(53)-H(53B)	107.8
C(55)-O(54)-C(53)	109.6(2)
O(54)-C(55)-C(56)	112.7(3)
O(54)-C(55)-H(55A)	109.1
C(56)-C(55)-H(55A)	109.1
O(54)-C(55)-H(55B)	109.1
C(56)-C(55)-H(55B)	109.1
H(55A)-C(55)-H(55B)	107.8
N(51)-C(56)-C(55)	112.1(2)
N(51)-C(56)-H(56A)	109.2
C(55)-C(56)-H(56A)	109.2
N(51)-C(56)-H(56B)	109.2
C(55)-C(56)-H(56B)	109.2
H(56A)-C(56)-H(56B)	107.9

Symmetry transformations used to generate equivalent atoms:

Supplementary Table 12. Anisotropic displacement parameters ($Å^2$ x 10^3) for 3i. The anisotropic displacement factor exponent takes the form: $-2\pi^2[h^2a^{*2}U^{11} + ... + 2hka^*b^*U^{12}]$

	U^{11}	U^{22}	U^{33}	U^{23}	U^{13}	U^{12}
N(1)	19(1)	35(1)	28(1)	3(1)	2(1)	-2(1)
C(2)	27(1)	42(2)	29(1)	10(1)	0(1)	-4(1)
C(3)	26(1)	46(2)	31(1)	6(1)	-4(1)	2(1)
C(4)	17(1)	43(2)	34(1)	-3(1)	0(1)	2(1)
C(5)	18(1)	29(1)	28(1)	-6(1)	3(1)	1(1)
C(6)	19(1)	23(1)	24(1)	-5(1)	2(1)	-1(1)
C(7)	19(1)	26(1)	28(1)	-2(1)	3(1)	1(1)
C(8)	25(1)	28(1)	33(1)	3(1)	5(1)	2(1)
C(9)	28(1)	31(1)	39(1)	2(1)	11(1)	-2(1)
C(10)	18(1)	35(1)	37(1)	-5(1)	6(1)	-4(1)
N(11)	18(1)	38(1)	32(1)	9(1)	1(1)	-2(1)
C(12)	24(1)	30(1)	28(1)	1(1)	1(1)	1(1)
O(13)	27(1)	46(1)	47(1)	20(1)	-1(1)	-3(1)
C(14)	20(1)	29(1)	27(1)	2(1)	0(1)	1(1)
C(15)	27(1)	34(1)	29(1)	-4(1)	-1(1)	-1(1)
C(16)	34(1)	31(1)	29(1)	-11(1)	10(1)	-2(1)
C(17)	25(1)	32(1)	40(1)	-6(1)	6(1)	2(1)
C(18)	24(1)	41(2)	38(1)	-10(1)	0(1)	2(1)
C(19)	23(1)	28(1)	24(1)	-1(1)	-1(1)	-1(1)
C(20)	23(1)	25(1)	28(1)	-1(1)	3(1)	0(1)
N(21)	29(1)	23(1)	22(1)	2(1)	0(1)	-1(1)
C(22)	33(1)	31(1)	27(1)	2(1)	2(1)	-1(1)
C(23)	42(2)	41(2)	27(1)	2(1)	-3(1)	6(1)
O(24)	54(1)	41(1)	25(1)	-4(1)	3(1)	5(1)
C(25)	53(2)	28(1)	37(2)	-2(1)	3(1)	-1(1)
C(26)	35(1)	29(1)	29(1)	2(1)	2(1)	1(1)
N(31)	22(1)	29(1)	29(1)	2(1)	5(1)	1(1)
C(32)	32(1)	32(1)	31(1)	-3(1)	9(1)	1(1)
C(33)	33(1)	32(1)	32(1)	-5(1)	1(1)	-3(1)
C(34)	23(1)	30(1)	35(1)	0(1)	0(1)	-5(1)
C(35)	22(1)	23(1)	26(1)	4(1)	2(1)	-2(1)
C(36)	22(1)	23(1)	25(1)	5(1)	3(1)	-1(1)
C(37)	21(1)	26(1)	26(1)	7(1)	1(1)	0(1)

C(38)	26(1)	28(1)	26(1)	0(1)	4(1)	-1(1)
C(39)	26(1)	28(1)	36(1)	-3(1)	9(1)	2(1)
C(40)	16(1)	31(1)	38(1)	2(1)	5(1)	-4(1)
N(41)	20(1)	38(1)	29(1)	-4(1)	1(1)	3(1)
C(42)	26(1)	30(1)	26(1)	3(1)	0(1)	-2(1)
O(43)	29(1)	42(1)	37(1)	-9(1)	-6(1)	3(1)
C(44)	24(1)	36(1)	27(1)	2(1)	0(1)	-2(1)
C(45)	34(1)	42(2)	30(1)	7(1)	2(1)	-2(1)
C(46)	34(1)	45(2)	33(1)	9(1)	10(1)	-5(1)
C(47)	27(1)	35(1)	46(2)	-1(1)	11(1)	-6(1)
C(48)	29(1)	42(2)	33(1)	0(1)	5(1)	2(1)
C(49)	30(1)	31(1)	22(1)	0(1)	-2(1)	0(1)
C(50)	26(1)	36(1)	28(1)	4(1)	5(1)	-1(1)
N(51)	30(1)	43(1)	30(1)	10(1)	0(1)	7(1)
C(52)	42(2)	52(2)	42(2)	7(1)	-7(1)	15(1)
C(53)	58(2)	83(3)	54(2)	20(2)	-11(2)	22(2)
O(54)	80(2)	56(1)	60(1)	29(1)	-4(1)	16(1)
C(55)	68(2)	66(2)	52(2)	32(2)	6(2)	8(2)
C(56)	51(2)	39(2)	39(2)	9(1)	-7(1)	-1(1)

Supplementary Table 13. Hydrogen coordinates (x 10^4) and isotropic displacement parameters ($Å^2$ x 10^3) for 3i.

	x	y	z	U(eq)
H(2)	9457	6455	8188	40
H(3)	11383	6354	8403	43
H(4)	12436	7353	7898	38
H(8)	9585	9557	6171	34
H(9)	11515	9624	6387	38
H(10)	12485	8664	7107	36
H(11)	8000(20)	8030(20)	6904(10)	35
H(14)	6213(19)	7910(20)	6455(9)	39
H(15A)	6320	9215	7117	37
H(15B)	6081	10166	6678	37
H(16A)	4542	9750	7117	37
H(16B)	4447	8596	6819	37
H(17A)	4336	10456	6149	38
H(17B)	3270	10185	6408	38
H(18A)	3271	9489	5488	42
H(18B)	2884	8665	5910	42
H(19)	4445(19)	7670(20)	6000(10)	39
H(20A)	5947	8319	5497	31
H(20B)	5496	9486	5630	31
H(22A)	4745	8430	4655	37
H(22B)	3627	9032	4732	37
H(23A)	2564	7635	4258	45
H(23B)	3364	8138	3882	45
H(25A)	4151	5337	4611	48
H(25B)	3042	5936	4700	48
H(26A)	4434	6193	5472	38
H(26B)	5229	6722	5099	38
H(32)	5610	1946	1772	38
H(33)	3707	1783	1483	39
H(34)	2519	2701	1953	36
H(38)	5053	5055	3735	32
H(39)	3127	5032	3453	36

H(40)	2295	3999	2724	34
H(41)	6830(20)	3570(20)	3061(10)	35
H(44)	8540(20)	3420(20)	3581(10)	44
H(45A)	8546	4861	2963	43
H(45B)	8750	5738	3442	43
H(46A)	10359	4161	3275	44
H(46B)	10369	5389	3071	44
H(47A)	10465	5820	4065	42
H(47B)	11566	5555	3836	42
H(48A)	11415	4680	4688	42
H(48B)	11752	3901	4234	42
H(49)	10130(20)	3020(20)	4090(10)	43
H(50A)	9208	4928	4439	36
H(50B)	8602	3817	4537	36
H(52A)	12044	3181	5092	57
H(52B)	11667	2056	4794	57
H(53A)	12415	1742	5715	81
H(53B)	11536	2555	5898	81
H(55A)	9610	2019	5727	75
H(55B)	9290	888	5425	75
H(56A)	9769	1553	4625	54
H(56B)	8868	2335	4814	54

Supplementary Table 14. Hydrogen bonds for 3i[Å and °].

D-H...A	d(D-H)	d(H...A)	d(D...A)	<(DHA)
N(11)-H(11)...N(1)	0.84(2)	2.23(2)	2.678(3)	114(2)
N(41)-H(41)...N(31)	0.87(2)	2.20(2)	2.666(3)	113.4(19)

Symmetry transformations used to generate equivalent atoms:

Appendix III

DFT Calculation Data

Supplementary Table 15. Computed energy components for optimized structures

	E(SCF)/(eV)	ZPE/(kcal/mol)	S(gas)/(cal/mol ·K)	G(solv)/(kcal/mol)
	cc-pVTZ(-f) /LACV3P**	LACVP**	LACVP**	LACVP**
**2a**	-19255.479	144.618	113.016	-8.53
**L8**	-37559.211	233.556	170.029	-10.69
**A**	-26001.842	176.641	148.531	-25.97
**A'**	-63561.910	410.590	267.558	-28.94
**TS1**	-45257.621	320.234	216.118	-28.78
**B**	-45260.582	321.402	219.174	-25.48
**TS2**	-45259.762	321.282	216.136	-24.81
**C**	-29211.799	256.195	167.562	-22.89
**TS3**	-63560.82	407.19	267.363	-28.95
**D**	-26001.184	173.613	156.427	-30.85
**TS4**	-26000.754	173.664	148.642	-29.41
**E**	-26001.467	176.316	152.198	-27.64
**TS5**	-45257.582	319.53	218.512	-29.4
**F**	-45260.469	320.784	222.247	-26.1
**TS6**	-45260.027	321.424	214.859	-24.37
**G**	-29211.627	256.341	169.757	-24.22
**$(PAr_3)_2$Ni–OBz**	-91168.531	535.493	333.1	-22.13
**$(PAr_3)_2$Ni–H**	-79743.703	472.799	294.453	-25.77
**s-A-dis**	-20850.637	176.834	126.765	-8.65
**s-A**	-63034.953	416.789	252.134	-18.64
**s-A-TS**	-63034.68	416.932	249.693	-15.33
**s-B**	-63035.492	419.033	259.97	-16.48

**s-B'**	-25475.064	185.106	133.043	-10.39
**s-B-TS**	-63034.594	415.913	254.839	-15.32
**s-C-dis**	-20850.76	176.781	134.049	-8.25
**s-C**	-63034.867	415.093	253.541	-21.3
**s-D**	-63035.371	419.263	256.849	-14.05
**s-D'**	-25475.193	184.486	135.737	-11.78
**s-E-dim**	-52004.855	347.883	265.839	-39.67
**s-E**	-26001.176	172.54	160.302	-34.56
**s-E-TS**	-26001	173.169	144.778	-26.2
**s-F-dim**	-52005.059	349.141	257.169	-37.25
**s-F**	-26001.471	172.94	149.021	-28.97
**s-F-TS**	-26001.061	173.2	144.819	-25.62

VI. Supplementary References.

[1] Lv, H., Kang, H., Zhou,B., Xue, X., Engle, K. M. & Zhao, D. Nickel-catalyzed intermolecular oxidative Heck arylation driven by transfer hydrogenation. *Nat. Commun.* **10**, 5025 (2019).

[2] Hu, P. & Bach, T. Synthesis of Alkyl-Substituted Pyridines by Directed Pd(II)-Catalyzed C–H Activation of Alkanoic Amides. *Synlett* **26**, 2853–2857 (2015).

[3] Conti, P., Tamborini, L., Pinto, A., Sola, L., Ettari, R., Mercurio, C. & Micheli, C. D. Design and synthesis of novel isoxazole-based HDAC inhibitors. *Eur. J. Med. Chem.* **45**, 4331–4338 (2010).

[4] Conti, P., Tamborini, L., Pinto, A., Sola, L., Ettari, R., Mercurio, C. & De Micheli, C. Design and synthesis of novel isoxazole-based HDAC inhibitors. *Eur. J. Med. Chem*. **45**, 4331–4338 (2010).

[5] Shi, P., Wang, J., Gan, Z., Zhang, J., Zeng, R. & Zhao, Y. A practical copper-catalyzed approach to β-lactams *via* radical carboamination of alkenyl carbonyl compounds. *Chem. Commun.* **55**, 10523–10526 (2019).

[6] Yang, K. S., Gurak, J. A., Liu, Z. & Engle, K. M. Catalytic, Regioselective Hydrocarbofunctionalization of Unactivated Alkenes with Diverse C–H Nucleophiles. *J. Am. Chem. Soc*. **138***,* 14705–14712 (2016).

[7] Jagtap, P. R., Ford, L., Deister, E., Pohl, R., Císařová, I., Hodek, J., Weber, J., Mackman, R., Bahador, G. & Jahn, U. Highly Functionalized and Potent Antiviral Cyclopentane Derivatives Formed by a Tandem Process Consisting of Organometallic, Transition-Metal-Catalyzed, and Radical Reaction Steps. *Chem. Eur. J.* **20**, 10298–10304 (2014).

[8] Courchay, F. C., Baughman, T. W. & Wagener, K. B. Understanding the effect of allylic methyls in olefin cross-metathesis. *J. of Organometallic Chem*. **691**, 585–594 (2006).

[9] Liu, M., Yang, P., Karunananda, M. K., Wang, Y., Liu, P. & Engle, K. M. C(alkenyl)–H Activation via Six-Membered Palladacycles: Catalytic 1,3-Diene Synthesis. *J. Am. Chem. Soc.* **140**, 5805–5813 (2018).

[10] Ortgies, S., Rieger, R., Rdoe, K., Koszinowski, K., Kind, J., Thiele, C. M., Rehbein, J. & Breder, A. Mechanistic and Synthetic Investigations on the Dual Selenium-π-Acid/Photoredox Catalysis in the Context of the Aerobic Dehydrogenative Lactonization of Alkenoic Acids. *ACS Catal.* **7**, 7578–7586 (2017).

[11] Kier, M. J., Leon, R. M., O'Rourke, N. F., Rheingold, A. L. & Micalizio, G. C. Synthesis of Highly Oxygenated Carbocycles by Stereoselective Coupling of Alkynes to 1,3- and 1,4-Dicarbonyl Systems. *J. Am. Chem. Soc.* **139**, 12374–12377 (2017).

[12] Xu, J. & Hu, L. Asymmetric one-pot synthesis of five- and six-membered lactones *via* dynamic covalent kinetic resolution: Exploring the regio- and stereoselectivities of lipase. *Tetrahedron Lett.* **60**,

868 (2019).

[13] Kelly, D. P., Giansiracusa, J. J., Leslie D. R., McKern, I. D. & Sinclair, G. C. ^{13}C–^{1}H Coupling Constants in Carbocations. 5.[1] Trishomocyclopropenium Cations Generated from Bicyclo[3.1.0]hex-3-yl, Tricyclo[3.2.1.0^{2,4}]oct-8-yl, and Pentacyclo[4.3.0.0^{2,4}.0^{3,8}.0^{5,7}]non-9-yl Precursors. *J. Org. Chem.* **53**, 2497–2504 (1988).

[14] Zhang, Z. -Z., Han, Y. -Q., Zhan, B. -B., Wang, S. & Shi, B. -F. Synthesis of Bicyclo[*n*.1.0]alkanes by a Cobalt-Catalyzed Multiple C(sp^3)–H Activation Strategy. *Angew. Chem. Int. Ed.* **56**, 13145–13149 (2017).

[15] Roman, M., Cannizzo, C., Pinault, T., Isare, B., Andrioletti, B., Schoot, P. & Bouteiller, L. Supramolecular Balance: Using Cooperativity To Amplify Weak Interactions. *J. Am. Chem. Soc.* **132**, 16818–16824 (2010).

[16] Archambeau, A. & Rovis, T. Rhodium(III)-Catalyzed Allylic C(sp^3)–H Activation of Alkenyl Sulfonamides: Unexpected Formation of Azabicycles. *Angew. Chem. Int. Ed.* **54**, 13337–13340 (2015).

[17] Dong, Z. & Dong, G. *Ortho vs Ipso*: Site-Selective Pd and Norbornene-Catalyzed Arene C–H Amination Using Aryl Halides. *J. Am. Chem. Soc.* **135**, 18350–18353 (2013).

[18] Yotphan, S., Beukeaw, D. & Reutrakul, V. Synthesis of 2-aminobenzoxazoles via copper-catalyzed electrophilic amination of benzoxazoles with *O*-benzoyl hydroxylamines. *Tetrahedron* 69, 6627–6633 (2013).

[19] Shi, H., Babinski, D. J. & Ritter, T. Modular C–H Functionalization Cascade of Aryl Iodides. *J. Am. Chem. Soc.* **137**, 3775–3778.

[20] van der Puyl, V. A., Derosa, J.& Engle, K. M. *A*CS Cata. irected, Nickel-Catalyzed Umpolung 1,2-Carboamination of Alkenyl Carbonyl Compounds. **9**, 224–229 (2019).

[21] Graßl, S., Chen, Y.-H., Hamze, C., Tüllmann, C. P. & Knochel, P. Late Stage Functionalization of Secondary Amines via a Cobalt-Catalyzed Electrophilic Amination of Organozinc Reagents. *Org. Lett.* **21**, 494–497 (2019).

[22] Yao, Z.-L., Wang, L., Shao, N.-Q., Guo, Y.-L. & Wang, D.-H. Copper-Catalyzed *ortho*-Selective Dearomative C–N Coupling of Simple Phenols with *O*-Benzoylhydroxylamines. *ACS Catal.* **9**, 7343–7349 (2019).

[23] Dhanju, S., Blazejewski, B. W. & Crich, D. Synthesis of Trialkylhydroxylamines by Stepwise Reduction of *O*-Acyl *N*,*N*-Disubstituted Hydroxylamines: Substituent Effects on the Reduction of *O*-(1-Acyloxyalkyl)hydroxylamines and on the Conformational Dynamics of *N*-Alkoxypiperidines. *J. Org. Chem.* **82**, 5345–5353 (2017).

[24] Zhao, H., Chen, X., Jiang, H. & Zhang, M. Copper-catalysed dehydrogenative α-C(sp^3)–H amination of tetrahydroquinolines with *O*-benzoyl hydroxylamines. *Org. Chem. Front.* **5**, 539–543

(2018).
[25] Cheng, W. -M., Shang, R., Zhao, B., Zhao, B., Xing, W. -L. & Fu, Y. Isonicotinate Ester Catalyzed Decarboxylative Borylation of (Hetero)Aryl and Alkenyl Carboxylic Acids through *N*-Hydroxyphthalimide Esters. *Org. Lett.* **19**, 4291–4294 (2017).
[26] Jeon, J., Lee, C., Seo, H. & Hong, S. NiH-Catalyzed Proximal-Selective Hydroamination of Unactivated Alkenes. *J. Am. Chem. Soc.* **142**, 20470–20480 (2020).
[27] Rucker, R. P., Whittaker, A. M., Dang, H. & Lalic, G. Synthesis of Hindered Anilines: Copper-Catalyzed Electrophilic Amination of Aryl Boronic Esters. *Angew. Chem. Int. Ed.* **51**, 3953–3956 (2012).
[28] Luo, B., Gao, J.-M. & Lautens, M. Palladium-Catalyzed Norbornene-Mediated Tandem Amination/Cyanation Reaction: A Method for the Synthesis of *ortho*-Aminated Benzonitriles. *Org. Lett.* **18**, 4166–4169 (2016).
[29] An, X.-D., Jiao, Y.-Y., Zhang, H., Gao, Y. & Yu, S. Photoredox-Induced Radical Relay toward Functionalized β-Amino Alcohol Derivatives. *Org. Lett.* **20**, 401–404 (2018).
[30] Parr, R. G. & Yang, W. *Density Functional Theory of Atoms and Molecules* (Oxford Univ. Press, Oxford, 1989).
[31] Bochevarov, A. D., Harder, E., Hughes, T. F., Greenwood, J. R., Braden, D. A., Philipp, D. M., Rinaldo, D., Halls, M. D., Zhang, J. & Friesner, R. A. Jaguar: A high-performance quantum chemistry software program with strengths in life and materials sciences. *Int. J. Quantum Chem.* **113**, 2110–2142 (2013).
[32] Becke, A. D. A new mixing of Hartree-Fock and local density-functional theories. *J. Chem. Phys.* **98**, 1372 (1993).
[33] Dunning, T.H., Jr. Gaussian basis sets for use in correlated molecular calculations. I. The a toms boron through neon and hydrogen. *J. Chem. Phys.* **90**, 1007 (1989).
[34] Marten, B., Kim, K., Cortis, C., Friesner, R. A., Murphy, R. B., Ringnalda, M. N., Sitkoff, D. & Honig, B. New Model for Calculation of Solvation Free Energies: Correction of Self-Consisten t Reaction Field Continuum Dielectric Theory for Short-Range Hydrogen-Bonding Effects. *J. Phys. Chem.* **100**, 11775–11788 (1996).
[35] Edinger, S. R., Cortis, C., Shenkin, P. S. & Friesner, R. A. Solvation Free Energies of Peptides: Comparison of Approximate Continuum Solvation Models with Accurate Solution of the Poiss on−Boltzmann Equation. *J. Phys. Chem. B* **101**, 1190–1197 (1997).
[36] Friedrichs, M., Zhou, R., Edinger, S. R. & Friesner, R. A. Poisson−Boltzmann Analytical Gradi ents for Molecular Modeling Calculations. *J. Phys. Chem. B* **103**, 3057–3061 (1999).
