# Supplementary material for: γ-Selective C(sp3)–H amination via controlled migratory hydroamination
Source: Nat Commun. 2021 Sep 27;12:5657. doi: 10.1038/s41467-021-25696-z (PMC8476554; doi:10.1038/s41467-021-25696-z)
Supplement: Supplementary file 2 — Description of Additional Supplementary Files [file 41467_2021_25696_MOESM2_ESM.docx]

Description of Additional Supplementary Files

File Name: Supplementary Data 1

Description: CIF file - XRD data of **3i**

File Name: Supplementary Data 2

Description: CheckCIF – XRD data of **3i**

File Name: Supplementary Data 3

Description: Cartesian coordinates of the optimized geometries

File Name: Supplementary Data 4

Description: Vibrational frequencies (in cm-1) of the optimized structures
