# Supplementary material for: γ-Selective C(sp3)–H amination via controlled migratory hydroamination
Source: Nat Commun. 2021 Sep 27;12:5657. doi: 10.1038/s41467-021-25696-z (PMC8476554; doi:10.1038/s41467-021-25696-z)

checkCIF/PLATON report

Structure factors have been supplied for datablock(s) A21051O_014

THIS REPORT IS FOR GUIDANCE ONLY. IF USED AS PART OF A REVIEW PROCEDURE FOR PUBLICATION, IT SHOULD NOT REPLACE THE EXPERTISE OF AN EXPERIENCED CRYSTALLOGRAPHIC REFEREE.

No syntax errors found. CIF dictionary Interpreting this report

Datablock: A21051O_014

```
Bond precision:  C-C = 0.0031 A          Wavelength=0.71073

Cell:           a=12.2984(7)    b=12.3285(6)          c=24.9341(13)
                alpha=90        beta=100.4467(16)     gamma=90
Temperature:    173 K

                Calculated                  Reported
Volume          3717.9(3)                   3717.9(3)
Space group     P 21/c                      P 21/c
Hall group      -P 2ybc                     -P 2ybc
Moiety formula  C21 H27 N3 O2               C21 H27 N3 O2
Sum formula     C21 H27 N3 O2               C21 H27 N3 O2
Mr              353.46                      353.45
Dx,g cm-3       1.263                       1.263
Z               8                           8
Mu (mm-1)       0.082                       0.082
F000            1520.0                      1520.0
F000'           1520.59
h,k,lmax        15,15,31                    15,15,31
Nref            7611                        7566
Tmin,Tmax       0.993,0.997                 0.623,0.745
Tmin'           0.988

Correction method= # Reported T Limits: Tmin=0.623 Tmax=0.745
AbsCorr = MULTI-SCAN

Data completeness= 0.994          Theta(max)= 26.364

R(reflections)= 0.0654( 5243)     wR2(reflections)= 0.1306( 7566)

S = 1.073                  Npar= 487
```

The following ALERTS were generated. Each ALERT has the format
test-name_ALERT_alert-type_alert-level.
Click on the hyperlinks for more details of the test.

Alert level C

```
PLAT410_ALERT_2_C Short Intra H...H Contact  H50B     ..H56B     .        1.96 Ang.
                                                      x,y,z  =      1_555 Check
PLAT906_ALERT_3_C Large K Value in the Analysis of Variance ......     15.434 Check
PLAT906_ALERT_3_C Large K Value in the Analysis of Variance ......      2.953 Check
PLAT910_ALERT_3_C Missing # of FCF Reflection(s) Below Theta(Min).          7 Note
PLAT911_ALERT_3_C Missing FCF Refl Between Thmin & STh/L=    0.600         28 Report
PLAT913_ALERT_3_C Missing # of Very Strong Reflections in FCF ....         10 Note
```

Alert level G

```
PLAT398_ALERT_2_G Deviating  C-O-C    Angle From 120 for O24            109.6 Degree
PLAT398_ALERT_2_G Deviating  C-O-C    Angle From 120 for O54            109.6 Degree
PLAT793_ALERT_4_G Model has Chirality at C14         (Centro SPGR)          S Verify
PLAT793_ALERT_4_G Model has Chirality at C19         (Centro SPGR)          R Verify
PLAT793_ALERT_4_G Model has Chirality at C44         (Centro SPGR)          S Verify
PLAT793_ALERT_4_G Model has Chirality at C49         (Centro SPGR)          R Verify
PLAT883_ALERT_1_G No Info/Value for _atom_sites_solution_primary .     Please Do !
PLAT912_ALERT_4_G Missing # of FCF Reflections Above STh/L=  0.600         10 Note
PLAT933_ALERT_2_G Number of OMIT Records in Embedded .res File ...         11 Note
PLAT978_ALERT_2_G Number C-C Bonds with Positive Residual Density.          7 Info
```

   0 **ALERT level A** = Most likely a serious problem - resolve or explain
   0 **ALERT level B** = A potentially serious problem, consider carefully
   6 **ALERT level C** = Check. Ensure it is not caused by an omission or oversight
  10 **ALERT level G** = General information/check it is not something unexpected

```
   1 ALERT type 1 CIF construction/syntax error, inconsistent or missing data
   5 ALERT type 2 Indicator that the structure model may be wrong or deficient
   5 ALERT type 3 Indicator that the structure quality may be low
   5 ALERT type 4 Improvement, methodology, query or suggestion
   0 ALERT type 5 Informative message, check
```

It is advisable to attempt to resolve as many as possible of the alerts in all categories. Often the minor alerts point to easily fixed oversights, errors and omissions in your CIF or refinement strategy, so attention to these fine details can be worthwhile. In order to resolve some of the more serious problems it may be necessary to carry out additional measurements or structure refinements. However, the purpose of your study may justify the reported deviations and the more serious of these should normally be commented upon in the discussion or experimental section of a paper or in the "special_details" fields of the CIF. checkCIF was carefully designed to identify outliers and unusual parameters, but every test has its limitations and alerts that are not important in a particular case may appear. Conversely, the absence of alerts does not guarantee there are no aspects of the results needing attention. It is up to the individual to critically assess their own results and, if necessary, seek expert advice.

Publication of your CIF in IUCr journals

A basic structural check has been run on your CIF. These basic checks will be run on all CIFs submitted for publication in IUCr journals (*Acta Crystallographica*, *Journal of Applied Crystallography*, *Journal of Synchrotron Radiation*); however, if you intend to submit to *Acta Crystallographica Section C* or *E* or *IUCrData*, you should make sure that full publication checks are run on the final version of your CIF prior to submission.

Publication of your CIF in other journals

Please refer to the *Notes for Authors* of the relevant journal for any special instructions relating to CIF submission.

PLATON version of 05/12/2020; check.def file version of 05/12/2020

Datablock A21051O_014 - ellipsoid plot

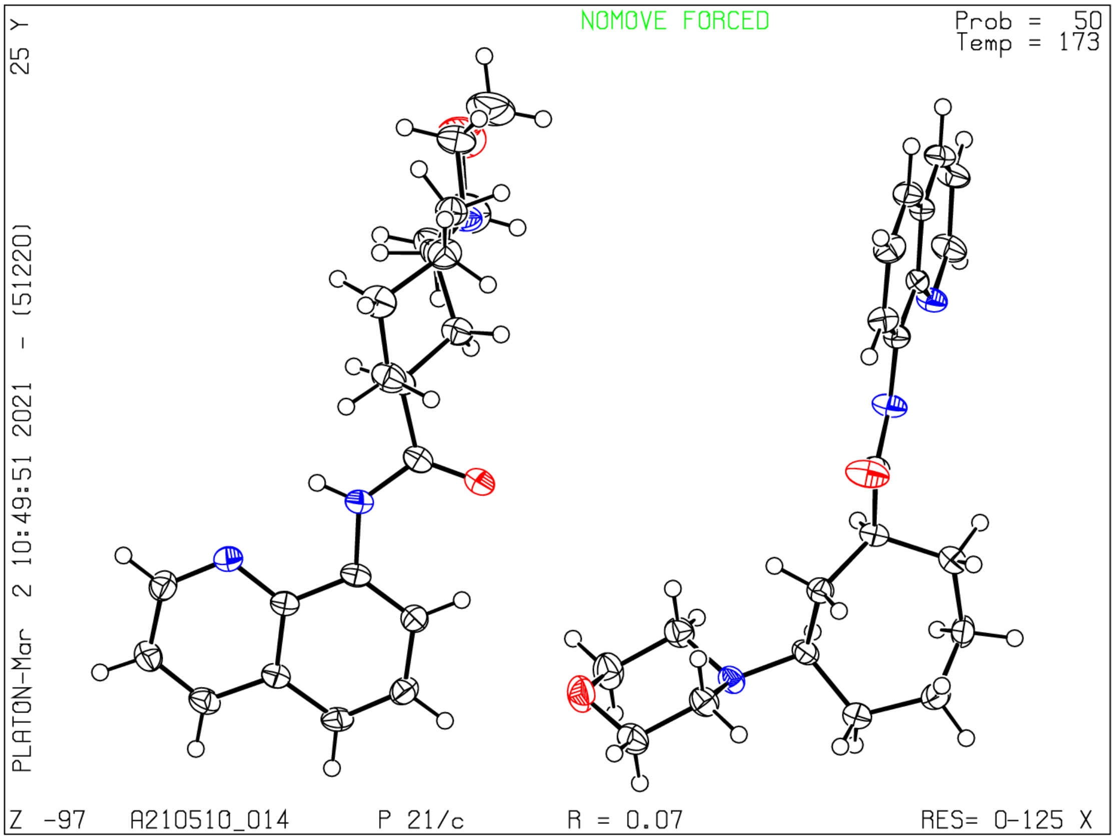

Supplement: Supplementary file 4 — Supplementary Data 2 [file 41467_2021_25696_MOESM4_ESM.pdf]
